# Enhanced Stereochemical Analysis of β‑Diastereomeric Amino Acids with Variants of Marfey’s Reagent

**DOI:** 10.1021/acsomega.5c07519

**Published:** 2025-10-24

**Authors:** Chloe I. Studinski, M. K. Powers, Brennan K. Martin, Angela L. Mosconi, Jacob A. Abraham, Kyle R. Koss, Samantha K. Bruffy, Meghan E. Campbell, Andrew R. Buller, Patrick H. Willoughby

**Affiliations:** † Department of Chemistry, 6902 Ripon College, 300 W. Seward Street, Ripon, Wisconsin 54971, United States; ‡ Department of Chemistry, University of Wisconsin–Madison, 1101 University Avenue, Madison, Wisconsin 53706, United States; § Department of Biochemistry, University of Wisconsin–Madison, 433 Babcock Drive, Madison, Wisconsin 53706, United States

## Abstract

The analysis of mixtures of amino acid stereoisomers is a classic challenge in bioorganic chemistry and organic synthesis. Chemical derivatization with Marfey’s reagent, 1-fluoro-2,4-dinitrophenyl-5-l-alanine amide, is frequently employed as chromatographic separation of the resulting diastereomers enables convenient assignment of d/l-configuration and determination of enantiomeric ratio. However, it is often challenging to resolve Cβ-epimeric diastereomers of noncanonical amino acids after treating with Marfey’s reagent. This report describes the effectiveness of alternative chiral derivatization reagents. We demonstrate that the Cβ epimers of l-phenylserine and other β-hydroxy noncanonical l-amino acids are more easily separated when the traditional Marfey’s reagent is substituted by a proline analogue or the d-enantiomers of several other Marfey’s reagents. Additionally, in many cases achiral Sanger’s reagent, 1-fluoro-2,4-dinitrobenzene, was found to produce the largest separation of β-diastereomers. The “mixed Marfey’s reaction” protocol is established, which allows for screening multiple Marfey’s reagents within a single reaction. This method was applied to the challenging case of resolving the methyl–ethyl Cβ stereocenter of l-isoleucine (Ile), providing accurate quantification of mixtures of l-Ile and l-*allo*-Ile. This strategy is applicable to a range of noncanonical amino acids and suggests that commercially available d-Marfey’s analogues or achiral Sanger’s reagent will provide enhanced separation without synthesis of more complex derivatizing agents.

## Graphical abstract

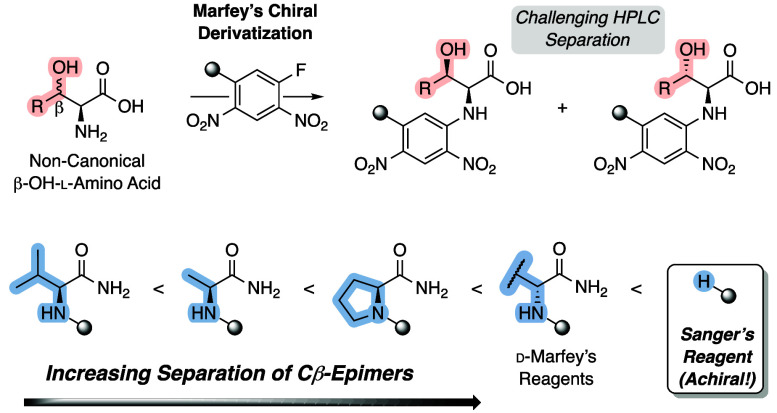

## Introduction

Noncanonical amino acids (ncAAs) are fundamental building blocks in chemical and biological synthesis.^1–4^ Recent advances in the medicinal chemistry of peptides and peptide-based therapeutics have driven a surge of interest in synthetic routes to ncAAs with structurally complex side chains.^5–7^ In many cases, these bespoke ncAAs contain side chains with additional stereogenic centers. Hence, the practical assessment of the stereochemical purity, such as diastereomeric ratio (dr) and enantiomeric excess (ee), of the amino acids on an analytical scale is essential for protein engineering and reaction optimization efforts. Direct methods often struggle to separate subtly different chiral amino acids, spurring the use of chiral auxiliaries to aid in separation. For decades, derivatization with Marfey’s reagent, 1-fluoro-2,4-dinitrophenyl-5-l-alanine amide (i.e., l-FDAA, **7**, Figure 1C), has served as the gold standard for such analysis.^8–10^ However, some ncAAs are poorly resolved by this compound,^11^ highlighting the need for new, streamlined procedures for rapid stereochemical analysis.

**Figure 1:**
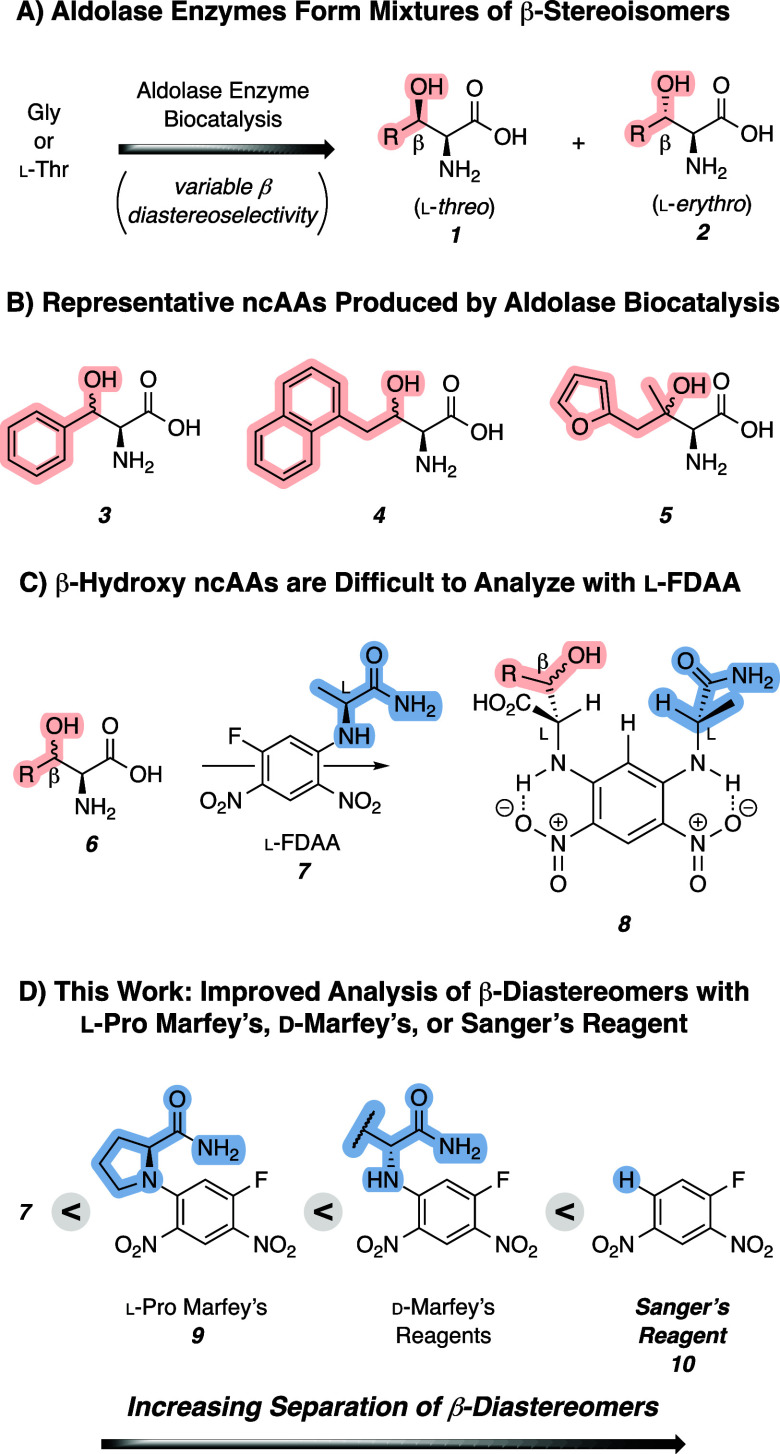
Noncanonical amino acids (ncAAs) produced by aldolase enzymes result in a mixture of β-stereoisomers that are difficult to resolve by the traditional Marfey’s analysis. (A) Aldolase enzymes form β-hydroxy ncAAs as a mixture of l-*threo* and l-*erythro* diastereomers. (B) Examples of β-hydroxy ncAA products produced by aldolase biocatalysts. (C) Derivatization of β-hydroxy ncAAs with l-FDAA forms a conformationally stable structure, but the β-stereocenter is remote from the hydrophobic arene, which reduces the ability to resolve the diastereomeric products. (D) Improved separation of the β-stereoisomers is observed if the l-FDAA is replaced with an l-Pro analogue, d-enantiomers of Marfey’s reagent, or the achiral Sanger’s reagent.

A variety of enzymatic strategies have been explored to build stereochemically complex ncAAs.^12^ For example, threonine aldolase^13–17^ and transaldolase^18–23^ enzymes construct analogues of l-threonine with additional functionality at the β-carbon (cf. Figure 1A,B). Recent progress in the application of these enzymes to biocatalytic systems has enabled the preparation of β-aryl (e.g., **3**),^24^ γ-aryl (e.g., **4**),^25^ and β,β-dialkyl (e.g., **5**)^26^ β-hydroxy ncAAs. Aldolase enzymes catalyze nucleophilic addition of a glycine enolate equivalent (i.e., glycyl quinoid), generated from glycine (Gly) or l-threonine (l-Thr), to a carbonyl electrophile. In accordance with Dunathan’s hypothesis,^27–29^ these pyridoxal phosphate-dependent enzymes ensure the α-amino chiral center is established with a high degree of fidelity for the l-isomer. However, the β-hydroxy stereocenter is formed with less configurational control,^17,24^ resulting in a mixture of l-*threo* and l-*erythro* diastereomers (cf. **1** and **2**, respectively). Additional examples include β-methyl phenylalanine analogues produced through innovative photobiocatalytic methods.^30,31^

Marfey’s chiral derivatization reaction is a practical and robust approach for analyzing stereoisomeric mixtures of amino acids.^32^ Numerous reports describe the application of Marfey’s reaction in various settings, such as assigning d/l stereochemistry at the α-carbon in natural and synthetic amino acids,^33^ determining the amino acid content of hydrolyzed peptides,^34^ and monitoring of both product yield and stereoselectivity in enzyme-catalyzed transformations.^24–26,30,31,35,36^ A general protocol for the Marfey’s reaction involves treating amino acid mixtures with the commercially available Marfey’s reagent, an amino amide derivatized with 1-fluoro-2,4-dinitrobenzene (e.g., **7**, Figure 1C). An amino acid substrate reacts with the reagent in an S_N_Ar reaction to give a 1,5-diamino-2,4-dinitrobenzene (cf. **8**). Because Marfey’s reagent contains a chiral center, reaction of a mixture of amino acid stereoisomers (e.g., **6**) results in diastereomeric products. These can be separated by routine reverse-phase chromatography to determine stereochemical purity of the original amino acid sample, and mass spectrometry detection enables low limits of detection.^37^ Separation is enhanced due to the hydrophobicity of the *meta*-dinitrobenzene moiety, which increases retention on C_18_ HPLC stationary phase. Furthermore, the derivatized structure strongly absorbs at ∼340 nm, allowing for quantification of the derivatized material. The d-enantiomer and racemic versions of Marfey’s reagent are available, allowing the absolute configuration to be established even when the (e.g., d-) enantiomer of the amino acid analyte is not readily available. Several analogues of Marfey’s reagent can be prepared, providing additional opportunities to resolve amino acid enantiomers.^10,32^

Previous work by Harada showed that Marfey’s adducts form intramolecular hydrogen bonds with a nitro oxygen (cf. **8**). This interaction produces a stable conformation with drastically different facial topologies depending on the relative configuration of the two α-carbons.^38^ The different faces of the α-amino diastereomers allow the Marfey’s adducts to be readily separated with routine HPLC instrumentation. As depicted in **8**, matching the α-stereocenters, such that an l-amino acid is derivatized with an l-Marfey’s reagent (and vice versa with the d-enantiomers), places the hydrophilic amide and carboxyl groups on opposite faces of the molecule. Derivatizing the d-enantiomer of the amino acid substrate with an l-Marfey’s reagent (and vice versa) results in mismatched α-stereocenters, which positions the hydrophilic groups on the same face of the molecule. Having the hydrophilic groups on the same face provides greater opportunity for the hydrophobic arene of the adduct to interact with the nonpolar HPLC stationary phase. In turn, this increases the retention time relative to the “matched” derivatives, resulting in a predictable elution order of matched followed by mismatched Marfey’s derivatives. As first observed by Marfey,^8^ l-amino acids are reported to elute faster than the d-enantiomers. This is often because the l-Marfey’s reagent is used, which forms a matched adduct with the l-amino acid, whereas the d-amino acids form the slower eluting mismatched adduct. Exceptions to this heuristic have been reported and usually occur when the side chains bear hydrophilic groups.^9^

Despite being epimeric diastereomers, we have observed that *threo* and *erythro* β-hydroxy-l-α-amino acids can be challenging to resolve under standard reverse phase conditions even after *N*-arylation with Marfey’s reagent **7**. For example, **4** and **5** required derivatization using more hydrophobic Marfey’s reagents, longer chromatography experiments, and ultraperformance liquid chromatography (i.e., UPLC) instrumentation.^25,26^ Considering Harada’s model, the β-stereocenter is likely too remote from the aryl core of the Marfey’s adduct to impose a substantial difference in the interaction of each of the β-epimers with C_18_ stationary phase. This report describes our evaluation of alternative Marfey’s reagents for their ability to resolve β-stereoisomers on HPLC instrumentation and reveals informative patterns to enable a general approach to determine suitable derivatization conditions for routine resolution of amino acid diastereomers (cf. Figure 1D).

## Results and Discussion

We have previously observed that the standard Marfey’s reagent (**7**) provided modest resolution of the β-diastereomers of several phenylserine analogues.^24^ With this in mind, we assessed the Marfey’s derivatization reaction on a synthetic sample of phenylserine (cf. Figure 2A), which was prepared as a mixture of all four stereoisomers where the d,l-*threo* isomers were the major products and d,l-*erythro* isomers were minor. When derivatized with **7**, four isomeric products were observed on HPLC/MS (cf. Figure 2B) corresponding with diastereomers **11**–**14**, indicating that the stereoisomers of phenylserine can be readily resolved. Based on literature precedence,^8,9^ the l-isomers eluted first, allowing us to assign the four peaks in order of elution as l-*erythro* **11**, l-*threo* **12**, d-*erythro* **13**, and d-*threo* **14**. Unsurprisingly, each of the α-stereoisomeric pairs was strongly resolved. However, the d-*threo* isomer eluted substantially slower than the other three isomers, resulting in a difference in retention time (i.e., Δ*t*_R_) for β-d-stereoisomers that was nearly 3-fold greater than that of its l counterpart.

**Figure 2:**
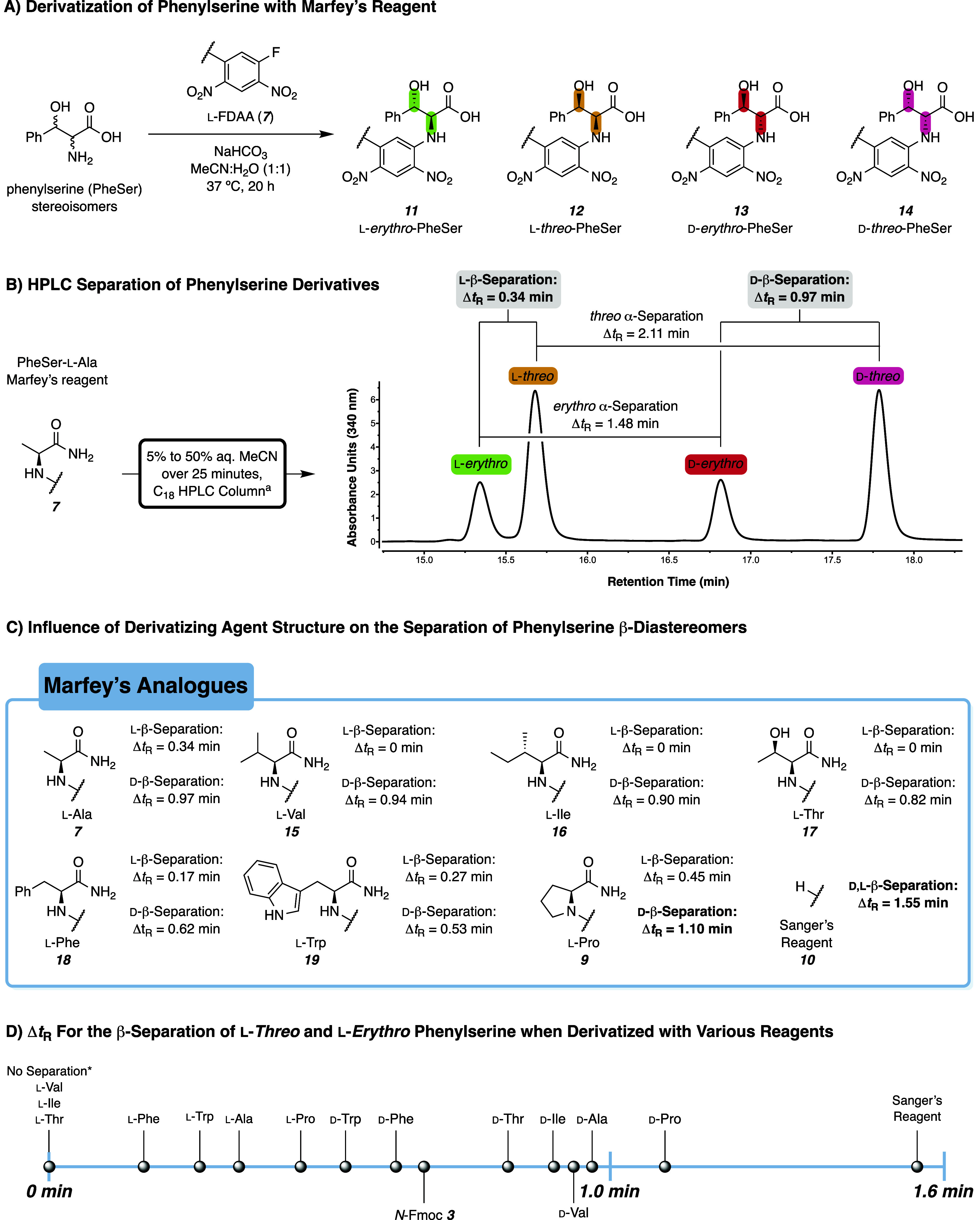
Evaluating Marfey’s reaction conditions for the separation of β-diastereomers of phenylserine (**3**) with C_18_ HPLC. (A) Marfey’s reaction of the stereoisomers of **3** produces four diastereomeric products. (B) VWD Chromatogram (340 nm) from HPLC separation of the stereoisomers of **3** derivatized with l-FDAA (**7**). The Δ*t*_R_ is the absolute difference in retention time at the peak maxima. (C) β-Separations^a^ of **3** when derivatized with Marfey’s reagent-bearing different amino amide side chains. The Δ*t*_R_ of the l-β-separation is the absolute difference in retention time at the peak maxima between the l-*erythro* and l-*threo* peaks. The Δ*t*_R_ of the d-β-separation is the absolute difference in retention time at the peak maxima between the d-*erythro* and d-*threo* peaks. (D) Separations^a^ of the l-β-diastereomers of **3** using l and d Marfey’s derivatization agents. Values for the separations with d-Marfey’s reagents are inferred from the d-β-separation data shown in Panel C. ^a^Conditions: Agilent Zorbax Extend-C18 column, 2.1 × 50 mm (1.8 μm), gradient of 5%–50% MeCN in H_2_O + 0.1% HCO_2_H over 25 min followed by 5 min column conditioning, flow rate 0.35 mL min^–1^.

Previous reports demonstrated that varying the chiral amino amide substituent on Marfey’s reagent could enhance stereoisomer resolution in certain contexts.^39^ With this in mind, we derivatized the stereoisomeric mixture of **3** with several analogues of Marfey’s reagent and measured the Δ*t*_R_ for the l and d β-separation (cf. Figure 2C). While all Marfey’s reagents could resolve the α-stereoisomers, only the alanine (**7**) and proline (**9**) reagents fully resolved the l-*threo* and l-*erythro* β-stereoisomers. Interestingly, valine-derived Marfey’s reagent **15**, which has been used on numerous occasions^40,41^ to enhance stereoisomer resolution, was incapable of resolving the β-l-diastereomers. Similarly, isoleucine (**16**) and threonine (**17**) analogues provided no resolution despite having a second stereocenter on the reagent, which could have enhanced resolution by providing more diastereomeric interactions during separation. Aryl-containing reagents **18** and **19** ^42,43^ provided improved l-β-separation compared with **15**–**17**, and the peaks were fully resolved like those observed with **7** and **9**. For all reagents, the d-*threo* isomers had a substantially longer Δ*t*_R_, enabling baseline resolution of the d-β-stereoisomeric pairs. It is particularly noteworthy that aliphatic reagents **15**–**17** offered no separation of l-β-diastereomers, but separation of the d-stereoisomers occurred to nearly the same degree as that of **7** and **9**. These data indicate that the additional hydrophobicity afforded to the mismatched derivatives (i.e., l-Marfey’s reagent + α-d-amino acids) enables baseline resolution of the β-epimers.

Because the *erythro* and *threo* forms of **3** are diastereomers, it is possible to resolve the isomers without chiral derivatization. However, analysis of the parent β-hydroxy amino acids (i.e., without precolumn derivatization) suggests that this class of compounds is too polar to enable retention and routine separation with standard C_18_ HPLC columns. This led us to consider achiral derivatization with Sanger’s reagent (i.e., 1-fluoro-2,4-dinitrobenzene, **10**),^44^ a truncated version of the Marfey’s reagent that does not include a chiral amine. HPLC analysis of the derivatized mixture revealed two peaks with a larger Δ*t*_R_ than all other Marfey’s reagents. In this instance, each of the peaks represent a racemic mixture of a β-stereoisomer where the *erythro* molecules elute first and are followed by the *threo* isomers. This demonstrates that the achiral variant of Marfey’s reagent provided the greatest separation of β-stereoisomers, with nearly a 5-fold increase in Δ*t*_R_ compared with that of the standard Marfey’s reagent. On its own, the chiral β stereocenter can sufficiently bias the different faces of the *N*-aryl amino acid without the need of an additional chiral moiety. Protecting the amino nitrogen as an Fmoc carbamate to make *N*-Fmoc **3** also enabled resolution of the β-stereoisomers, albeit with a reduced Δ*t*_R_ (i.e., 0.67 min) compared to Sanger’s reagent. The increased hydrophobicity of the Fmoc-protected amine likely increases retention on C_18_, providing greater opportunity to resolve the diastereomers. It is noteworthy that the Δ*t*_R_ was larger in the case of Fmoc phenylserine than the l-β-separation using any other Marfey’s reagent. When the HPLC gradient time was reduced from 25 to 10 min, derivatizing agents d-**7**, d-**9**, and **10** maintained sufficiently high Δ*t*_R_ (i.e., 0.40, 0.46, and 0.59 min, respectively) between the d-β-stereoisomers for useful peak resolution. This suggests that the trends observed under the 25 min HPLC gradients could be applied to shorter methods, enabling high-throughput stereochemical analysis.

Aldolase and transaldolase enzymes generally form β-hydroxy-α-amino acids with the l-configuration about the α-stereocenter. A useful feature of assessing a mixture of all phenylserine stereoisomers is that we can infer the effectiveness of derivatizing the commonly encountered l-amino acids with less available d-Marfey’s reagents. Specifically, the Δ*t*_R_ of the d-β-separations observed when the stereoisomer mixture of **3** was derivatized with l-Marfey’s reagents would be equivalent to those of the l-β-separations if treated with the d-Marfey’s reagents. With this in mind, we plotted the Δ*t*_R_ for the separation of l-*erythro* and l-*threo* phenylserine when treated with the l and d isomers of the aforementioned derivatizing agents (cf. Figure 2D). It is striking that none of the typical l-Marfey’s reagents proved more effective than their d-counterparts, suggesting that practitioners should consider using d-Marfey’s reagents when attempting to resolve β-stereoisomers of this important class of molecules. A similar observation was made by Pérez-Victoria and co-workers,^45^ where the separation of the Cβ-epimers of β-hydroxy-l-leucine was enhanced when derivatized with d-**15** (i.e., Δ*t*_R_ = 1.5 min) versus l-**15** (i.e., Δ*t*_R_ = 0.2 min). Furthermore, achiral derivatization with less expensive reagents may offer superior resolution of the β-stereoisomers as evidenced by the Sanger’s and Fmoc data.

The phenylserine separation data suggested that Sanger’s reagent (**10**), along with the d and l isomers of the proline- (**9**) and alanine- (**7**) derived Marfey’s reagents, have the greatest potential to separate amino acid β-stereoisomers. With this in mind, we screened these reagents on a series of diastereomeric mixtures of noncanonical β-hydroxy-l-amino acids produced under biocatalytic reaction conditions (cf. Figure 3). Consistent with the results for chemically synthesized phenylserine, β-separation of the l-β-diastereomers of **3** was greatest for Sanger’s reagent, followed by the d-enantiomers of the proline and alanine-derived Marfey’s reagents. This pattern held for the β-naphthyl (**20**) and 4-bromophenyl (**21**) β-hydroxy amino acids along with aliphatic *n*-pentyl (**23**) and *i*-butyl (**24**) variants. However, Sanger’s reagent offered no resolution for γ-arylated substrates (i.e., **5**, **25**, and **26**) and 4-trifloxyphenyl **22**. For **4** and **22**, d-proline Marfey’s reagent provided the widest Δ*t*_R_. Interestingly, in the case of tertiary alcohol-containing substrates (**5**, **25**, and **26**), l-enantiomers of the alanine and proline-derived reagents afforded the highest degree of peak separation despite eluting nearly 2 min faster (cf. Supporting Information) than the d-Marfey’s reagents. These results highlight that, while there are trends for effective separation, no single reagent is uniformly better.

**Figure 3:**
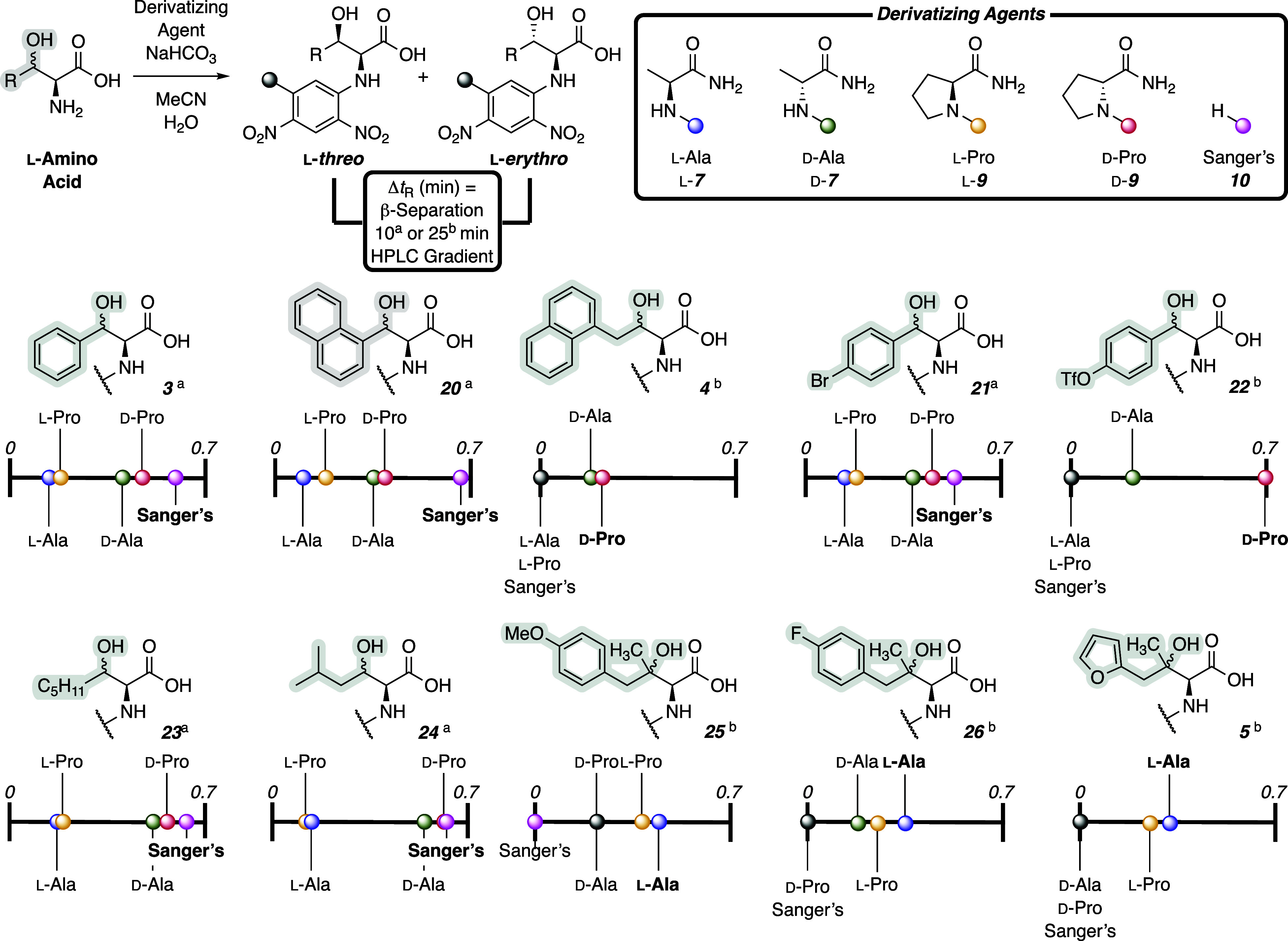
Evaluating the separation of l-β-diastereomers of different classes of β-hydroxy ncAAs with several derivatizing reagents. The Δ*t*_R_ of the l-β-separation is the absolute difference in retention time at the peak maxima between the l-*erythro* and l-*threo* peaks. ^a^Conditions: Agilent Zorbax Extend-C18 column, 2.1 × 50 mm (1.8 μm), gradient of 5%–50% MeCN in H_2_O + 0.1% HCO_2_H over 10 min followed by 5 min column conditioning, flow rate 0.35 mL min^–1^. ^b^Conditions: Agilent Zorbax Extend-C18 column, 2.1 × 50 mm (1.8 μm), gradient of 5%–50% MeCN in H_2_O + 0.1% HCO_2_H over 25 min followed by 5 min column conditioning, flow rate 0.35 mL min^–1^.

Harada’s model describes the importance of hydrogen bonding between an amino N–H and a nitro oxygen to establish conformational rigidity.^38^ With this in mind, the strong ability for the proline-containing Marfey’s reagent to resolve both the α and β-stereoisomers is surprising because the tertiary amine removes one of these crucial hydrogen bonds. This led us to consider alternative ring structures to see if the separation could be further influenced (cf. Figure 4). In addition to the l-Pro-NH_2_ reagent (**9**), we prepared the six (**28**) and four (**29**)-membered ring structures by treating the corresponding amino amide with 1,5-difluoro-2,4-dinitrobenzene (**27**). Derivatization of the stereoisomeric mixture of phenylserine with **28** and **29** allowed for detectable separation of all stereoisomers, albeit with reduced efficacy compared with **9**. Despite having a longer retention time than **9**, piperidine-containing **28** provided a reduced Δ*t*_R_ for both β-separations. In contrast to the other Marfey’s reagents, azetidine-containing **29** provided poor separation of the α-stereoisomers as the d adducts eluted nearly as fast as the l-isomers. However, the Δ*t*_R_ of the β-stereoisomers was similar to those of **9**, suggesting that **29** is still capable of efficiently resolving β-diastereomers of phenylserine. Collectively, these data demonstrate the utility of the proline-derived Marfey’s reagent with its greater ability to effectively resolve both the α- and β-stereoisomers, especially compared with cyclic Marfey’s reagents **28** and **29**. Additionally, the d and l amino amide needed to produce **9** are commercially available, enabling convenient access to the derivatizing agent in one synthetic step.

**Figure 4:**
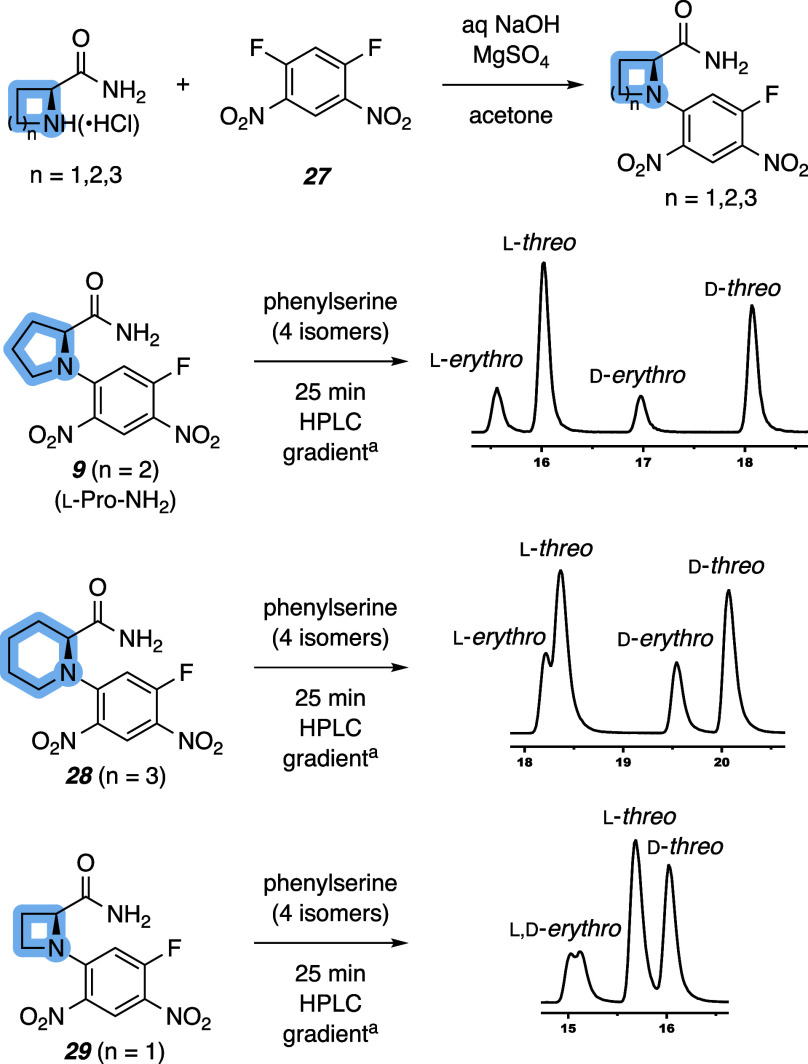
Synthesis and evaluation of the performance of cyclic amine-containing Marfey’s reagents in the separation of phenylserine. ^a^Conditions: Agilent Zorbax Extend-C18 column, 2.1 × 50 mm (1.8 μm), gradient of 5%–50% MeCN in H_2_O + 0.1% HCO_2_H over 25 min followed by 5 min column conditioning, flow rate 0.35 mL min^–1^.

The data included in this report reinforce the need to consider multiple types of derivatizing agents for each class of stereoisomers as different chiral environments on a derivatized analyte can greatly affect interaction with the C_18_ stationary phase. To streamline the screening of multiple Marfey’s reagents, we performed a multiplexed method scouting, which we call the “mixed Marfey’s reaction” (cf. Figure 5). Specifically, the stereoisomeric mixture of **3** was treated with the eight derivatizing agents shown in Figure 2 (i.e., **7**, **9**, **10**, **15**–**19**), each at one-eighth concentration used in a typical Marfey’s reaction (i.e., 0.6 mM). The reaction was otherwise treated like a typical “single” Marfey’s reaction, and we attempted separation of the derivatives using conventional C_18_ HPLC with a 25 min gradient. Despite producing highly complex total ion (cf. Figure 5A) and 340 nm absorption chromatograms, extracted ion chromatograms for each of the derivatized phenylserine stereoisomers allowed us to readily assess the ability for each of the derivatizing agents to resolve the stereoisomers of phenylserine (cf. Figure 5A black chromatogram snippets). Comparison of the extracted ion chromatograms from the single Marfey’s reaction (cf. gray chromatogram snippets) shows that the retention time of the derivatized stereoisomers is unchanged between the single and mixed Marfey’s reaction. Despite reducing the concentration of each Marfey’s reagent, the area of the derivatized stereoisomers was similar to those observed in the single Marfey’s reaction, with the exception of the l-Pro Marfey’s. Furthermore, when nine additional amino acids were added to the mixed Marfey’s reaction mixture, the retention times and degree of stereoisomer separation was unaffected (cf. Supporting Information). This suggests the method could be applied to mixtures of numerous amino acids that result from peptide or protein hydrolysis. Collectively, these data suggest that one can determine the efficacy of numerous chiral derivatizing reagents in a single experiment using the mixed Marfey’s reaction conditions.

**Figure 5:**
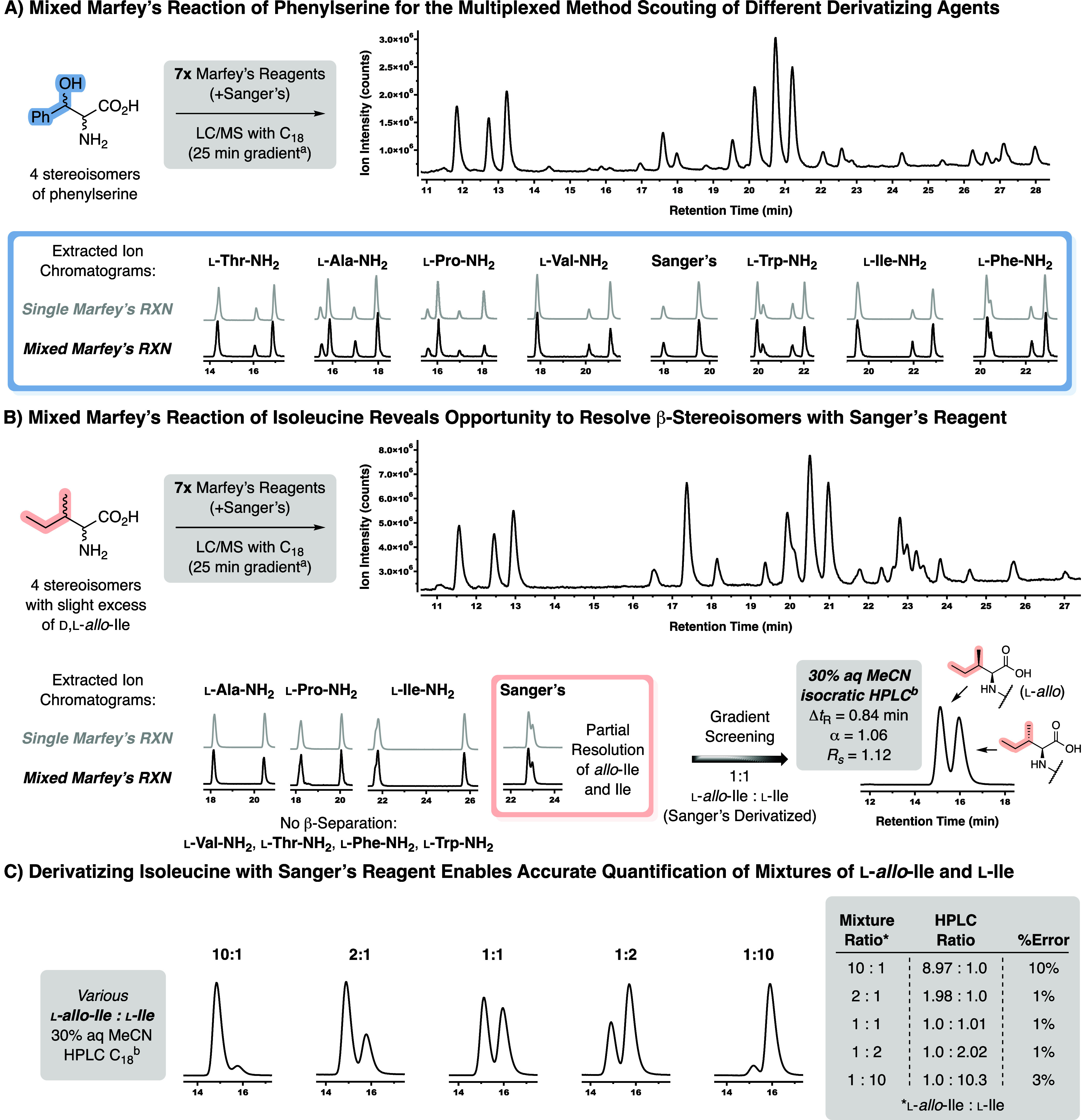
“Mixed Marfey’s reaction” for the multiplexed method scouting of different chiral derivatizing agents. (A) Comparing mixed Marfey’s reaction to the typical Marfey’s reaction protocol of phenylserine demonstrates that separation efficiency is not compromised if several derivatizing agents are added in a single experiment. (B) Mixed Marfey’s reaction of isoleucine stereoisomers reveals that Sanger’s reagent is most able to separate the β-methyl diastereomers. The Δ*t*_R_ is the absolute difference in retention time at the peak maxima. The α = (*t*_RA_ – *t*_0_)/(*t*_RB_ – *t*_0_), and Rs = 1.18 * (*t*_RA_ – *t*_RB_)/(*W*_0.5hA_ + *W*_0.5hB_) where *t*_0_ is the void volume, *W*_0.5h_ is the peak width at half-height, and A and B are the slower and faster eluting analytes, respectively. (C) Sufficient separation of Sanger’s-derived l-isoleucine and l-*allo*-isoleucine is possible with an isocratic HPLC method to enable accurate assessment of dr for varying ratios of stereoisomers. The HPLC Ratio was determined from the VWD (340 nm) integrations. ^a^Conditions: Agilent Zorbax Extend-C18 column, 2.1 × 50 mm (1.8 μm), gradient of 5%–50% MeCN in H_2_O + 0.1% HCO_2_H over 25 min followed by 5 min column conditioning, flow rate 0.35 mL min^–1^. ^b^Conditions: Agilent Zorbax Extend-C18 column, 2.1 × 50 mm (1.8 μm), isocratic 30% MeCN in H_2_O + 0.1% HCO_2_H over 30 min, flow rate 0.35 mL min^–1^.

Having established that the mixed Marfey’s reaction protocol will afford reliable separations for the stereoisomers of phenylserine, we were curious if the method would allow us to identify a derivatizing agent that would enable the resolution of the β-stereoisomers of isoleucine on routine HPLC instrumentation (cf. Figure 5B). Mixtures of isoleucine and its β-stereoisomer, *allo*-isoleucine, are notoriously difficult to analyze.^34,46–48^ Employing the mixed Marfey’s reaction protocol, we were able to screen seven Marfey’s reagents and Sanger’s reagent within a single run. By using both the l and d series of isoleucine stereoisomers, we were able to simultaneously evaluate the enantiomer of each of the Marfey’s reagents, bringing the total to 15 β-stereoisomer separations. Extracted ion chromatograms (i.e., black chromatogram snippets) revealed that all derivatizing reagents easily resolved the α-stereoisomers and comparing the retention times and areas to the single Marfey’s reaction (i.e., gray snippets) once again demonstrated that mixing several derivatizing reagents into a single reaction will not affect the individual separations. Of the numerous Marfey’s reagents screened, only the Ile-derived reagent (i.e., l-Ile-NH_2_) provided detectable separation of the l-Cβ-stereoisomers. However, Sanger’s reagent once again proved to be most capable, showing a Δ*t*_R_ of 0.18 min with the d,l-*allo* isomers eluting faster. It is interesting that Fmoc protection, another achiral derivatization, did not enable resolution of the β-diastereomers of isoleucine (cf. Supporting Information). This insight from the mixed Marfey’s reaction allowed us to move forward and identify alternative HPLC conditions to improve resolution and enable accurate quantification of the diastereomeric ratio. An isocratic method of 30% aqueous acetonitrile provided improved separation [i.e., Δ*t*_R_ = 0.84 min, resolution (*R*_S_) = 1.13, separation factor (α) = 1.06] on a practical time scale (i.e., elution in ∼15 min). Similarly, it has been reported that C_8_ stationary phase can separate l-Ile and l-*allo*-Ile (Δ*t*_R_ = 0.9 min) using an isocratic method (i.e., 22% aq. MeCN) when derivatized with the valine-derived Marfey’s reagent.^49^ Despite not being fully resolved, we were able to accurately evaluate the stereoisomer ratio for several premixed solutions of l-Ile and l-*allo*-Ile (cf. Figure 5C), where the average deviation from the experimental ratio was less than 5%. These data are particularly noteworthy because they indicate that those interested in evaluating diastereomeric ratio of mixtures of Ile and *allo*-Ile, a classic challenge in amino acid analysis,^48^ need only turn to the use of a readily available achiral derivatizing agent and a simple isocratic HPLC method.

## Conclusion

Since its discovery more than 40 years ago, Marfey’s chiral derivatization reaction has been invaluable to the study of amino acid structure and stereochemistry. Despite its widespread success in the evaluation of α-stereochemistry, chromatographic separation of Marfey’s adducts often proves challenging for β-diastereomeric ncAAs. To address this shortcoming, we have evaluated alternative derivatizing agents, demonstrating that many β-hydroxy ncAA diastereomers are best resolved with the achiral, hydrophobic arene, Sanger’s reagent. Where Sanger’s reagent was ineffective, d-enantiomers of FDAA and the proline-derived Marfey’s reagent proved capable. It was only in the case of β-tertiary alcohol ncAAs that the standard reagent, l-FDAA, was superior. To aid in determining an optimal Marfey’s reagent for other classes of molecules, we demonstrate a multiplexed screening approach, the “mixed Marfey’s reaction,” whereby several derivatizing agents are mixed in a single experiment and HPLC coupled with mass spectrometry enables efficient evaluation of separation by each individual reagent. The mixed Marfey’s reaction was used to establish Sanger’s reagent as being an effective reagent for the separation of β-diastereomers of isoleucine, allowing for accurate quantification of mixtures of l-isoleucine and l-*allo*-isoleucine with common HPLC instrumentation. These analytical strategies may also be more generally applicable to other classes of ncAAs and chiral amines,^35,36^ providing a useful approach for analyzing complex mixtures of amino acids resulting from samples of hydrolyzed proteins or peptides.

## Supplementary Material



## Notes

Expanded experimental details; procedures for the synthesis of all new compounds; spectral data used for the characterization of all new compounds; raw HPLC-MS data; and analyses of all HPLC-MS data (PDF)
