# Supplementary material for: Enhanced Stereochemical Analysis of β‑Diastereomeric Amino Acids with Variants of Marfey’s Reagent
Source: ACS Omega. 2025 Oct 24;10(43):51677–85. doi: 10.1021/acsomega.5c07519 (PMC12593104; doi:10.1021/acsomega.5c07519)
Supplement: Supplementary file 1 [file ao5c07519_si_001.pdf]

Supporting Information for:

Enhanced Stereochemical Analysis of β-Diastereomeric Amino Acids with Variants of Marfey's Reagent

Chloe I. Studinski[1], M. K. Powers[1], Brennan K. Martin[1], Angela L. Mosconi[1], Jacob A. Abraham[1], Kyle R. Koss[1], Samantha K. Bruffy[2], Meghan E. Campbell[2], Andrew R. Buller[2,3*], Patrick H. Willoughby[1*]

[1]Department of Chemistry, Ripon College, 300 W. Seward Street, Ripon, Wisconsin 54971, United States

[2]Department of Chemistry, University of Wisconsin−Madison, 1101 University Avenue, Madison, Wisconsin 53706, United States

[3]Department of Biochemistry, University of Wisconsin−Madison, 433 Babcock Drive, Madison, Wisconsin 53706, United States

*To whom correspondence should be addressed: Patrick H. Willoughby (willoughbyp@ripon.edu), Andrew R. Buller (arbuller@wisc.edu)

Contents of Supporting Information

I. General Experimental Details **S2**

II. Synthesis of Variants of the Marfey's Reagent **S3–S12**

A. General Procedure A: Synthesis of Marfey's Reagents S3
B. Details on the Preparation and Characterization of All New Compounds S3

III. Derivatization and Separation of Amino Acid Stereoisomers with Various Marfey's Reagents **S13–S198**

A. HPLC Methods Used for the Separation of Derivatized Amino Acids S13
B. Procedures for Derivatization and Separation of a Stereoisomeric Mixture of Phenylserine with Various Marfey's Reagents S13
C. Raw LC/MS Data for the Separation of Stereoisomeric Mixtures of Derivatized Amino Acids S14
D. Mixed Marfey's Reaction for the Multiplexed Method Scouting of Different Chiral Derivatizing Agents in the Resolution of the Four Stereoisomers of Phenylserine S143
E. Comparison of the Mixed Marfey's Reaction on a Pure Sample Versus a "Complex" Sample of Phenylserine Diastereomers S152
F. Mixed Marfey's Reaction for the Multiplexed Method Scouting of Different Chiral Derivatizing Agents in the Resolution of the Four Stereoisomers of Isoleucine S161
G. Separation of a Stereoisomeric Mixture of Isoleucine with Various Marfey's Reagents S170
H. Separation of Various Mixtures of L-*allo*-Isoleucine and L-Isoleucine S188

IV. References for Supporting Information **S199**

I. General Experimental Details

Reagents and reactants were purchased from Sigma Aldrich, AK Scientific, Oakwood Chemical, Strem Chemicals, and Ambeed and were used without further purification unless otherwise noted. NMR spectra were recorded on a Bruker Ascend Evo 400 MHz spectrometer. ^{1}H NMR chemical shifts are reported in ppm (δ) relative to the solvent resonance for DMSO-d_6 (δ 2.50 ppm). ^{13}C and ^{19}F NMR spectra were acquired with ^{1}H decoupling. ^{13}C NMR chemical shifts are reported in ppm (δ) relative to the solvent resonance for DMSO-d_6 (δ 39.52 ppm). ^{19}F NMR spectra were acquired in DMSO-d_6 with hexafluorobenzene as an internal standard and referenced to δ -164.90 ppm.[1] Data are reported as follows: chemical shift (multiplicity [singlet (s), doublet (d), doublet of doublets (dd), multiplet (m)], coupling constants [Hz], integration). All NMR spectra were recorded at ambient temperature.

High-resolution mass spectrometry (HRMS) measurements were performed on an Agilent 6230 TOF LC/MS accurate-mass time-of-flight (ToF) instrument, using electrospray ionization (ESI) calibrated against purine and hexakis(1H,1H,3H-perfluoropropoxy)phosphazene. In the positive mode, purine is 121.050873 m/z $[M + H]^+$ and hexakis(1H,1H,3H-perfluoropropoxy)phosphazene is 922.009798 m/z $[M + H]^+$. In the negative mode, purine is 119.036320 m/z $[M - H]^-$ and the formate adduct of hexakis(1H,1H,3H-perfluoropropoxy)phosphazene is 966.000725 m/z $[M + HCO_2]^-$. Samples were introduced as solutions in 50% aq. MeCN by injecting through an Agilent 1260 Infinity HPLC system either 1) without a column equipped (i.e., no-column injection) or 2) using an Agilent Zorbax Extend-C18 column, 2.1 x 50 mm (1.8 µm). The Agilent column part number is 727700-902.

Specific rotation values were acquired on a Rudolph AUTOPOL III Automatic Polarimeter at two wavelengths, 589 nm (i.e., $[\boldsymbol{\alpha}]_{\boldsymbol{D}}$) and 546 nm (i.e., $[\boldsymbol{\alpha}]_{\mathbf{546}}$). Analytes were loaded as solutions in acetone (i.e., 0.5 g / 100 mL or c = 0.5) into sample cells with a path length of 50 mm and volume of 1 mL. The specific rotation values were obtained as follows.

$$[\alpha]_D^T \ or \ [\alpha]_{546}^T = \frac{\alpha}{c \bullet d}$$

Where T is the temperature (ºC), α is the observed rotation at 589 nm or 546 nm, c is the concentration (i.e., 0.5 g / 100 mL), and d is the path length of the sample cell in dm (i.e., 0.5 dm).

Infrared spectra were acquired on an Agilent Cary 630 FT-IR using neat samples. The most intense and/or diagnostic peaks are reported. Melting points for solid products were recorded without correction.

II. Synthesis of Variants of the Marfey's Reagent

II-A. General Procedure A: Synthesis of Marfey's Reagents

The previously reported Marfey's reagents were prepared from 1,5-difluoro-2,4-dinitrobenzene (**27**) and the corresponding amino amide by the following established[2,3] method. Specifically, 1 M aqueous NaOH (4.0 mL, 4.0 mmol) was added to the amino amide hydrochloride (3.7 mmol) at room temperature and with stirring. After the mixture became homogenous (<5 minutes), acetone (60 mL) and magnesium sulfate (8.6 g, 72 mmol) were added. After 2 h, a solution of 1,5-difluoro-2,4-dinitrobenzene (**27**, 667 mg, 3.3 mmol) in acetone (15 mL) was added dropwise over five minutes. After 25 minutes, the mixture was filtered to remove the magnesium sulfate and diluted with water (80 mL) to induce product precipitation. The mixture was frozen in a -20 ºC freezer, thawed, and vacuum filtered. The solid was rinsed with water (3x 5 mL) and diethyl ether (3x 5 mL), and residual solvent was removed from the product using rotary evaporation.

Spectroscopic data of the known Marfey's reagents matched previous reports (i.e., manuscript Figure 2C: Ala **7**,[2] Val **15**,[4] Phe **18**,[4] Trp **19**,[5] and Pro **9**[6]-derived Marfey's reagents).

II-B. Details on the Preparation and Characterization of All New Compounds

(2*S*,3*S*)-2-((5-Fluoro-2,4-dinitrophenyl)amino)-3-methylpentanamide (16)

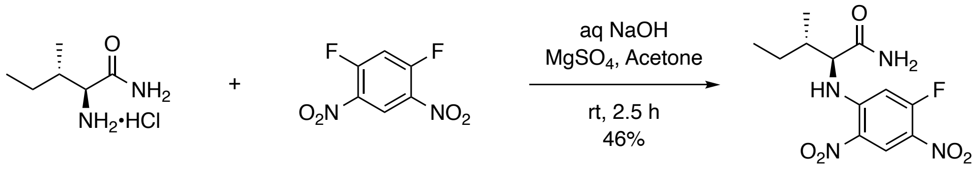

The L-Ile analogue of Marfey's reagent (i.e., **16**) was prepared following General Procedure A by using (2*S*,3*S*)-2-amino-3-methylpentanamide hydrochloride (**S1**, 610 mg, 3.7 mmol). Product **16** was recovered as a yellow solid (478 mg, 1.52 mmol, 46%).

^{1}H NMR (400 MHz, DMSO-d_6) δ 8.89 (d, *J* = 8.1 Hz, 1H), 8.82 (d, *J* = 8.2 Hz, 1H), 7.67 (s, 1H), 7.47 (s, 1H), 7.02 (d, *J* = 14.4 Hz, 1H), 4.24 (dd, *J* = 8.3, 5.9 Hz, 1H), 2.04 – 1.91 (m, 1H), 1.64 – 1.52 (m, 1H), 1.25 – 1.11 (m, 1H), 0.94 (d, *J* = 6.8 Hz, 3H), and 0.90 (t, *J* = 7.3 Hz, 3H).

^{13}C{^{1}H} NMR (101 MHz, DMSO-d_6) δ 171.0, 159.3 (d, *J* = 266.7 Hz), 148.6 (d, *J* = 14.1 Hz), 127.6, 127.5, 125.3 (d, *J* = 9.6 Hz), 102.0 (d, *J* = 27.5 Hz), 61.0, 37.1, 24.5, 15.2, and 11.4.

^{19}F{^{1}H} NMR (376 MHz, DMSO-d_6) δ -109.2.

HRMS (ESI$^-$): Calcd for $C_{12}H_{14}FN_4O_5^-$ [M – H]$^-$ requires 313.0954; found 313.0976.

$[\alpha]_D^{24}$ = -2.8º (c = 0.5, acetone); $[\alpha]_{546}^{24}$ = -1.6º (c = 0.5, acetone)

FTIR (neat): 3444, 3310, 3198, 3109, 2967, 1670, 1640, 1580, 1416, 1327, 1282, 1118, 1044, 835, and 738 cm^{-1}.

MP: 208 – 210 ºC.

Figure S1: ^{1}H NMR (400 MHz) spectrum of **16** in DMSO-d_6

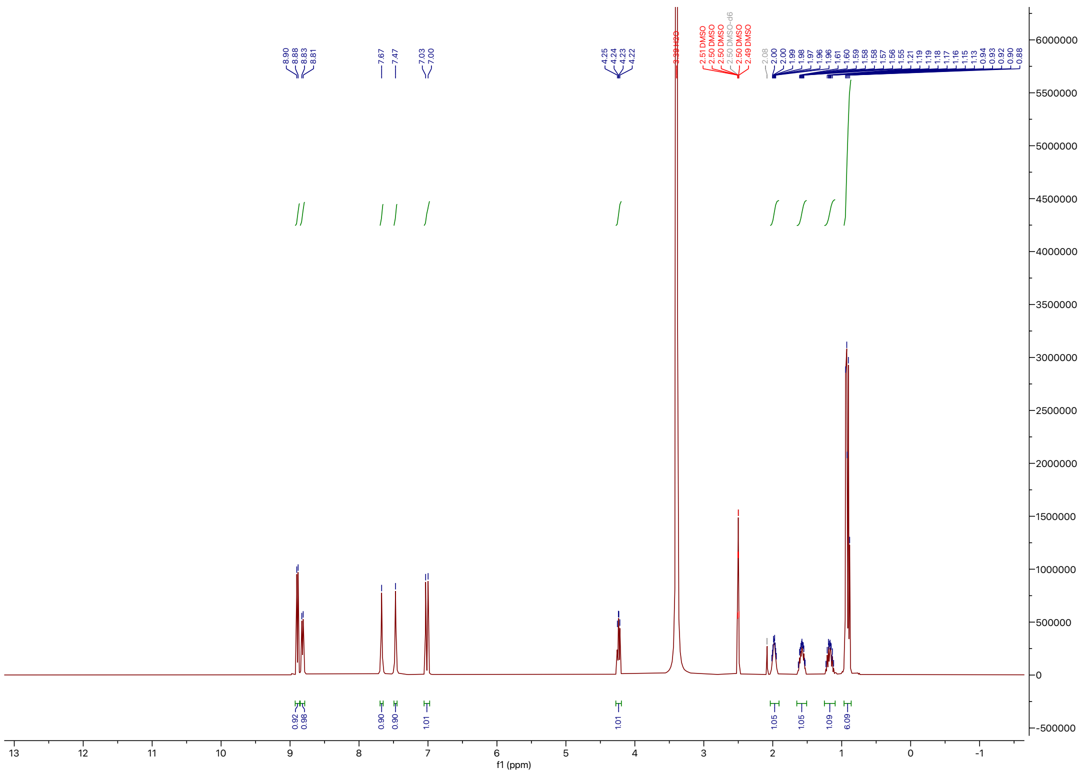

Figure S2: ^{13}C NMR (101 MHz) spectrum of **16** in DMSO-d_6

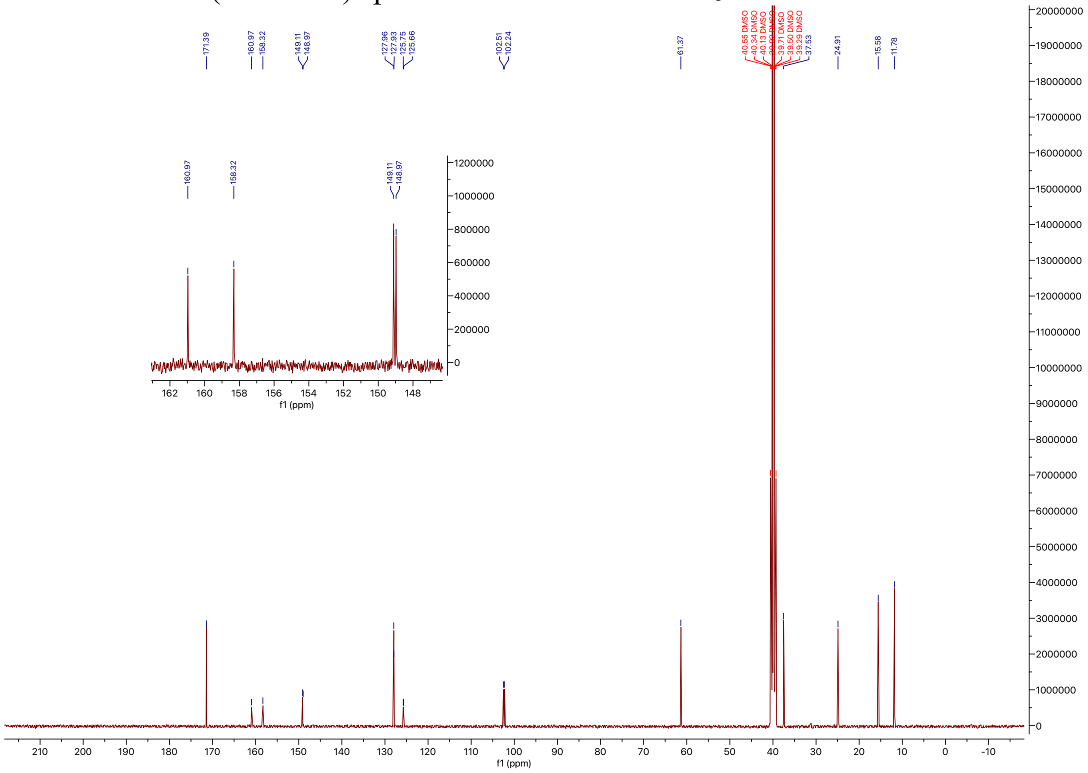

Figure S3: ^{19}F NMR (376 MHz) spectrum of **16** in DMSO-d_6 referenced to hexafluorobenzene internal standard (δ -164.9 ppm).

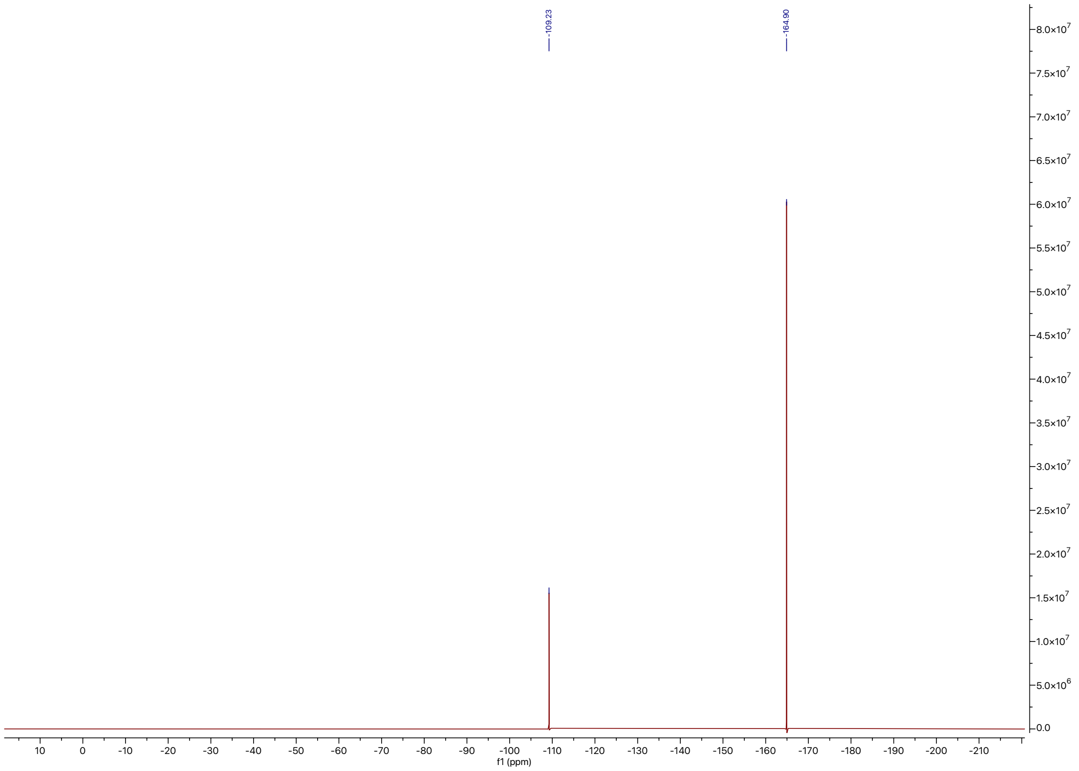

(2*S*,3*R*)-2-((5-Fluoro-2,4-dinitrophenyl)amino)-3-hydroxybutanamide (17)

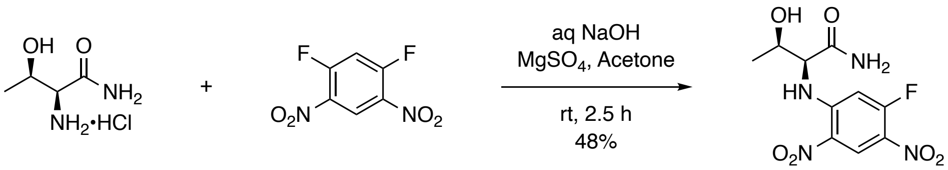

S2 ***27*** ***17***

The L-Thr analogue of Marfey's reagent (i.e., **17**) was prepared following General Procedure A using (2*S*,3*R*)-2-amino-3-hydroxybutanamide hydrochloride (**S2**, 586 mg, 3.80 mmol). Product **17** was recovered as a yellow solid (511 mg, 1.69 mmol, 48%).

^{1}H NMR (400 MHz, DMSO-d_6): δ 9.02 (d, J = 7.6 Hz, 1H), 8.90 (d, J = 8.0 Hz, 1H), 7.58 (s, 1H), 7.40 (s, 1H), 7.00 (d, J = 14.5 Hz, 1H), 5.53 (d, J = 4.1 Hz, 1H), 4.28 – 4.20 (m, 2H), and 1.15 (d, J = 6.0 Hz, 3H).

^{13}C{^{1}H} NMR (101 MHz, DMSO-d_6) δ 170.6, 159.0 (d, J = 266.3 Hz), 149.2 (d, J = 14.1 Hz), 127.8, 127.2, 125.1 (d, J = 9.6 Hz), 102.2 (d, J = 27.4 Hz), 66.3, 62.2, and 20.6.

^{19}F{^{1}H} NMR (376 MHz, DMSO-d_6) δ -110.0.

HRMS (ESI$^-$): Calcd for $C_{10}H_{10}FN_4O_6^-$ [M – H]$^-$ requires 301.0590; found 301.0608.

$[\boldsymbol{\alpha}]_{\boldsymbol{D}}^{\mathbf{23}}$ = 16.4º (c = 0.5, acetone); $[\boldsymbol{\alpha}]_{\mathbf{546}}^{\mathbf{23}}$ = 24.4º (c = 0.5, acetone)

FTIR (neat): 3429, 3310, 1633, 1573, 1401, 1275, 1238, 1215, 1088, 1044, 850, and 716 cm^{-1}.

MP: 216–217 ºC.

Figure S4: ^{1}H NMR (400 MHz) spectrum of **17** in DMSO-d_6

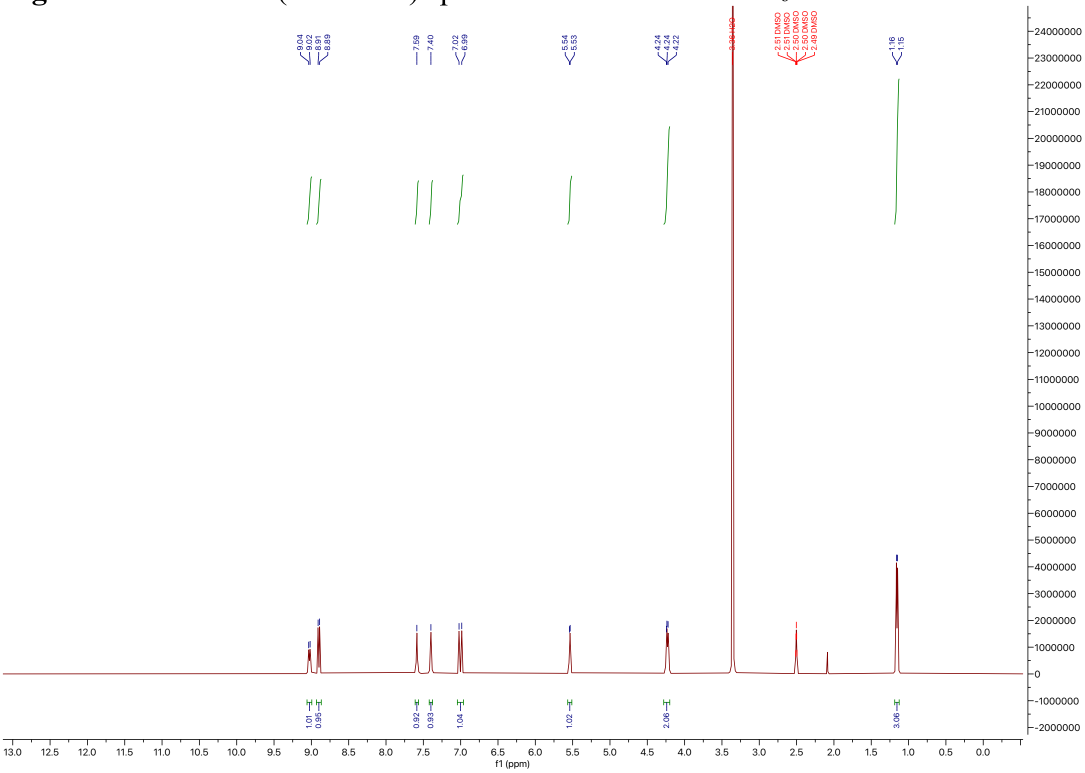

Figure S5: ^{13}C NMR (101 MHz) spectrum of **17** in DMSO-d_6

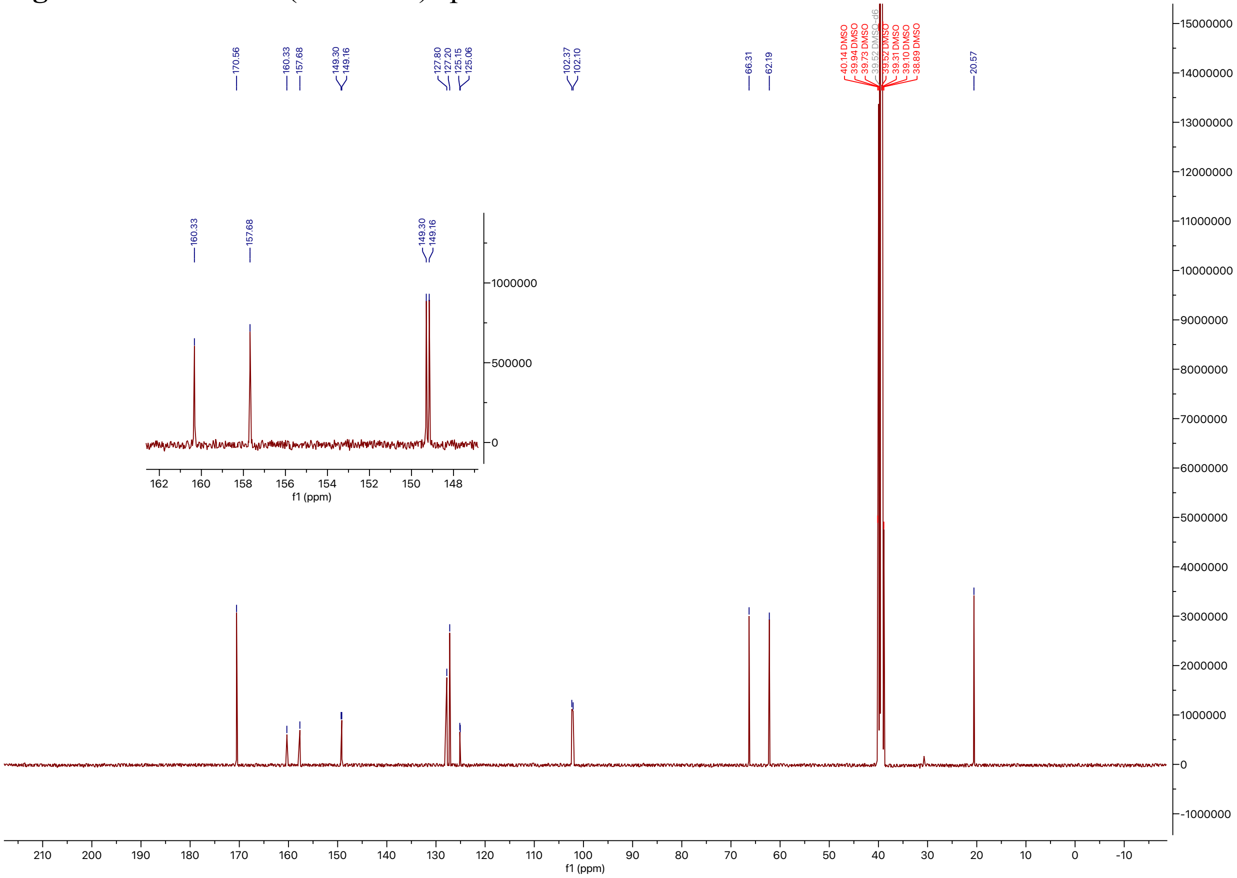

Figure S6: ^{19}F NMR (376 MHz) spectrum of **17** in DMSO-d_6 referenced to hexafluorobenzene internal standard (δ -164.9 ppm).

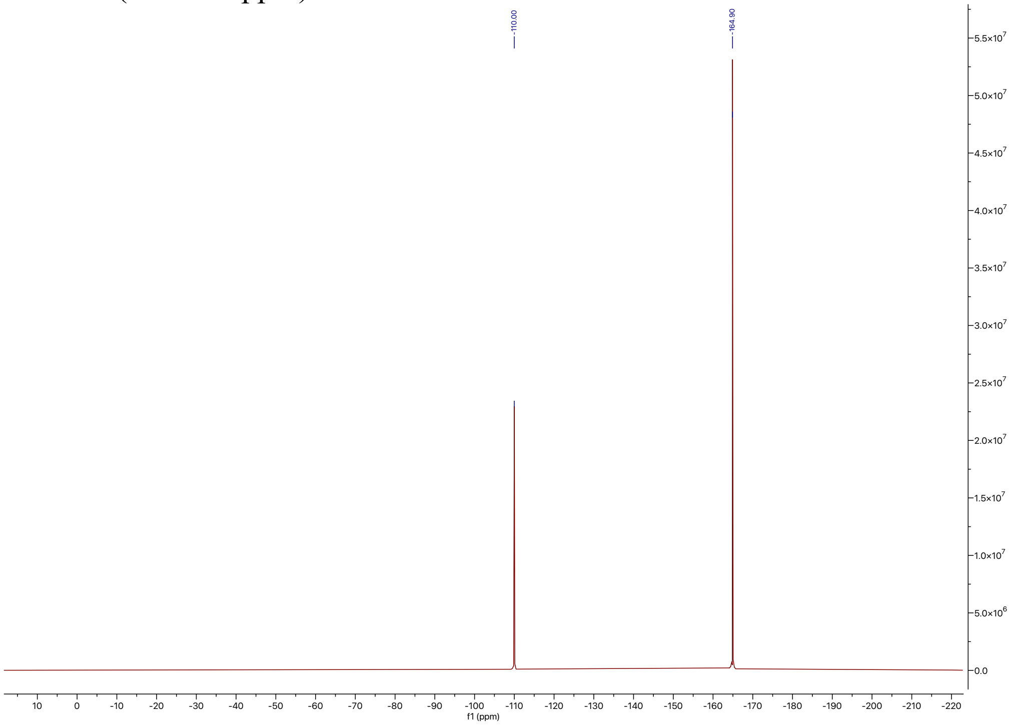

(*S*)-1-(5-Fluoro-2,4-dinitrophenyl)piperidine-2-carboxamide (28)

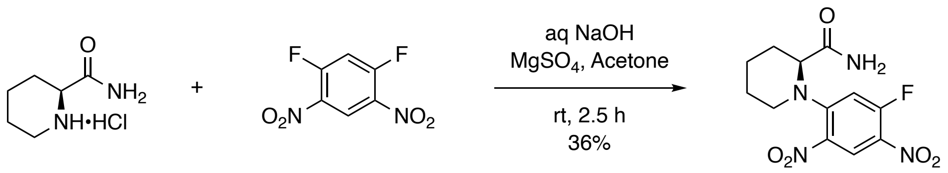

S3 ***27*** ***28***

(*S*)-Piperidine-2-carboxamide can be purchased, but we found it more cost-effective to treat methyl (*S*)-piperidine-2-carboxylate hydrochloride[7] (i.e., **S3**) with concentrated aqueous ammonia (5 mL ammonia per gram of substrate) with stirring at room temperature. After 20 h, the mixture was concentrated, and the resulting solid was used as is for the preparation of **28**.

The piperidine-containing analogue of Marfey's reagent (i.e., **28**) was prepared following General Procedure A by using (*S*)-piperidine-2-carboxamide hydrochloride (**S3**, 608 mg, 4.8 mmol). Product **28** was recovered as a yellow solid (372 mg, 1.19 mmol, 36%).

^{1}H NMR (400 MHz, DMSO-d_6) δ 8.62 (d, J = 8.2 Hz, 1H), 7.37 (s, 1H), 7.32 (s, 1H), 7.23 (d, J = 14.8 Hz, 1H), 4.32 – 4.27 (m, 1H), 3.47 (td, J = 12.7, 3.0 Hz, 1H), 3.10 (d, J = 13.3 Hz, 1H), 2.20 (d, J = 14.2 Hz, 1H), 1.83 – 1.71 (m, 1H), 1.69 – 1.60 (m, 2H), 1.59 – 1.47 (m, 1H), and 1.44 – 1.30 (m, 1H).

^{13}C{^{1}H} NMR (101 MHz, DMSO-d_6) δ 171.1, 157.5 (d, J = 266.0 Hz), 151.0 (d, J = 12.7 Hz), 133.7, 126.8, 126.5 (d, J = 9.1 Hz), 108.3 (d, J = 25.8 Hz), 60.3, 49.2, 27.2, 24.1, and 19.6.

^{19}F{^{1}H} NMR (376 MHz, DMSO-d_6) δ -111.8.

HRMS (ESI$^-$): Calcd for $C_{12}H_{12}FN_4O_5^-$ [M – H]$^-$ requires 311.0797; found 311.0794.

$[\alpha]_D^{24}$ = -340.8° (c = 0.5, acetone); $[\alpha]_{546}^{24}$ = -228.4 (c = 0.5, acetone)

FTIR (neat): 3474, 3370, 3049, 2930, 1685, 1610, 1573, 1506, 1439, 1327, 1305, 1275, 1245, 1208, 1118, 1066, 999, 906, and 716 cm^{-1}.

MP: 176 – 178 °C (decomposed).

Figure S7: ^{1}H NMR (400 MHz) spectrum of **28** in DMSO-d_6

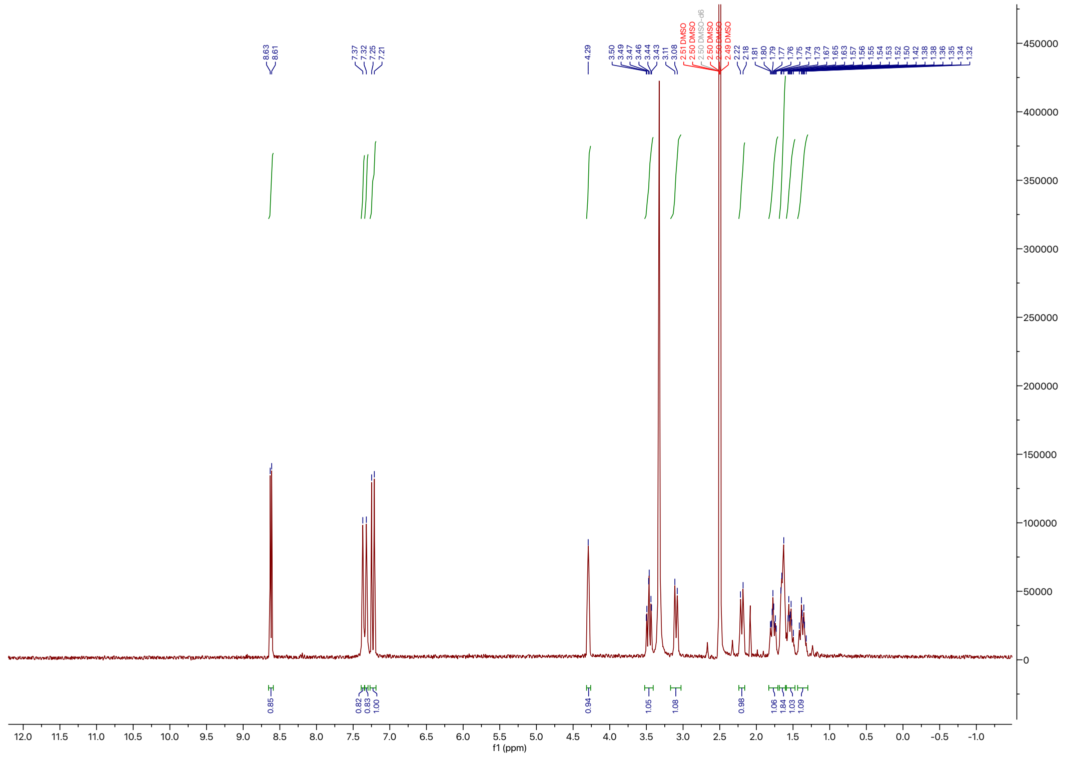

Figure S8: ^{13}C NMR (101 MHz) spectrum of **28** in DMSO-d_6

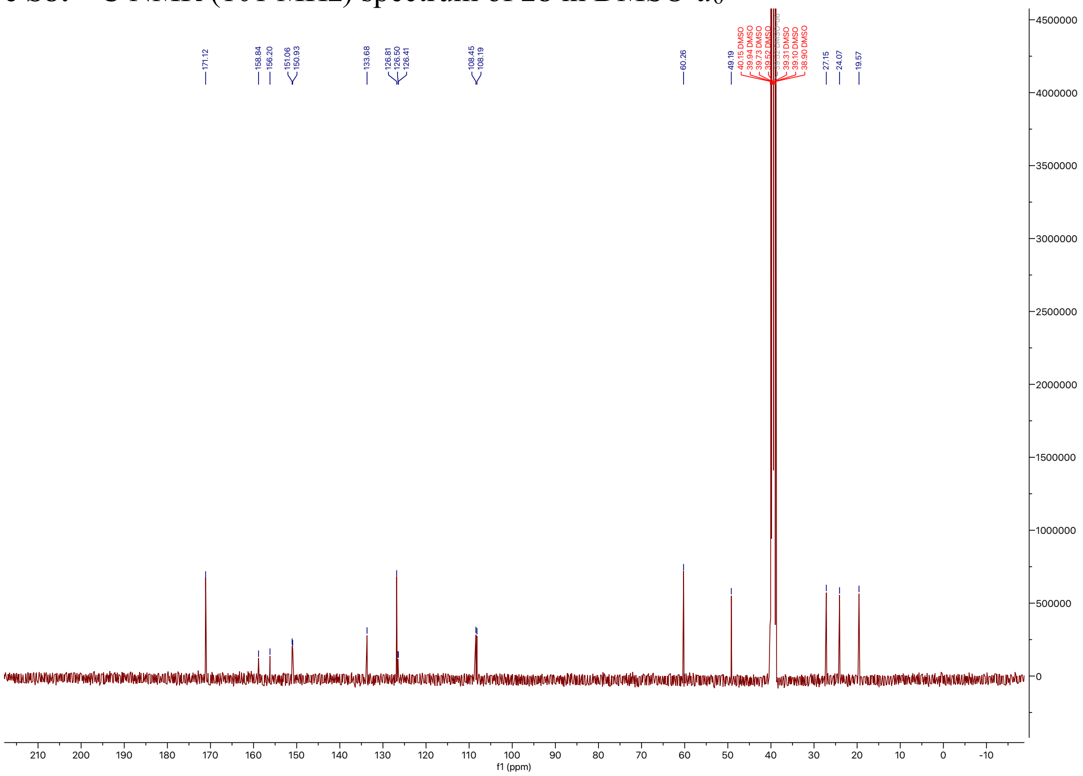

Figure S9: ^{19}F NMR (376 MHz) spectrum of **28** in DMSO-d_6 referenced to hexafluorobenzene internal standard (δ -164.9 ppm).

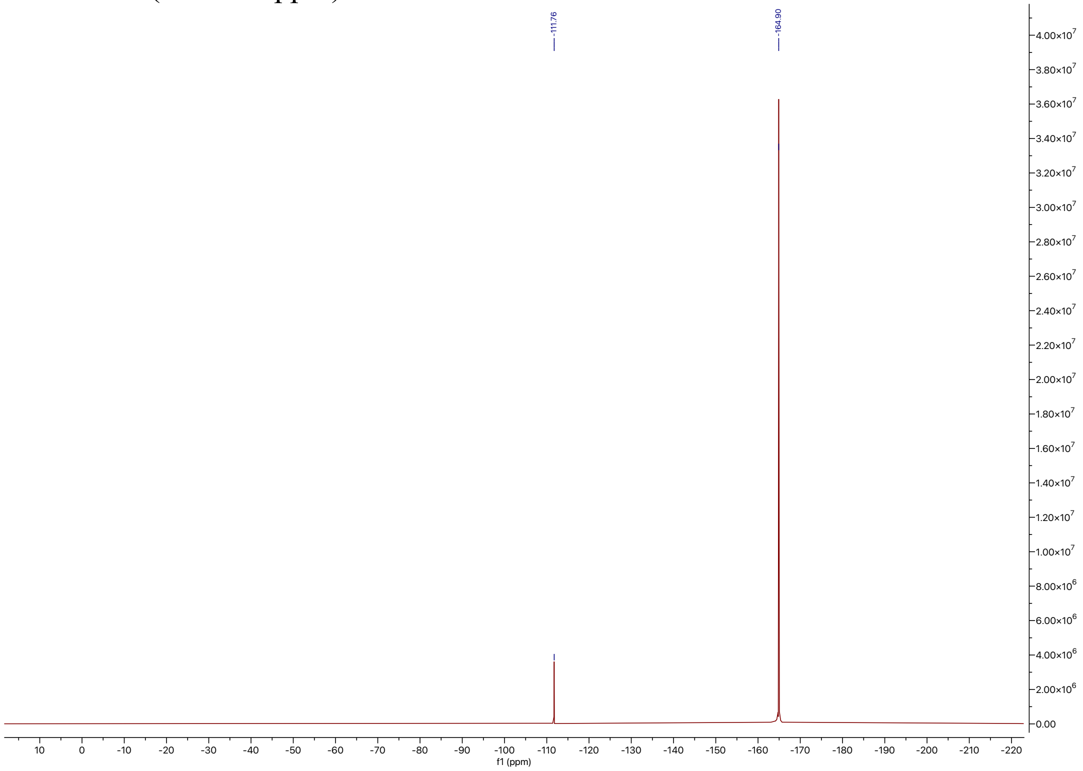

(*S*)-1-(5-Fluoro-2,4-dinitrophenyl)azetidine-2-carboxamide (29)

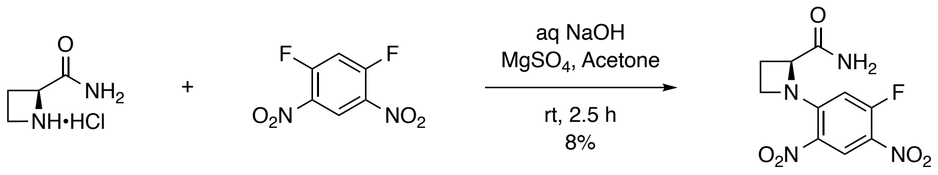

S4 *27* *29*

(*S*)-Azetidine-2-carboxamide can be purchased, but we found it more cost-effective to treat methyl (*S*)-azetidine-2-carboxylate hydrochloride[7] (i.e., **S4**) with concentrated aqueous ammonia (5 mL ammonia per gram of substrate) with stirring at room temperature. After 20 h, the mixture was concentrated, and the resulting solid was used as is for the preparation of **29**.

The azetidine-containing analogue of Marfey's reagent (i.e., **29**) was prepared following General Procedure A by using (*S*)-azetidine-2-carboxamide hydrochloride (**S4**, 506 mg, 6.1 mmol). Product **29** was recovered as a yellow solid 75 mg, 0.26 mmol, 8%). When **S4** was purchased from Ambeed, the yield for the procedure improved to 12%.

^{1}H NMR (400 MHz, DMSO-d_6) δ 8.58 (d, *J* = 7.8 Hz, 1H), 7.52 (br s, 1H), 7.20 (br s, 1H), 6.75 (d, *J* = 14.2 Hz, 1H), 5.17 – 5.10 (m, 1H), 4.15 (q, *J* = 8.8 Hz, 1H), 4.10 – 5.06 (s, 1H), 2.70 (p, *J* = 9.5 Hz, 1H), and 2.20 – 2.11 (m, 1H).

^{13}C{^{1}H} NMR (101 MHz, DMSO-d_6) δ 171.1, 157.5 (d, J = 264.7 Hz), 147.9 (d, J = 13.3 Hz), 130.1, 126.5, 124.4 (d, J = 9.3 Hz), 103.0 (d, J = 26.8 Hz), 66.3, 52.1, and 20.8.

^{19}F{^{1}H} NMR (376 MHz, DMSO-d_6) δ -113.2.

HRMS (ESI$^-$): Calcd for $C_{10}H_8FN_4O_5^-$ [M – H]$^-$ requires 283.0484; found 283.0484.

$[\boldsymbol{\alpha}]_{\boldsymbol{D}}^{\boldsymbol{23}}$ = -1076.2º (c = 0.5, acetone); $[\boldsymbol{\alpha}]_{\boldsymbol{546}}^{\boldsymbol{23}}$ = -1452.4º (c = 0.5, acetone)

FTIR (neat): 3474, 3347, 1670, 1625, 1580, 1521, 1327, 1282, 1223, 1148, 1074, 1021, 954, 910, 835, and 716 cm^{-1}.

MP: 235–240 ºC (decomposed).

Figure S7: ^{1}H NMR (400 MHz) spectrum of **29** in DMSO-d_6

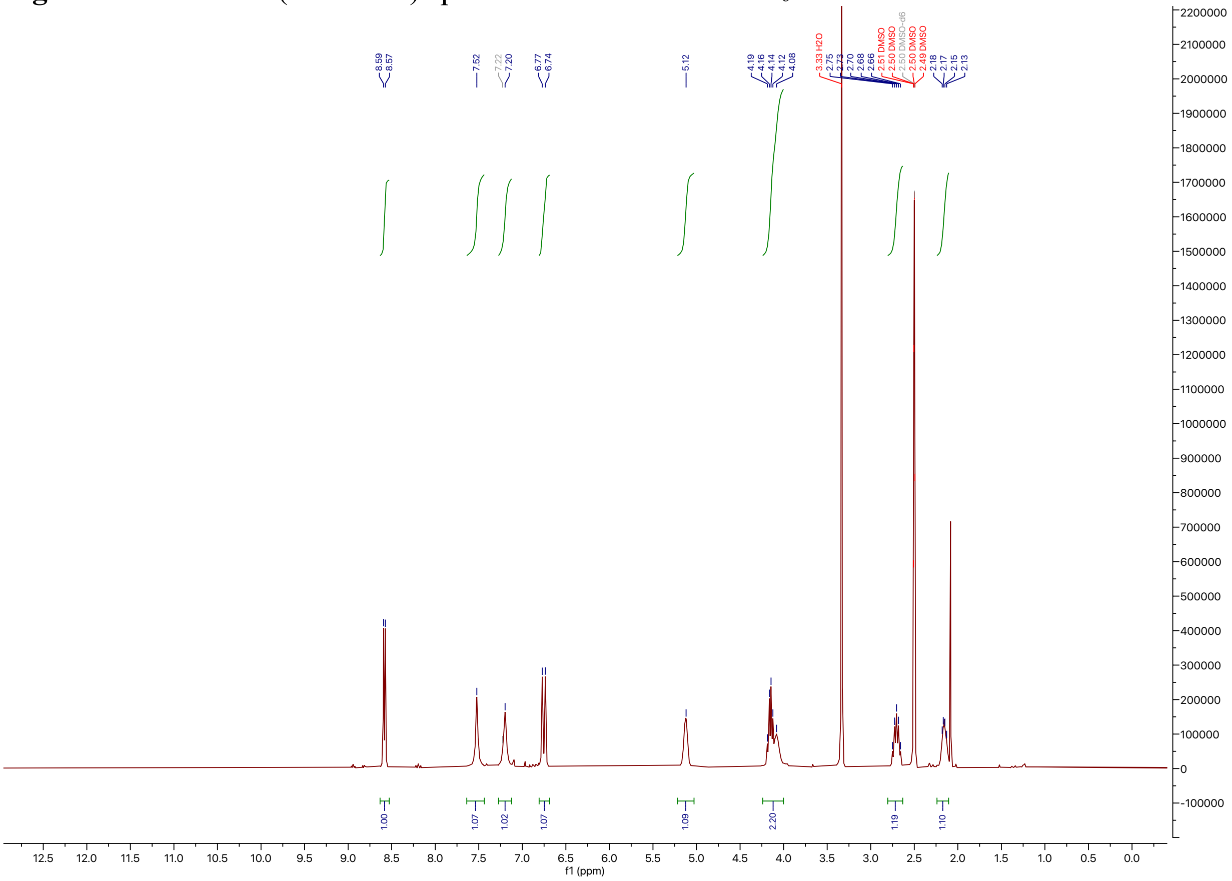

Figure S8: ^{13}C NMR (101 MHz) spectrum of **29** in DMSO-d_6

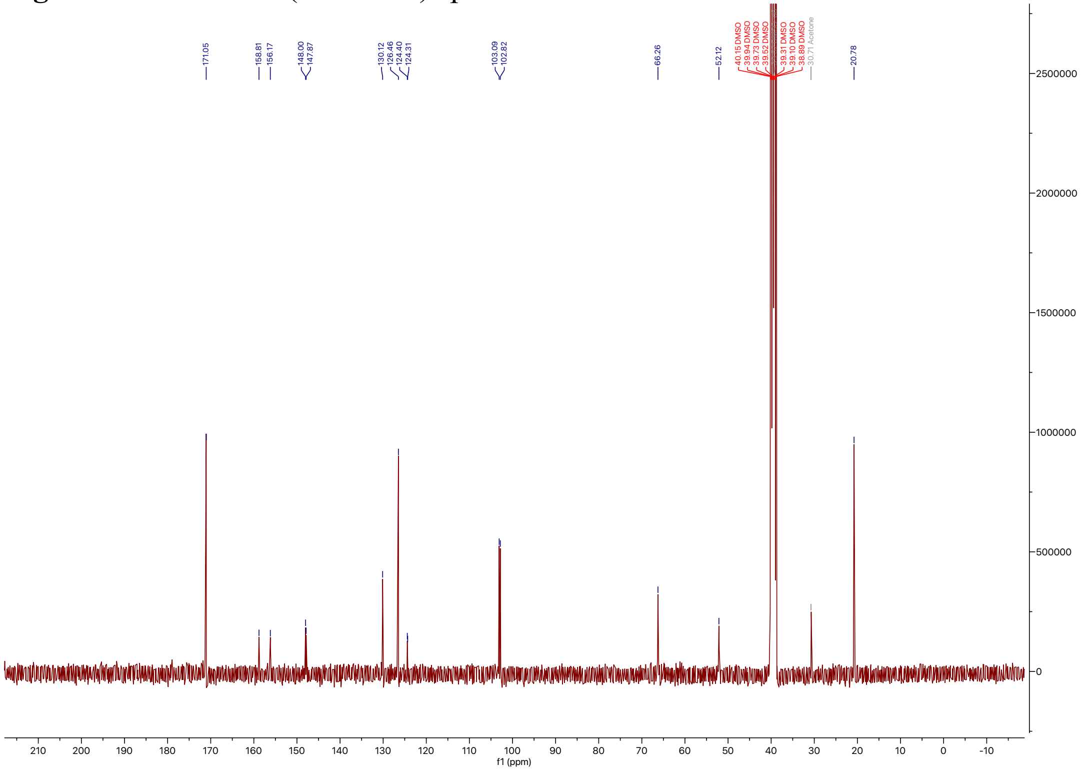

Figure S9: ^{19}F NMR (376 MHz) spectrum of **29** in DMSO-d_6 referenced to hexafluorobenzene internal standard (δ -164.9 ppm).

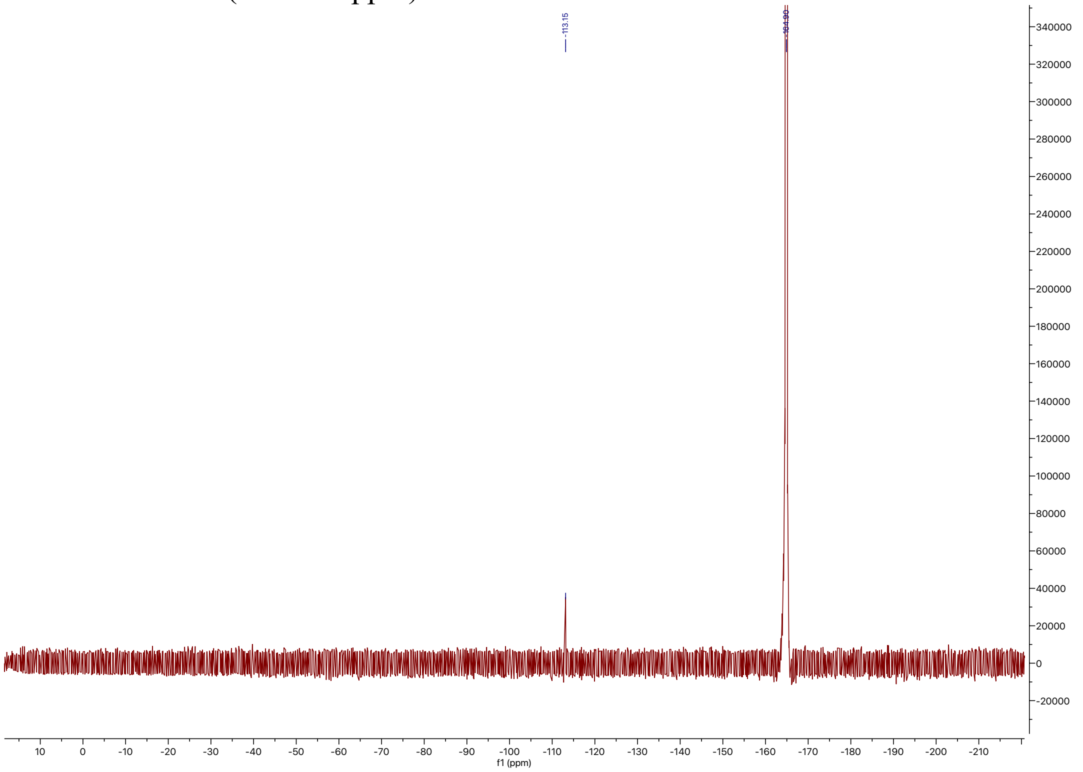

III. Derivatization and Separation of Amino Acid Stereoisomers with Various Marfey's Reagents

III-A. HPLC Methods Used for the Separation of Derivatized Amino Acids

HPLC Method A (i.e., 25-minute method)**:** Agilent Zorbax Extend-C18 column, 2.1 x 50 mm (1.8 µm), gradient of 5%–50% MeCN in H_2O + 0.1% HCO_2H over 25 min followed by 2 min gradient of 50%–5% MeCN then 3 min hold at 5% MeCN for column conditioning, flow rate 0.35 mL min^{-1}. The Agilent column part number is 727700-902.

HPLC Method B (i.e., 10-minute method)**:** Agilent Zorbax Extend-C18 column, 2.1 x 50 mm (1.8 µm), gradient of 5%–50% MeCN in H_2O + 0.1% HCO_2H over 10 min followed by 2 min gradient of 50%–5% MeCN then 3 min hold at 5% MeCN for column conditioning, flow rate 0.35 mL min^{-1}. The Agilent column part number is 727700-902.

HPLC Method C (i.e., 30-minute isocratic method)**:** Agilent Zorbax Extend-C18 column, 2.1 x 50 mm (1.8 µm), isocratic of 30% MeCN in H_2O + 0.1% HCO_2H, flow rate 0.35 mL min^{-1}. The Agilent column part number is 727700-902.

III-B. Procedures for Derivatization of Amino Acid Substrates

General Procedure B: Derivatization and Separation of a Stereoisomeric Mixture of Phenylserine with Various Marfey's Reagents

A synthetic sample of phenylserine was prepared as previously reported,[8] which gave a mixture of the four possible stereoisomers (i.e., L-*erythro* **11**, L-*threo* **12**, D-*erythro* **13**, and D-*threo* **14**), favoring the *threo* isomers ~2:1. The synthetic sample of phenylserine was used to prepare a 10 mM solution of the amino acid in 15 mM aq. $NaHCO_3$. The Marfey's derivatization reactions were performed by adding 30 µL of the 10 mM phenylserine solution, 120 µL of 15 mM aq. $NaHCO_3$, and 150 µL of 10 mM Marfey's reagent in MeCN to a plastic Eppendorf tube. The final reaction concentrations were 1 mM amino acid, 5 mM Marfey's reagent, and 7.5 mM $NaHCO_3$. The reaction was incubated in the dark at 37 ºC. After 20 hours, the reactions were quenched at room temperature by adding 150 µL of 60 mM aq. HCl and 150 µL of MeCN. The quenched reaction mixture was diluted 5-fold in 1:1 MeCN:H_2O by diluting 200 µL of the mixture into 800 µL of 1:1 MeCN:H_2O. HPLC retention times for each of the derivatized stereoisomers were assessed by LC/MS using either HPLC Method A or B.

General Procedure C: Derivatization and Separation of Stereoisomeric Mixtures of Nonstandard Amino Acids with Various Marfey's Reagents

Sample Preparation from Purified Amino Acid Sources: Pure samples of noncanonical amino acids corresponding to adducts **4**, **5**, **25**, and **26** were diluted in 15 mM $NaHCO_3$ such that the final amino acid concentration ranged from 0.1–2 mg per mL of $NaHCO_3$ solution. The Marfey's derivatization reactions were performed by adding 30 µL amino acid solution, 120 µL of 15 mM aq. $NaHCO_3$, and 150 µL of 10 mM Marfey's reagent in MeCN to a plastic Eppendorf tube. The final reaction concentrations were 1 mM amino acid, 5 mM Marfey's reagent, and 7.5 mM $NaHCO_3$. The reaction was incubated in the dark at 37 ºC. After 20 hours, the reactions were quenched at room temperature by adding 150 µL of 60 mM aq. HCl and 150 µL of MeCN. The quenched reaction mixture was diluted 5-fold in 1:1 MeCN:H_2O by diluting 200 µL of the mixture into 800 µL of 1:1 MeCN:H_2O. HPLC retention times for each of the derivatized stereoisomers were assessed by LC/MS using either HPLC Method A or B.

Sample Preparation from Biocatalytic Reaction Mixtures: Noncanonical amino acids corresponding to adducts **3** and **20–24**, were be prepared under previously described[9] biocatalytic conditions, and derivatization could be performed directly on the reaction mixtures. A 30 µL aliquot of the reaction mixture containing 0.1–100 mM noncanonical amino acid was quenched with 30 µL of acetonitrile and diluted in 570 µL of 1:1 MeCN:H_2O, achieving a 20-fold dilution of the amino acid substrate. The mixture was clarified by centrifugation at 13,300 g for 10 minutes prior to reaction with the Marfey's reagent. The Marfey's derivatization reactions were performed by adding 30 µL amino acid solution, 120 µL of 15 mM aq. $NaHCO_3$, and 150 µL of 10 mM Marfey's reagent in MeCN to a plastic Eppendorf tube. The final reaction concentrations were 1 mM amino acid, 5 mM Marfey's reagent, and 7.5 mM $NaHCO_3$. The reaction was incubated in the dark at 37 ºC. After 20 hours, the reactions were quenched at room temperature by adding 150 µL of 60 mM aq. HCl and 150 µL of MeCN. The quenched reaction mixture was diluted 5-fold in 1:1 MeCN:H_2O by diluting 200 µL of the mixture into 800 µL of 1:1 MeCN:H_2O. HPLC retention times for each of the derivatized stereoisomers were assessed by LC/MS using either HPLC Method A or B.

III-C. Raw LC/MS Data for the Separation of Stereoisomeric Mixtures of Derivatized Amino Acids

A Note on Peak Assignment in the Resolution of Marfey's Reagent Adducts with the Four Stereoisomers of Phenylserine

Because L-amino acid adducts of the Marfey's reagent are known to elute faster on reverse phase HPLC,[10,11] the first two peaks were assigned to the alpha-L diastereomers, and the second two peaks were assigned to the alpha-D diastereomers. Consistent with the NMR of the synthetic sample of phenylserine, which showed the *threo* isomers to be the major products, for each pair of diastereomers, the larger peak was assigned to the *threo* isomer, and the smaller peak was assigned to the *erythro* isomer (cf. Figure 2B of the manuscript).

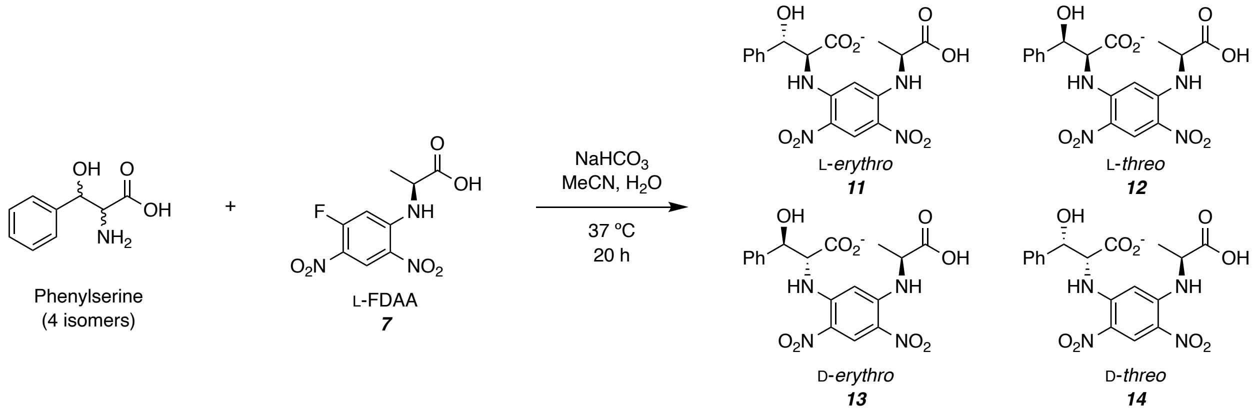

LC/MS Traces for the Attempted Resolution of the Four Stereoisomers of Phenylserine with 1-Fluoro-2,4-dinitrophenyl-5-L-alanine Amide (i.e., L-FDAA 7) with HPLC Method A (i.e., 25 min.)

Total Ion Mass Chromatogram (ESI-ToF). The Y-axis is ion counts, and the X-axis is acquisition time in minutes.

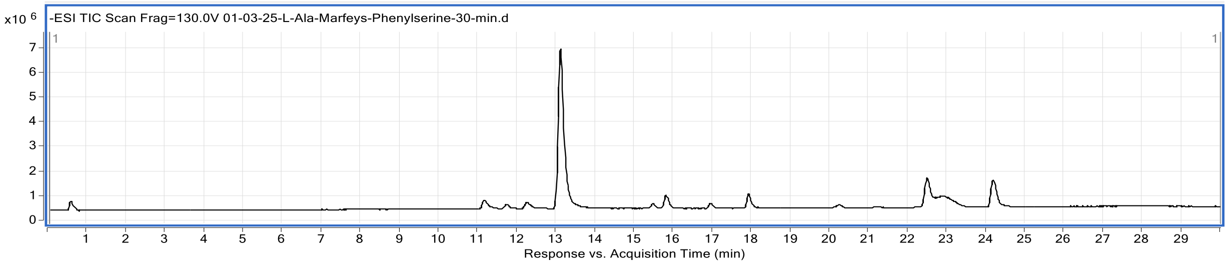

Variable Wavelength Detector Chromatogram (340 nm). The Y-axis is absorbance units, and the X-axis is acquisition time in minutes.

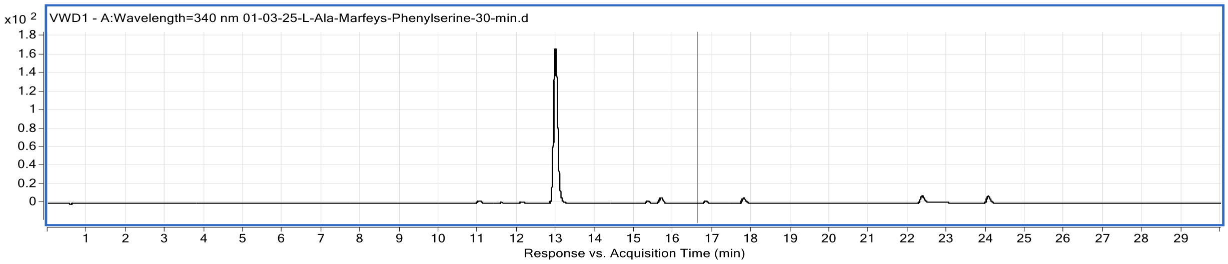

Extracted Ion Mass Chromatogram (ESI-ToF, extracted for m/z 432 ± 0.5). The Y-axis is ion counts, and the X-axis is acquisition time in minutes.

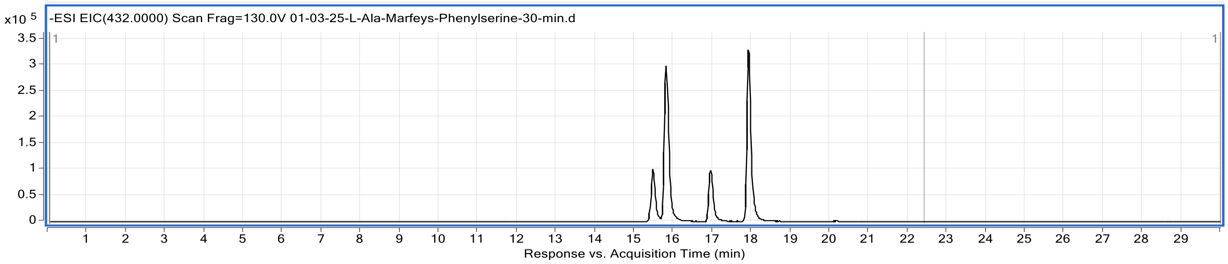

Zoomed and rescaled Extracted Ion (top) and Variable Wavelength Detector (bottom) Chromatograms. The Y axis on top is ion counts, the Y-axis on the bottom is absorbance units, and the X-axis for both is acquisition time in minutes. The four peaks are labeled from left to right as Peak 1, Peak 2, Peak 3, and Peak 4.

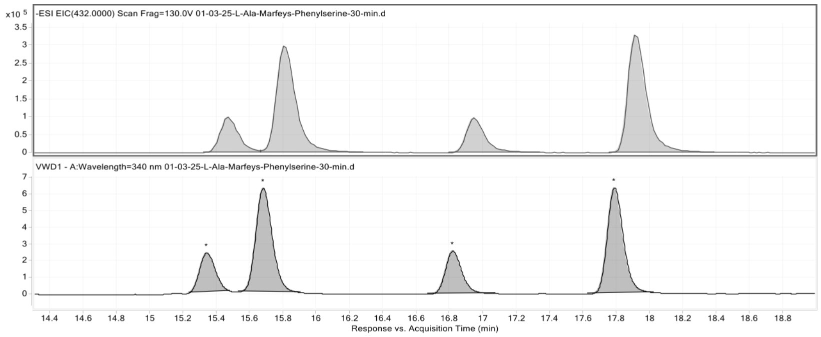

The following is a table of the peak data from the mass chromatogram. The Area % is the percent area relative to the tallest integrated peak, which has been set to 100%.

Peak	Assignment	t_R (min)	ES-ToF m/z [Neg]	Area %
1	L-*Erythro*	15.467	432.1167	29.40
2	L-*Threo*	15.799	432.1174	94.32
3	D-*Erythro*	16.945	432.1181	29.66
4	D-*Threo*	17.907	432.1181	100

The calculated m/z for the adduct of phenylserine with the Marfey's reagent is 432.1161 for $C_{18}H_{18}N_5O_8^-$ $[M - H]^{-1}$.

The following is a table of the peak data from the variable wavelength detector chromatogram (340 nm). The Area % is the percent area relative to the tallest integrated peak, which has been set to 100%.

Peak	Assignment	t_R (min)	Area %
1	L-*Erythro*	15.340	15.66
2	L-*Threo*	15.680	43.14
3	D-*Erythro*	16.817	17.70
4	D-*Threo*	17.787	42.62

The following is a table showing the difference in retention time on the extracted ion chromatogram (i.e., m/z Δt_R) and variable wavelength detector (i.e., VWD Δt_R) between the *threo* diastereomers (i.e., *Threo* separation = absolute difference between L-*threo* and D-*threo*), the *erythro* diastereomers (i.e., *Erythro* separation = absolute difference between L-*erythro* and D-*erythro*), alpha-L-diastereomers (i.e., L-separation = absolute difference between L-*threo* and L-*erythro*), and the alpha-D-diastereomers (i.e., D-separation = absolute difference between D-*threo* and D-*erythro*). The Average Δt_R values are the average difference in retention time between the extracted ion and variable wavelength detector chromatograms.

	m/z Δt_R (min)	VWD Δt_R (min)	Average Δt_R (min)
Threo Separation	2.108	2.107	2.11
Erythro Separation	1.478	1.477	1.48
L-Separation	0.332	0.34	0.34
D-Separation	0.962	0.97	0.97

LC/MS Traces for the Attempted Resolution of the Four Stereoisomers of Phenylserine with 1-Fluoro-2,4-dinitrophenyl-5-L-valine Amide (i.e., L-FDVA 15) with HPLC Method A (i.e., 25 min.)

Total Ion Mass Chromatogram (ESI-ToF). The Y-axis is ion counts, and the X-axis is acquisition time in minutes.

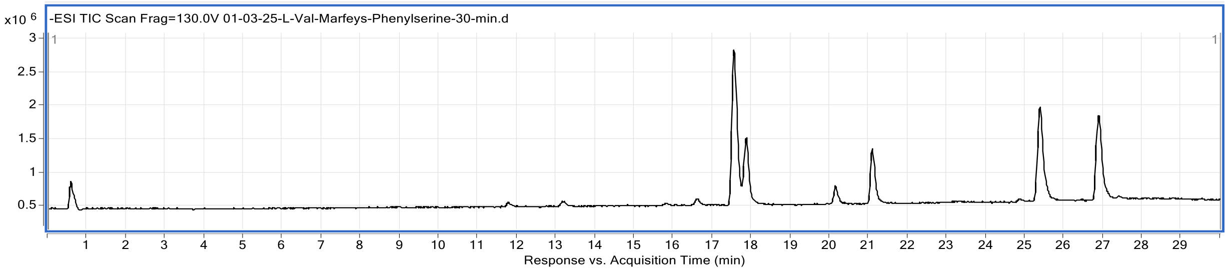

Variable Wavelength Detector Chromatogram (340 nm). The Y-axis is absorbance units, and the X-axis is acquisition time in minutes.

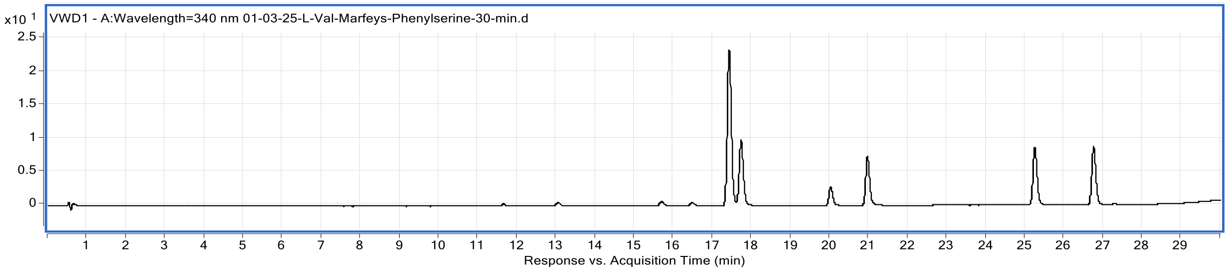

Extracted Ion Mass Chromatogram (ESI-ToF, extracted for m/z 460 ± 0.5). The Y-axis is ion counts, and the X-axis is acquisition time in minutes.

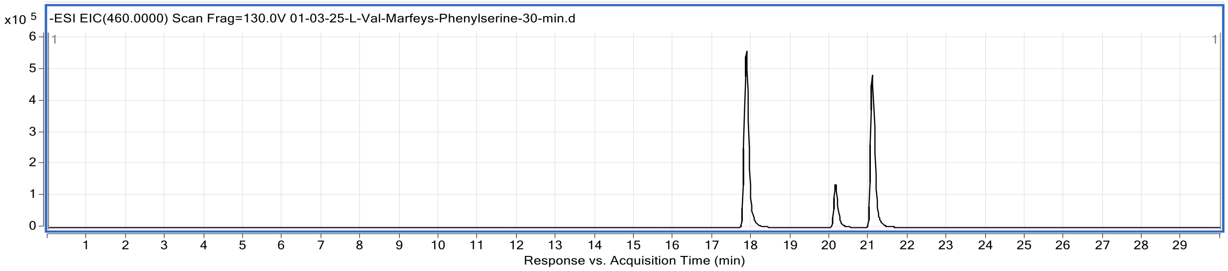

Zoomed and rescaled Extracted Ion (top) and Variable Wavelength Detector (bottom) Chromatograms. The Y axis on top is ion counts, the Y-axis on the bottom is absorbance units, and the X-axis for both is acquisition time in minutes. The peaks are labeled from left to right as Peak 1, Peak 2, and Peak 3.

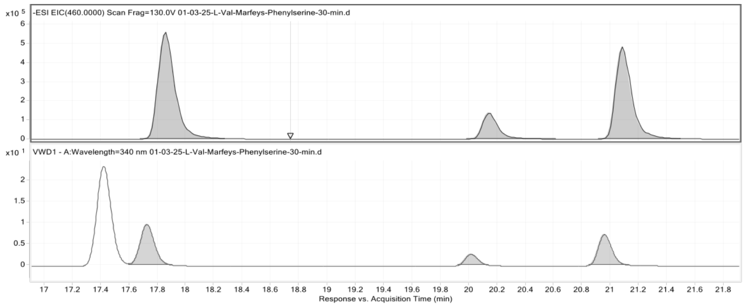

The following is a table of the peak data from the mass chromatogram. The Area % is the percent area relative to the tallest integrated peak, which has been set to 100%.

Peak	Assignment	t_R (min)	ES-ToF m/z [Neg]	Area %
1	L-*Erythro*/L-*Threo*	17.861	460.1472	100
2	D-*Erythro*	20.135	460.1467	22.69
3	D-*Threo*	21.081	460.1473	82.39

The calculated m/z for the adduct of phenylserine with the Marfey's reagent is 460.1474 for $C_{20}H_{22}N_5O_8^-$ $[M - H]^{-1}$.

The following is a table of the peak data from the variable wavelength detector chromatogram (340 nm). The Area % is the percent area relative to the tallest integrated peak, which has been set to 100%.

Peak	Assignment	t_R (min)	Area %
1	L-*Erythro*/L-*Threo*	17.723	100
2	D-*Erythro*	20.013	23.44
3	D-*Threo*	20.953	70.16

The following is a table showing the difference in retention time on the extracted ion chromatogram (i.e., m/z Δt_R) and variable wavelength detector (i.e., VWD Δt_R) between the *threo* diastereomers (i.e., *Threo* separation = absolute difference between L-*threo* and D-*threo*), the *erythro* diastereomers (i.e., *Erythro* separation = absolute difference between L-*erythro* and D-*erythro*), alpha-L-diastereomers (i.e., L-separation = absolute difference between L-*threo* and L-*erythro*), and the alpha-D-diastereomers (i.e., D-separation = absolute difference between D-*threo* and D-*erythro*). The Average Δt_R values are the average difference in retention time between the extracted ion and variable wavelength detector chromatograms.

	m/z Δt_R (min)	VWD Δt_R (min)	Average Δt_R (min)
Threo Separation	3.220	3.230	3.23
Erythro Separation	2.274	2.290	2.28
L-Separation	0	0	0
D-Separation	0.946	0.940	0.94

LC/MS Traces for the Attempted Resolution of the Four Stereoisomers of Phenylserine with 1-Fluoro-2,4-dinitrophenyl-5-L-isoleucine Amide (i.e., L-FDIA 16) with HPLC Method A (i.e., 25 min.)

Total Ion Mass Chromatogram (ESI-ToF). The Y-axis is ion counts, and the X-axis is acquisition time in minutes.

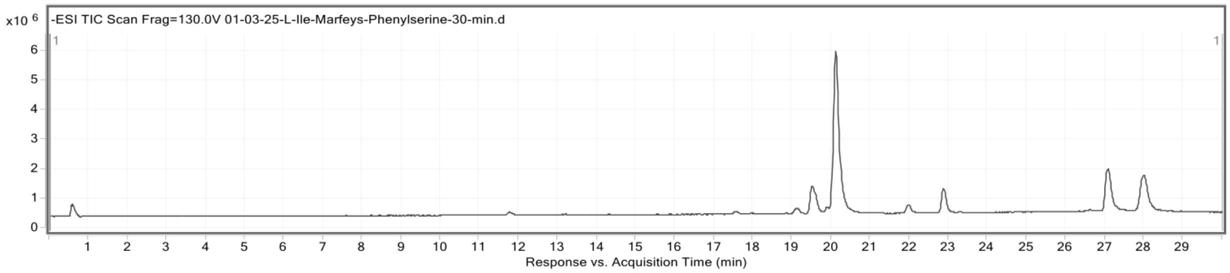

Variable Wavelength Detector Chromatogram (340 nm). The Y-axis is absorbance units, and the X-axis is acquisition time in minutes.

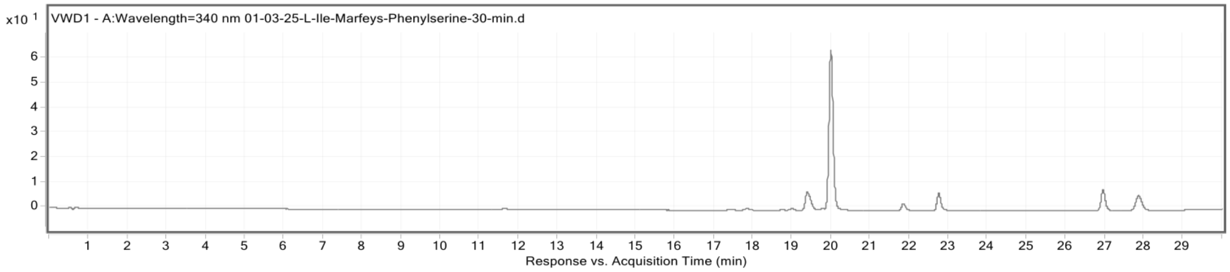

Extracted Ion Mass Chromatogram (ESI-ToF, extracted for m/z 474 ± 0.5). The Y-axis is ion counts, and the X-axis is acquisition time in minutes.

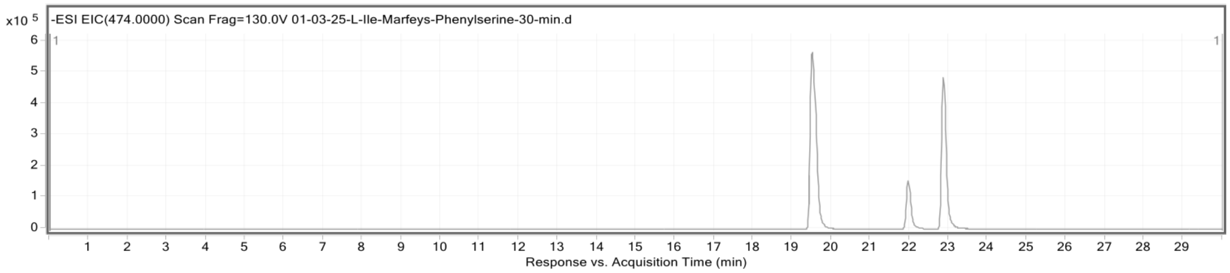

Zoomed and rescaled Extracted Ion (top) and Variable Wavelength Detector (bottom) Chromatograms. The Y axis on top is ion counts, the Y-axis on the bottom is absorbance units, and the X-axis for both is acquisition time in minutes. The peaks are labeled from left to right as Peak 1, Peak 2, and Peak 3.

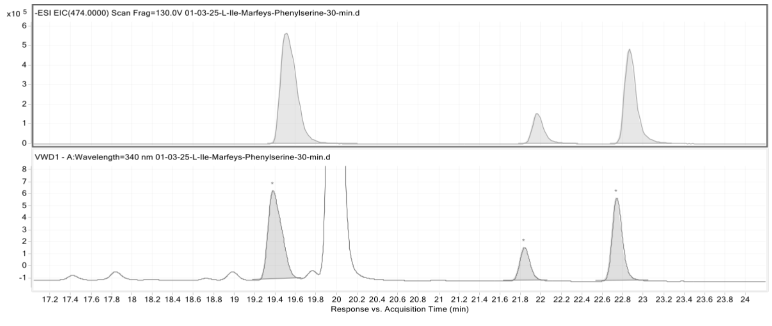

The following is a table of the peak data from the mass chromatogram. The Area % is the percent area relative to the tallest integrated peak, which has been set to 100%.

Peak	Assignment	t_R (min)	ES-ToF m/z [Neg]	Area %
1	L-*Erythro*/L-*Threo*	19.506	474.1649	100
2	D-*Erythro*	21.963	474.1637	20.01
3	D-*Threo*	22.859	474.1643	64.37

The calculated m/z for the adduct of phenylserine with the Marfey's reagent is 474.1630 for $C_{21}H_{24}N_5O_8^-$ $[M-H]^{-1}$.

The following is a table of the peak data from the variable wavelength detector chromatogram (340 nm). The Area % is the percent area relative to the tallest integrated peak, which has been set to 100%.

Peak	Assignment	t_R (min)	Area %
1	L-*Erythro*/L-*Threo*	19.373	100
2	D-*Erythro*	21.83	28.37
3	D-*Threo*	22.733	70.78

The following is a table showing the difference in retention time on the extracted ion chromatogram (i.e., m/z Δt_R) and variable wavelength detector (i.e., VWD Δt_R) between the *threo* diastereomers (i.e., *Threo* separation = absolute difference between L-*threo* and D-*threo*), the *erythro* diastereomers (i.e., *Erythro* separation = absolute difference between L-*erythro* and D-*erythro*), alpha-L-diastereomers (i.e., L-separation = absolute difference between L-*threo* and L-*erythro*), and the alpha-D-diastereomers (i.e., D-separation = absolute difference between D-*threo* and D-*erythro*). The Average Δt_R values are the average difference in retention time between the extracted ion and variable wavelength detector chromatograms.

	m/z Δt_R (min)	VWD Δt_R (min)	Average Δt_R (min)
Threo Separation	3.353	3.360	3.36
Erythro Separation	2.457	2.457	2.46
L-Separation	0	0	0
D-Separation	0.896	0.903	0.90

LC/MS Traces for the Attempted Resolution of the Four Stereoisomers of Phenylserine with 1-Fluoro-2,4-dinitrophenyl-5-L-threonine Amide (i.e., L-FDTA 17) with HPLC Method A (i.e., 25 min.)

Total Ion Mass Chromatogram (ESI-ToF). The Y-axis is ion counts, and the X-axis is acquisition time in minutes.

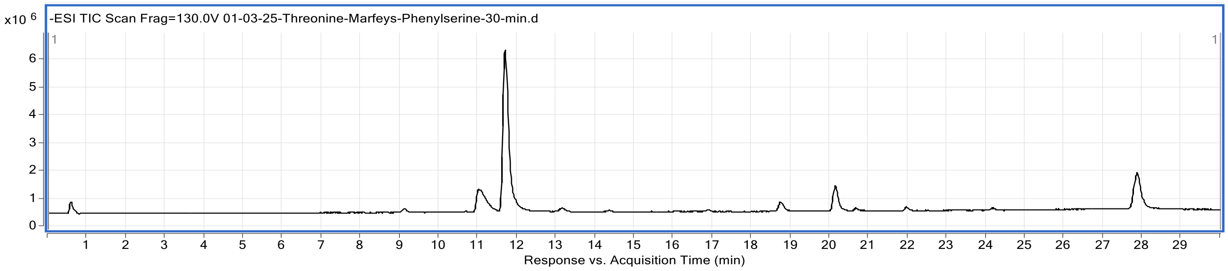

Variable Wavelength Detector Chromatogram (340 nm). The Y-axis is absorbance units, and the X-axis is acquisition time in minutes.

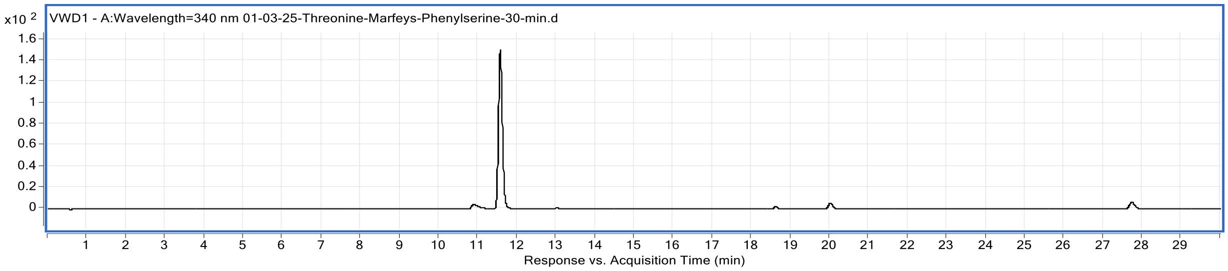

Extracted Ion Mass Chromatogram (ESI-ToF, extracted for m/z 462 ± 0.5). The Y-axis is ion counts, and the X-axis is acquisition time in minutes.

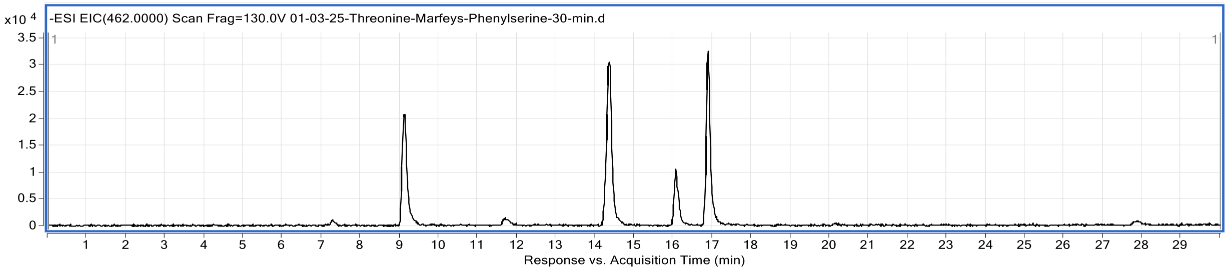

Zoomed and rescaled Extracted Ion (top) and Variable Wavelength Detector (bottom) Chromatograms. The Y axis on top is ion counts, the Y-axis on the bottom is absorbance units, and the X-axis for both is acquisition time in minutes. The peaks are labeled from left to right as Peak 1, Peak 2, and Peak 3. The VWD chromatogram showed minor resolution, but the resolution was not sufficient to quantify isomer separation

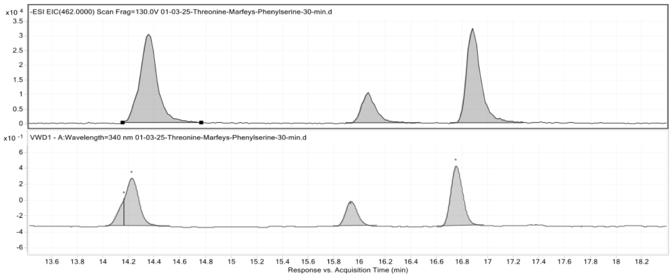

The following is a table of the peak data from the mass chromatogram. The Area % is the percent area relative to the tallest integrated peak, which has been set to 100%.

Peak	Assignment	t_R (min)	ES-ToF m/z [Neg]	Area %
1	L-*Erythro*/L-*Threo*	14.353	462.1277	100
2	D-*Erythro*	16.062	462.1264	28.67
3	D-*Threo*	16.876	462.1285	88.04

The calculated m/z for the adduct of phenylserine with the Marfey's reagent is 462.1267 for $C_{19}H_{20}N_5O_9^-$ $[M - H]^{-1}$.

The following is a table of the peak data from the variable wavelength detector chromatogram (340 nm). The Area % is the percent area relative to the tallest integrated peak, which has been set to 100%.

Peak	Assignment	t_R (min)	Area %
1	L-*Erythro*/L-*Threo*	14.163	22.83
2	D-*Erythro*	15.930	41.44
3	D-*Threo*	16.750	100

The following is a table showing the difference in retention time on the extracted ion chromatogram (i.e., m/z Δt_R) and variable wavelength detector (i.e., VWD Δt_R) between the *threo* diastereomers (i.e., *Threo* separation = absolute difference between L-*threo* and D-*threo*), the *erythro* diastereomers (i.e., *Erythro* separation = absolute difference between L-*erythro* and D-*erythro*), alpha-L-diastereomers (i.e., L-separation = absolute difference between L-*threo* and L-*erythro*), and the alpha-D-diastereomers (i.e., D-separation = absolute difference between D-*threo* and D-*erythro*). The Average Δt_R values are the average difference in retention time between the extracted ion and variable wavelength detector chromatograms.

	m/z Δt_R (min)	VWD Δt_R (min)	Average Δt_R (min)
Threo Separation	2.523	2.527	2.53
Erythro Separation	1.709	1.767	1.74
L-Separation	0	0	0
D-Separation	0.814	0.820	0.82

LC/MS Traces for the Attempted Resolution of the Four Stereoisomers of Phenylserine with 1-Fluoro-2,4-dinitrophenyl-5-L-phenylalanine Amide (i.e., L-FDFA 18) with HPLC Method A (i.e., 25 min.)

Total Ion Mass Chromatogram (ESI-ToF). The Y-axis is ion counts, and the X-axis is acquisition time in minutes.

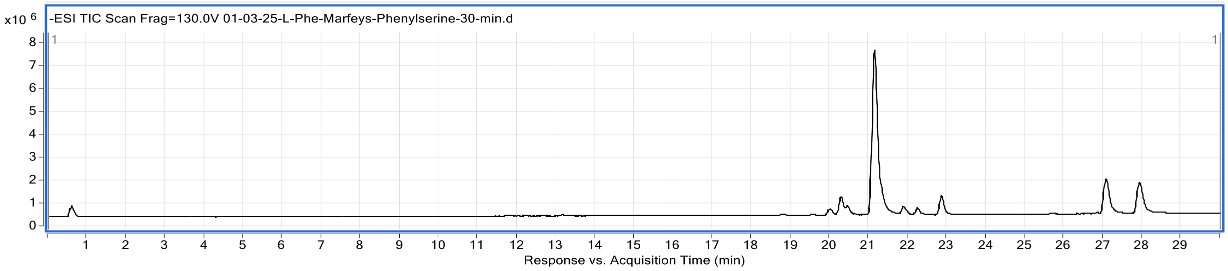

Variable Wavelength Detector Chromatogram (340 nm). The Y-axis is absorbance units, and the X-axis is acquisition time in minutes.

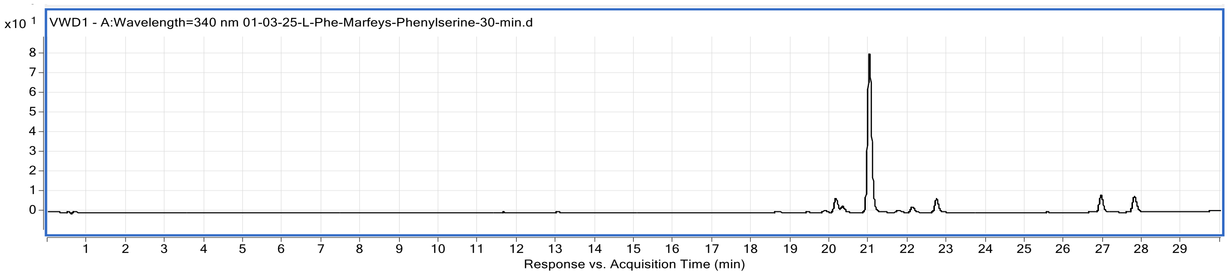

Extracted Ion Mass Chromatogram (ESI-ToF, extracted for m/z 508 ± 0.5). The Y-axis is ion counts, and the X-axis is acquisition time in minutes.

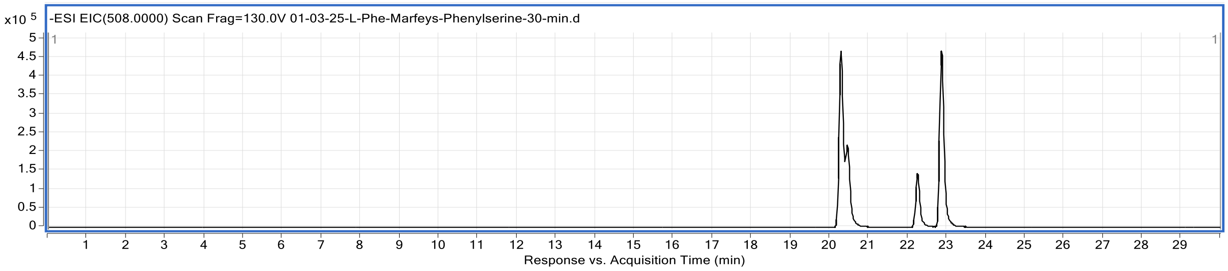

Zoomed and rescaled Extracted Ion (top) and Variable Wavelength Detector (bottom) Chromatograms. The Y axis on top is ion counts, the Y-axis on the bottom is absorbance units, and the X-axis for both is acquisition time in minutes. The peaks are labeled from left to right as Peak 1, Peak 2, Peak 3, and Peak 4.

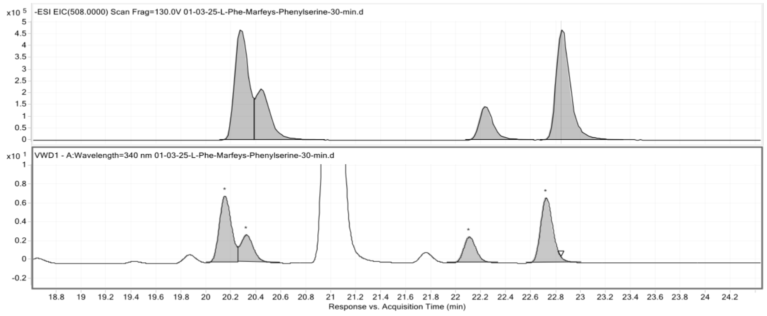

The following is a table of the peak data from the mass chromatogram. The Area % is the percent area relative to the tallest integrated peak, which has been set to 100%.

Peak	Assignment	t_R (min)	ES-ToF m/z [Neg]	Area %
1	L-*Threo*	20.271	508.1478	98.11
2	L-*Erythro*	20.437	508.1462	47.33
3	D-*Erythro*	22.229	508.1473	30.04
4	D-*Threo*	22.843	508.1473	100

The calculated m/z for the adduct of phenylserine with the Marfey's reagent is 508.1474 for $C_{24}H_{22}N_5O_8^-$ $[M - H]^{-1}$.

The following is a table of the peak data from the variable wavelength detector chromatogram (340 nm). The Area % is the percent area relative to the tallest integrated peak, which has been set to 100%.

Peak	Assignment	t_R (min)	Area %
1	L-*Threo*	20.150	100
2	L-*Erythro*	20.320	42.07
3	D-*Erythro*	22.103	39.27
4	D-*Threo*	22.720	98.69

The following is a table showing the difference in retention time on the extracted ion chromatogram (i.e., m/z Δt_R) and variable wavelength detector (i.e., VWD Δt_R) between the *threo* diastereomers (i.e., *Threo* separation = absolute difference between L-*threo* and D-*threo*), the *erythro* diastereomers (i.e., *Erythro* separation = absolute difference between L-*erythro* and D-*erythro*), alpha-L-diastereomers (i.e., L-separation = absolute difference between L-*threo* and L-*erythro*), and the alpha-D-diastereomers (i.e., D-separation = absolute difference between D-*threo* and D-*erythro*). The Average Δt_R values are the average difference in retention time between the extracted ion and variable wavelength detector chromatograms.

	m/z Δt_R (min)	VWD Δt_R (min)	Average Δt_R (min)
Threo Separation	2.572	2.570	2.57
Erythro Separation	1.792	1.783	1.79
L-Separation	0.166	0.170	0.17
D-Separation	0.614	0.617	0.62

LC/MS Traces for the Attempted Resolution of the Four Stereoisomers of Phenylserine with 1-Fluoro-2,4-dinitrophenyl-5-L-tryptophan Amide (i.e., L-FDWA 19) with HPLC Method A (i.e., 25 min.)

Total Ion Mass Chromatogram (ESI-ToF). The Y-axis is ion counts, and the X-axis is acquisition time in minutes.

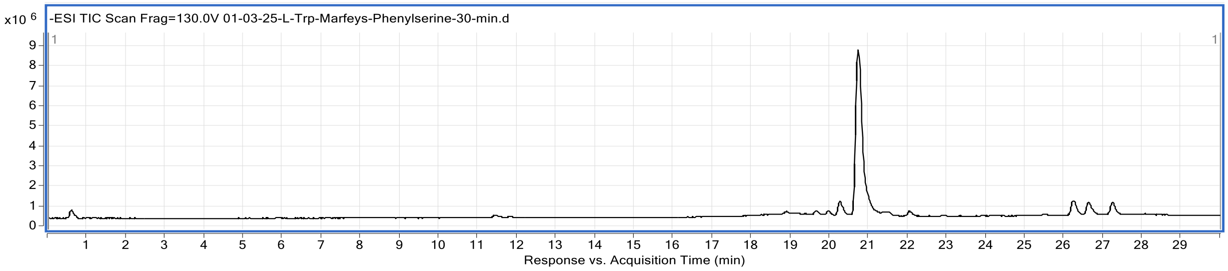

Variable Wavelength Detector Chromatogram (340 nm). The Y-axis is absorbance units, and the X-axis is acquisition time in minutes.

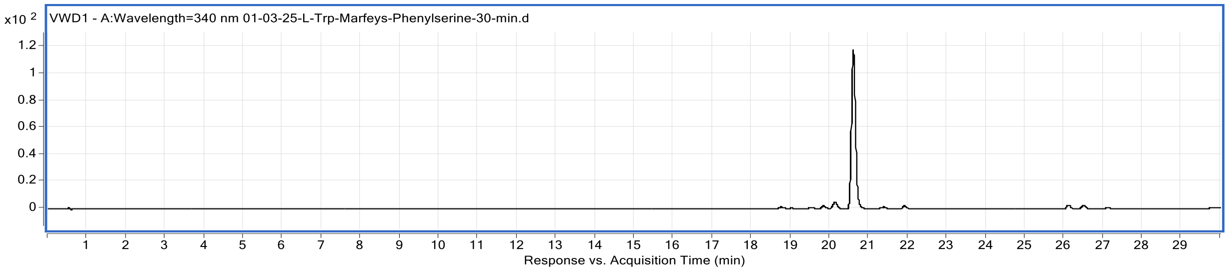

Extracted Ion Mass Chromatogram (ESI-ToF, extracted for m/z 547 ± 0.5). The Y-axis is ion counts, and the X-axis is acquisition time in minutes.

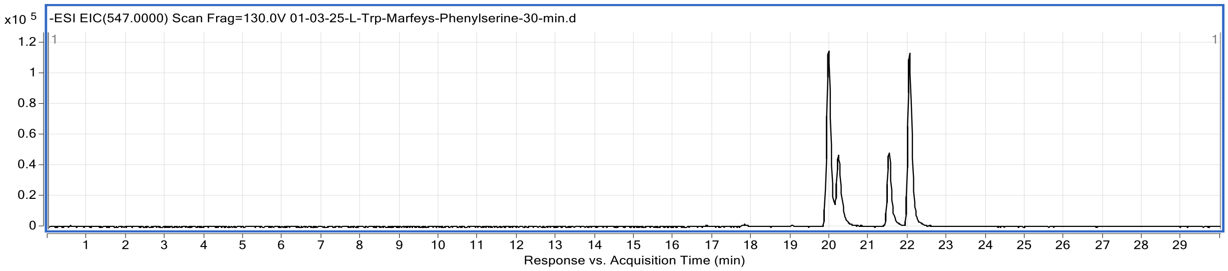

Zoomed and rescaled Extracted Ion (top) and Variable Wavelength Detector (bottom) Chromatograms. The Y axis on top is ion counts, the Y-axis on the bottom is absorbance units, and the X-axis for both is acquisition time in minutes. The peaks are labeled from left to right as Peak 1, Peak 2, Peak 3, and Peak 4.

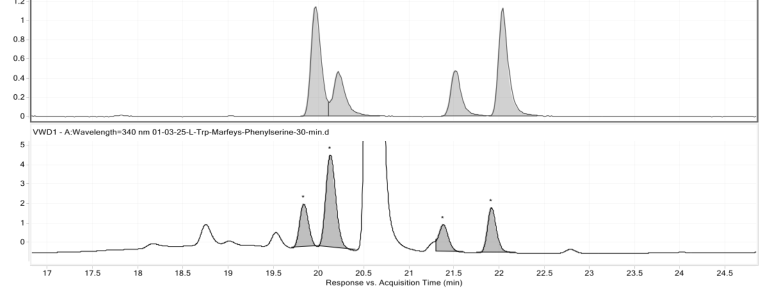

The following is a table of the peak data from the mass chromatogram. The Area % is the percent area relative to the tallest integrated peak, which has been set to 100%.

Peak	Assignment	t_R (min)	ES-ToF m/z [Neg]	Area %
1	L-*Threo*	19.963	547.1578	100
2	L-*Erythro*	20.212	547.1572	49.89
3	D-*Erythro*	21.506	547.1590	44.61
4	D-*Threo*	22.037	547.1578	95.32

The calculated m/z for the adduct of phenylserine with the Marfey's reagent is 547.1583 for $C_{26}H_{23}N_6O_8^-$ $[M - H]^{-1}$.

The following is a table of the peak data from the variable wavelength detector chromatogram (340 nm). The Area % is the percent area relative to the tallest integrated peak, which has been set to 100%.

Peak	Assignment	t_R (min)	Area %
1	L-*Threo*	19.830	41.22
2	L-*Erythro*	20.123	100*
3	D-*Erythro*	21.373	28.11
4	D-*Threo*	21.907	44.51

*Peak 2 overlapped with an unidentified byproduct.

The following is a table showing the difference in retention time on the extracted ion chromatogram (i.e., m/z Δt_R) and variable wavelength detector (i.e., VWD Δt_R) between the *threo* diastereomers (i.e., *Threo* separation = absolute difference between L-*threo* and D-*threo*), the *erythro* diastereomers (i.e., *Erythro* separation = absolute difference between L-*erythro* and D-*erythro*), alpha-L-diastereomers (i.e., L-separation = absolute difference between L-*threo* and L-*erythro*), and the alpha-D-diastereomers (i.e., D-separation = absolute difference between D-*threo* and D-*erythro*). The Average Δt_R values are the average difference in retention time between the extracted ion and variable wavelength detector chromatograms.

	m/z Δt_R (min)	VWD Δt_R (min)	Average Δt_R (min)
Threo Separation	2.074	2.077	2.08
Erythro Separation	1.294	1.250	1.27
L-Separation	0.249	0.293	0.27
D-Separation	0.531	0.534	0.53

LC/MS Traces for the Attempted Resolution of the Four Stereoisomers of Phenylserine with 1-Fluoro-2,4-dinitrophenyl-5-L-proline Amide (i.e., L-FDPA 9) with HPLC Method A (i.e., 25 min.)

Total Ion Mass Chromatogram (ESI-ToF). The Y-axis is ion counts, and the X-axis is acquisition time in minutes.

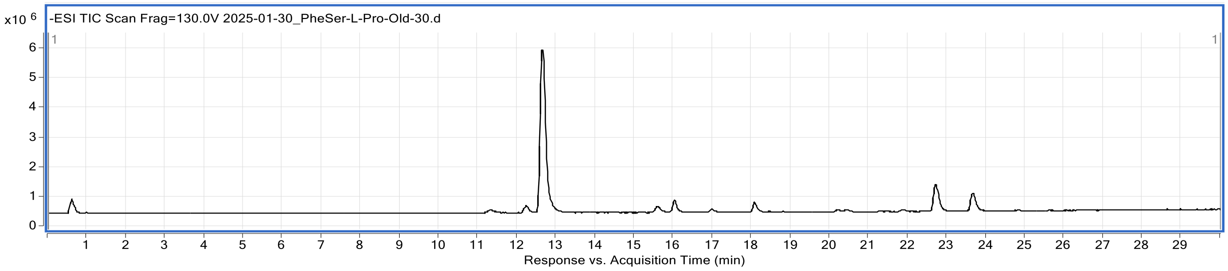

Variable Wavelength Detector Chromatogram (340 nm). The Y-axis is absorbance units, and the X-axis is acquisition time in minutes.

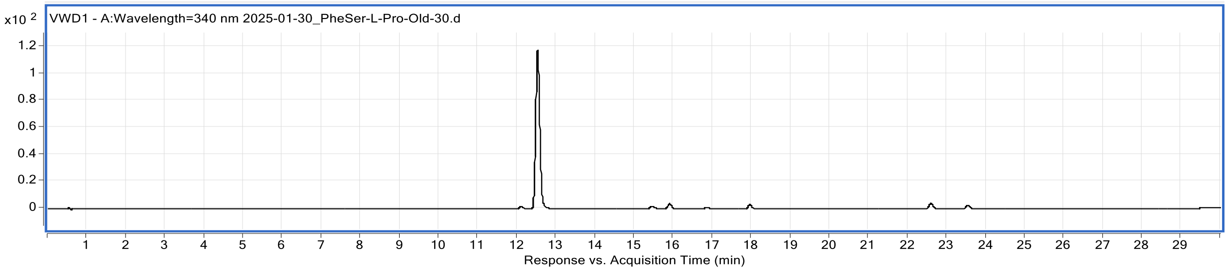

Extracted Ion Mass Chromatogram (ESI-ToF, extracted for m/z 458 ± 0.5). The Y-axis is ion counts, and the X-axis is acquisition time in minutes.

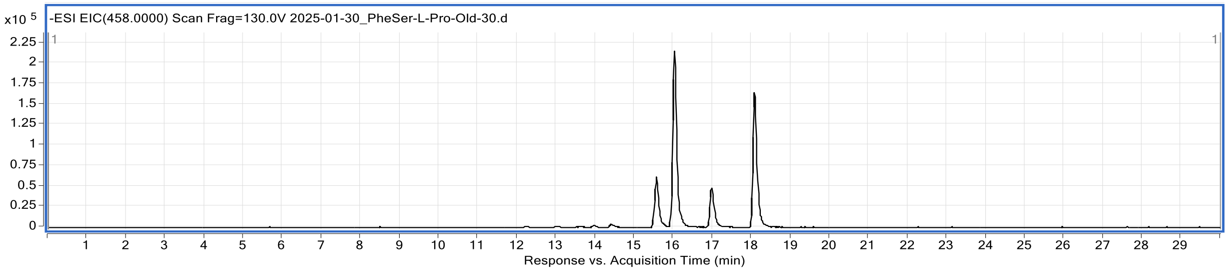

Zoomed and rescaled Extracted Ion (top) and Variable Wavelength Detector (bottom) Chromatograms. The Y axis on top is ion counts, the Y-axis on the bottom is absorbance units, and the X-axis for both is acquisition time in minutes. The four peaks are labeled from left to right as Peak 1, Peak 2, Peak 3, and Peak 4.

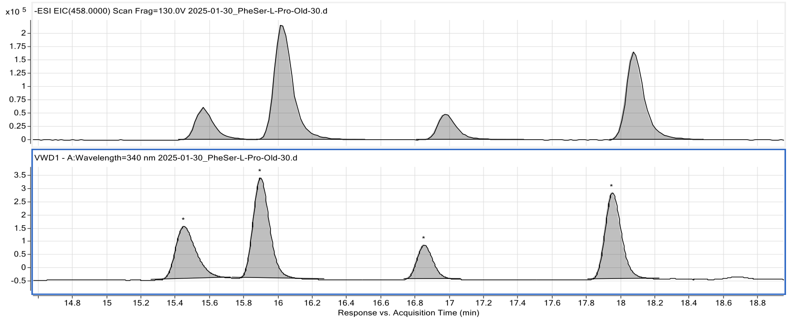

The following is a table of the peak data from the mass chromatogram. The Area % is the percent area relative to the tallest integrated peak, which has been set to 100%.

Peak	Assignment	t_R (min)	ES-ToF m/z [Neg]	Area %
1	L-*Erythro*	15.561	458.1337	27.94
2	L-*Threo*	16.010	458.1336	100
3	D-*Erythro*	16.972	458.1314	23.27
4	D-*Threo*	18.068	458.1335	74.69

The calculated m/z for the adduct of phenylserine with the Marfey's reagent is 458.1317 for $C_{20}H_{20}N_5O_8^-$ $[M - H]^{-1}$.

The following is a table of the peak data from the variable wavelength detector chromatogram (340 nm). The Area % is the percent area relative to the tallest integrated peak, which has been set to 100%.

Peak	Assignment	t_R (min)	Area %
1	L-*Erythro*	15.447	61.72
2	L-*Threo*	15.893	100
3	D-*Erythro*	16.850	33.43
4	D-*Threo*	17.947	85.39

The following is a table showing the difference in retention time on the extracted ion chromatogram (i.e., m/z Δt_R) and variable wavelength detector (i.e., VWD Δt_R) between the *threo* diastereomers (i.e., *Threo* separation = absolute difference between L-*threo* and D-*threo*), the *erythro* diastereomers (i.e., *Erythro* separation = absolute difference between L-*erythro* and D-*erythro*), alpha-L-diastereomers (i.e., L-separation = absolute difference between L-*threo* and L-*erythro*), and the alpha-D-diastereomers (i.e., D-separation = absolute difference between D-*threo* and D-*erythro*). The Average Δt_R values are the average difference in retention time between the extracted ion and variable wavelength detector chromatograms.

	m/z Δt_R (min)	VWD Δt_R (min)	Average Δt_R (min)
Threo Separation	2.058	2.054	2.06
Erythro Separation	1.411	1.403	1.41
L-Separation	0.449	0.446	0.45
D-Separation	1.096	1.097	1.10

LC/MS Traces for the Attempted Resolution of the Diastereomers of Phenylserine with 1-Fluoro-2,4-dinitrobenzene (i.e., Sanger's Reagent 10) with HPLC Method A (i.e., 25 min.)

Total Ion Mass Chromatogram (ESI-ToF). The Y-axis is ion counts, and the X-axis is acquisition time in minutes.

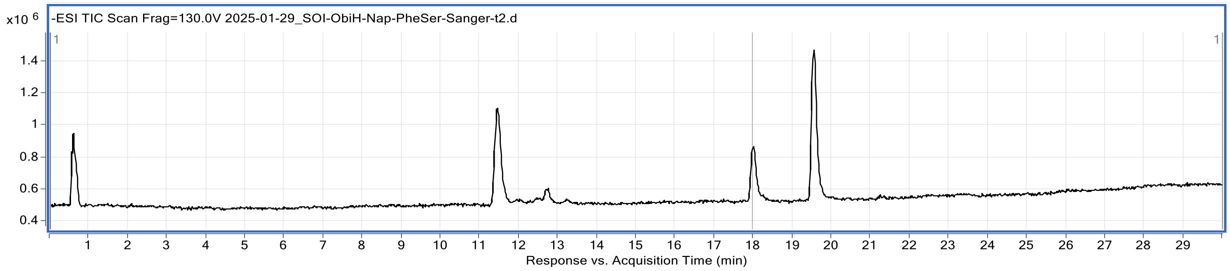

Variable Wavelength Detector Chromatogram (340 nm). The Y-axis is absorbance units, and the X-axis is acquisition time in minutes.

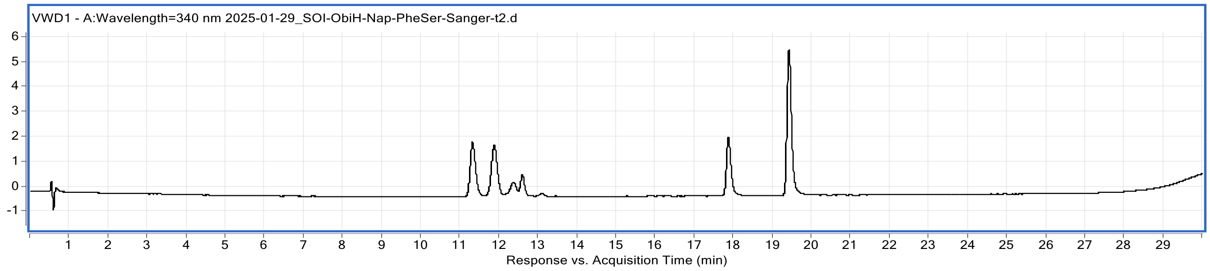

Extracted Ion Mass Chromatogram (ESI-ToF, extracted for m/z 346 ± 0.5). The Y-axis is ion counts, and the X-axis is acquisition time in minutes.

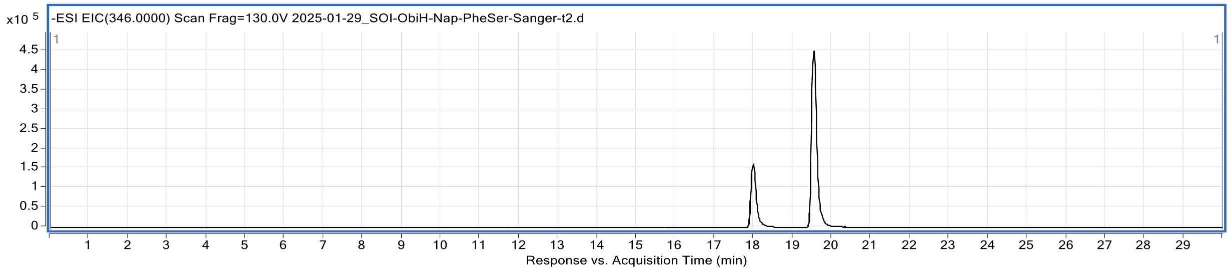

Zoomed and rescaled Extracted Ion (top) and Variable Wavelength Detector (bottom) Chromatograms. The Y axis on top is ion counts, the Y-axis on the bottom is absorbance units, and the X-axis for both is acquisition time in minutes. The peaks are labeled from left to right as Peak 1 and Peak 2.

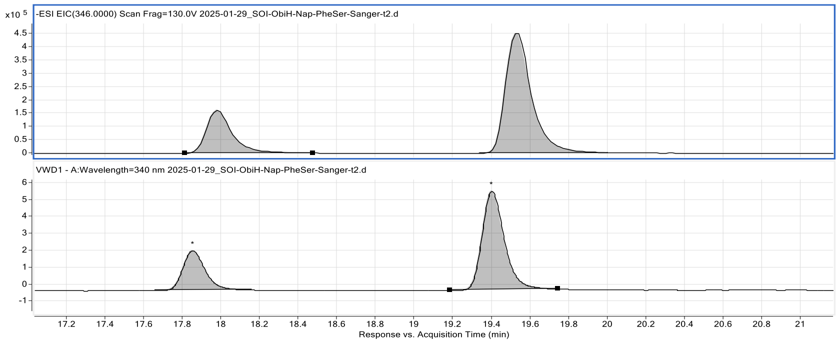

The following is a table of the peak data from the mass chromatogram. The Area % is the percent area relative to the tallest integrated peak, which has been set to 100%.

Peak	Assignment	t_R (min)	ES-ToF m/z [Neg]	Area %
1	D,L-*Erythro*	17.977	346.0706	35.68
2	D,L-*Threo*	19.520	346.0710	100

The calculated m/z for the adduct of phenylserine with Sanger's reagent is 346.0681 for $C_{15}H_{12}N_3O_7^-$ $[M-H]^{-1}$.

The following is a table of the peak data from the variable wavelength detector chromatogram (340 nm). The Area % is the percent area relative to the tallest integrated peak, which has been set to 100%.

Peak	Assignment	t_R (min)	Area %
1	D,L-*Erythro*	17.853	40.37
2	D,L-*Threo*	19.400	100

The following is a table showing the difference in retention time on the extracted ion chromatogram (i.e., m/z Δt_R) and variable wavelength detector (i.e., VWD Δt_R) between the two diastereomers (i.e., separation = absolute difference between D,L-*erythro* and D,L-*threo*). The Average Δt_R values are the average difference in retention time between the extracted ion and variable wavelength detector chromatograms.

	m/z Δt_R (min)	VWD Δt_R (min)	Average Δt_R (min)
Peak Separation	1.543	1.547	1.55

LC/MS Traces for the Attempted Resolution of the Diastereomers of Phenylserine after Fmoc Derivatization to Make 2-((((9*H*-Fluoren-9-yl)methoxy)carbonyl)amino)-3-hydroxy-3-phenylpropanoic Acid (*N*-Fmoc-3) with HPLC Method A (i.e., 25 min.)

Total Ion Mass Chromatogram (ESI-ToF). The Y-axis is ion counts, and the X-axis is acquisition time in minutes.

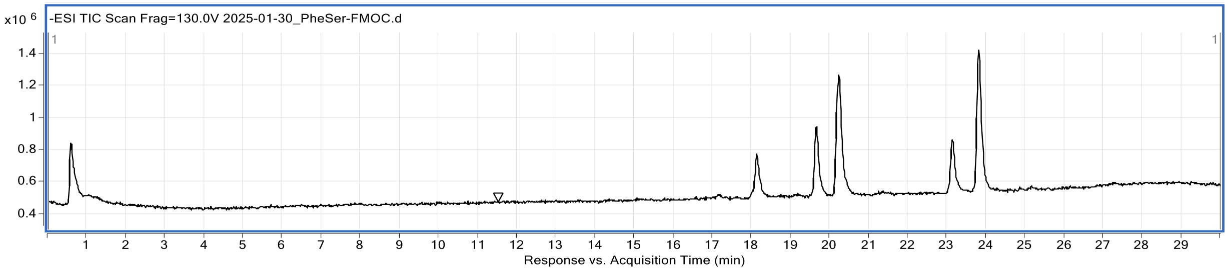

Variable Wavelength Detector Chromatogram (300 nm). The Y-axis is absorbance units, and the X-axis is acquisition time in minutes.

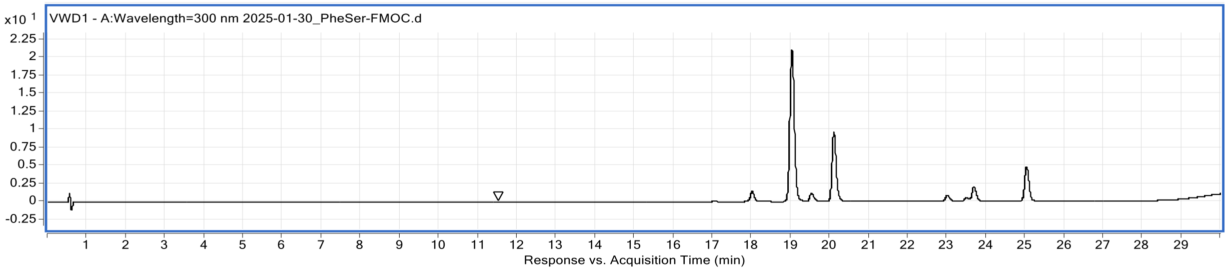

Extracted Ion Mass Chromatogram (ESI-ToF, extracted for m/z 402 ± 0.5). The Y-axis is ion counts, and the X-axis is acquisition time in minutes.

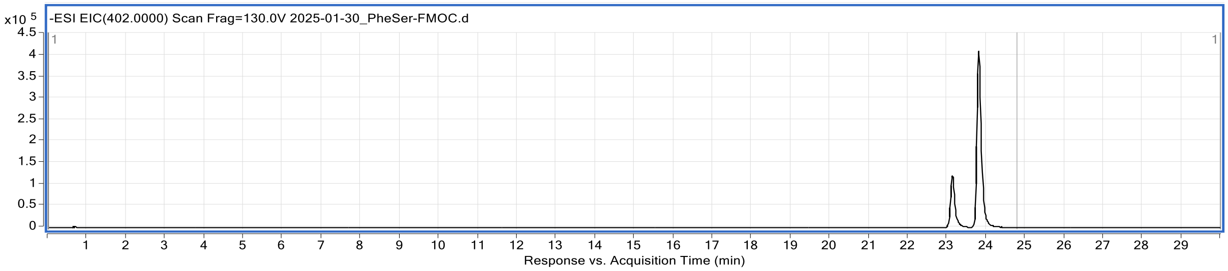

Zoomed and rescaled Extracted Ion (top) and Variable Wavelength Detector (bottom) Chromatograms. The Y axis on top is ion counts, the Y-axis on the bottom is absorbance units, and the X-axis for both is acquisition time in minutes. The peaks are labeled from left to right as Peak 1 and Peak 2.

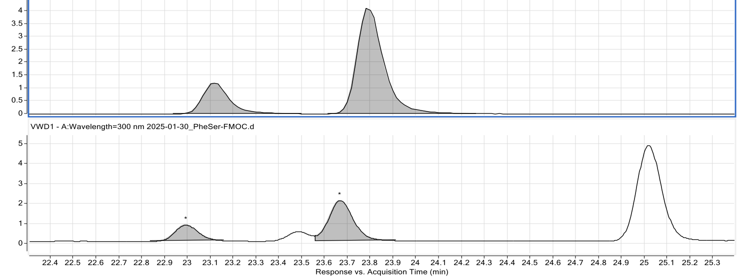

The following is a table of the peak data from the mass chromatogram. The Area % is the percent area relative to the tallest integrated peak, which has been set to 100%.

Peak	Assignment	t_R (min)	ES-ToF m/z [Neg]	Area %
1	D,L-*Erythro*	23.117	402.1379	29.18
2	D,L-*Threo*	23.781	402.1373	100

The calculated m/z is 402.1347 for $C_{24}H_{20}NO_5^-$ $[M - H]^{-1}$.

The following is a table of the peak data from the variable wavelength detector chromatogram (300 nm).

Peak	Assignment	t_R (min)	Area %
1	D,L-*Erythro*	22.993	37.2
2	D,L-*Threo*	23.667	100

The following is a table showing the difference in retention time on the extracted ion chromatogram (i.e., m/z Δt_R) and variable wavelength detector (i.e., VWD Δt_R) between the two diastereomers (i.e., separation = absolute difference between D,L-*erythro* and D,L-*threo*). The Average Δt_R values are the average difference in retention time between the extracted ion and variable wavelength detector chromatograms.

	m/z Δt_R (min)	VWD Δt_R (min)	Average Δt_R (min)
Peak Separation	0.664	0.674	0.67

LC/MS Traces for the Attempted Resolution of the Four Stereoisomers of Phenylserine with 1-Fluoro-2,4-dinitrophenyl-5-L-alanine Amide (i.e., L-FDAA 7) with HPLC Method B (i.e., 10 min.)

Total Ion Mass Chromatogram (ESI-ToF). The Y-axis is ion counts, and the X-axis is acquisition time in minutes.

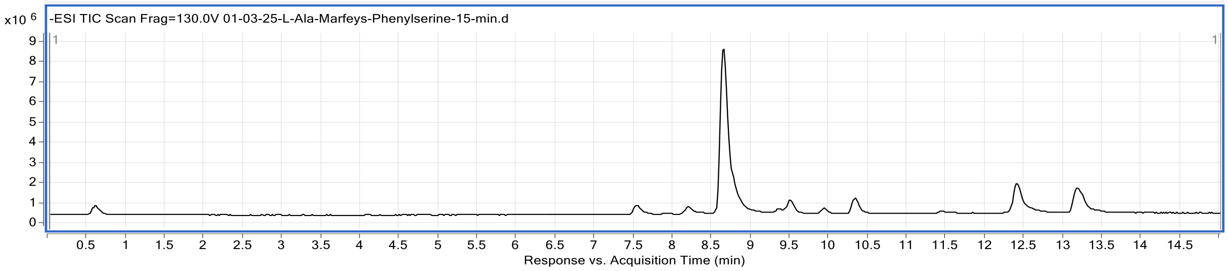

Variable Wavelength Detector Chromatogram (340 nm). The Y-axis is absorbance units, and the X-axis is acquisition time in minutes.

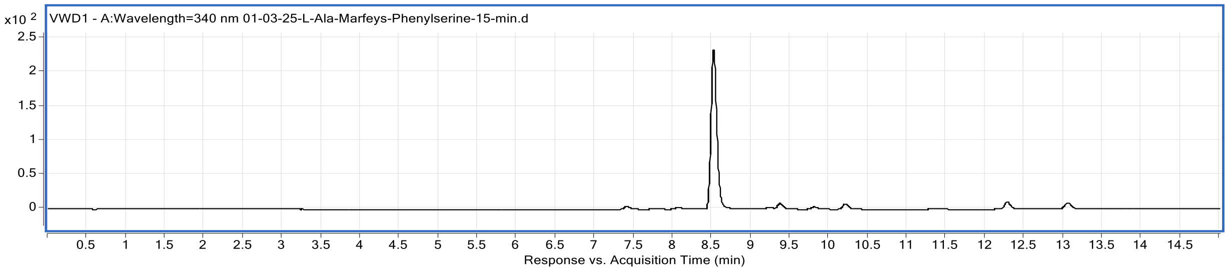

Extracted Ion Mass Chromatogram (ESI-ToF, extracted for m/z 432 ± 0.5). The Y-axis is ion counts, and the X-axis is acquisition time in minutes.

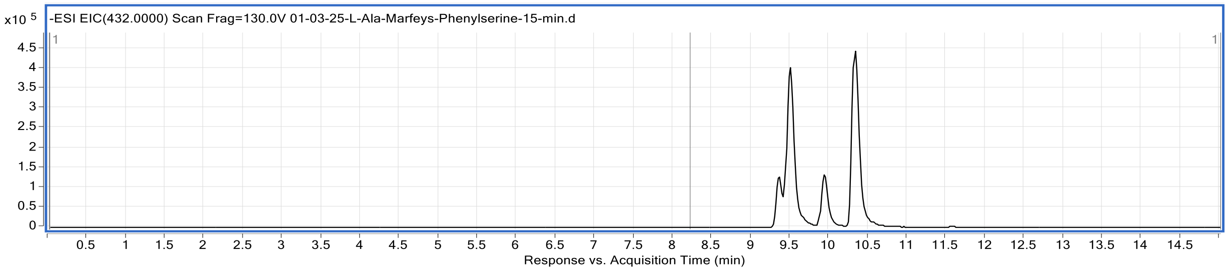

Zoomed and rescaled Extracted Ion (top) and Variable Wavelength Detector (bottom) Chromatograms. The Y axis on top is ion counts, the Y-axis on the bottom is absorbance units, and the X-axis for both is acquisition time in minutes. The four peaks are labeled from left to right as Peak 1, Peak 2, Peak 3, and Peak 4.

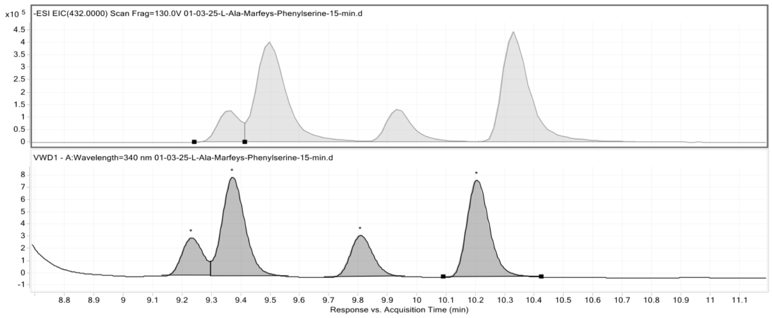

The following is a table of the peak data from the mass chromatogram. The Area % is the percent area relative to the tallest integrated peak, which has been set to 100%.

Peak	Assignment	t_R (min)	ES-ToF m/z [Neg]	Area %
1	L-*Erythro*	9.364	432.1183	23.19
2	L-*Threo*	9.497	432.1189	97.7
3	D-*Erythro*	9.928	432.1188	29.91
4	D-*Threo*	10.327	432.1184	100

The calculated m/z for the adduct of phenylserine with the Marfey's reagent is 432.1161 for $C_{18}H_{18}N_5O_8^-$ $[M - H]^{-1}$.

The following is a table of the peak data from the variable wavelength detector chromatogram (340 nm). The Area % is the percent area relative to the tallest integrated peak, which has been set to 100%.

Peak	Assignment	t_R (min)	Area %
1	L-*Erythro*	9.230	15.51
2	L-*Threo*	9.370	44.55
3	D-*Erythro*	9.807	18.33
4	D-*Threo*	10.203	42.51

The following is a table showing the difference in retention time on the extracted ion chromatogram (i.e., m/z Δt_R) and variable wavelength detector (i.e., VWD Δt_R) between the *threo* diastereomers (i.e., *Threo* separation = absolute difference between L-*threo* and D-*threo*), the *erythro* diastereomers (i.e., *Erythro* separation = absolute difference between L-*erythro* and D-*erythro*), alpha-L-diastereomers (i.e., L-separation = absolute difference between L-*threo* and L-*erythro*), and the alpha-D-diastereomers (i.e., D-separation = absolute difference between D-*threo* and D-*erythro*). The Average Δt_R values are the average difference in retention time between the extracted ion and variable wavelength detector chromatograms.

	m/z Δt_R (min)	VWD Δt_R (min)	Average Δt_R (min)
Threo Separation	0.830	0.833	0.83
Erythro Separation	0.564	0.577	0.57
L-Separation	0.133	0.140	0.14
D-Separation	0.399	0.396	0.40

LC/MS Traces for the Attempted Resolution of the Four Stereoisomers of Phenylserine with 1-Fluoro-2,4-dinitrophenyl-5-L-proline Amide (i.e., L-FDPA 9) with HPLC Method B (i.e., 10 min.)

Total Ion Mass Chromatogram (ESI-ToF). The Y-axis is ion counts, and the X-axis is acquisition time in minutes.

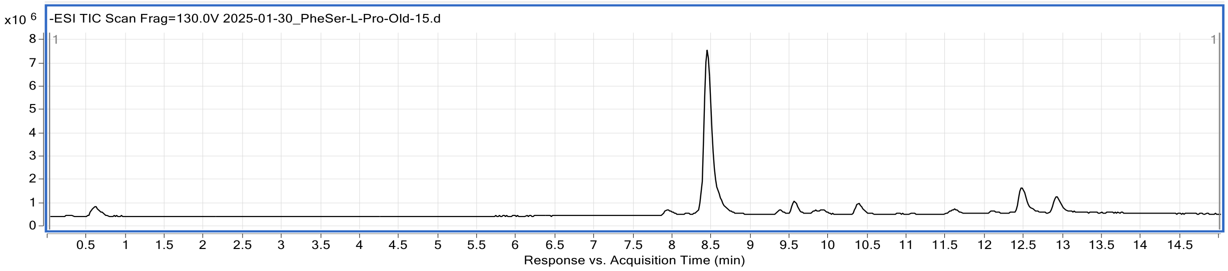

Variable Wavelength Detector Chromatogram (340 nm). The Y-axis is absorbance units, and the X-axis is acquisition time in minutes.

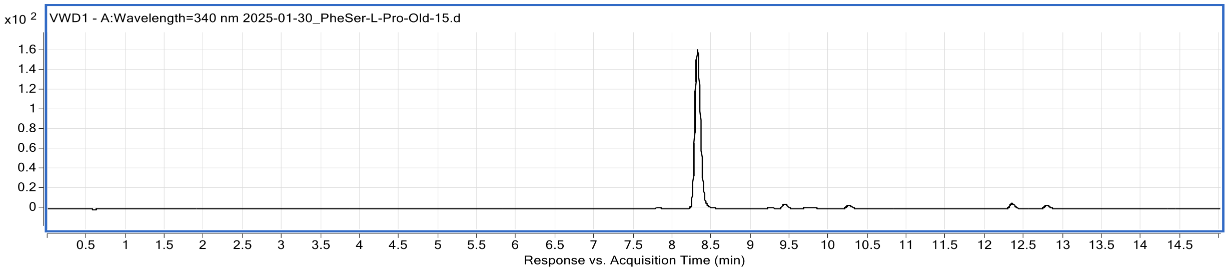

Extracted Ion Mass Chromatogram (ESI-ToF, extracted for m/z 458 ± 0.5). The Y-axis is ion counts, and the X-axis is acquisition time in minutes.

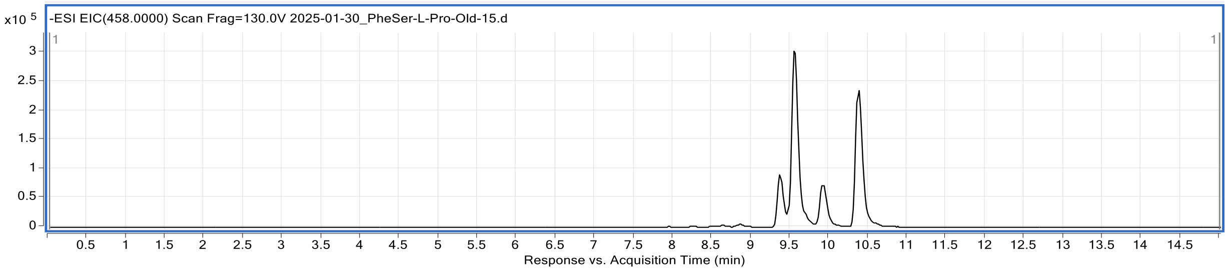

Zoomed and rescaled Extracted Ion (top) and Variable Wavelength Detector (bottom) Chromatograms. The Y axis on top is ion counts, the Y-axis on the bottom is absorbance units, and the X-axis for both is acquisition time in minutes. The four peaks are labeled from left to right as Peak 1, Peak 2, Peak 3, and Peak 4.

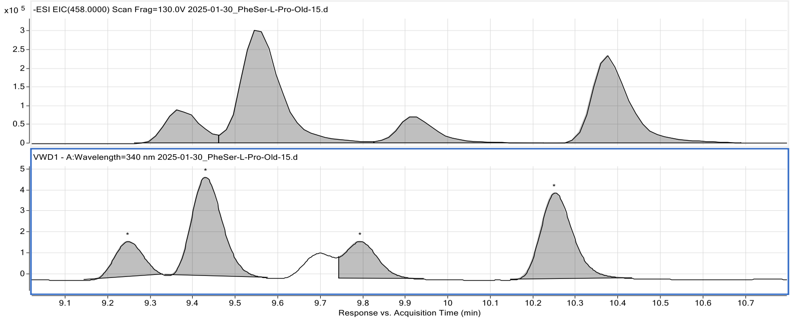

The following is a table of the peak data from the mass chromatogram. The Area % is the percent area relative to the tallest integrated peak, which has been set to 100%.

Peak	Assignment	t_R (min)	ES-ToF m/z [Neg]	Area %
1	L-*Erythro*	9.361	458.1346	26.14
2	L-*Threo*	9.544	458.1345	100
3	D-*Erythro*	9.909	458.1342	23.59
4	D-*Threo*	10.374	458.1342	73.58

The calculated m/z for the adduct of phenylserine with the Marfey's reagent is 458.1317 for $C_{20}H_{20}N_5O_8^-$ $[M – H]^{-1}$.

The following is a table of the peak data from the variable wavelength detector chromatogram (340 nm). The Area % is the percent area relative to the tallest integrated peak, which has been set to 100%.

Peak	Assignment	t_R (min)	Area %
1	L-*Erythro*	9.247	33.21
2	L-*Threo*	9.430	100
3	D-*Erythro*	9.793	39.99
4	D-*Threo*	10.25	91.58

The following is a table showing the difference in retention time on the extracted ion chromatogram (i.e., m/z Δt_R) and variable wavelength detector (i.e., VWD Δt_R) between the *threo* diastereomers (i.e., *Threo* separation = absolute difference between L-*threo* and D-*threo*), the *erythro* diastereomers (i.e., *Erythro* separation = absolute difference between L-*erythro* and D-*erythro*), alpha-L-diastereomers (i.e., L-separation = absolute difference between L-*threo* and L-*erythro*), and the alpha-D-diastereomers (i.e., D-separation = absolute difference between D-*threo* and D-*erythro*). The Average Δt_R values are the average difference in retention time between the extracted ion and variable wavelength detector chromatograms.

	m/z Δt_R (min)	VWD Δt_R (min)	Average Δt_R (min)
Threo Separation	0.830	0.820	0.83
Erythro Separation	0.548	0.546	0.55
L-Separation	0.183	0.183	0.18
D-Separation	0.465	0.457	0.46

LC/MS Traces for the Attempted Resolution of the Diastereomers of Phenylserine with 1-Fluoro-2,4-dinitrobenzene (i.e., Sanger's Reagent 10) with HPLC Method B (i.e., 10 min.)

Total Ion Mass Chromatogram (ESI-ToF). The Y-axis is ion counts, and the X-axis is acquisition time in minutes.

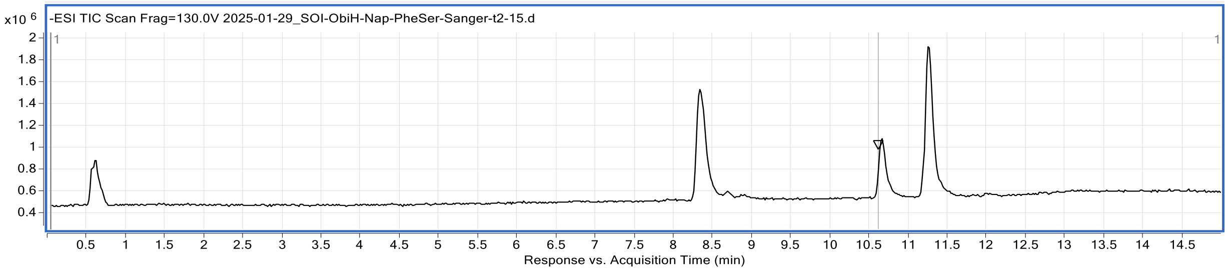

Variable Wavelength Detector Chromatogram (340 nm). The Y-axis is absorbance units, and the X-axis is acquisition time in minutes.

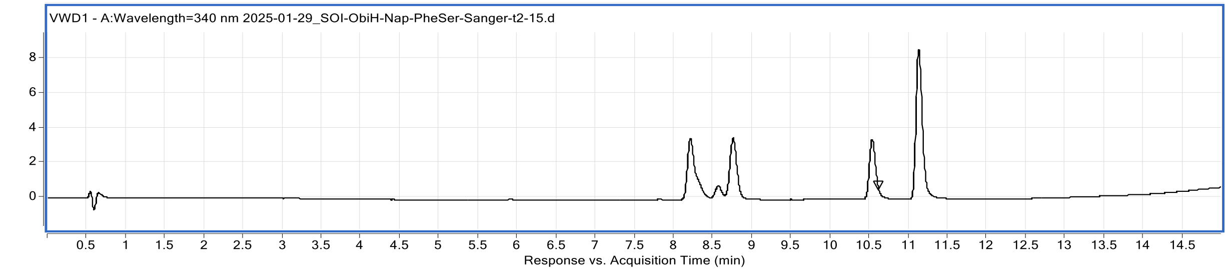

Extracted Ion Mass Chromatogram (ESI-ToF, extracted for m/z 346 ± 0.5). The Y-axis is ion counts, and the X-axis is acquisition time in minutes.

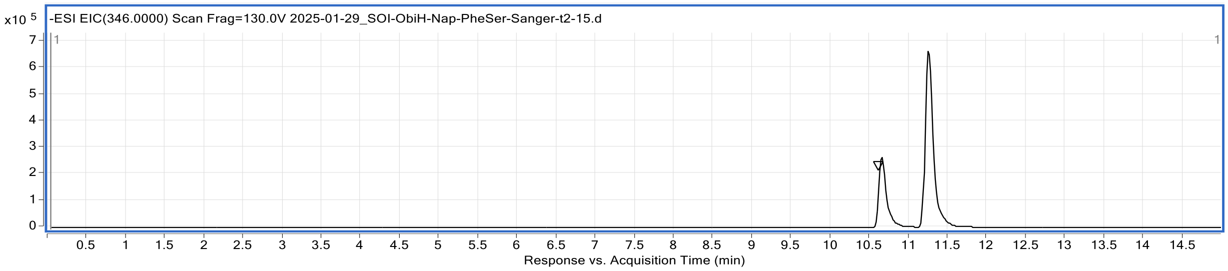

Zoomed and rescaled Extracted Ion (top) and Variable Wavelength Detector (bottom) Chromatograms. The Y axis on top is ion counts, the Y-axis on the bottom is absorbance units, and the X-axis for both is acquisition time in minutes. The peaks are labeled from left to right as Peak 1 and Peak 2.

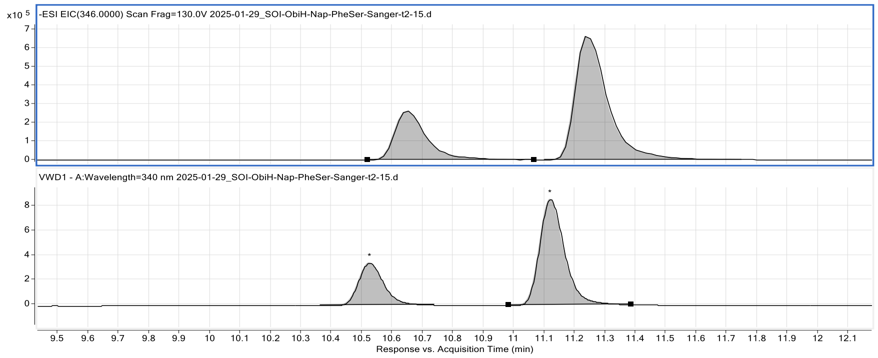

The following is a table of the peak data from the mass chromatogram. The Area % is the percent area relative to the tallest integrated peak, which has been set to 100%.

Peak	Assignment	t_R (min)	ES-ToF m/z [Neg]	Area %
1	D,L-*Erythro*	10.653	346.0692	38.39
2	D,L-*Threo*	11.234	346.0693	100

The calculated m/z for the adduct of phenylserine with Sanger's reagent is 346.0681 for $C_{15}H_{12}N_3O_7^-$ $[M-H]^{-1}$.

The following is a table of the peak data from the variable wavelength detector chromatogram (340 nm). The Area % is the percent area relative to the tallest integrated peak, which has been set to 100%.

Peak	Assignment	t_R (min)	Area %
1	D,L-*Erythro*	10.527	40.2
2	D,L-*Threo*	11.120	100

The following is a table showing the difference in retention time on the extracted ion chromatogram (i.e., m/z Δt_R) and variable wavelength detector (i.e., VWD Δt_R) between the two diastereomers (i.e., separation = absolute difference between D,L-*erythro* and D,L-*threo*). The Average Δt_R values are the average difference in retention time between the extracted ion and variable wavelength detector chromatograms.

	m/z Δt_R (min)	VWD Δt_R (min)	Average Δt_R (min)
Peak Separation	0.581	0.593	0.59

LC/MS Traces for the Attempted Resolution of the Beta Stereoisomers of the β-hydroxy-α-Amino Acid Corresponding to Adduct 3 with 1-Fluoro-2,4-dinitrophenyl-5-L-alanine Amide (i.e., L-FDAA 7) with HPLC Method B (i.e., 10 min.)

Total Ion Mass Chromatogram (ESI-ToF). The Y-axis is ion counts, and the X-axis is acquisition time in minutes.

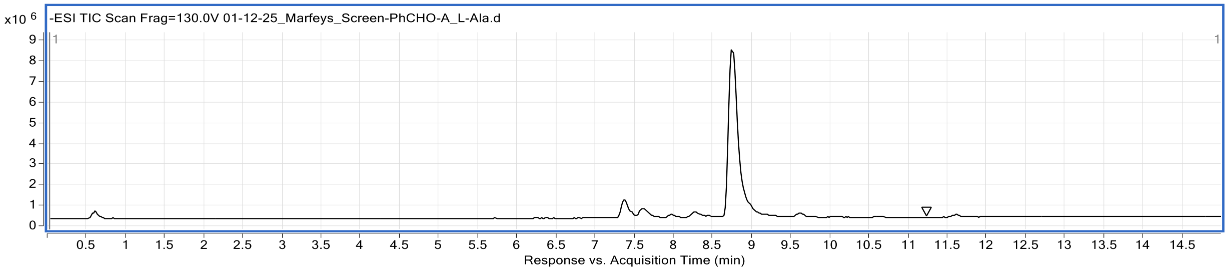

Variable Wavelength Detector Chromatogram (340 nm). The Y-axis is absorbance units, and the X-axis is acquisition time in minutes.

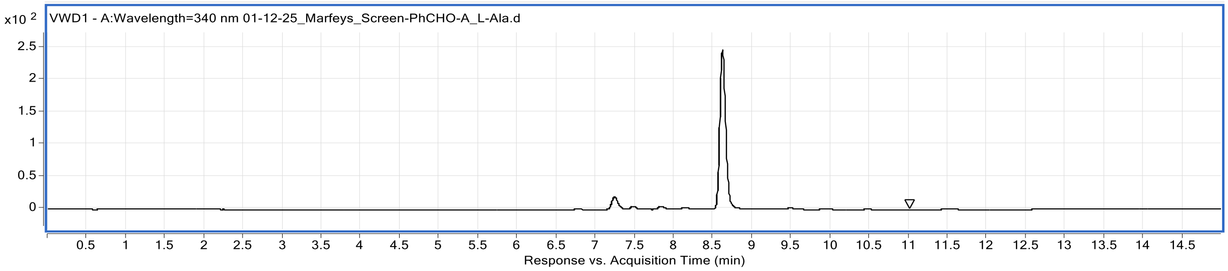

Extracted Ion Mass Chromatogram (ESI-ToF, extracted for m/z 432 ± 0.5). The Y-axis is ion counts, and the X-axis is acquisition time in minutes.

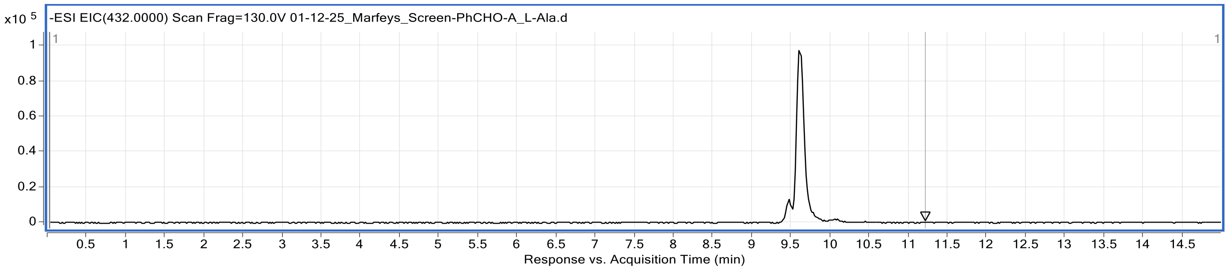

Zoomed and rescaled Extracted Ion (top) and Variable Wavelength Detector (bottom) Chromatograms. The Y axis on top is ion counts, the Y-axis on the bottom is absorbance units, and the X-axis for both is acquisition time in minutes. The peaks are labeled from left to right as Peak 1 and Peak 2.

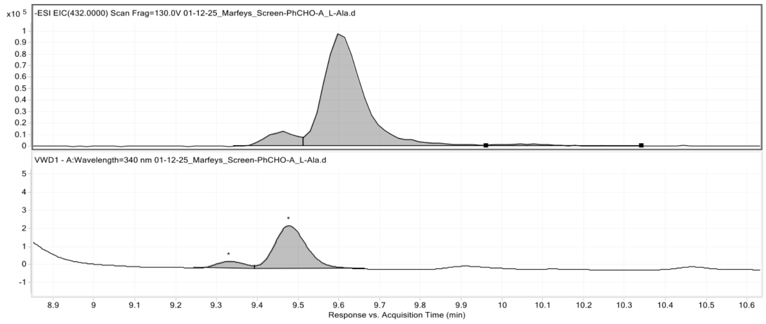

The following is a table of the peak data from the mass chromatogram. The Area % is the percent area relative to the tallest integrated peak, which has been set to 100%.

Peak	Assignment	t_R (min)	ES-ToF m/z [Neg]	Area %
1	L-*Erythro*	9.463	432.1169	10.11
2	L-*Threo*	9.595	432.1149	100

The calculated m/z for the adduct is 432.1161 for $C_{18}H_{18}N_5O_8^-$ $[M – H]^{-1}$.

The following is a table of the peak data from the variable wavelength detector chromatogram (340 nm). The Area % is the percent area relative to the tallest integrated peak, which has been set to 100%.

Peak	Assignment	t_R (min)	Area %
1	L-*Erythro*	9.330	15.31
2	L-*Threo*	9.477	100

The following is a table showing the difference in retention time on the extracted ion chromatogram (i.e., m/z Δt_R) and variable wavelength detector (i.e., VWD Δt_R) between the two diastereomers (i.e., separation = absolute difference between L-*erythro* and L-*threo*). The Average Δt_R values are the average difference in retention time between the extracted ion and variable wavelength detector chromatograms.

	m/z Δt_R (min)	VWD Δt_R (min)	Average Δt_R (min)
Peak Separation	0.132	0.147	0.14

LC/MS Traces for the Attempted Resolution of the Beta Stereoisomers of the β-hydroxy-α-Amino Acid Corresponding to Adduct 3 with 1-Fluoro-2,4-dinitrophenyl-5-D-alanine Amide (i.e., D-FDAA D-7) with HPLC Method B (i.e., 10 min.)

Total Ion Mass Chromatogram (ESI-ToF). The Y-axis is ion counts, and the X-axis is acquisition time in minutes.

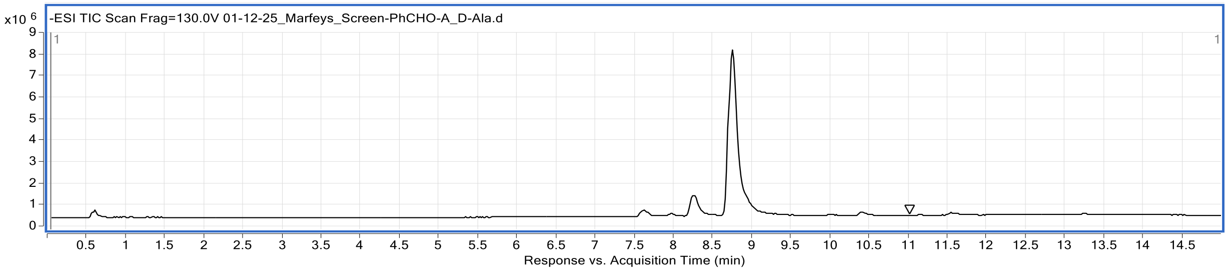

Variable Wavelength Detector Chromatogram (340 nm). The Y-axis is absorbance units, and the X-axis is acquisition time in minutes.

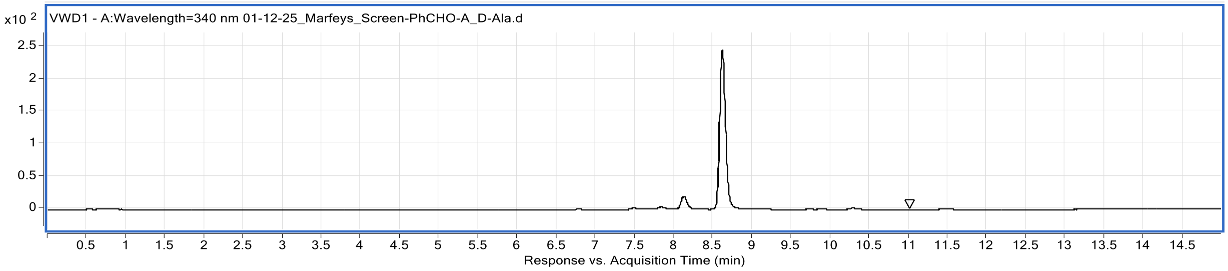

Extracted Ion Mass Chromatogram (ESI-ToF, extracted for m/z 432 ± 0.5). The Y-axis is ion counts, and the X-axis is acquisition time in minutes.

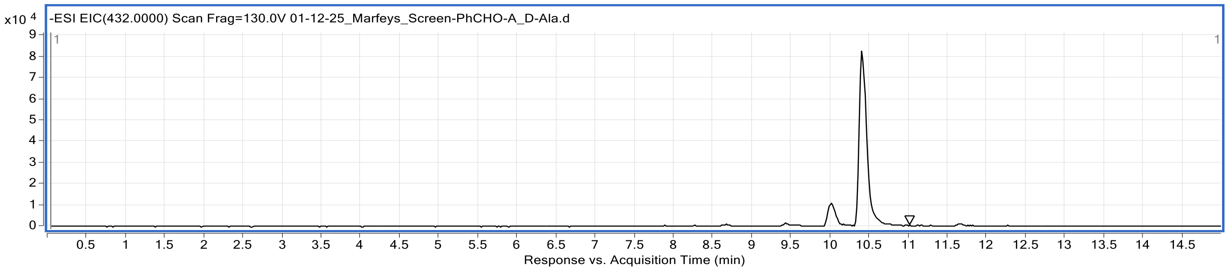

Zoomed and rescaled Extracted Ion (top) and Variable Wavelength Detector (bottom) Chromatograms. The Y axis on top is ion counts, the Y-axis on the bottom is absorbance units, and the X-axis for both is acquisition time in minutes. The peaks are labeled from left to right as Peak 1 and Peak 2.

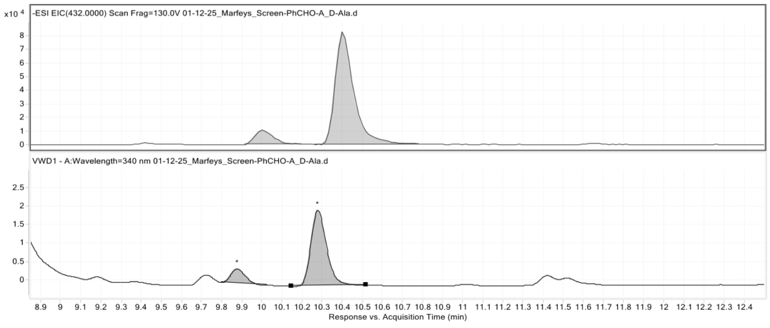

The following is a table of the peak data from the mass chromatogram. The Area % is the percent area relative to the tallest integrated peak, which has been set to 100%.

Peak	Assignment	t_R (min)	ES-ToF m/z [Neg]	Area %
1	L-*Erythro*	9.998	432.1151	12.4
2	L-*Threo*	10.396	432.1131	100

The calculated m/z for the adduct is 432.1161 for $C_{18}H_{18}N_5O_8^-$ $[M - H]^{-1}$.

The following is a table of the peak data from the variable wavelength detector chromatogram (340 nm). The Area % is the percent area relative to the tallest integrated peak, which has been set to 100%.

Peak	Assignment	t_R (min)	Area %
1	L-*Erythro*	9.877	17.63
2	L-*Threo*	10.277	100

The following is a table showing the difference in retention time on the extracted ion chromatogram (i.e., m/z Δt_R) and variable wavelength detector (i.e., VWD Δt_R) between the two diastereomers (i.e., separation = absolute difference between L-*erythro* and L-*threo*). The Average Δt_R values are the average difference in retention time between the extracted ion and variable wavelength detector chromatograms.

	m/z Δt_R (min)	VWD Δt_R (min)	Average Δt_R (min)
Peak Separation	0.398	0.400	0.40

LC/MS Traces for the Attempted Resolution of the Beta Stereoisomers of the β-hydroxy-α-Amino Acid Corresponding to Adduct 3 with 1-Fluoro-2,4-dinitrophenyl-5-L-proline Amide (i.e., L-FDPA 9) with HPLC Method B (i.e., 10 min.)

Total Ion Mass Chromatogram (ESI-ToF). The Y-axis is ion counts, and the X-axis is acquisition time in minutes.

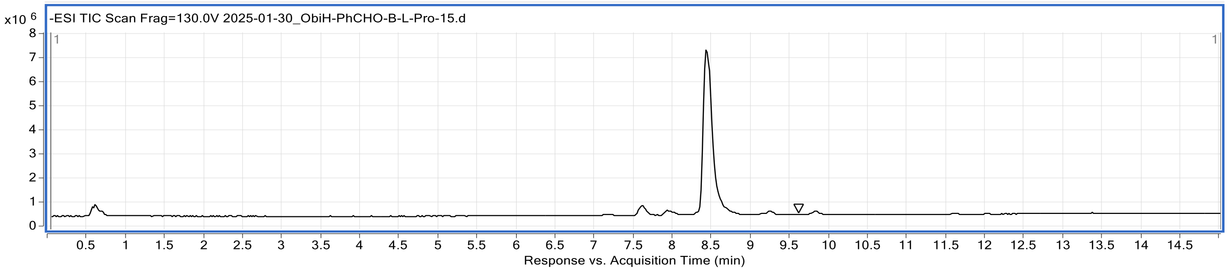

Variable Wavelength Detector Chromatogram (340 nm). The Y-axis is absorbance units, and the X-axis is acquisition time in minutes.

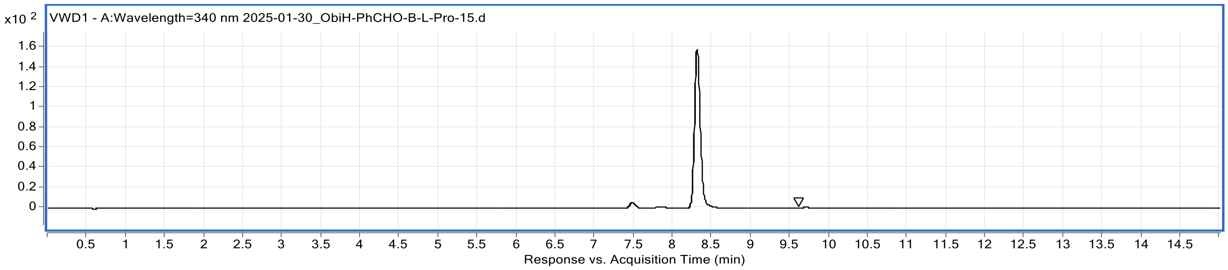

Extracted Ion Mass Chromatogram (ESI-ToF, extracted for m/z 458 ± 0.5). The Y-axis is ion counts, and the X-axis is acquisition time in minutes.

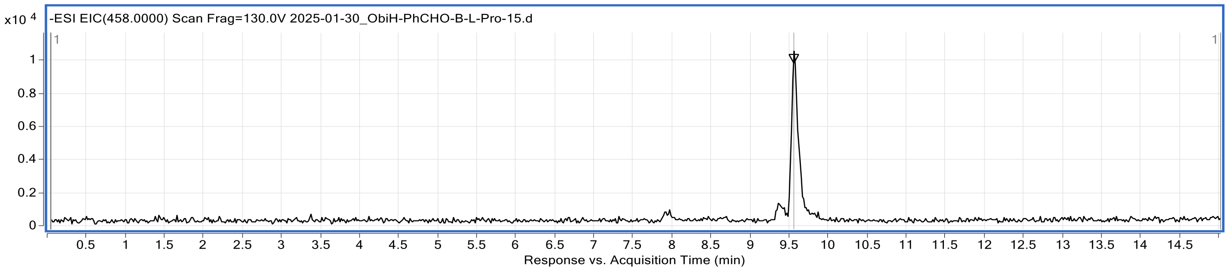

Zoomed and rescaled Extracted Ion (top) and Variable Wavelength Detector (bottom) Chromatograms. The Y axis on top is ion counts, the Y-axis on the bottom is absorbance units, and the X-axis for both is acquisition time in minutes. The peaks are labeled from left to right as Peak 1 and Peak 2.

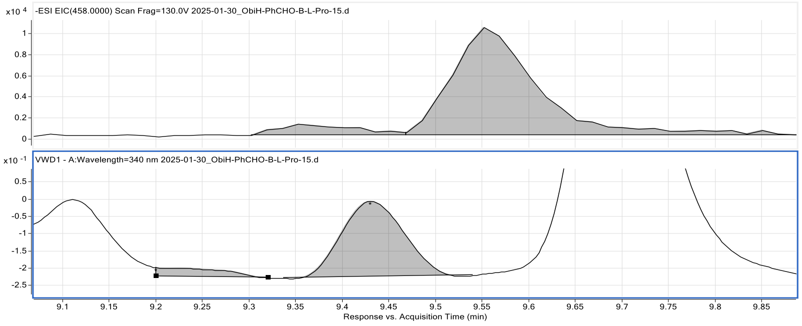

The following is a table of the peak data from the mass chromatogram. The Area % is the percent area relative to the tallest integrated peak, which has been set to 100%.

Peak	Assignment	t_R (min)	ES-ToF m/z [Neg]	Area %
1	L-*Erythro*	9.369	458.1317	10.4
2	L-*Threo*	9.551	458.1339	100

The calculated m/z for the adduct is 458.1317 for $C_{20}H_{20}N_5O_8^-$ $[M - H]^{-1}$.

The following is a table of the peak data from the variable wavelength detector chromatogram (340 nm). The Area % is the percent area relative to the tallest integrated peak, which has been set to 100%.

Peak	Assignment	t_R (min)	Area %
1	L-*Erythro*	9.252	12.75
2	L-*Threo*	9.430	100

The following is a table showing the difference in retention time on the extracted ion chromatogram (i.e., m/z Δt_R) and variable wavelength detector (i.e., VWD Δt_R) between the two diastereomers (i.e., separation = absolute difference between L-*erythro* and L-*threo*). The Average Δt_R values are the average difference in retention time between the extracted ion and variable wavelength detector chromatograms.

	m/z Δt_R (min)	VWD Δt_R (min)	Average Δt_R (min)
Peak Separation	0.182	0.178	0.18

LC/MS Traces for the Attempted Resolution of the Beta Stereoisomers of the β-hydroxy-α-Amino Acid Corresponding to Adduct 3 with 1-Fluoro-2,4-dinitrophenyl-5-D-proline Amide (i.e., D-FDPA D-9) with HPLC Method B (i.e., 10 min.)

Total Ion Mass Chromatogram (ESI-ToF). The Y-axis is ion counts, and the X-axis is acquisition time in minutes.

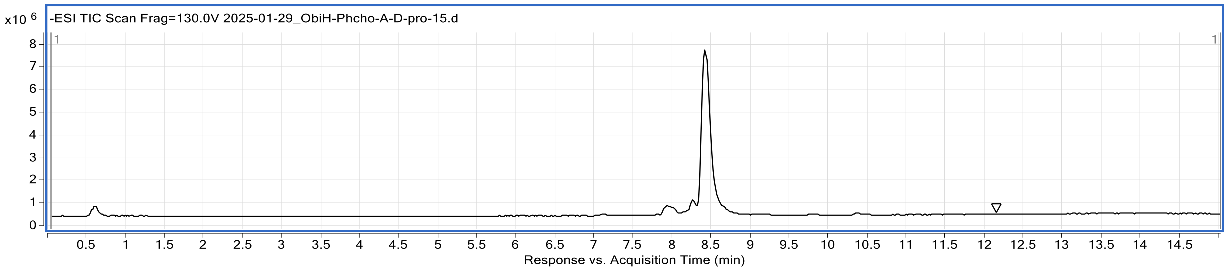

Variable Wavelength Detector Chromatogram (340 nm). The Y-axis is absorbance units, and the X-axis is acquisition time in minutes.

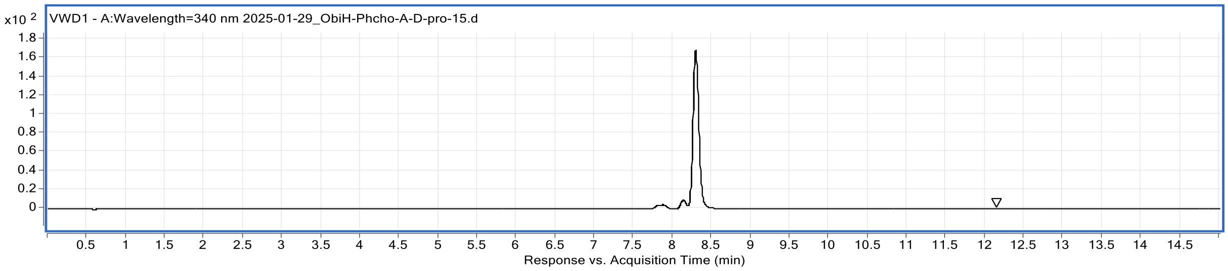

Extracted Ion Mass Chromatogram (ESI-ToF, extracted for m/z 458 ± 0.5). The Y-axis is ion counts, and the X-axis is acquisition time in minutes. Control experiments confirmed that the spurious peak at 8.7 min did not result from stereoisomer of the amino acid.

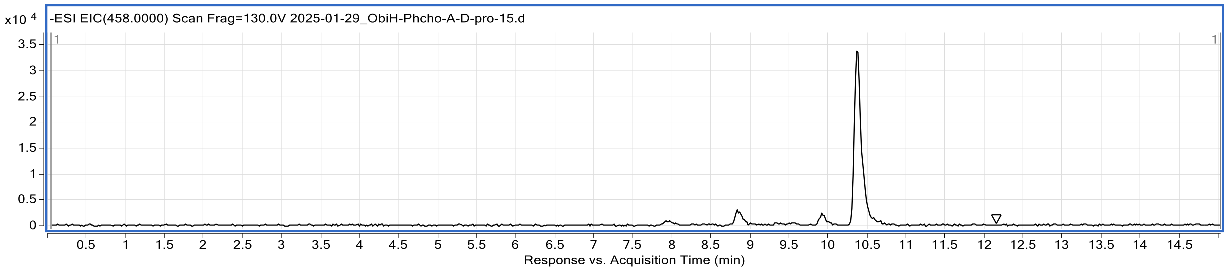

Zoomed and rescaled Extracted Ion (top) and Variable Wavelength Detector (bottom) Chromatograms. The Y axis on top is ion counts, the Y-axis on the bottom is absorbance units, and the X-axis for both is acquisition time in minutes. The peaks are labeled from left to right as Peak 1 and Peak 2.

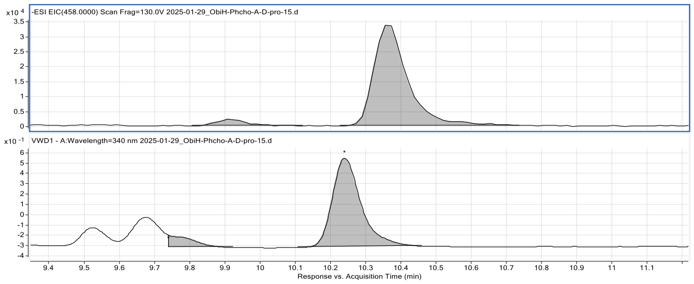

The following is a table of the peak data from the mass chromatogram. The Area % is the percent area relative to the tallest integrated peak, which has been set to 100%.

Peak	Assignment	t_R (min)	ES-ToF m/z [Neg]	Area %
1	L-*Erythro*	9.906	458.1302	6.06
2	L-*Threo*	10.354	458.1314	100

The calculated m/z for the adduct is 458.1317 for $C_{20}H_{20}N_5O_8^-$ $[M - H]^{-1}$.

The following is a table of the peak data from the variable wavelength detector chromatogram (340 nm). The Area % is the percent area relative to the tallest integrated peak, which has been set to 100%.

Peak	Assignment	t_R (min)	Area %
1	L-*Erythro*	9.740	10.85
2	L-*Threo*	10.240	100

The following is a table showing the difference in retention time on the extracted ion chromatogram (i.e., m/z Δt_R) and variable wavelength detector (i.e., VWD Δt_R) between the two diastereomers (i.e., separation = absolute difference between L-*erythro* and L-*threo*). The Average Δt_R values are the average difference in retention time between the extracted ion and variable wavelength detector chromatograms.

	m/z Δt_R (min)	VWD Δt_R (min)	Average Δt_R (min)
Peak Separation	0.448	0.500	0.47

LC/MS Traces for the Attempted Resolution of the Beta Stereoisomers of the β-hydroxy-α-Amino Acid Corresponding to Adduct 3 with 1-Fluoro-2,4-dinitrobenzene (i.e., Sanger's Reagent 10) with HPLC Method B (i.e., 10 min.)

Total Ion Mass Chromatogram (ESI-ToF). The Y-axis is ion counts, and the X-axis is acquisition time in minutes.

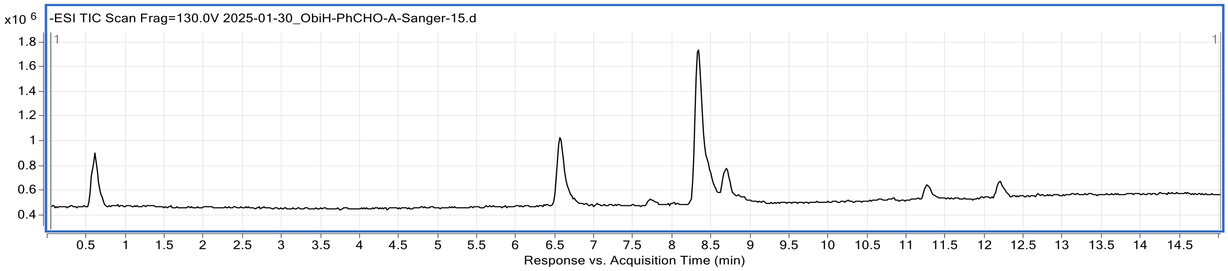

Variable Wavelength Detector Chromatogram (340 nm). The Y-axis is absorbance units, and the X-axis is acquisition time in minutes.

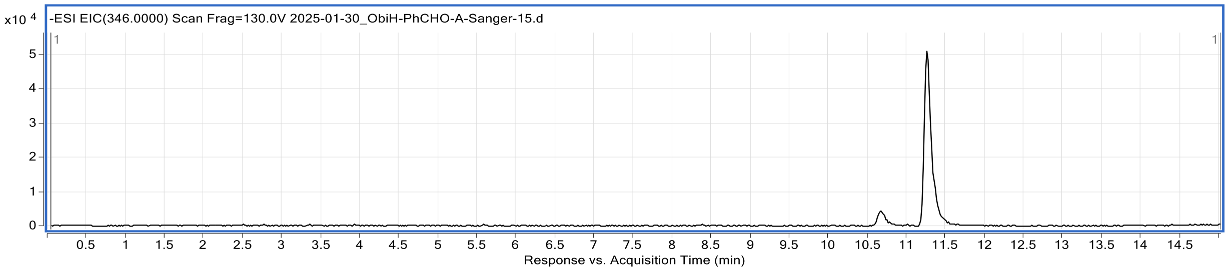

Extracted Ion Mass Chromatogram (ESI-ToF, extracted for m/z 346 ± 0.5). The Y-axis is ion counts, and the X-axis is acquisition time in minutes.

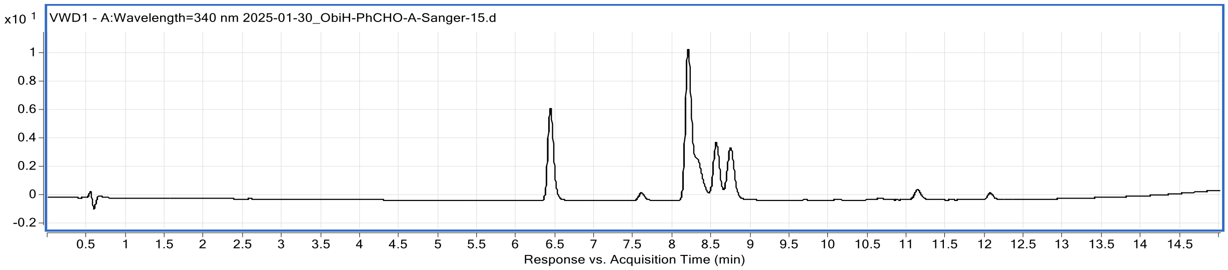

Zoomed and rescaled Extracted Ion (top) and Variable Wavelength Detector (bottom) Chromatograms. The Y axis on top is ion counts, the Y-axis on the bottom is absorbance units, and the X-axis for both is acquisition time in minutes. The peaks are labeled from left to right as Peak 1 and Peak 2.

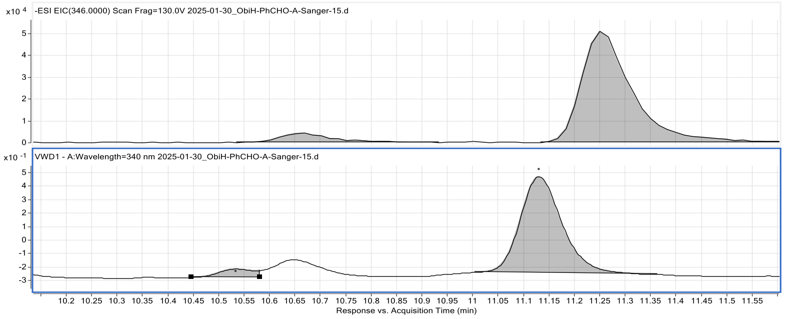

The following is a table of the peak data from the mass chromatogram. The Area % is the percent area relative to the tallest integrated peak, which has been set to 100%.

Peak	Assignment	t_R (min)	ES-ToF m/z [Neg]	Area %
1	L-*Erythro*	10.668	346.0679	9.01
2	L-*Threo*	11.248	346.0686	100

The calculated m/z for the adduct with Sanger's reagent is 346.0681 for $C_{15}H_{12}N_3O_7^-$ $[M – H]^{-1}$.

The following is a table of the peak data from the variable wavelength detector chromatogram (340 nm). The Area % is the percent area relative to the tallest integrated peak, which has been set to 100%.

Peak	Assignment	t_R (min)	Area %
1	L-*Erythro*	10.533	7.67
2	L-*Threo*	11.130	100

The following is a table showing the difference in retention time on the extracted ion chromatogram (i.e., m/z Δt_R) and variable wavelength detector (i.e., VWD Δt_R) between the two diastereomers (i.e., separation = absolute difference between L-*erythro* and L-*threo*). The Average Δt_R values are the average difference in retention time between the extracted ion and variable wavelength detector chromatograms.

	m/z Δt_R (min)	VWD Δt_R (min)	Average Δt_R (min)
Peak Separation	0.580	0.597	0.59

LC/MS Traces for the Attempted Resolution of the Beta Stereoisomers of the β-hydroxy-α-Amino Acid Corresponding to Adduct 20 with 1-Fluoro-2,4-dinitrophenyl-5-L-alanine Amide (i.e., L-FDAA 7) with HPLC Method B (i.e., 10 min.)

Total Ion Mass Chromatogram (ESI-ToF). The Y-axis is ion counts, and the X-axis is acquisition time in minutes.

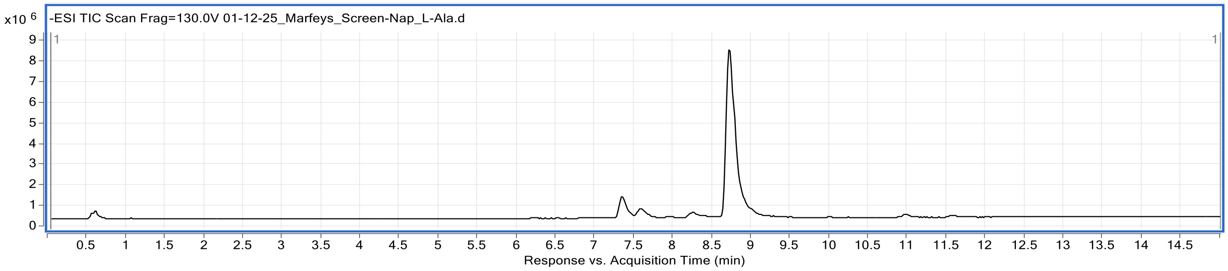

Variable Wavelength Detector Chromatogram (340 nm). The Y-axis is absorbance units, and the X-axis is acquisition time in minutes.

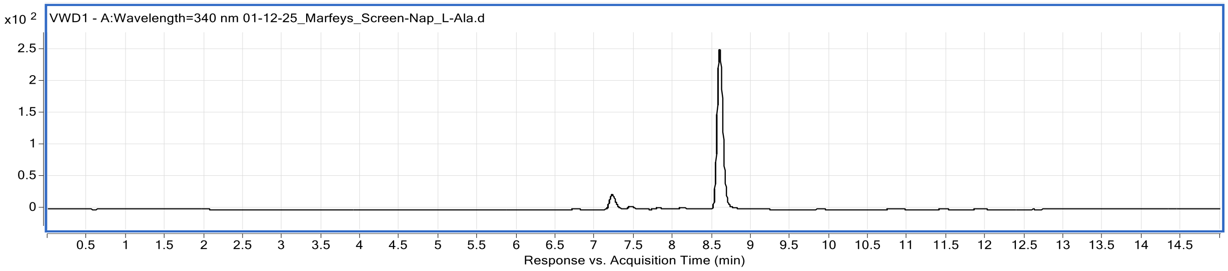

Extracted Ion Mass Chromatogram (ESI-ToF, extracted for m/z 482 ± 0.5). The Y-axis is ion counts, and the X-axis is acquisition time in minutes.

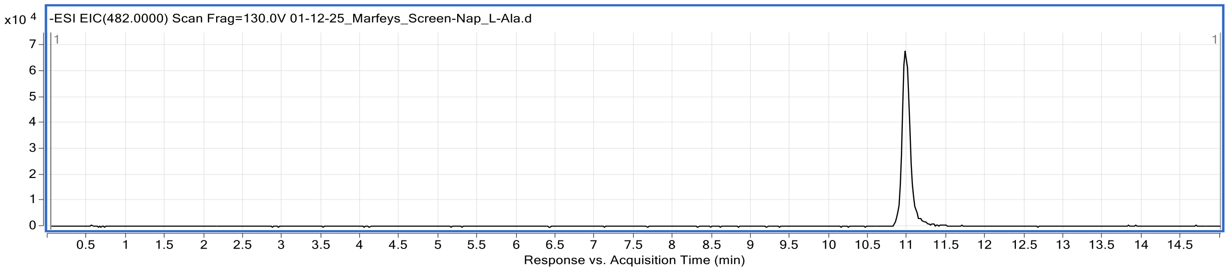

Zoomed and rescaled Extracted Ion (top) and Variable Wavelength Detector (bottom) Chromatograms. The Y axis on top is ion counts, the Y-axis on the bottom is absorbance units, and the X-axis for both is acquisition time in minutes. The minor adduct can be seen from the peak tailing, but the resolution is not sufficient to evaluate isomer separation.

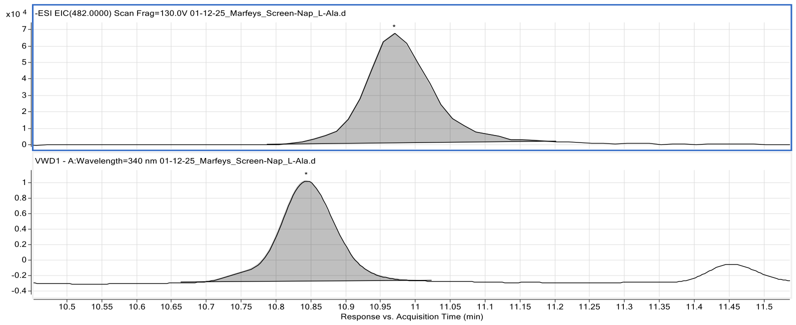

The following is a table of the peak data from the mass chromatogram. The Area % is the percent area relative to the tallest integrated peak, which has been set to 100%.

Peak	Assignment	t_R (min)	ES-ToF m/z [Neg]	Area %
1	L-*Erythro*/ L-*Threo*	10.970	482.1324	100

The calculated m/z for the adduct is 482.1317 for $C_{22}H_{20}N_5O_8^-$ $[M - H]^{-1}$.

The following is a table of the peak data from the variable wavelength detector chromatogram (340 nm). The Area % is the percent area relative to the tallest integrated peak, which has been set to 100%.

Peak	Assignment	t_R (min)	Area %
1	L-*Erythro*/ L-*Threo*	10.843	100

The following is a table showing the difference in retention time on the extracted ion chromatogram (i.e., m/z Δt_R) and variable wavelength detector (i.e., VWD Δt_R) between the two diastereomers (i.e., separation = absolute difference between L-*erythro* and L-*threo*). The Average Δt_R values are the average difference in retention time between the extracted ion and variable wavelength detector chromatograms.

	m/z Δt_R (min)	VWD Δt_R (min)	Average Δt_R (min)
Peak Separation	0.100	0.093	0.10

LC/MS Traces for the Attempted Resolution of the Beta Stereoisomers of the β-hydroxy-α-Amino Acid Corresponding to Adduct 20 with 1-Fluoro-2,4-dinitrophenyl-5-D-alanine Amide (i.e., D-FDAA D-7) with HPLC Method B (i.e., 10 min.)

Total Ion Mass Chromatogram (ESI-ToF). The Y-axis is ion counts, and the X-axis is acquisition time in minutes.

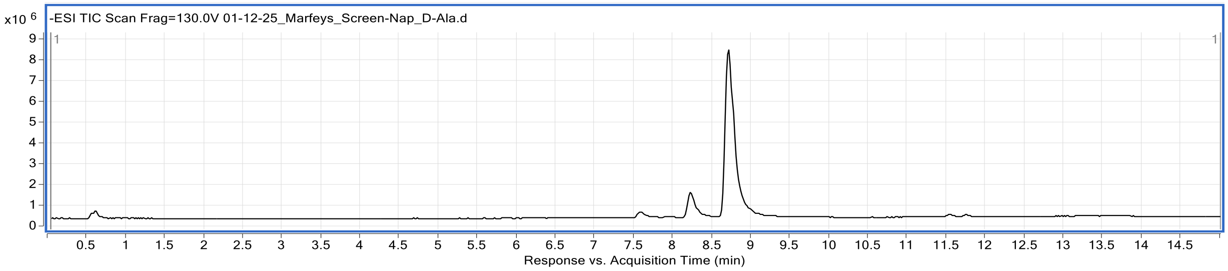

Variable Wavelength Detector Chromatogram (340 nm). The Y-axis is absorbance units, and the X-axis is acquisition time in minutes.

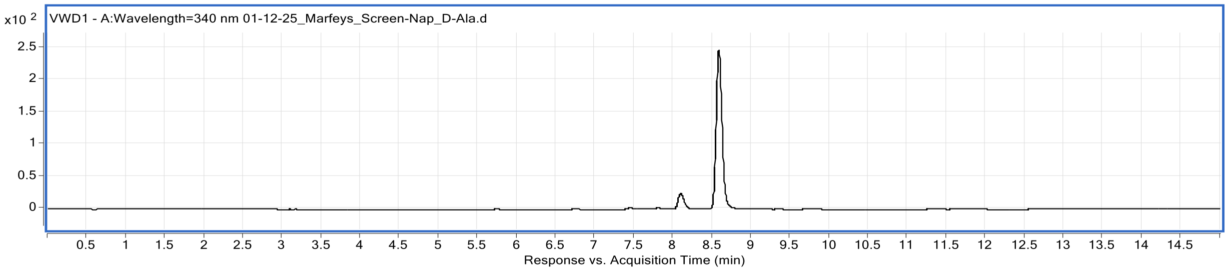

Extracted Ion Mass Chromatogram (ESI-ToF, extracted for m/z 482 ± 0.5). The Y-axis is ion counts, and the X-axis is acquisition time in minutes.

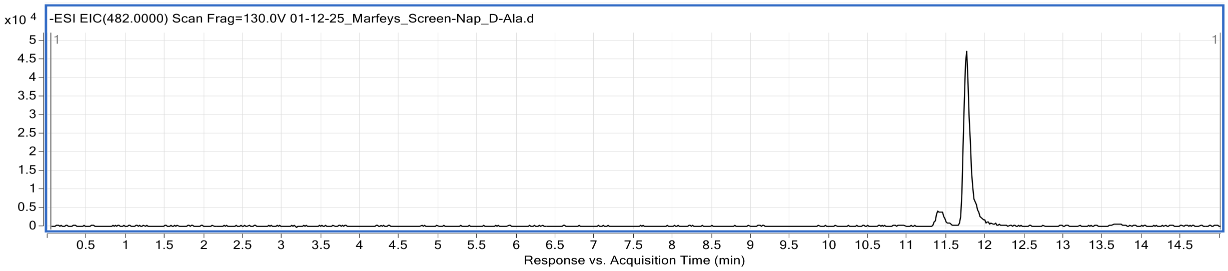

Zoomed and rescaled Extracted Ion (top) and Variable Wavelength Detector (bottom) Chromatograms. The Y axis on top is ion counts, the Y-axis on the bottom is absorbance units, and the X-axis for both is acquisition time in minutes. The peaks are labeled from left to right as Peak 1 and Peak 2.

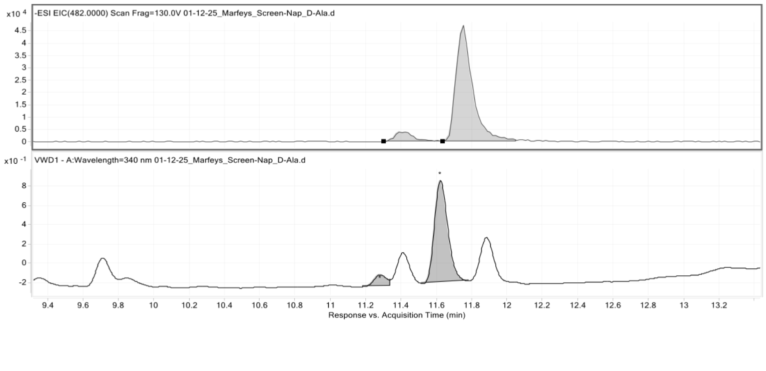

The following is a table of the peak data from the mass chromatogram. The Area % is the percent area relative to the tallest integrated peak, which has been set to 100%.

Peak	Assignment	t_R (min)	ES-ToF m/z [Neg]	Area %
1	L-*Erythro*	11.385	482.1319	9.79
2	L-*Threo*	11.75	482.1311	100

The calculated m/z for the adduct is 482.1317 for $C_{22}H_{20}N_5O_8^-$ $[M - H]^{-1}$.

The following is a table of the peak data from the variable wavelength detector chromatogram (340 nm). The Area % is the percent area relative to the tallest integrated peak, which has been set to 100%.

Peak	Assignment	t_R (min)	Area %
1	L-*Erythro*	11.280	10.97
2	L-*Threo*	11.620	100

The following is a table showing the difference in retention time on the extracted ion chromatogram (i.e., m/z Δt_R) and variable wavelength detector (i.e., VWD Δt_R) between the two diastereomers (i.e., separation = absolute difference between L-*erythro* and L-*threo*). The Average Δt_R values are the average difference in retention time between the extracted ion and variable wavelength detector chromatograms.

	m/z Δt_R (min)	VWD Δt_R (min)	Average Δt_R (min)
Peak Separation	0.365	0.340	0.35

LC/MS Traces for the Attempted Resolution of the Beta Stereoisomers of the β-hydroxy-α-Amino Acid Corresponding to Adduct 20 with 1-Fluoro-2,4-dinitrophenyl-5-L-proline Amide (i.e., L-FDPA 9) with HPLC Method B (i.e., 10 min.)

Total Ion Mass Chromatogram (ESI-ToF). The Y-axis is ion counts, and the X-axis is acquisition time in minutes.

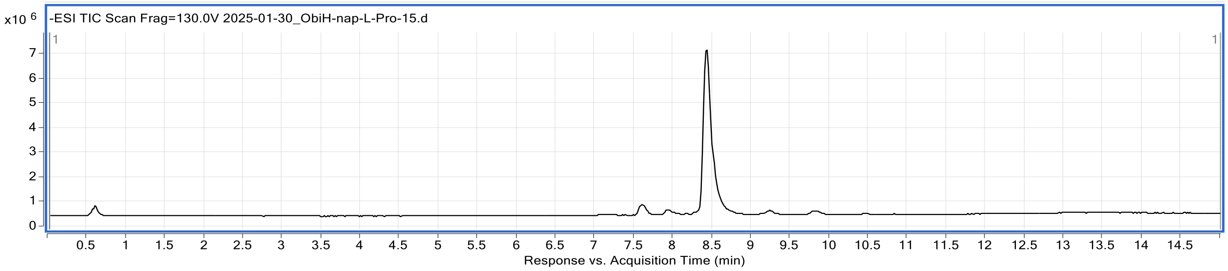

Variable Wavelength Detector Chromatogram (340 nm). The Y-axis is absorbance units, and the X-axis is acquisition time in minutes.

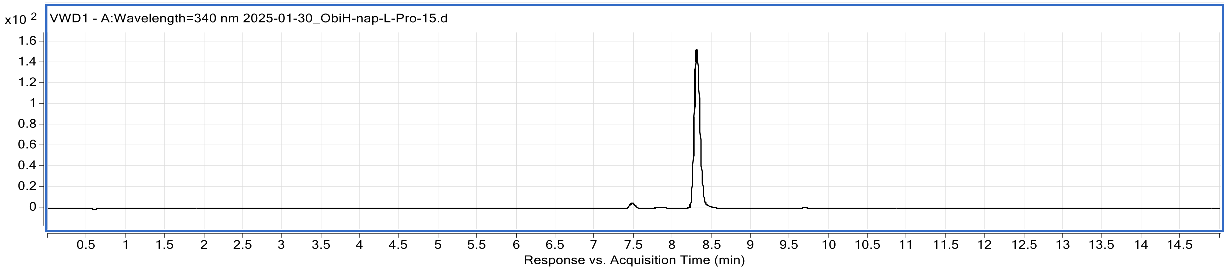

Extracted Ion Mass Chromatogram (ESI-ToF, extracted for m/z 508 ± 0.5). The Y-axis is ion counts, and the X-axis is acquisition time in minutes.

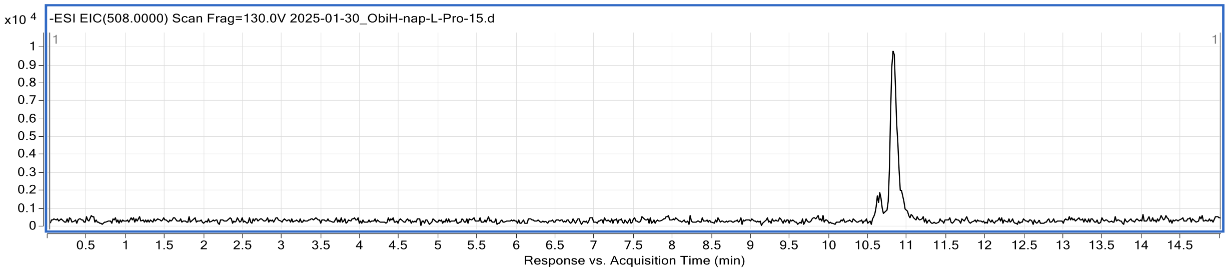

Zoomed and rescaled Extracted Ion (top) and Variable Wavelength Detector (bottom) Chromatograms. The Y axis on top is ion counts, the Y-axis on the bottom is absorbance units, and the X-axis for both is acquisition time in minutes. The peaks are labeled from left to right as Peak 1 and Peak 2.

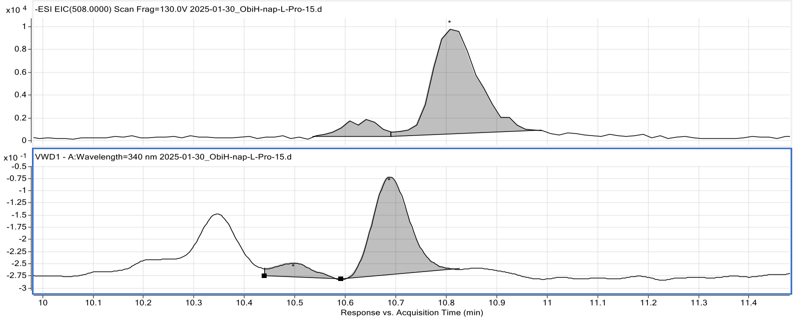

The following is a table of the peak data from the mass chromatogram. The Area % is the percent area relative to the tallest integrated peak, which has been set to 100%.

Peak	Assignment	t_R (min)	ES-ToF m/z [Neg]	Area %
1	L-*Erythro*	10.641	508.1476	12.86
2	L-*Threo*	10.807	508.1484	100

The calculated m/z for the adduct is 508.1474 for $C_{24}H_{22}N_5O_8^-$ $[M - H]^{-1}$.

The following is a table of the peak data from the variable wavelength detector chromatogram (340 nm). The Area % is the percent area relative to the tallest integrated peak, which has been set to 100%.

Peak	Assignment	t_R (min)	Area %
1	L-*Erythro*	10.497	15.61
2	L-*Threo*	10.687	100

The following is a table showing the difference in retention time on the extracted ion chromatogram (i.e., m/z Δt_R) and variable wavelength detector (i.e., VWD Δt_R) between the two diastereomers (i.e., separation = absolute difference between L-*erythro* and L-*threo*). The Average Δt_R values are the average difference in retention time between the extracted ion and variable wavelength detector chromatograms.

	m/z Δt_R (min)	VWD Δt_R (min)	Average Δt_R (min)
Peak Separation	0.166	0.190	0.18

LC/MS Traces for the Attempted Resolution of the Beta Stereoisomers of the β-hydroxy-α-Amino Acid Corresponding to Adduct 20 with 1-Fluoro-2,4-dinitrophenyl-5-D-proline Amide (i.e., D-FDPA D-9) with HPLC Method B (i.e., 10 min.)

Total Ion Mass Chromatogram (ESI-ToF). The Y-axis is ion counts, and the X-axis is acquisition time in minutes.

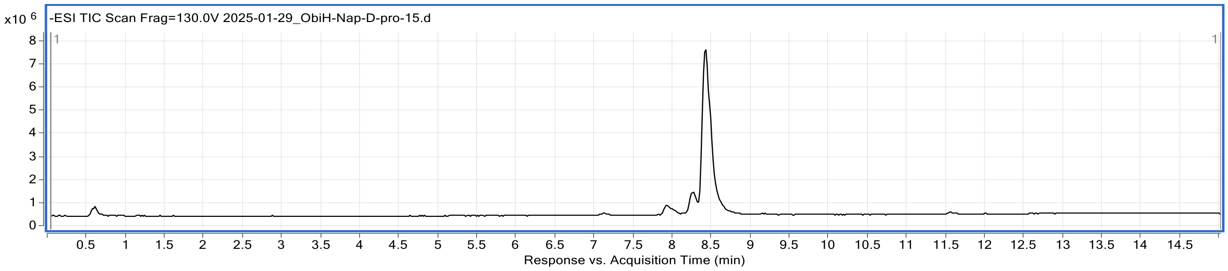

Variable Wavelength Detector Chromatogram (340 nm). The Y-axis is absorbance units, and the X-axis is acquisition time in minutes.

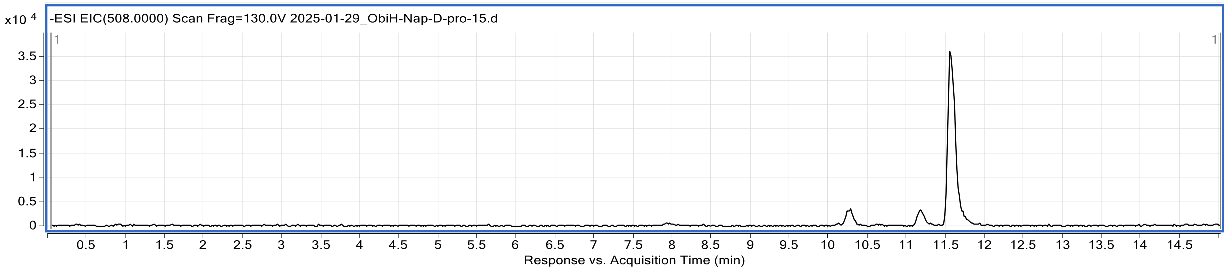

Extracted Ion Mass Chromatogram (ESI-ToF, extracted for m/z 508 ± 0.5). The Y-axis is ion counts, and the X-axis is acquisition time in minutes.

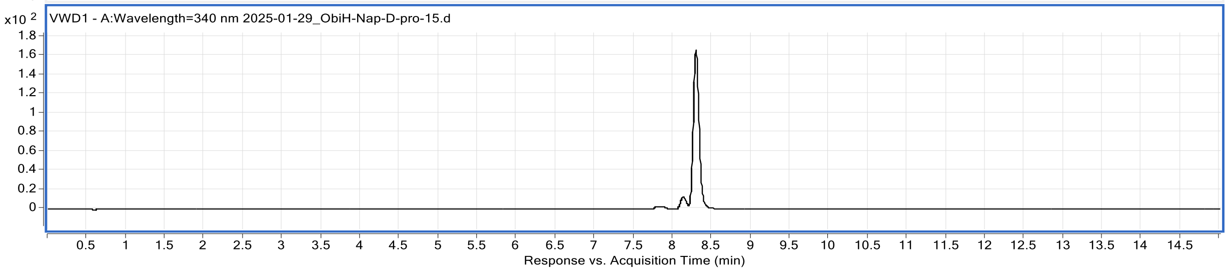

Zoomed and rescaled Extracted Ion (top) and Variable Wavelength Detector (bottom) Chromatograms. The Y axis on top is ion counts, the Y-axis on the bottom is absorbance units, and the X-axis for both is acquisition time in minutes. The peaks are labeled from left to right as Peak 1 and Peak 2.

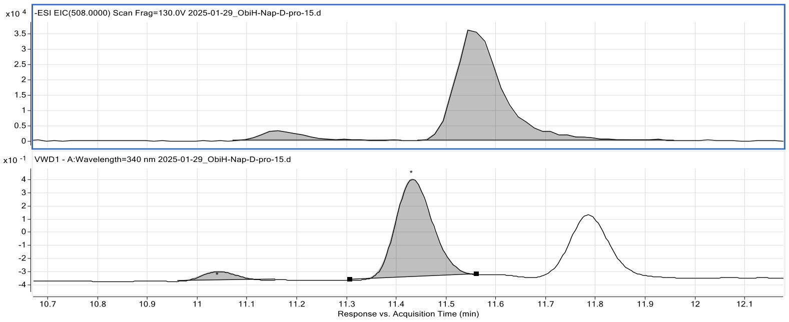

The following is a table of the peak data from the mass chromatogram. The Area % is the percent area relative to the tallest integrated peak, which has been set to 100%.

Peak	Assignment	t_R (min)	ES-ToF m/z [Neg]	Area %
1	L-*Erythro*	11.161	508.1485	8.71
2	L-*Threo*	11.543	508.1477	100

The calculated m/z for the adduct is 508.1474 for $C_{24}H_{22}N_5O_8^-$ $[M - H]^{-1}$.

The following is a table of the peak data from the variable wavelength detector chromatogram (340 nm). The Area % is the percent area relative to the tallest integrated peak, which has been set to 100%.

Peak	Assignment	t_R (min)	Area %
1	L-*Erythro*	11.040	8.53
2	L-*Threo*	11.430	100

The following is a table showing the difference in retention time on the extracted ion chromatogram (i.e., m/z Δt_R) and variable wavelength detector (i.e., VWD Δt_R) between the two diastereomers (i.e., separation = absolute difference between L-*erythro* and L-*threo*). The Average Δt_R values are the average difference in retention time between the extracted ion and variable wavelength detector chromatograms.

	m/z Δt_R (min)	VWD Δt_R (min)	Average Δt_R (min)
Peak Separation	0.382	0.390	0.39

LC/MS Traces for the Attempted Resolution of the Beta Stereoisomers of the β-hydroxy-α-Amino Acid Corresponding to Adduct 20 with 1-Fluoro-2,4-dinitrobenzene (i.e., Sanger's reagent 10) with HPLC Method B (i.e., 10 min.)

Total Ion Mass Chromatogram (ESI-ToF). The Y-axis is ion counts, and the X-axis is acquisition time in minutes.

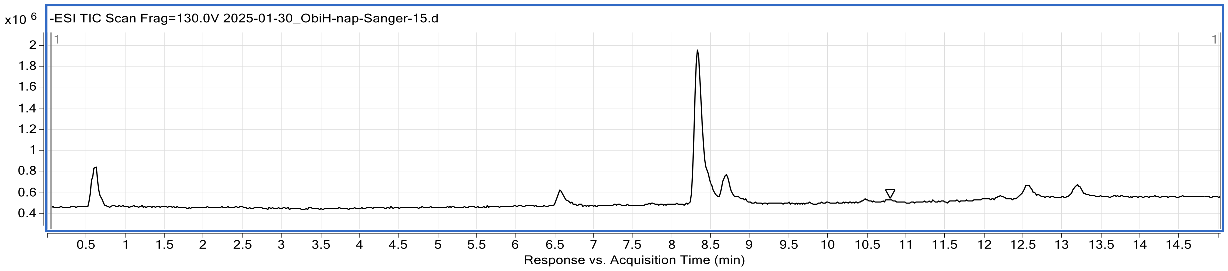

Variable Wavelength Detector Chromatogram (340 nm). The Y-axis is absorbance units, and the X-axis is acquisition time in minutes.

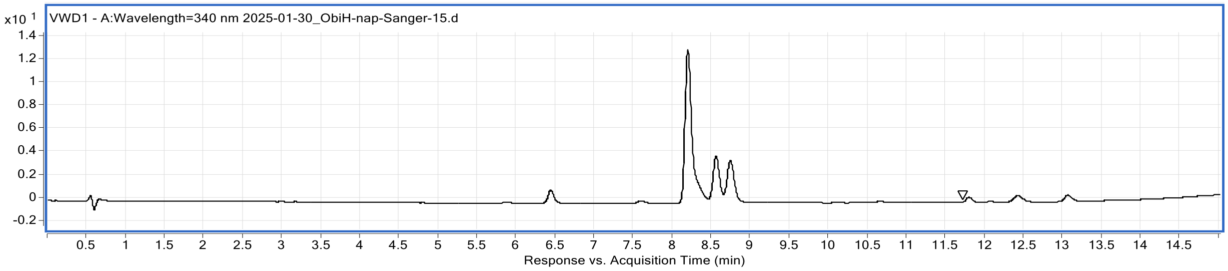

Extracted Ion Mass Chromatogram (ESI-ToF, extracted for m/z 396 ± 0.5). The Y-axis is ion counts, and the X-axis is acquisition time in minutes.

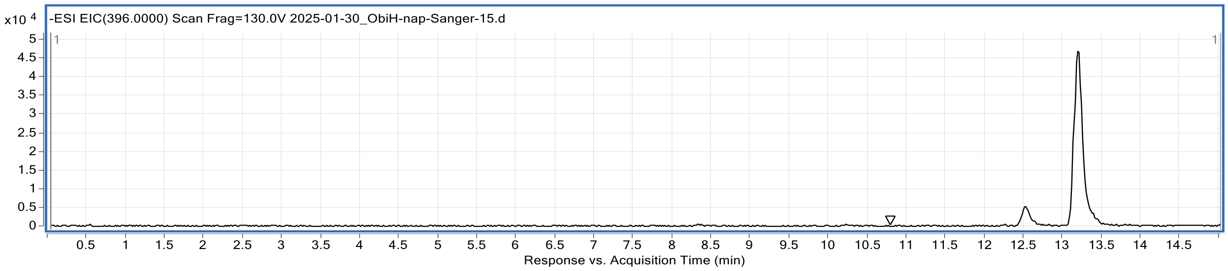

Zoomed and rescaled Extracted Ion (top) and Variable Wavelength Detector (bottom) Chromatograms. The Y axis on top is ion counts, the Y-axis on the bottom is absorbance units, and the X-axis for both is acquisition time in minutes. The peaks are labeled from left to right as Peak 1 and Peak 2.

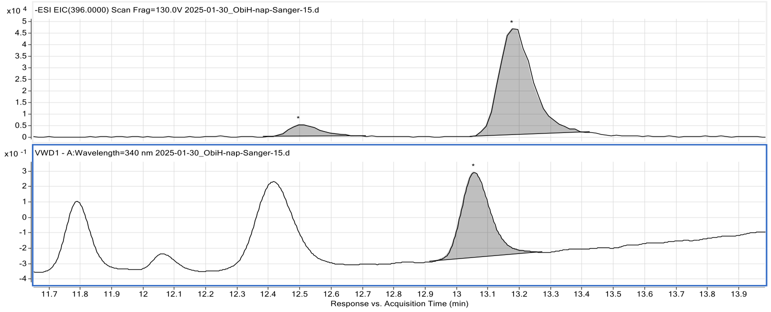

The following is a table of the peak data from the mass chromatogram. The Area % is the percent area relative to the tallest integrated peak, which has been set to 100%.

Peak	Assignment	t_R (min)	ES-ToF m/z [Neg]	Area %
1	L-*Erythro*	12.495	396.0842	10.91
2	L-*Threo*	13.176	396.0849	100

The calculated m/z for the adduct is 396.0837 for $C_{19}H_{14}N_3O_7^-$ $[M - H]^{-1}$.

The following is a table of the peak data from the variable wavelength detector chromatogram (340 nm). The Area % is the percent area relative to the tallest integrated peak, which has been set to 100%.

Peak	Assignment	t_R (min)	Area %
1	L-*Erythro*	12.413	NA*
2	L-*Threo*	13.053	100

The L-*erythro* peak had coincidental overlap with a 340 nm absorbing impurity, and its VWD area could not be accurately determined.

The following is a table showing the difference in retention time on the extracted ion chromatogram (i.e., m/z Δt_R) and variable wavelength detector (i.e., VWD Δt_R) between the two diastereomers (i.e., separation = absolute difference between L-*erythro* and L-*threo*). The Average Δt_R values are the average difference in retention time between the extracted ion and variable wavelength detector chromatograms.

	m/z Δt_R (min)	VWD Δt_R (min)	Average Δ t_R (min)
Peak Separation	0.681	0.640	0.66

LC/MS Traces for the Attempted Resolution of the Beta Stereoisomers of the β-hydroxy-α-Amino Acid Corresponding to Adduct 4 with 1-Fluoro-2,4-dinitrophenyl-5-L-alanine Amide (i.e., L-FDAA 7) with HPLC Method A (i.e., 25 min.)

Total Ion Mass Chromatogram (ESI-ToF). The Y-axis is ion counts, and the X-axis is acquisition time in minutes.

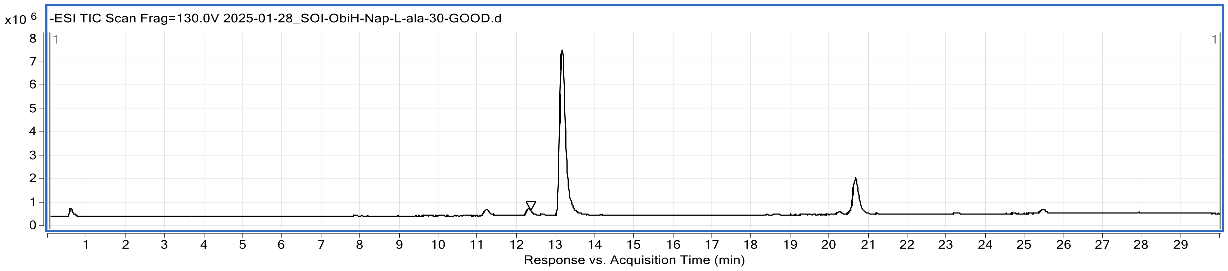

Variable Wavelength Detector Chromatogram (340 nm). The Y-axis is absorbance units, and the X-axis is acquisition time in minutes.

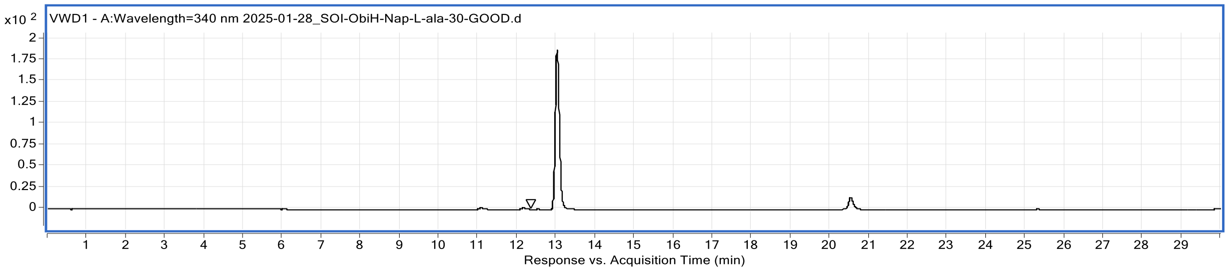

Extracted Ion Mass Chromatogram (ESI-ToF, extracted for m/z 496 ± 0.5). The Y-axis is ion counts, and the X-axis is acquisition time in minutes.

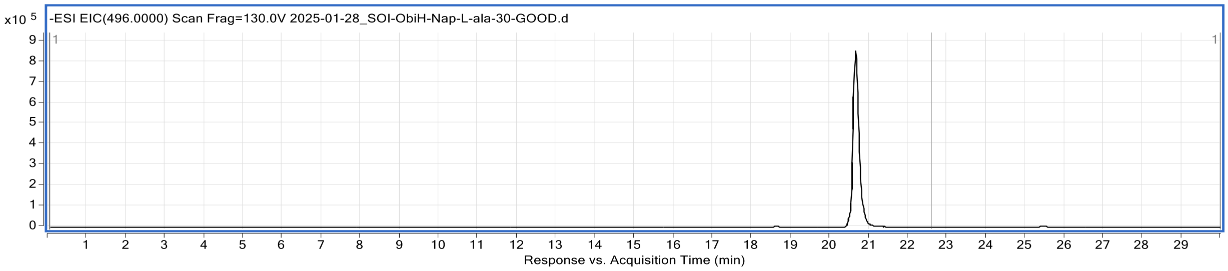

Zoomed and rescaled Extracted Ion (top) and Variable Wavelength Detector (bottom) Chromatograms. The Y axis on top is ion counts, the Y-axis on the bottom is absorbance units, and the X-axis for both is acquisition time in minutes. The minor adduct can be seen from the peak tailing, but the resolution is not sufficient to evaluate isomer separation.

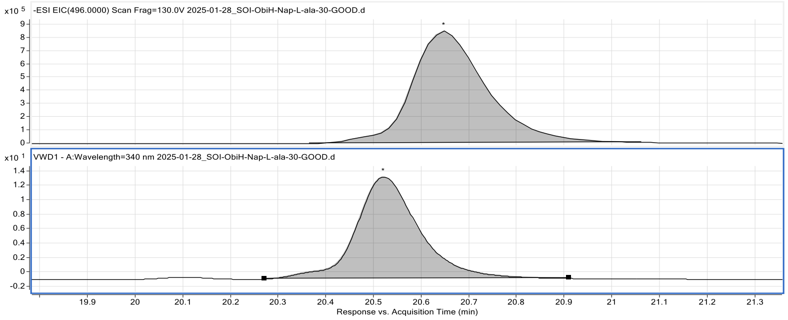

The following is a table of the peak data from the mass chromatogram. The Area % is the percent area relative to the tallest integrated peak, which has been set to 100%.

Peak	Assignment	t_R (min)	ES-ToF m/z [Neg]	Area %
1	L-*Erythro* and L-*Threo*	20.647	496.1499	100

The calculated m/z for the adduct is 496.1474 for $C_{23}H_{22}N_5O_8^-$ $[M – H]^{-1}$.

The following is a table of the peak data from the variable wavelength detector chromatogram (340 nm). The Area % is the percent area relative to the tallest integrated peak, which has been set to 100%.

Peak	Assignment	t_R (min)	Area %
1	L-*Erythro* and L-*Threo*	20.520	100

The following is a table showing the difference in retention time on the extracted ion chromatogram (i.e., m/z Δt_R) and variable wavelength detector (i.e., VWD Δt_R) between the two diastereomers (i.e., separation = absolute difference between L-*erythro* and L-*threo*). The Average Δt_R values are the average difference in retention time between the extracted ion and variable wavelength detector chromatograms.

	m/z Δt_R (min)	VWD Δt_R (min)	Average Δt_R (min)
Peak Separation	0	0	0

LC/MS Traces for the Attempted Resolution of the Beta Stereoisomers of the β-hydroxy-α-Amino Acid Corresponding to Adduct 4 with 1-Fluoro-2,4-dinitrophenyl-5-D-alanine Amide (i.e., D-FDAA D-7) with HPLC Method A (i.e., 25 min.)

Total Ion Mass Chromatogram (ESI-ToF). The Y-axis is ion counts, and the X-axis is acquisition time in minutes.

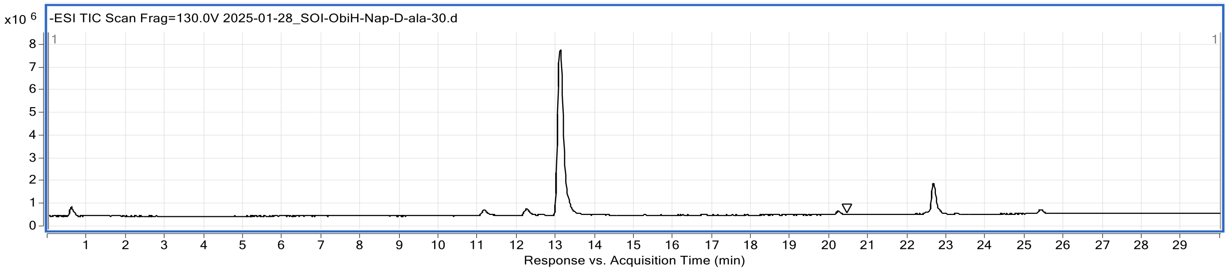

Variable Wavelength Detector Chromatogram (340 nm). The Y-axis is absorbance units, and the X-axis is acquisition time in minutes.

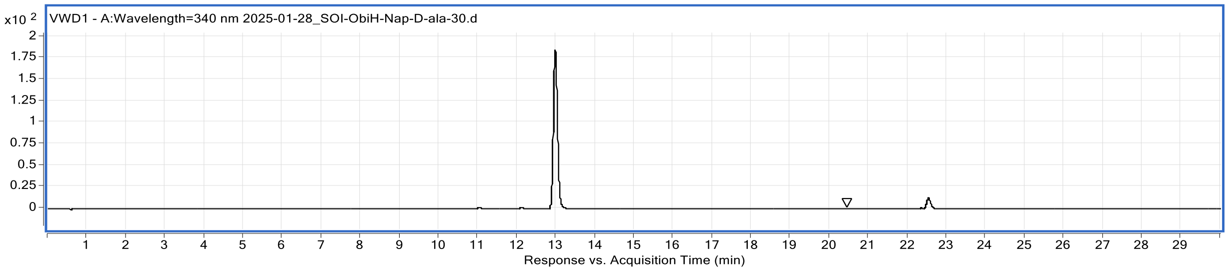

Extracted Ion Mass Chromatogram (ESI-ToF, extracted for m/z 496 ± 0.5). The Y-axis is ion counts, and the X-axis is acquisition time in minutes.

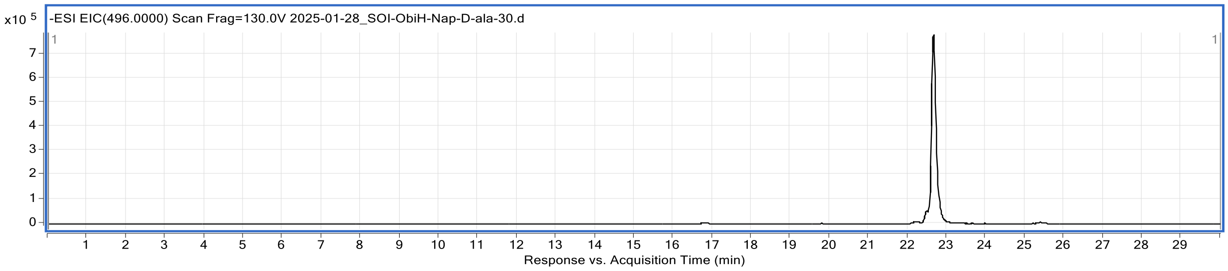

Zoomed and rescaled Extracted Ion (top) and Variable Wavelength Detector (bottom) Chromatograms. The Y axis on top is ion counts, the Y-axis on the bottom is absorbance units, and the X-axis for both is acquisition time in minutes. The peaks are labeled from left to right as Peak 1 and Peak 2.

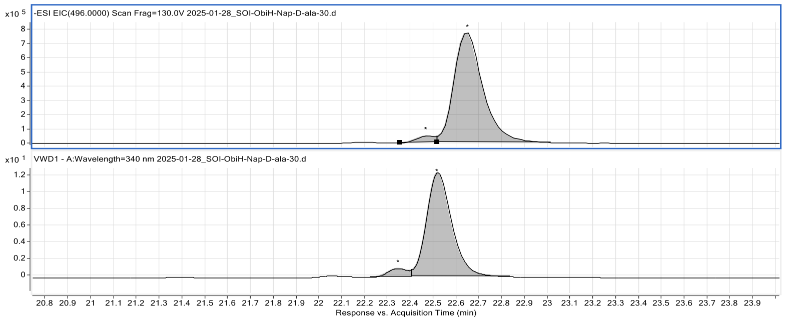

The following is a table of the peak data from the mass chromatogram. The Area % is the percent area relative to the tallest integrated peak, which has been set to 100%.

Peak	Assignment	t_R (min)	ES-ToF m/z [Neg]	Area %
1	L-*Erythro*	22.468	496.1495	4.01
2	L-*Threo*	22.65	496.1500	100

The calculated m/z for the adduct is 496.1474 for $C_{23}H_{22}N_5O_8^-$ $[M – H]^{-1}$.

The following is a table of the peak data from the variable wavelength detector chromatogram (340 nm). The Area % is the percent area relative to the tallest integrated peak, which has been set to 100%.

Peak	Assignment	t_R (min)	Area %
1	L-*Erythro*	22.347	6.88
2	L-*Threo*	22.517	100

The following is a table showing the difference in retention time on the extracted ion chromatogram (i.e., m/z Δt_R) and variable wavelength detector (i.e., VWD Δt_R) between the two diastereomers (i.e., separation = absolute difference between L-*erythro* and L-*threo*). The Average Δt_R values are the average difference in retention time between the extracted ion and variable wavelength detector chromatograms.

	m/z Δt_R (min)	VWD Δt_R (min)	Average Δt_R (min)
Peak Separation	0.182	0.170	0.18

LC/MS Traces for the Attempted Resolution of the Beta Stereoisomers of the β-hydroxy-α-Amino Acid Corresponding to Adduct 4 with 1-Fluoro-2,4-dinitrophenyl-5-L-proline Amide (i.e., L-FDPA 9) with HPLC Method A (i.e., 25 min.)

Total Ion Mass Chromatogram (ESI-ToF). The Y-axis is ion counts, and the X-axis is acquisition time in minutes.

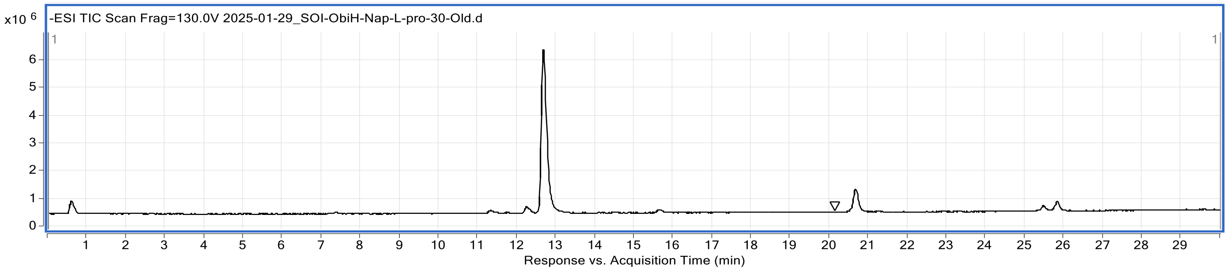

Variable Wavelength Detector Chromatogram (340 nm). The Y-axis is absorbance units, and the X-axis is acquisition time in minutes.

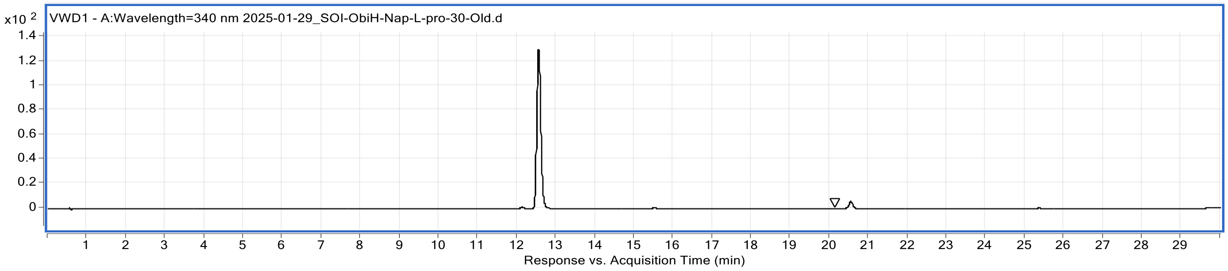

Extracted Ion Mass Chromatogram (ESI-ToF, extracted for m/z 522 ± 0.5). The Y-axis is ion counts, and the X-axis is acquisition time in minutes.

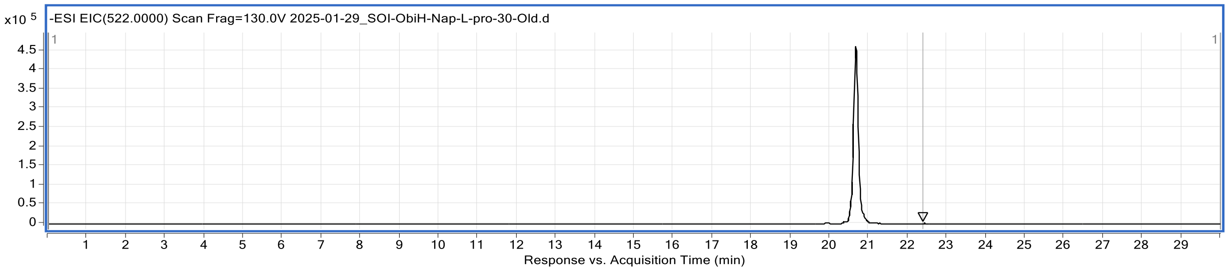

Zoomed and rescaled Extracted Ion (top) and Variable Wavelength Detector (bottom) Chromatograms. The Y axis on top is ion counts, the Y-axis on the bottom is absorbance units, and the X-axis for both is acquisition time in minutes. The minor adduct can be seen from the peak tailing, but the resolution is not sufficient to evaluate isomer separation.

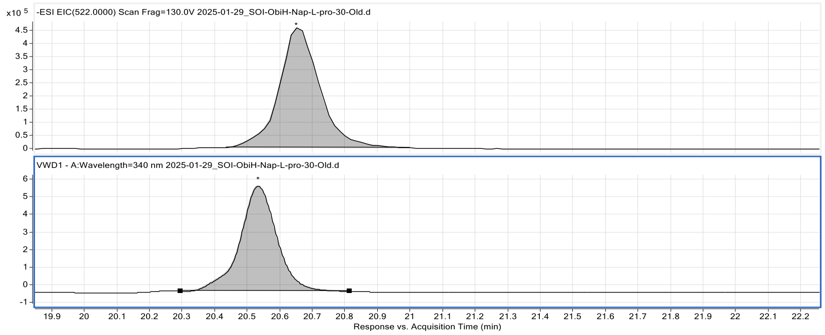

The following is a table of the peak data from the mass chromatogram. The Area % is the percent area relative to the tallest integrated peak, which has been set to 100%.

Peak	Assignment	t_R (min)	ES-ToF m/z [Neg]	Area %
1	L-*Erythro* and L-*Threo*	20.651	522.1656	100

The calculated m/z for the adduct is 522.1630 for $C_{25}H_{24}N_5O_8^-$ $[M - H]^{-1}$.

The following is a table of the peak data from the variable wavelength detector chromatogram (340 nm). The Area % is the percent area relative to the tallest integrated peak, which has been set to 100%.

Peak	Assignment	t_R (min)	Area %
1	L-*Erythro* and L-*Threo*	20.533	100

The following is a table showing the difference in retention time on the extracted ion chromatogram (i.e., m/z Δt_R) and variable wavelength detector (i.e., VWD Δt_R) between the two diastereomers (i.e., separation = absolute difference between L-*erythro* and L-*threo*). The Average Δt_R values are the average difference in retention time between the extracted ion and variable wavelength detector chromatograms.

	m/z Δt_R (min)	VWD Δt_R (min)	Average Δt_R (min)
Peak Separation	0	0	0

LC/MS Traces for the Attempted Resolution of the Beta Stereoisomers of the β-hydroxy-α-Amino Acid Corresponding to Adduct 4 with 1-Fluoro-2,4-dinitrophenyl-5-D-proline Amide (i.e., D-FDPA D-9) with HPLC Method A (i.e., 25 min.)

Total Ion Mass Chromatogram (ESI-ToF). The Y-axis is ion counts, and the X-axis is acquisition time in minutes.

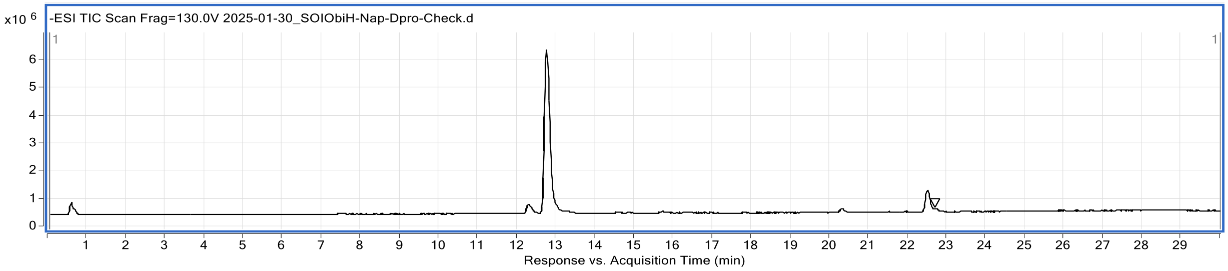

Variable Wavelength Detector Chromatogram (340 nm). The Y-axis is absorbance units, and the X-axis is acquisition time in minutes.

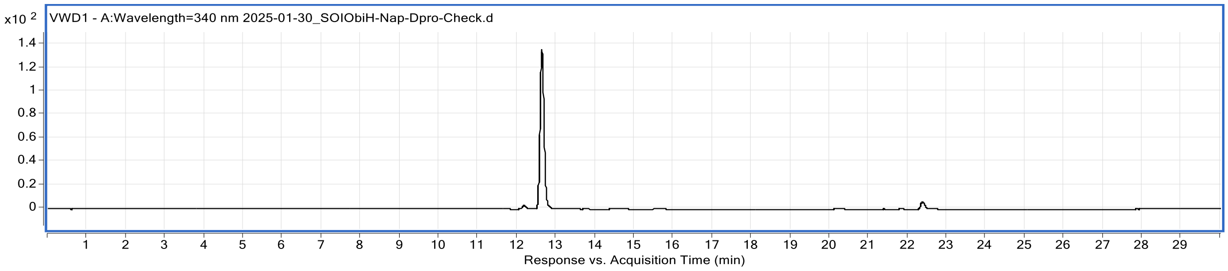

Extracted Ion Mass Chromatogram (ESI-ToF, extracted for m/z 522 ± 0.5). The Y-axis is ion counts, and the X-axis is acquisition time in minutes. Control experiments confirmed that the spurious peak at 20.4 min did not result from stereoisomer of the amino acid.

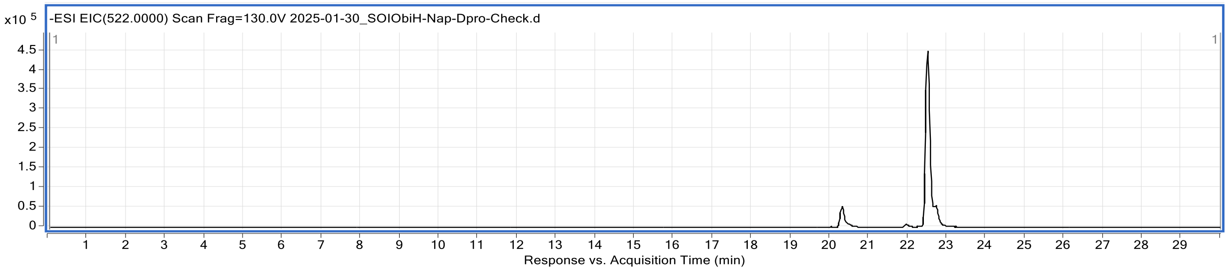

Zoomed and rescaled Extracted Ion (top) and Variable Wavelength Detector (bottom) Chromatograms. The Y axis on top is ion counts, the Y-axis on the bottom is absorbance units, and the X-axis for both is acquisition time in minutes. The peaks are labeled from left to right as Peak 1 and Peak 2.

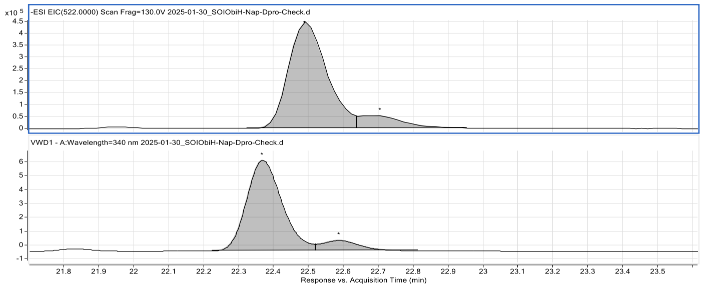

The following is a table of the peak data from the mass chromatogram. The Area % is the percent area relative to the tallest integrated peak, which has been set to 100%.

Peak	Assignment	t_R (min)	ES-ToF m/z [Neg]	Area %
1	L-*Threo*	22.489	522.1636	100
2	L-*Erythro*	22.705	522.1626	13.24

The calculated m/z for the adduct is 522.1630 for $C_{25}H_{24}N_5O_8^-$ $[M - H]^{-1}$.

The following is a table of the peak data from the variable wavelength detector chromatogram (340 nm). The Area % is the percent area relative to the tallest integrated peak, which has been set to 100%.

Peak	Assignment	t_R (min)	Area %
1	L-*Threo*	22.367	100
2	L-*Erythro*	22.587	11.89

The following is a table showing the difference in retention time on the extracted ion chromatogram (i.e., m/z Δt_R) and variable wavelength detector (i.e., VWD Δt_R) between the two diastereomers (i.e., separation = absolute difference between L-*erythro* and L-*threo*). The Average Δt_R values are the average difference in retention time between the extracted ion and variable wavelength detector chromatograms.

	m/z Δt_R (min)	VWD Δt_R (min)	Average Δt_R (min)
Peak Separation	0.216	0.220	0.22

LC/MS Traces for the Attempted Resolution of the Beta Stereoisomers of the β-hydroxy-α-Amino Acid Corresponding to Adduct 4 with 1-Fluoro-2,4-dinitrobenzene (i.e., Sanger's reagent 10) with HPLC Method A (i.e., 25 min.)

Total Ion Mass Chromatogram (ESI-ToF). The Y-axis is ion counts, and the X-axis is acquisition time in minutes.

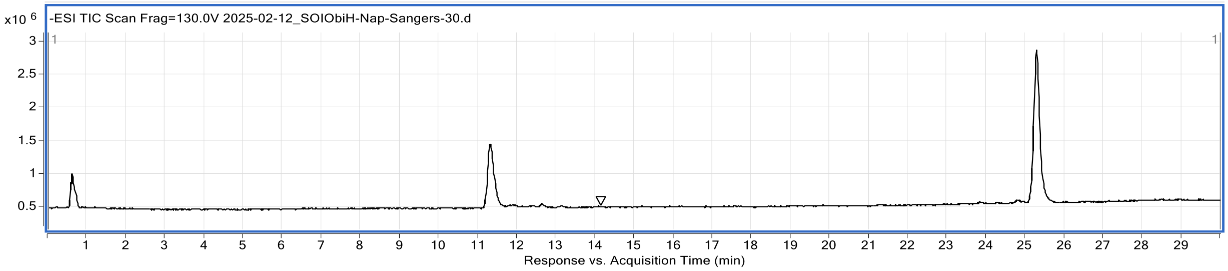

Variable Wavelength Detector Chromatogram (340 nm). The Y-axis is absorbance units, and the X-axis is acquisition time in minutes.

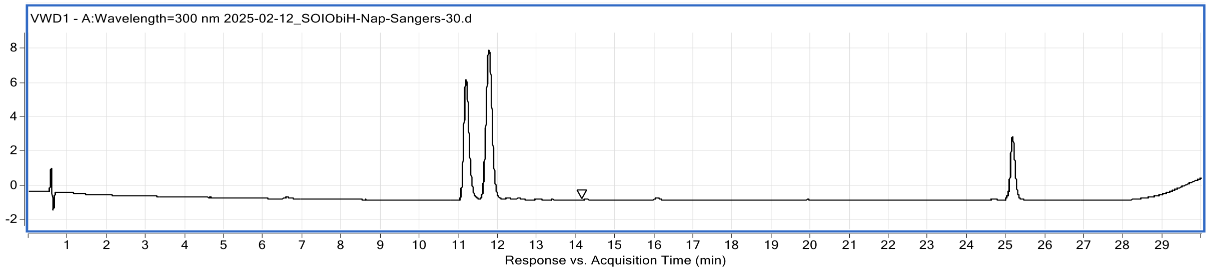

Extracted Ion Mass Chromatogram (ESI-ToF, extracted for m/z 410 ± 0.5). The Y-axis is ion counts, and the X-axis is acquisition time in minutes.

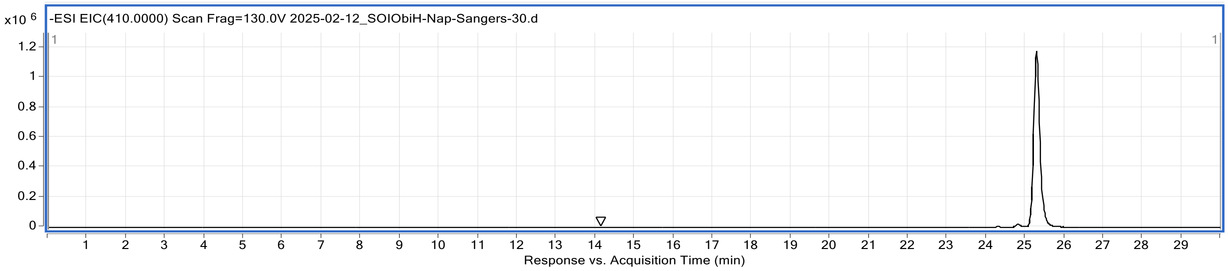

Zoomed and rescaled Extracted Ion (top) and Variable Wavelength Detector (bottom) Chromatograms. The Y axis on top is ion counts, the Y-axis on the bottom is absorbance units, and the X-axis for both is acquisition time in minutes. The peaks are labeled from left to right as Peak 1 and Peak 2.

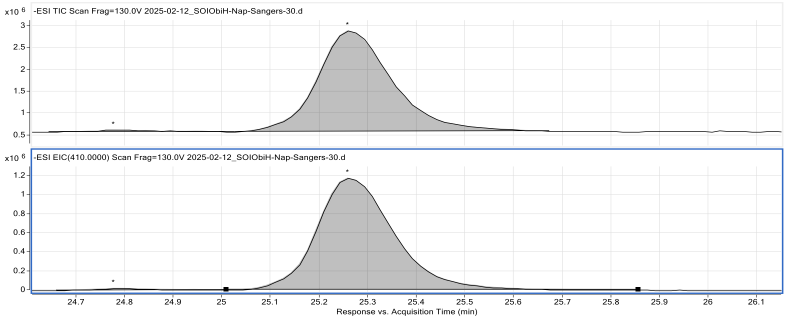

The following is a table of the peak data from the mass chromatogram. The Area % is the percent area relative to the tallest integrated peak, which has been set to 100%.

Peak	Assignment	t_R (min)	ES-ToF m/z [Neg]	Area %
1	L-*Erythro* and L-*Threo*	25.258	410.1047	100

The calculated m/z for the adduct is 410.0994 for $C_{20}H_{16}N_3O_7^-$ $[M - H]^{-1}$.

The following is a table of the peak data from the variable wavelength detector chromatogram (340 nm). The Area % is the percent area relative to the tallest integrated peak, which has been set to 100%.

Peak	Assignment	t_R (min)	Area %
1	L-*Erythro* and L-*Threo*	25.140	100

The following is a table showing the difference in retention time on the extracted ion chromatogram (i.e., m/z Δt_R) and variable wavelength detector (i.e., VWD Δt_R) between the two diastereomers (i.e., separation = absolute difference between L-*erythro* and L-*threo*). The Average Δt_R values are the average difference in retention time between the extracted ion and variable wavelength detector chromatograms.

	m/z Δt_R (min)	VWD Δt_R (min)	Average Δt_R (min)
Peak Separation	0	0	0

LC/MS Traces for the Attempted Resolution of the Beta Stereoisomers of the β-hydroxy-α-Amino Acid Corresponding to Adduct 21 with 1-Fluoro-2,4-dinitrophenyl-5-L-alanine Amide (i.e., L-FDAA 7) with HPLC Method B (i.e., 10 min.)

Total Ion Mass Chromatogram (ESI-ToF). The Y-axis is ion counts, and the X-axis is acquisition time in minutes.

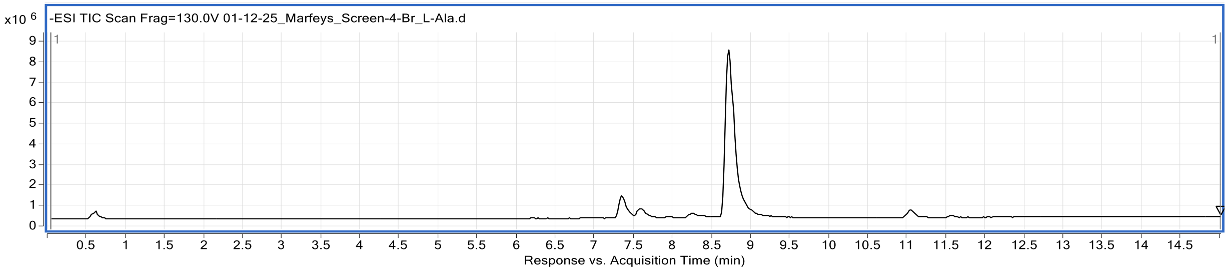

Variable Wavelength Detector Chromatogram (340 nm). The Y-axis is absorbance units, and the X-axis is acquisition time in minutes.

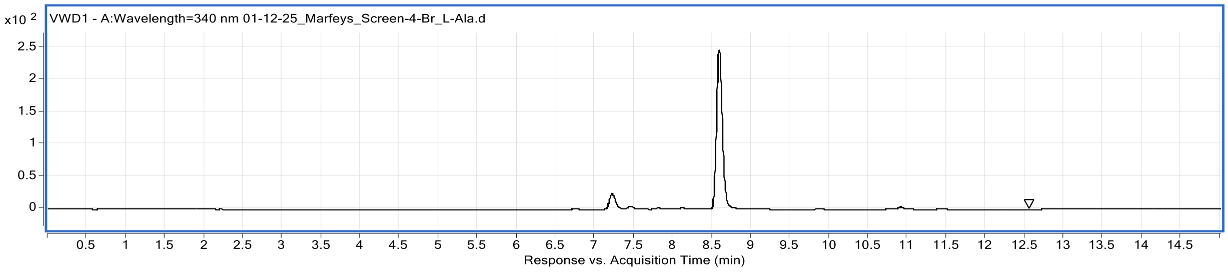

Extracted Ion Mass Chromatogram (ESI-ToF, extracted for m/z 510 ± 0.5). The Y-axis is ion counts, and the X-axis is acquisition time in minutes.

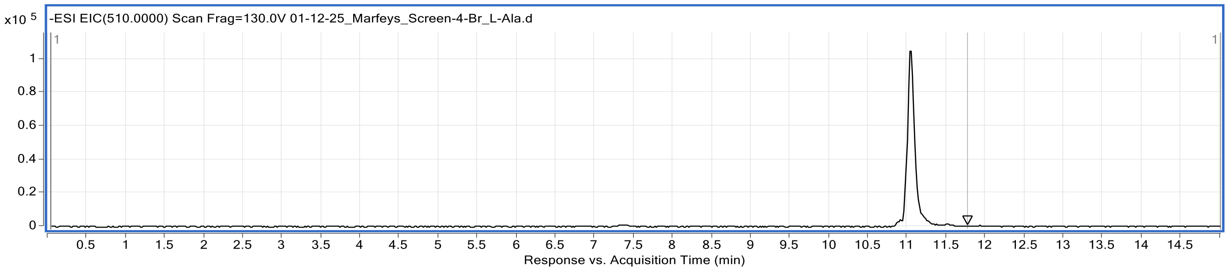

Zoomed and rescaled Extracted Ion (top) and Variable Wavelength Detector (bottom) Chromatograms. The Y axis on top is ion counts, the Y-axis on the bottom is absorbance units, and the X-axis for both is acquisition time in minutes. The peaks are labeled from left to right as Peak 1 and Peak 2.

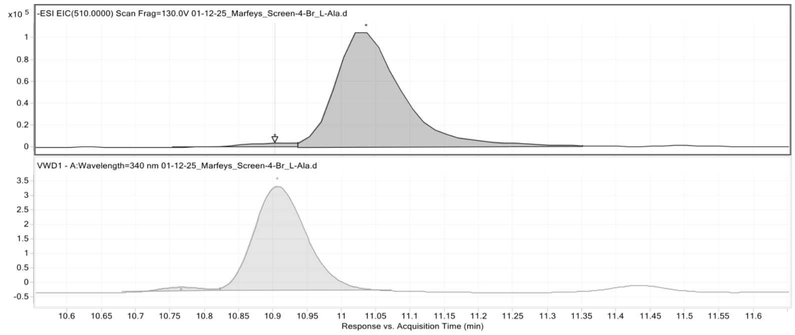

The following is a table of the peak data from the mass chromatogram. The Area % is the percent area relative to the tallest integrated peak, which has been set to 100%.

Peak	Assignment	t_R (min)	ES-ToF m/z [Neg]	Area %
1	L-*Erythro*	10.903	510.0254	3.1
2	L-*Threo*	11.036	510.0277	100

The calculated m/z for the adduct is 510.0266 for $C_{18}H_{17}BrN_5O_8^-$ $[M - H]^{-1}$.

The following is a table of the peak data from the variable wavelength detector chromatogram (340 nm). The Area % is the percent area relative to the tallest integrated peak, which has been set to 100%.

Peak	Assignment	t_R (min)	Area %
1	L-*Erythro*	10.767	3.76
2	L-*Threo*	10.907	100

The following is a table showing the difference in retention time on the extracted ion chromatogram (i.e., m/z Δt_R) and variable wavelength detector (i.e., VWD Δt_R) between the two diastereomers (i.e., separation = absolute difference between L-*erythro* and L-*threo*). The Average Δt_R values are the average difference in retention time between the extracted ion and variable wavelength detector chromatograms.

	m/z Δt_R (min)	VWD Δt_R (min)	Average Δt_R (min)
Peak Separation	0.133	0.140	0.14

LC/MS Traces for the Attempted Resolution of the Beta Stereoisomers of the β-hydroxy-α-Amino Acid Corresponding to Adduct 21 with 1-Fluoro-2,4-dinitrophenyl-5-D-alanine Amide (i.e., D-FDAA D-7) with HPLC Method B (i.e., 10 min.)

Total Ion Mass Chromatogram (ESI-ToF). The Y-axis is ion counts, and the X-axis is acquisition time in minutes.

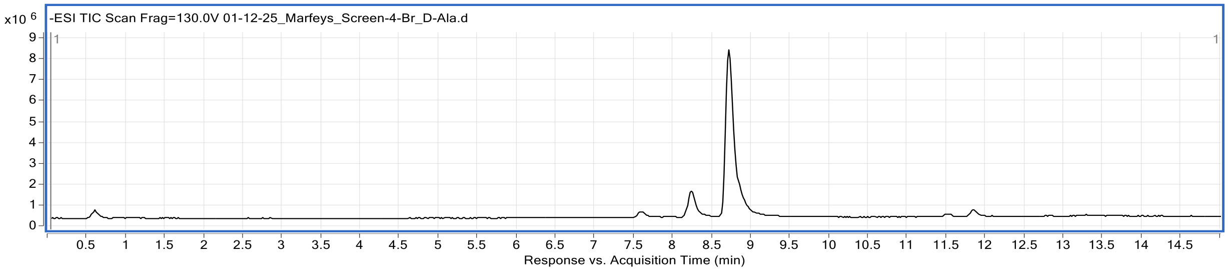

Variable Wavelength Detector Chromatogram (340 nm). The Y-axis is absorbance units, and the X-axis is acquisition time in minutes.

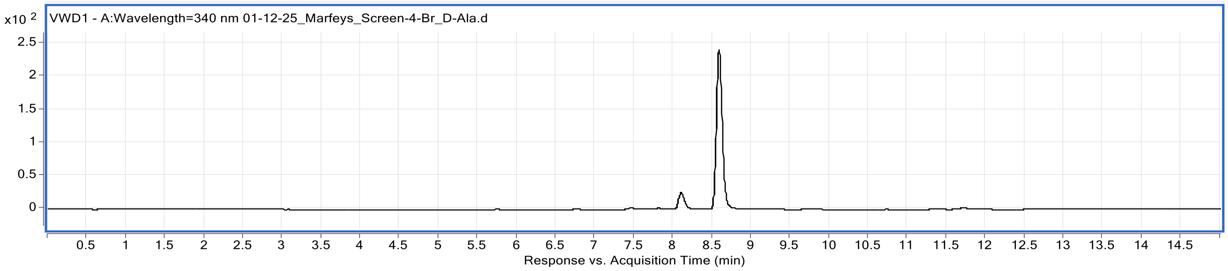

Extracted Ion Mass Chromatogram (ESI-ToF, extracted for m/z 510 ± 0.5). The Y-axis is ion counts, and the X-axis is acquisition time in minutes.

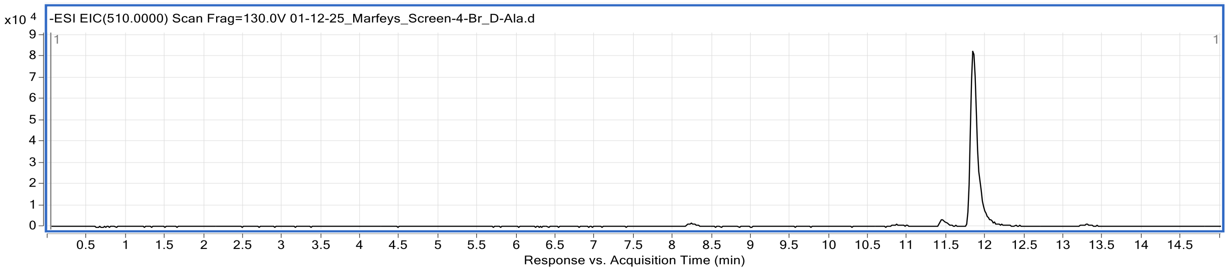

Zoomed and rescaled Extracted Ion (top) and Variable Wavelength Detector (bottom) Chromatograms. The Y axis on top is ion counts, the Y-axis on the bottom is absorbance units, and the X-axis for both is acquisition time in minutes. The peaks are labeled from left to right as Peak 1 and Peak 2.

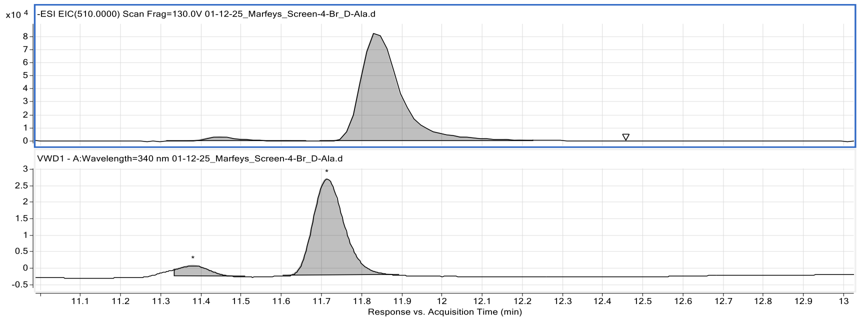

The following is a table of the peak data from the mass chromatogram. The Area % is the percent area relative to the tallest integrated peak, which has been set to 100%.

Peak	Assignment	t_R (min)	ES-ToF m/z [Neg]	Area %
1	L-*Erythro*	11.446	510.0268	3.88
2	L-*Threo*	11.828	510.0269	100

The calculated m/z for the adduct is 510.0266 for $C_{18}H_{17}BrN_5O_8^-$ $[M – H]^{-1}$.

The following is a table of the peak data from the variable wavelength detector chromatogram (340 nm). The Area % is the percent area relative to the tallest integrated peak, which has been set to 100%.

Peak	Assignment	t_R (min)	Area %
1	L-*Erythro*	11.340	3.74
2	L-*Threo*	11.713	100

The following is a table showing the difference in retention time on the extracted ion chromatogram (i.e., m/z Δt_R) and variable wavelength detector (i.e., VWD Δt_R) between the two diastereomers (i.e., separation = absolute difference between L-*erythro* and L-*threo*). The Average Δt_R values are the average difference in retention time between the extracted ion and variable wavelength detector chromatograms.

	m/z Δt_R (min)	VWD Δt_R (min)	Average Δt_R (min)
Peak Separation	0.382	0.373	0.38

LC/MS Traces for the Attempted Resolution of the Beta Stereoisomers of the β-hydroxy-α-Amino Acid Corresponding to Adduct 21 with 1-Fluoro-2,4-dinitrophenyl-5-L-proline Amide (i.e., L-FDPA 9) with HPLC Method B (i.e., 10 min.)

Total Ion Mass Chromatogram (ESI-ToF). The Y-axis is ion counts, and the X-axis is acquisition time in minutes.

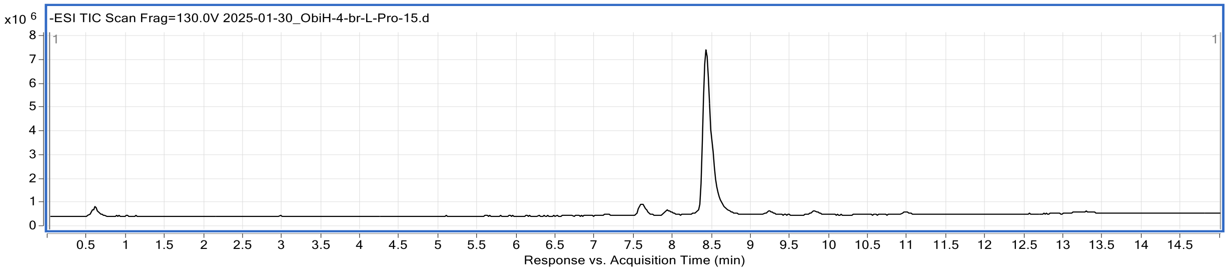

Variable Wavelength Detector Chromatogram (340 nm). The Y-axis is absorbance units, and the X-axis is acquisition time in minutes.

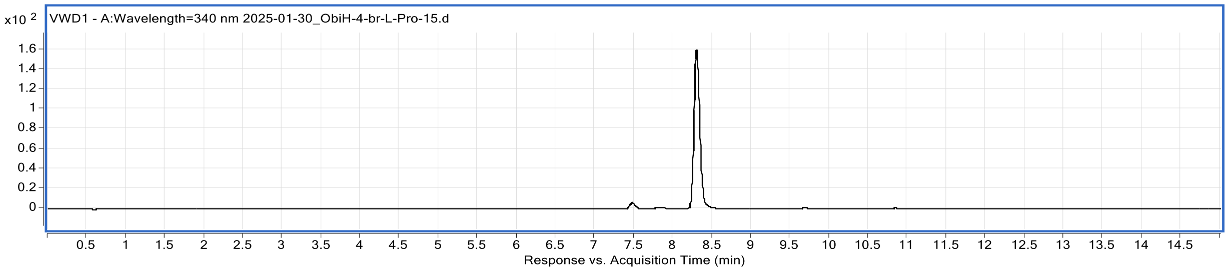

Extracted Ion Mass Chromatogram (ESI-ToF, extracted for m/z 536 ± 0.5). The Y-axis is ion counts, and the X-axis is acquisition time in minutes.

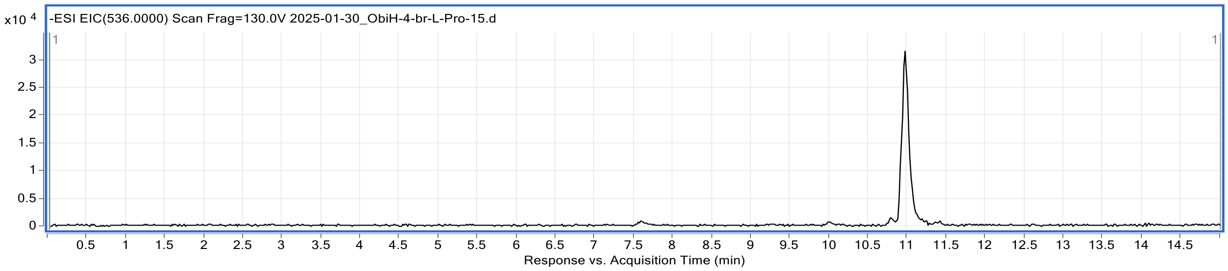

Zoomed and rescaled Extracted Ion (top) and Variable Wavelength Detector (bottom) Chromatograms. The Y axis on top is ion counts, the Y-axis on the bottom is absorbance units, and the X-axis for both is acquisition time in minutes. The peaks are labeled from left to right as Peak 1 and Peak 2.

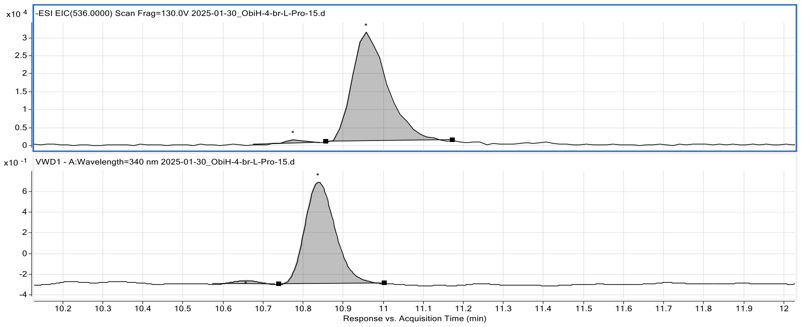

The following is a table of the peak data from the mass chromatogram. The Area % is the percent area relative to the tallest integrated peak, which has been set to 100%.

Peak	Assignment	t_R (min)	ES-ToF m/z [Neg]	Area %
1	L-*Erythro*	10.774	536.0429	2.02
2	L-*Threo*	10.957	536.0430	100

The calculated m/z for the adduct is 536.0422 for $C_{20}H_{19}BrN_5O_8^-$ $[M - H]^{-1}$.

The following is a table of the peak data from the variable wavelength detector chromatogram (340 nm). The Area % is the percent area relative to the tallest integrated peak, which has been set to 100%.

Peak	Assignment	t_R (min)	Area %
1	L-*Erythro*	10.657	3.3
2	L-*Threo*	10.837	100

The following is a table showing the difference in retention time on the extracted ion chromatogram (i.e., m/z Δt_R) and variable wavelength detector (i.e., VWD Δt_R) between the two diastereomers (i.e., separation = absolute difference between L-*erythro* and L-*threo*). The Average Δt_R values are the average difference in retention time between the extracted ion and variable wavelength detector chromatograms.

	m/z Δt_R (min)	VWD Δt_R (min)	Average Δt_R (min)
Peak Separation	0.183	0.180	0.18

LC/MS Traces for the Attempted Resolution of the Beta Stereoisomers of the β-hydroxy-α-Amino Acid Corresponding to Adduct 21 with 1-Fluoro-2,4-dinitrophenyl-5-D-proline Amide (i.e., D-FDPA D-9) with HPLC Method B (i.e., 10 min.)

Total Ion Mass Chromatogram (ESI-ToF). The Y-axis is ion counts, and the X-axis is acquisition time in minutes.

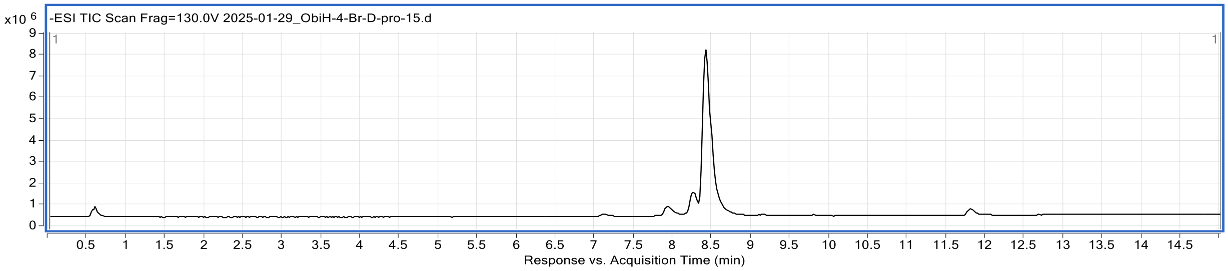

Variable Wavelength Detector Chromatogram (340 nm). The Y-axis is absorbance units, and the X-axis is acquisition time in minutes.

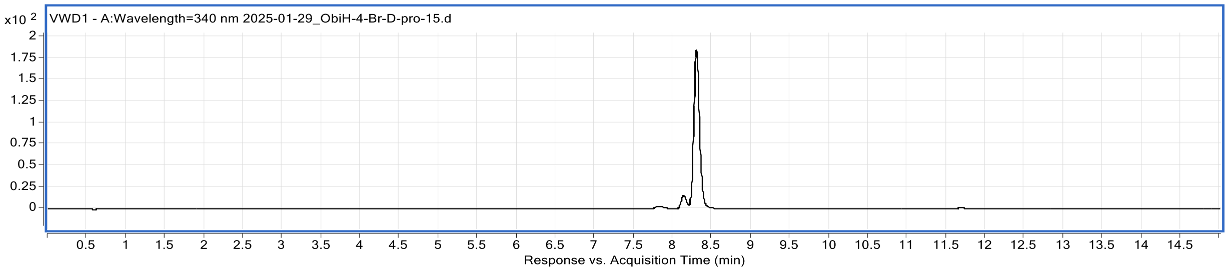

Extracted Ion Mass Chromatogram (ESI-ToF, extracted for m/z 536 ± 0.5). The Y-axis is ion counts, and the X-axis is acquisition time in minutes. Control experiments confirmed that the spurious peak at 10.4 min did not result from stereoisomer of the amino acid.

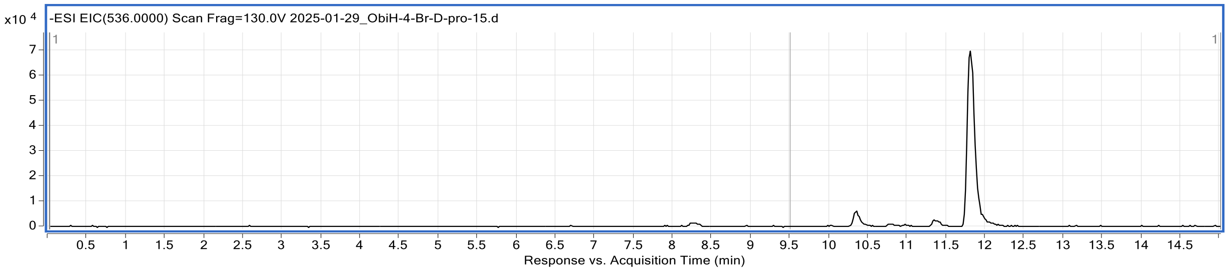

Zoomed and rescaled Extracted Ion (top) and Variable Wavelength Detector (bottom) Chromatograms. The Y axis on top is ion counts, the Y-axis on the bottom is absorbance units, and the X-axis for both is acquisition time in minutes. The peaks are labeled from left to right as Peak 1 and Peak 2.

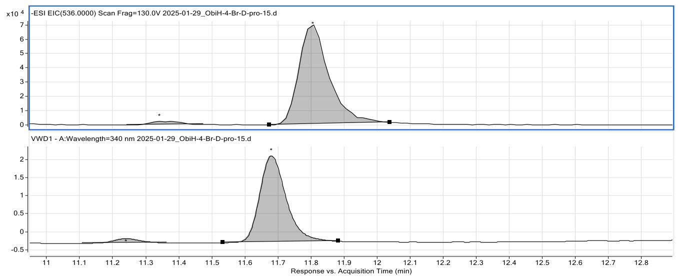

The following is a table of the peak data from the mass chromatogram. The Area % is the percent area relative to the tallest integrated peak, which has been set to 100%.

Peak	Assignment	t_R (min)	ES-ToF m/z [Neg]	Area %
1	L-*Erythro*	11.341	536.0415	3.04
2	L-*Threo*	11.806	536.0448	100

The calculated m/z for the adduct is 536.0422 for $C_{20}H_{19}BrN_5O_8^-$ $[M - H]^{-1}$.

The following is a table of the peak data from the variable wavelength detector chromatogram (340 nm). The Area % is the percent area relative to the tallest integrated peak, which has been set to 100%.

Peak	Assignment	t_R (min)	Area %
1	L-*Erythro*	11.240	5.1
2	L-*Threo*	11.680	100

The following is a table showing the difference in retention time on the extracted ion chromatogram (i.e., m/z Δt_R) and variable wavelength detector (i.e., VWD Δt_R) between the two diastereomers (i.e., separation = absolute difference between L-*erythro* and L-*threo*). The Average Δt_R values are the average difference in retention time between the extracted ion and variable wavelength detector chromatograms.

	m/z Δt_R (min)	VWD Δt_R (min)	Average Δt_R (min)
Peak Separation	0.465	0.440	0.45

LC/MS Traces for the Attempted Resolution of the Beta Stereoisomers of the β-hydroxy-α-Amino Acid Corresponding to Adduct 21 with 1-Fluoro-2,4-dinitrobenzene (i.e., Sanger's reagent 10) with HPLC Method B (i.e., 10 min.)

Total Ion Mass Chromatogram (ESI-ToF). The Y-axis is ion counts, and the X-axis is acquisition time in minutes.

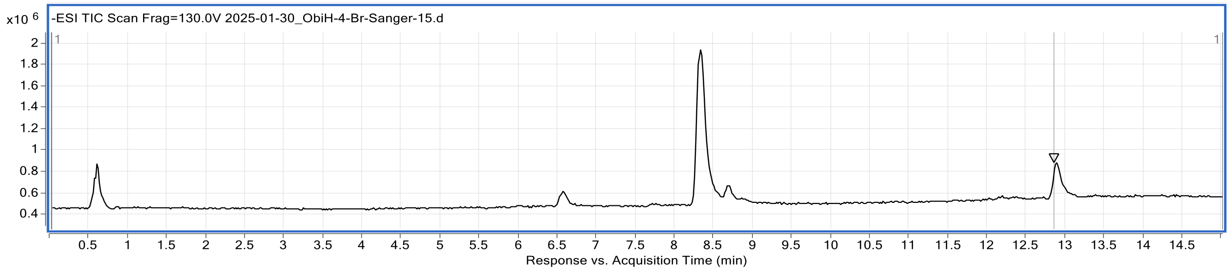

Variable Wavelength Detector Chromatogram (340 nm). The Y-axis is absorbance units, and the X-axis is acquisition time in minutes.

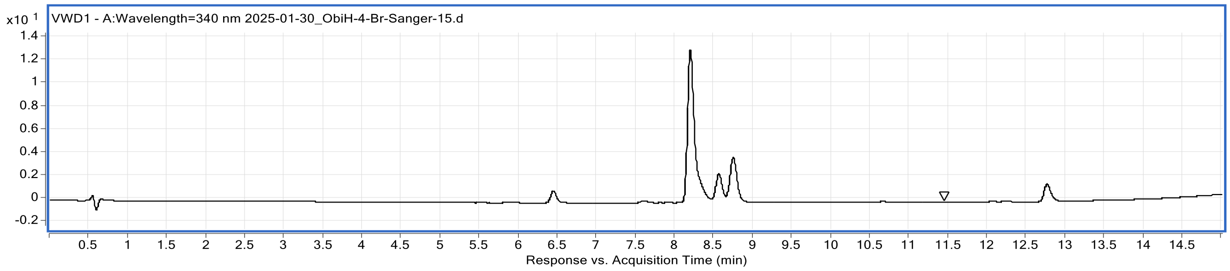

Extracted Ion Mass Chromatogram (ESI-ToF, extracted for m/z 424 ± 0.5). The Y-axis is ion counts, and the X-axis is acquisition time in minutes.

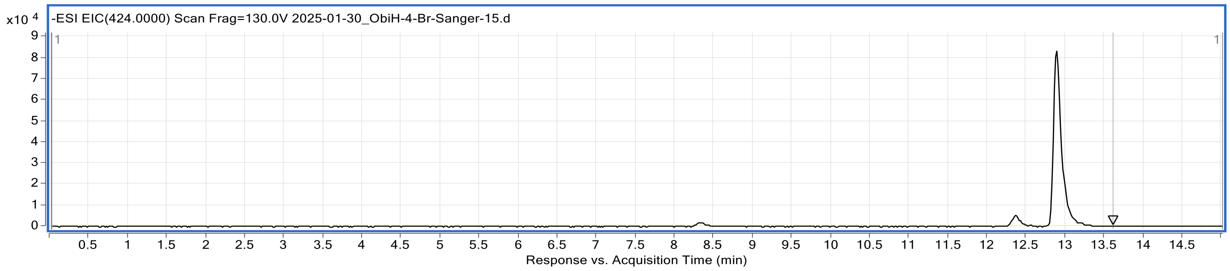

Zoomed and rescaled Extracted Ion (top) and Variable Wavelength Detector (bottom) Chromatograms. The Y axis on top is ion counts, the Y-axis on the bottom is absorbance units, and the X-axis for both is acquisition time in minutes. The peaks are labeled from left to right as Peak 1 and Peak 2.

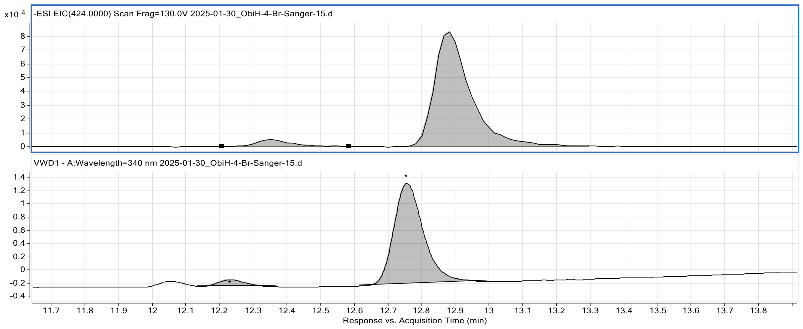

The following is a table of the peak data from the mass chromatogram. The Area % is the percent area relative to the tallest integrated peak, which has been set to 100%.

Peak	Assignment	t_R (min)	ES-ToF m/z [Neg]	Area %
1	L-*Erythro*	12.350	423.9762	6.17
2	L-*Threo*	12.881	423.9811	100

The calculated m/z for the adduct is 423.9786 for $C_{15}H_{11}BrN_3O_7^-$ $[M - H]^{-1}$.

The following is a table of the peak data from the variable wavelength detector chromatogram (340 nm). The Area % is the percent area relative to the tallest integrated peak, which has been set to 100%.

Peak	Assignment	t_R (min)	Area %
1	L-*Erythro*	12.230	5.83
2	L-*Threo*	12.753	100

The following is a table showing the difference in retention time on the extracted ion chromatogram (i.e., m/z Δt_R) and variable wavelength detector (i.e., VWD Δt_R) between the two diastereomers (i.e., separation = absolute difference between L-*erythro* and L-*threo*). The Average Δt_R values are the average difference in retention time between the extracted ion and variable wavelength detector chromatograms.

	m/z Δt_R (min)	VWD Δt_R (min)	Average Δt_R (min)
Peak Separation	0.531	0.523	0.53

LC/MS Traces for the Attempted Resolution of the Beta Stereoisomers of the β-hydroxy-α-Amino Acid Corresponding to Adduct 22 with 1-Fluoro-2,4-dinitrophenyl-5-L-alanine Amide (i.e., L-FDAA 7) with HPLC Method A (i.e., 25 min.)

Total Ion Mass Chromatogram (ESI-ToF). The Y-axis is ion counts, and the X-axis is acquisition time in minutes.

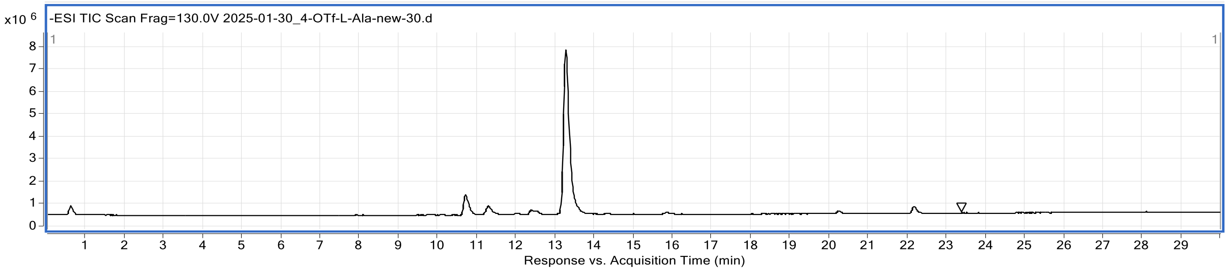

Variable Wavelength Detector Chromatogram (340 nm). The Y-axis is absorbance units, and the X-axis is acquisition time in minutes.

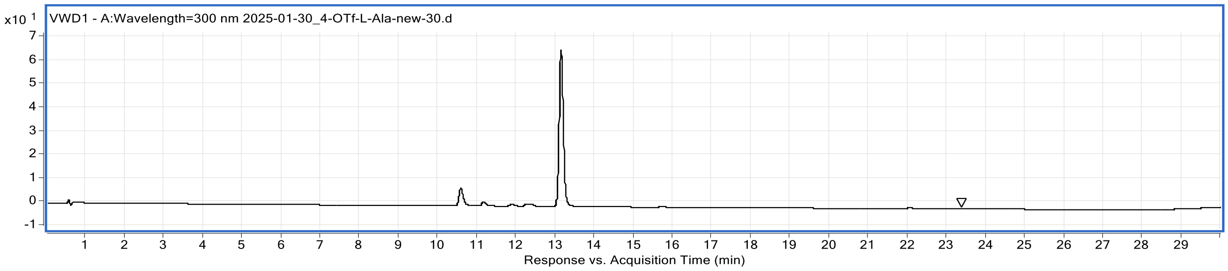

Extracted Ion Mass Chromatogram (ESI-ToF, extracted for m/z 580 ± 0.5). The Y-axis is ion counts, and the X-axis is acquisition time in minutes.

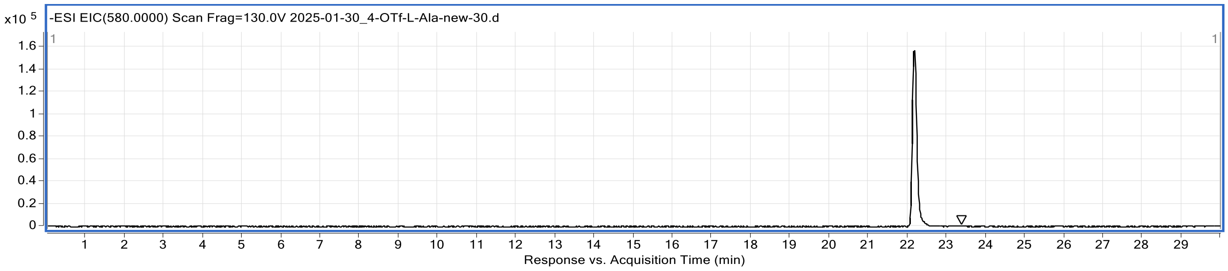

Zoomed and rescaled Extracted Ion (top) and Variable Wavelength Detector (bottom) Chromatograms. The Y axis on top is ion counts, the Y-axis on the bottom is absorbance units, and the X-axis for both is acquisition time in minutes. The peaks are labeled from left to right as Peak 1 and Peak 2.

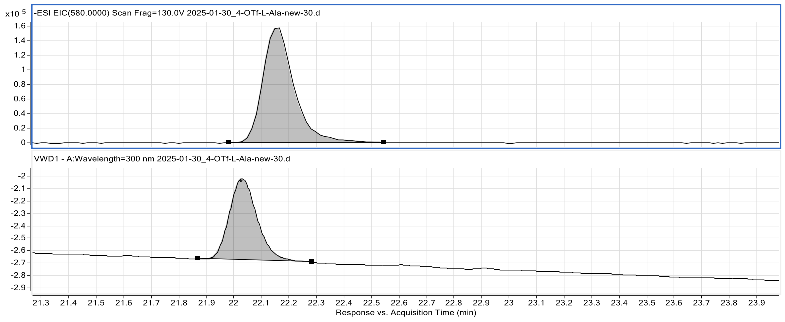

The following is a table of the peak data from the mass chromatogram. The Area % is the percent area relative to the tallest integrated peak, which has been set to 100%.

Peak	Assignment	t_R (min)	ES-ToF m/z [Neg]	Area %
1	L-*Erythro* and L-*Threo*	22.163	580.0594	100

The calculated m/z for the adduct is 580.0603 for $C_{19}H_{17}F_3N_5O_{11}S^-$ $[M - H]^{-1}$.

The following is a table of the peak data from the variable wavelength detector chromatogram (340 nm). The Area % is the percent area relative to the tallest integrated peak, which has been set to 100%.

Peak	Assignment	t_R (min)	Area %
1	L-*Erythro* and L-*Threo*	22.027	100

The following is a table showing the difference in retention time on the extracted ion chromatogram (i.e., m/z Δt_R) and variable wavelength detector (i.e., VWD Δt_R) between the two diastereomers (i.e., separation = absolute difference between L-*erythro* and L-*threo*). The Average Δt_R values are the average difference in retention time between the extracted ion and variable wavelength detector chromatograms.

	m/z Δt_R (min)	VWD Δt_R (min)	Average Δt_R (min)
Peak Separation	0	0	0

LC/MS Traces for the Attempted Resolution of the Beta Stereoisomers of the β-hydroxy-α-Amino Acid Corresponding to Adduct 22 with 1-Fluoro-2,4-dinitrophenyl-5-D-alanine Amide (i.e., D-FDAA D-7) with HPLC Method A (i.e., 25 min.)

Total Ion Mass Chromatogram (ESI-ToF). The Y-axis is ion counts, and the X-axis is acquisition time in minutes.

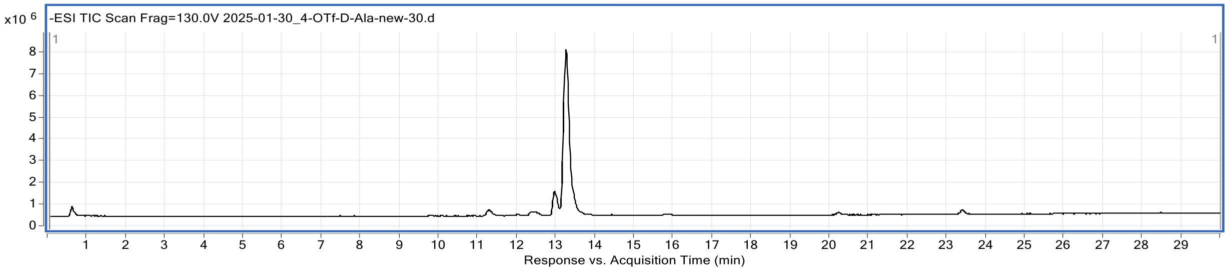

Variable Wavelength Detector Chromatogram (340 nm). The Y-axis is absorbance units, and the X-axis is acquisition time in minutes.

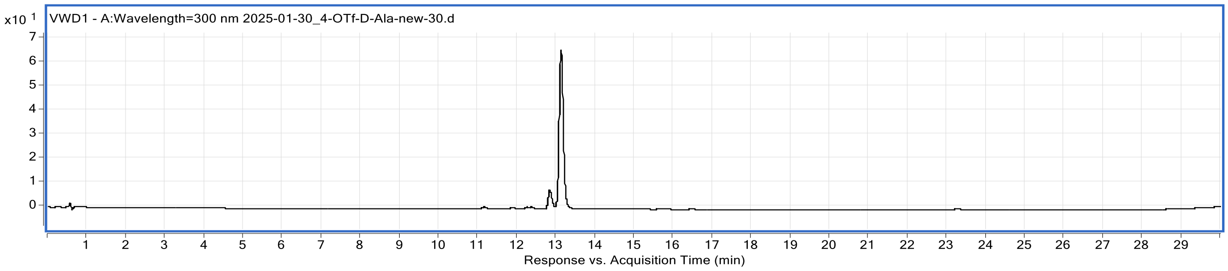

Extracted Ion Mass Chromatogram (ESI-ToF, extracted for m/z 580 ± 0.5). The Y-axis is ion counts, and the X-axis is acquisition time in minutes.

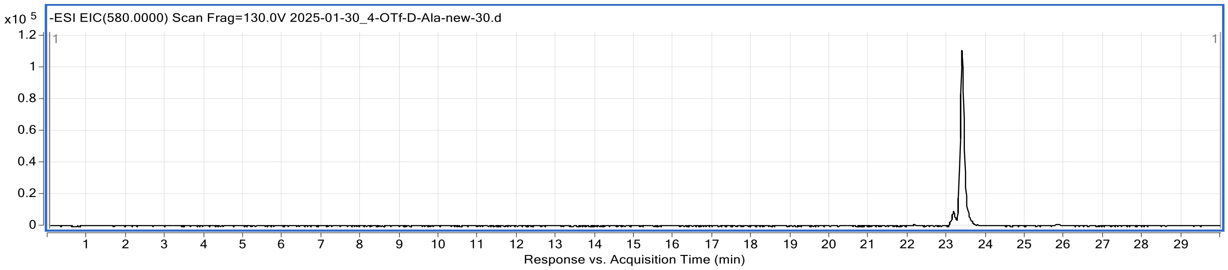

Zoomed and rescaled Extracted Ion (top) and Variable Wavelength Detector (bottom) Chromatograms. The Y axis on top is ion counts, the Y-axis on the bottom is absorbance units, and the X-axis for both is acquisition time in minutes. The peaks are labeled from left to right as Peak 1 and Peak 2.

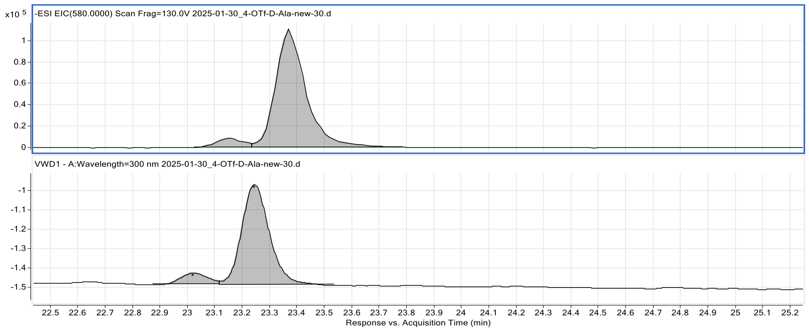

The following is a table of the peak data from the mass chromatogram. The Area % is the percent area relative to the tallest integrated peak, which has been set to 100%.

Peak	Assignment	t_R (min)	ES-ToF m/z [Neg]	Area %
1	L-*Erythro*	23.152	580.0606	7.06
2	L-*Threo*	23.368	580.0599	100

The calculated m/z for the adduct is 580.0603 for $C_{19}H_{17}F_3N_5O_{11}S^-$ $[M - H]^{-1}$.

The following is a table of the peak data from the variable wavelength detector chromatogram (340 nm). The Area % is the percent area relative to the tallest integrated peak, which has been set to 100%.

Peak	Assignment	t_R (min)	Area %
1	L-*Erythro*	23.02	11.36
2	L-*Threo*	23.243	100

The following is a table showing the difference in retention time on the extracted ion chromatogram (i.e., m/z Δt_R) and variable wavelength detector (i.e., VWD Δt_R) between the two diastereomers (i.e., separation = absolute difference between L-*erythro* and L-*threo*). The Average Δt_R values are the average difference in retention time between the extracted ion and variable wavelength detector chromatograms.

	m/z Δt_R (min)	VWD Δt_R (min)	Average Δt_R (min)
Peak Separation	0.216	0.223	0.22

LC/MS Traces for the Attempted Resolution of the Beta Stereoisomers of the β-hydroxy-α-Amino Acid Corresponding to Adduct 22 with 1-Fluoro-2,4-dinitrophenyl-5-L-proline Amide (i.e., L-FDPA 9) with HPLC Method A (i.e., 25 min.)

Total Ion Mass Chromatogram (ESI-ToF). The Y-axis is ion counts, and the X-axis is acquisition time in minutes.

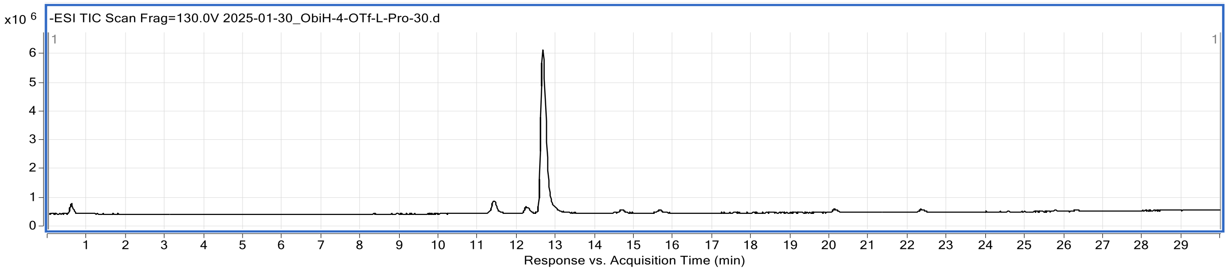

Variable Wavelength Detector Chromatogram (340 nm). The Y-axis is absorbance units, and the X-axis is acquisition time in minutes.

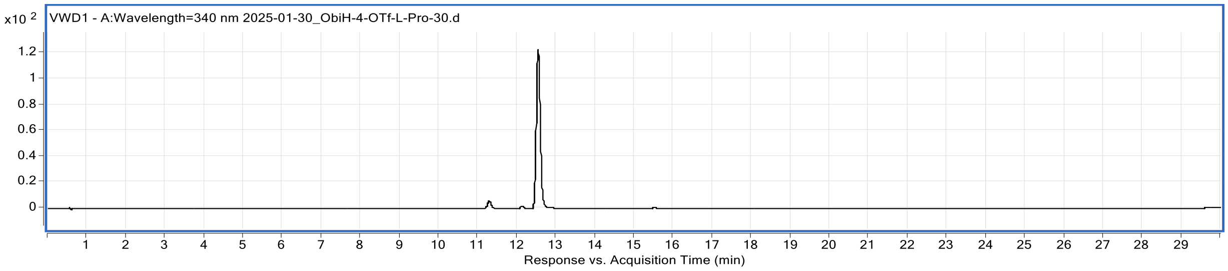

Extracted Ion Mass Chromatogram (ESI-ToF, extracted for m/z 606 ± 0.5). The Y-axis is ion counts, and the X-axis is acquisition time in minutes.

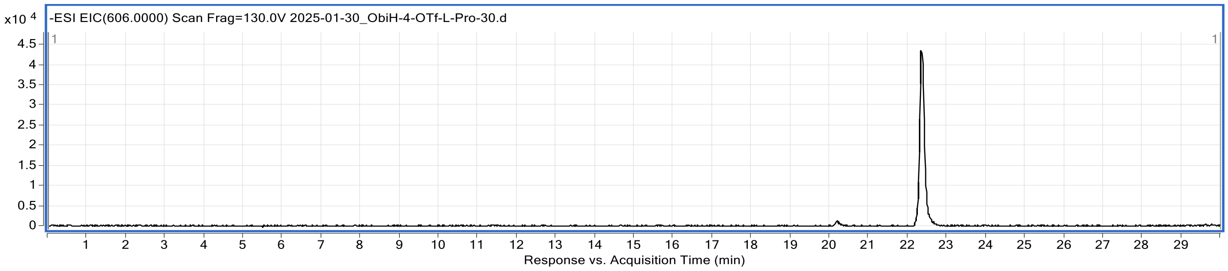

Zoomed and rescaled Extracted Ion (top) and Variable Wavelength Detector (bottom) Chromatograms. The Y axis on top is ion counts, the Y-axis on the bottom is absorbance units, and the X-axis for both is acquisition time in minutes. The peaks are labeled from left to right as Peak 1 and Peak 2.

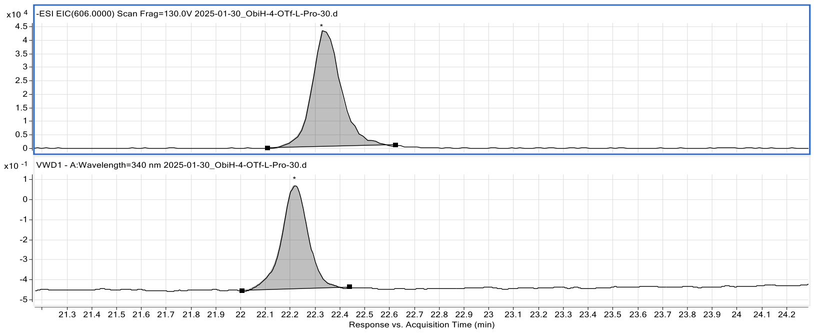

The following is a table of the peak data from the mass chromatogram. The Area % is the percent area relative to the tallest integrated peak, which has been set to 100%.

Peak	Assignment	t_R (min)	ES-ToF m/z [Neg]	Area %
1	L-*Erythro* and L-*Threo*	22.326	606.0772	100

The calculated m/z for the adduct is 606.0759 for $C_{19}H_{17}F_3N_5O_{11}S^-$ $[M - H]^{-1}$.

The following is a table of the peak data from the variable wavelength detector chromatogram (340 nm). The Area % is the percent area relative to the tallest integrated peak, which has been set to 100%.

Peak	Assignment	t_R (min)	Area %
1	L-*Erythro* and L-*Threo*	22.217	100

The following is a table showing the difference in retention time on the extracted ion chromatogram (i.e., m/z Δt_R) and variable wavelength detector (i.e., VWD Δt_R) between the two diastereomers (i.e., separation = absolute difference between L-*erythro* and L-*threo*). The Average Δt_R values are the average difference in retention time between the extracted ion and variable wavelength detector chromatograms.

	m/z Δt_R (min)	VWD Δt_R (min)	Average Δt_R (min)
Peak Separation	0	0	0

LC/MS Traces for the Attempted Resolution of the Beta Stereoisomers of the β-hydroxy-α-Amino Acid Corresponding to Adduct 22 with 1-Fluoro-2,4-dinitrophenyl-5-D-proline Amide (i.e., D-FDPA D-9) with HPLC Method A (i.e., 25 min.)

Total Ion Mass Chromatogram (ESI-ToF). The Y-axis is ion counts, and the X-axis is acquisition time in minutes.

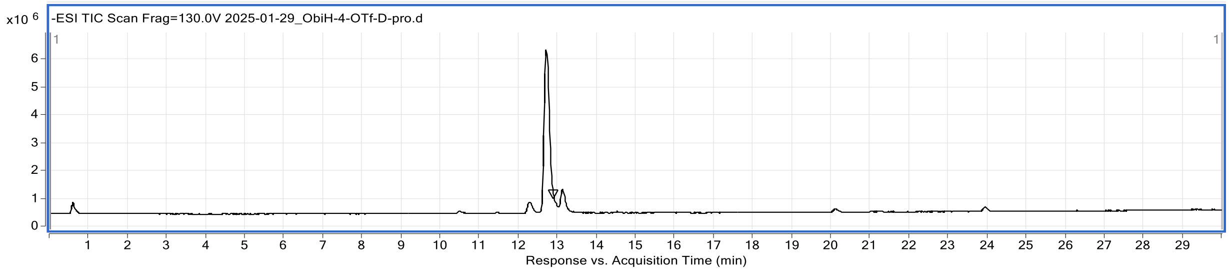

Variable Wavelength Detector Chromatogram (340 nm). The Y-axis is absorbance units, and the X-axis is acquisition time in minutes.

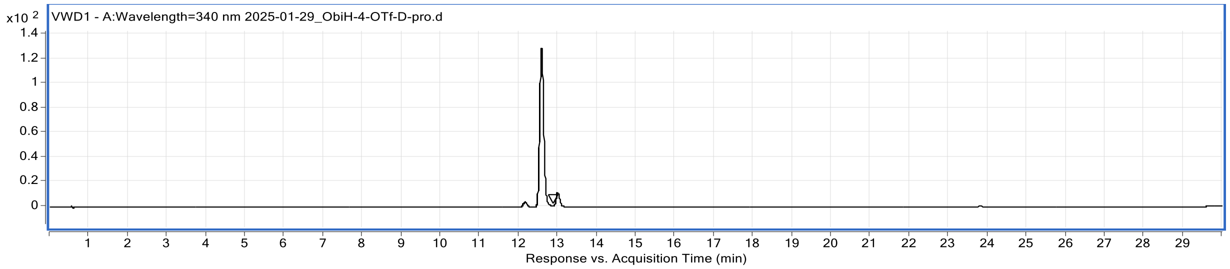

Extracted Ion Mass Chromatogram (ESI-ToF, extracted for m/z 606 ± 0.5). The Y-axis is ion counts, and the X-axis is acquisition time in minutes. Control experiments confirmed that the spurious peak at 21.2 min did not result from stereoisomer of the amino acid.

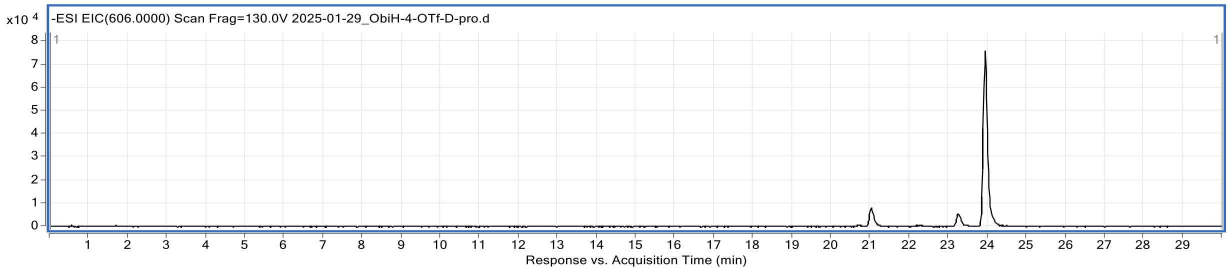

Zoomed and rescaled Extracted Ion (top) and Variable Wavelength Detector (bottom) Chromatograms. The Y axis on top is ion counts, the Y-axis on the bottom is absorbance units, and the X-axis for both is acquisition time in minutes. The peaks are labeled from left to right as Peak 1 and Peak 2.

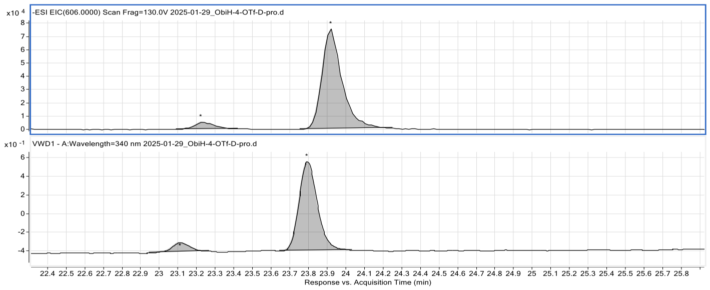

The following is a table of the peak data from the mass chromatogram. The Area % is the percent area relative to the tallest integrated peak, which has been set to 100%.

Peak	Assignment	t_R (min)	ES-ToF m/z [Neg]	Area %
1	L-*Erythro*	23.221	606.0763	6.6
2	L-*Threo*	23.919	606.0771	100

The calculated m/z for the adduct is 606.0759 for $C_{19}H_{17}F_3N_5O_{11}S^-$ $[M - H]^{-1}$.

The following is a table of the peak data from the variable wavelength detector chromatogram (340 nm). The Area % is the percent area relative to the tallest integrated peak, which has been set to 100%.

Peak	Assignment	t_R (min)	Area %
1	L-*Erythro*	23.110	10.21
2	L-*Threo*	23.790	100

The following is a table showing the difference in retention time on the extracted ion chromatogram (i.e., m/z Δt_R) and variable wavelength detector (i.e., VWD Δt_R) between the two diastereomers (i.e., separation = absolute difference between L-*erythro* and L-*threo*). The Average Δt_R values are the average difference in retention time between the extracted ion and variable wavelength detector chromatograms.

	m/z Δt_R (min)	VWD Δt_R (min)	Average Δt_R (min)
Peak Separation	0.698	0.680	0.69

LC/MS Traces for the Attempted Resolution of the Beta Stereoisomers of the β-hydroxy-α-Amino Acid Corresponding to Adduct 22 with 1-Fluoro-2,4-dinitrobenzene (i.e., Sanger's reagent 10) with HPLC Method A (i.e., 25 min.)

Total Ion Mass Chromatogram (ESI-ToF). The Y-axis is ion counts, and the X-axis is acquisition time in minutes.

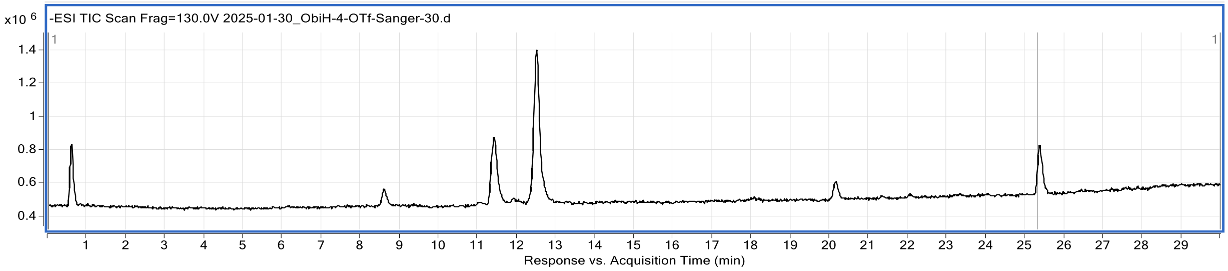

Variable Wavelength Detector Chromatogram (340 nm). The Y-axis is absorbance units, and the X-axis is acquisition time in minutes.

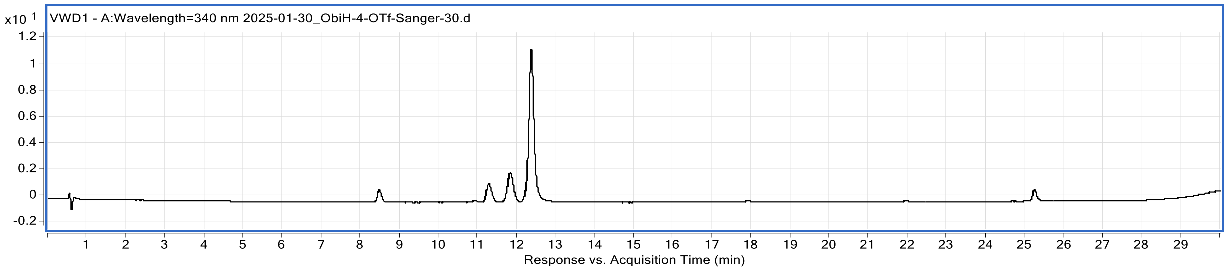

Extracted Ion Mass Chromatogram (ESI-ToF, extracted for m/z 494 ± 0.5). The Y-axis is ion counts, and the X-axis is acquisition time in minutes.

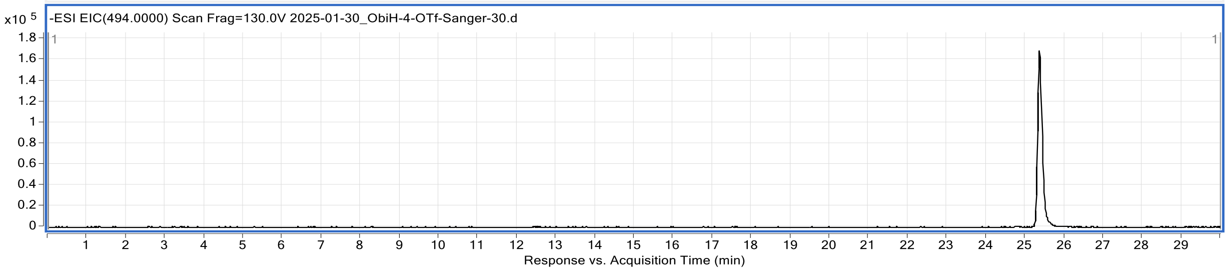

Zoomed and rescaled Extracted Ion (top) and Variable Wavelength Detector (bottom) Chromatograms. The Y axis on top is ion counts, the Y-axis on the bottom is absorbance units, and the X-axis for both is acquisition time in minutes. The peaks are labeled from left to right as Peak 1 and Peak 2.

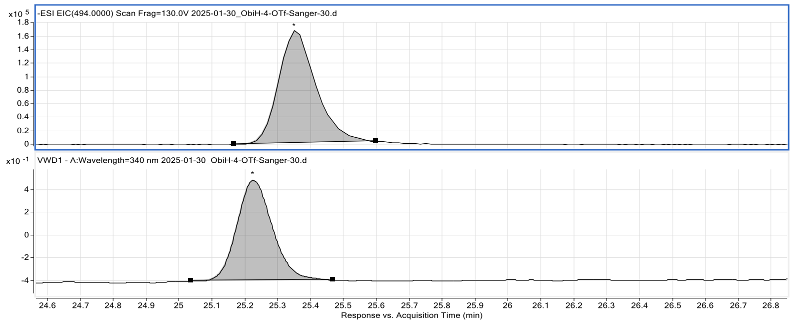

The following is a table of the peak data from the mass chromatogram. The Area % is the percent area relative to the tallest integrated peak, which has been set to 100%.

Peak	Assignment	t_R (min)	ES-ToF m/z [Neg]	Area %
1	L-*Erythro* and L-*Threo*	25.349	494.0151	100

The calculated m/z for the adduct is 494.0123 for $C_{16}H_{11}F_3N_3O_{10}S^-$ $[M – H]^{-1}$.

The following is a table of the peak data from the variable wavelength detector chromatogram (340 nm). The Area % is the percent area relative to the tallest integrated peak, which has been set to 100%.

Peak	Assignment	t_R (min)	Area %
1	L-*Erythro* and L-*Threo*	25.223	100

The following is a table showing the difference in retention time on the extracted ion chromatogram (i.e., m/z Δt_R) and variable wavelength detector (i.e., VWD Δt_R) between the two diastereomers (i.e., separation = absolute difference between L-*erythro* and L-*threo*). The Average Δt_R values are the average difference in retention time between the extracted ion and variable wavelength detector chromatograms.

	m/z Δt_R (min)	VWD Δt_R (min)	Average Δt_R (min)
Peak Separation	0	0	0

LC/MS Traces for the Attempted Resolution of the Beta Stereoisomers of the β-hydroxy-α-Amino Acid Corresponding to Adduct 23 with 1-Fluoro-2,4-dinitrophenyl-5-L-alanine Amide (i.e., L-FDAA 7) with HPLC Method B (i.e., 10 min.)

Total Ion Mass Chromatogram (ESI-ToF). The Y-axis is ion counts, and the X-axis is acquisition time in minutes.

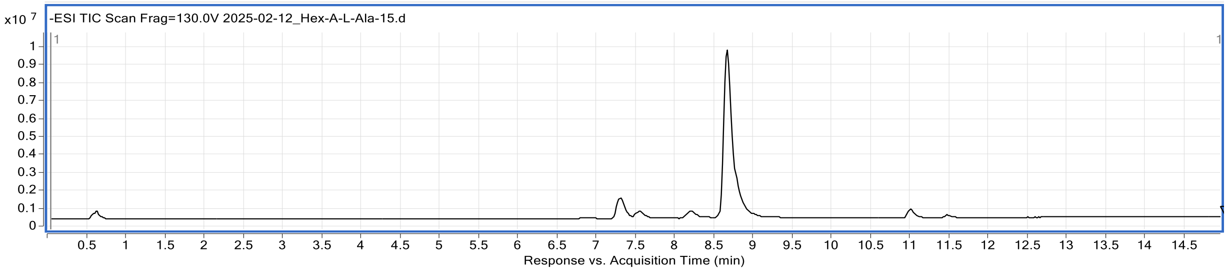

Variable Wavelength Detector Chromatogram (340 nm). The Y-axis is absorbance units, and the X-axis is acquisition time in minutes.

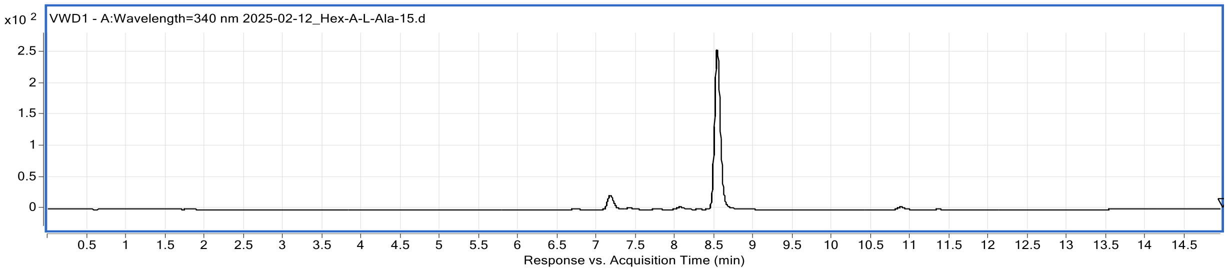

Extracted Ion Mass Chromatogram (ESI-ToF, extracted for m/z 426 ± 0.5). The Y-axis is ion counts, and the X-axis is acquisition time in minutes.

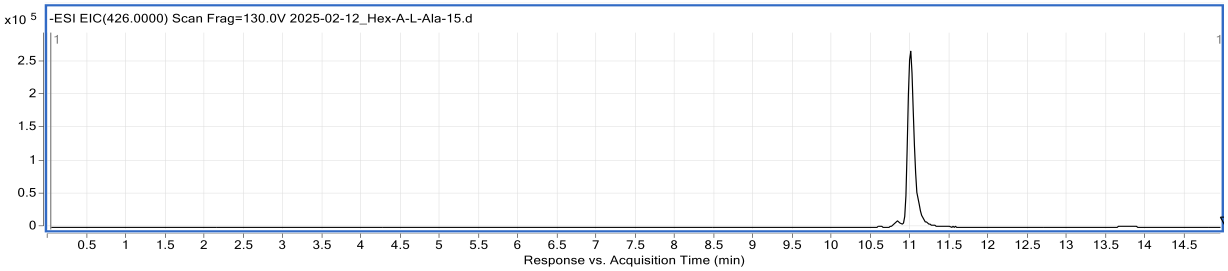

Zoomed and rescaled Extracted Ion (top) and Variable Wavelength Detector (bottom) Chromatograms. The Y axis on top is ion counts, the Y-axis on the bottom is absorbance units, and the X-axis for both is acquisition time in minutes. The peaks are labeled from left to right as Peak 1 and Peak 2.

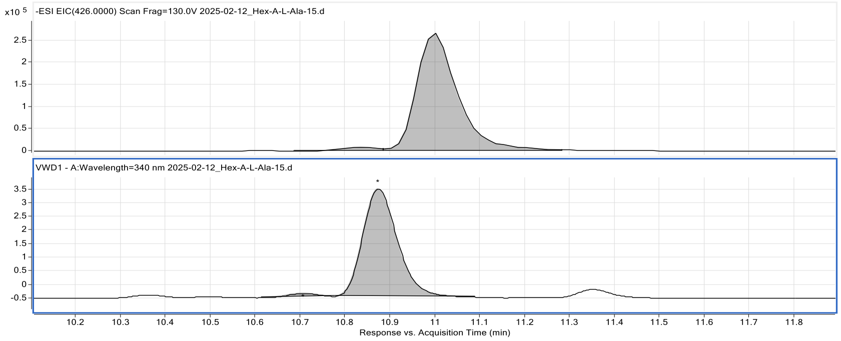

The following is a table of the peak data from the mass chromatogram. The Area % is the percent area relative to the tallest integrated peak, which has been set to 100%.

Peak	Assignment	t_R (min)	ES-ToF m/z [Neg]	Area %
1	L-*Erythro*	10.836	426.1657	2.76
2	L-*Threo*	11.002	426.1660	100

The calculated m/z for the adduct is 426.1630 for $C_{17}H_{24}N_5O_8^-$ $[M - H]^{-1}$.

The following is a table of the peak data from the variable wavelength detector chromatogram (340 nm). The Area % is the percent area relative to the tallest integrated peak, which has been set to 100%.

Peak	Assignment	t_R (min)	Area %
1	L-*Erythro*	10.707	2.72
2	L-*Threo*	10.873	100

The following is a table showing the difference in retention time on the extracted ion chromatogram (i.e., m/z Δt_R) and variable wavelength detector (i.e., VWD Δt_R) between the two diastereomers (i.e., separation = absolute difference between L-*erythro* and L-*threo*). The Average Δt_R values are the average difference in retention time between the extracted ion and variable wavelength detector chromatograms.

	m/z Δt_R (min)	VWD Δt_R (min)	Average Δt_R (min)
Peak Separation	0.166	0.166	0.17

LC/MS Traces for the Attempted Resolution of the Beta Stereoisomers of the β-hydroxy-α-Amino Acid Corresponding to Adduct 23 with 1-Fluoro-2,4-dinitrophenyl-5-D-alanine Amide (i.e., D-FDAA D-7) with HPLC Method B (i.e., 10 min.)

Total Ion Mass Chromatogram (ESI-ToF). The Y-axis is ion counts, and the X-axis is acquisition time in minutes.

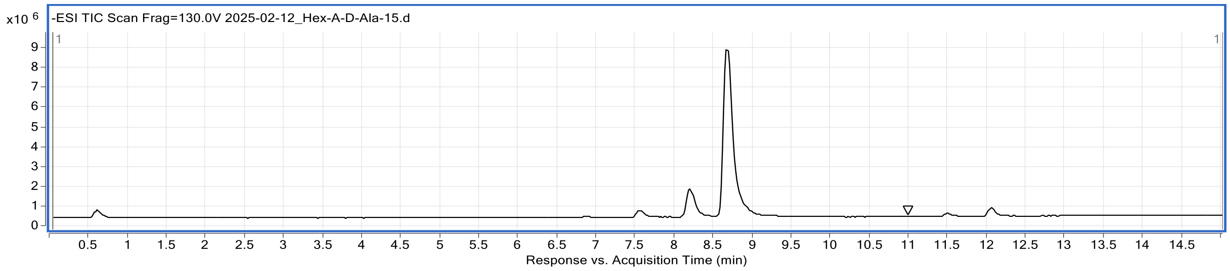

Variable Wavelength Detector Chromatogram (340 nm). The Y-axis is absorbance units, and the X-axis is acquisition time in minutes.

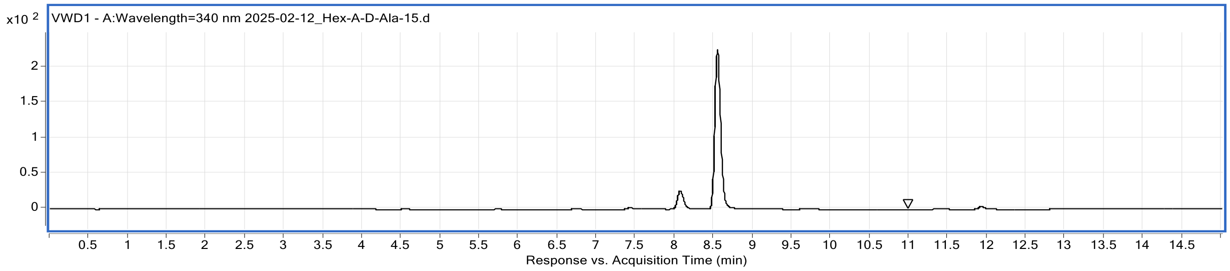

Extracted Ion Mass Chromatogram (ESI-ToF, extracted for m/z 426 ± 0.5). The Y-axis is ion counts, and the X-axis is acquisition time in minutes.

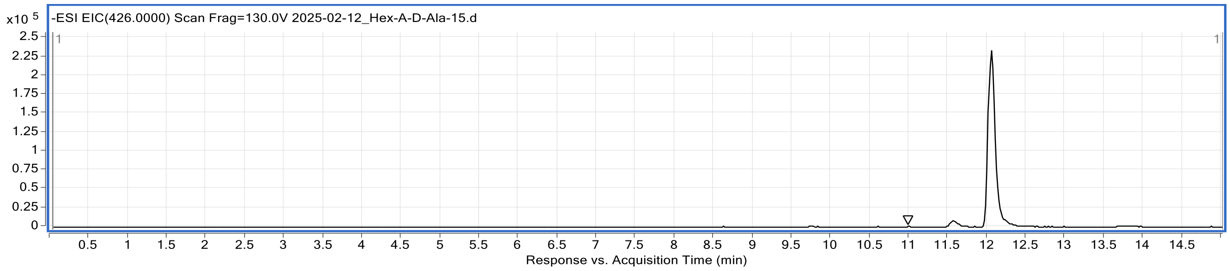

Zoomed and rescaled Extracted Ion (top) and Variable Wavelength Detector (bottom) Chromatograms. The Y axis on top is ion counts, the Y-axis on the bottom is absorbance units, and the X-axis for both is acquisition time in minutes. The peaks are labeled from left to right as Peak 1 and Peak 2.

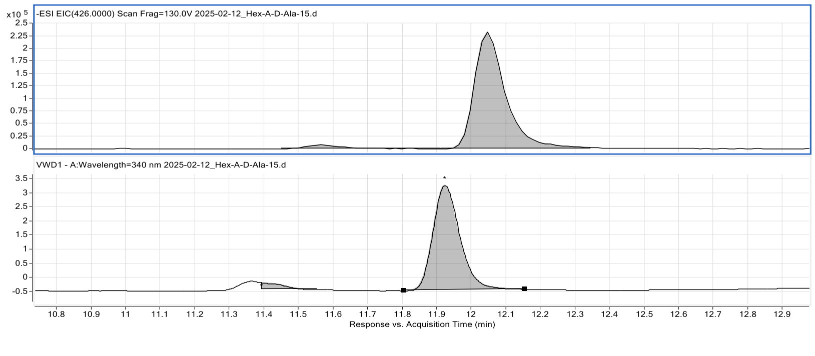

The following is a table of the peak data from the mass chromatogram. The Area % is the percent area relative to the tallest integrated peak, which has been set to 100%.

Peak	Assignment	t_R (min)	ES-ToF m/z [Neg]	Area %
1	L-*Erythro*	11.563	426.1638	3.69
2	L-*Threo*	12.045	426.4656	100

The calculated m/z for the adduct is 426.1630 for $C_{17}H_{24}N_5O_8^-$ $[M - H]^{-1}$.

The following is a table of the peak data from the variable wavelength detector chromatogram (340 nm). The Area % is the percent area relative to the tallest integrated peak, which has been set to 100%.

Peak	Assignment	t_R (min)	Area %
1	L-*Erythro*	11.393	4.76
2	L-*Threo*	11.923	100

The following is a table showing the difference in retention time on the extracted ion chromatogram (i.e., m/z Δt_R) and variable wavelength detector (i.e., VWD Δt_R) between the two diastereomers (i.e., separation = absolute difference between L-*erythro* and L-*threo*). The Average Δt_R values are the average difference in retention time between the extracted ion and variable wavelength detector chromatograms.

	m/z Δt_R (min)	VWD Δt_R (min)	Average Δt_R (min)
Peak Separation	0.482	0.530	0.51

LC/MS Traces for the Attempted Resolution of the Beta Stereoisomers of the β-hydroxy-α-Amino Acid Corresponding to Adduct 23 with 1-Fluoro-2,4-dinitrophenyl-5-L-proline Amide (i.e., L-FDPA 9) with HPLC Method B (i.e., 10 min.)

Total Ion Mass Chromatogram (ESI-ToF). The Y-axis is ion counts, and the X-axis is acquisition time in minutes.

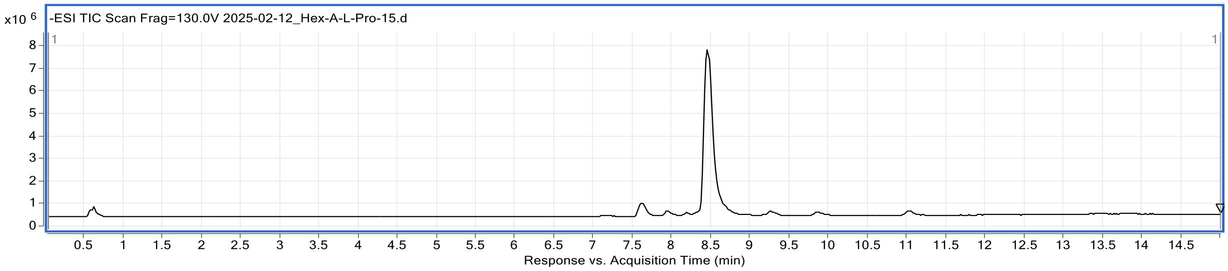

Variable Wavelength Detector Chromatogram (340 nm). The Y-axis is absorbance units, and the X-axis is acquisition time in minutes.

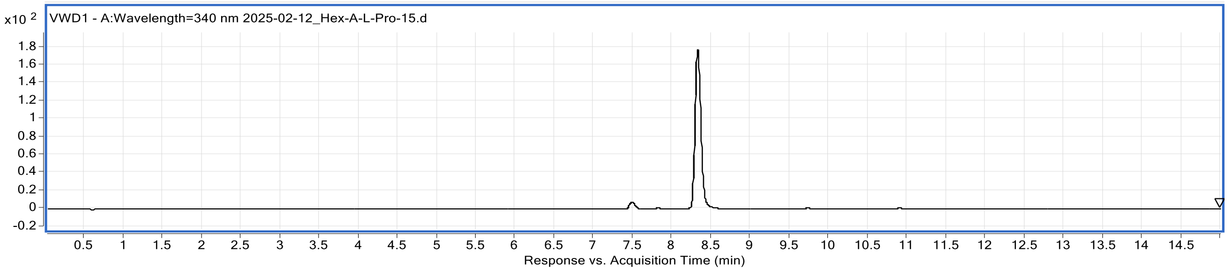

Extracted Ion Mass Chromatogram (ESI-ToF, extracted for m/z 452 ± 0.5). The Y-axis is ion counts, and the X-axis is acquisition time in minutes.

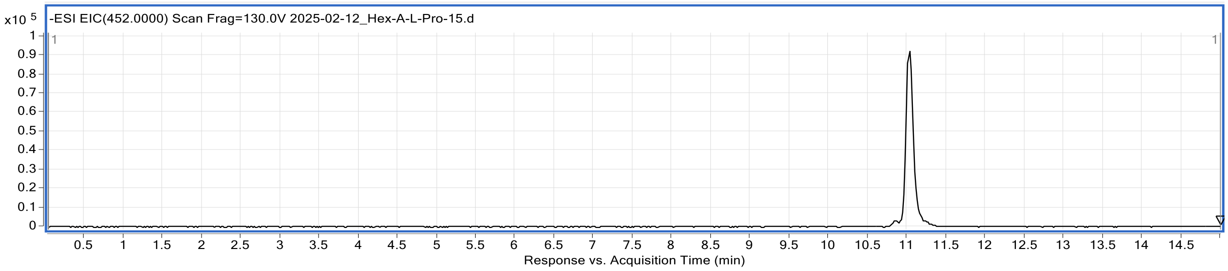

Zoomed and rescaled Extracted Ion (top) and Variable Wavelength Detector (bottom) Chromatograms. The Y axis on top is ion counts, the Y-axis on the bottom is absorbance units, and the X-axis for both is acquisition time in minutes. The peaks are labeled from left to right as Peak 1 and Peak 2.

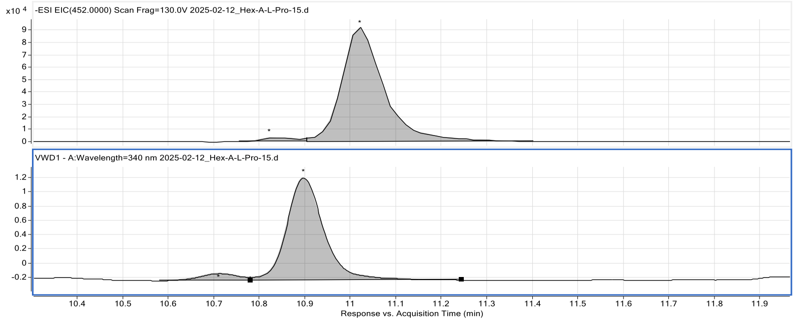

The following is a table of the peak data from the mass chromatogram. The Area % is the percent area relative to the tallest integrated peak, which has been set to 100%.

Peak	Assignment	t_R (min)	ES-ToF m/z [Neg]	Area %
1	L-*Erythro*	10.838	452.1768	2.80
2	L-*Threo*	11.021	452.1799	100

The calculated m/z for the adduct is 452.1787 for $C_{19}H_{26}N_5O_8^-$ $[M - H]^{-1}$.

The following is a table of the peak data from the variable wavelength detector chromatogram (340 nm). The Area % is the percent area relative to the tallest integrated peak, which has been set to 100%.

Peak	Assignment	t_R (min)	Area %
1	L-*Erythro*	10.710	7.79
2	L-*Threo*	10.897	100

The following is a table showing the difference in retention time on the extracted ion chromatogram (i.e., m/z Δt_R) and variable wavelength detector (i.e., VWD Δt_R) between the two diastereomers (i.e., separation = absolute difference between L-*erythro* and L-*threo*). The Average Δt_R values are the average difference in retention time between the extracted ion and variable wavelength detector chromatograms.

	m/z Δt_R (min)	VWD Δt_R (min)	Average Δt_R (min)
Peak Separation	0.183	0.187	0.19

LC/MS Traces for the Attempted Resolution of the Beta Stereoisomers of the β-hydroxy-α-Amino Acid Corresponding to Adduct 23 with 1-Fluoro-2,4-dinitrophenyl-5-D-proline Amide (i.e., D-FDPA D-9) with HPLC Method B (i.e., 10 min.)

Total Ion Mass Chromatogram (ESI-ToF). The Y-axis is ion counts, and the X-axis is acquisition time in minutes.

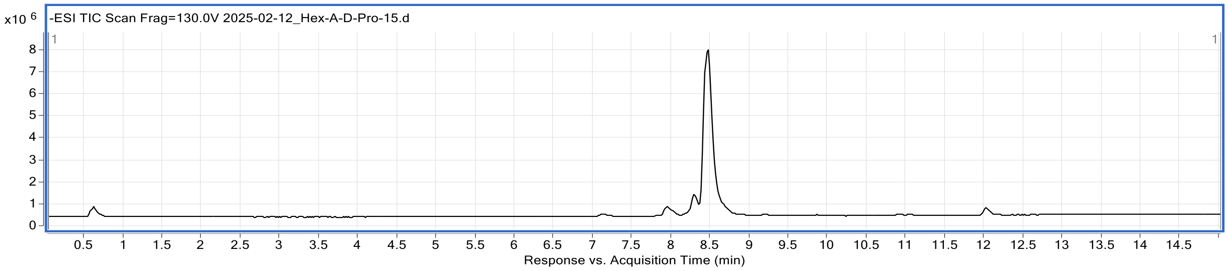

Variable Wavelength Detector Chromatogram (340 nm). The Y-axis is absorbance units, and the X-axis is acquisition time in minutes.

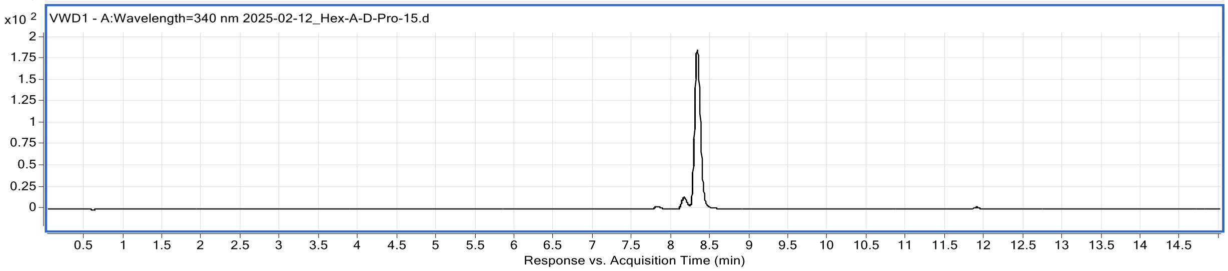

Extracted Ion Mass Chromatogram (ESI-ToF, extracted for m/z 452 ± 0.5). The Y-axis is ion counts, and the X-axis is acquisition time in minutes. Control experiments confirmed that the spurious peak at 10.9 min did not result from stereoisomer of the amino acid.

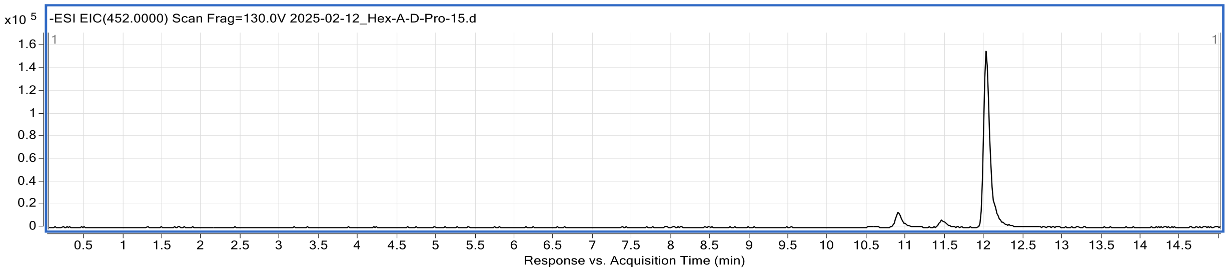

Zoomed and rescaled Extracted Ion (top) and Variable Wavelength Detector (bottom) Chromatograms. The Y axis on top is ion counts, the Y-axis on the bottom is absorbance units, and the X-axis for both is acquisition time in minutes. The peaks are labeled from left to right as Peak 1 and Peak 2.

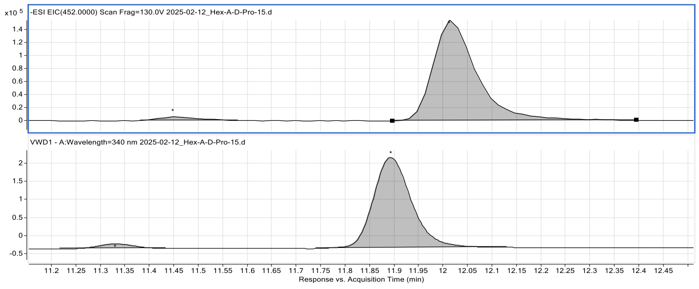

The following is a table of the peak data from the mass chromatogram. The Area % is the percent area relative to the tallest integrated peak, which has been set to 100%.

Peak	Assignment	t_R (min)	ES-ToF m/z [Neg]	Area %
1	L-*Erythro*	11.448	452.1770	3.33
2	L-*Threo*	12.012	452.1776	100

The calculated m/z for the adduct is 452.1787 for $C_{19}H_{26}N_5O_8^-$ $[M - H]^{-1}$.

The following is a table of the peak data from the variable wavelength detector chromatogram (340 nm). The Area % is the percent area relative to the tallest integrated peak, which has been set to 100%.

Peak	Assignment	t_R (min)	Area %
1	L-*Erythro*	11.33	4.72
2	L-*Threo*	11.893	100

The following is a table showing the difference in retention time on the extracted ion chromatogram (i.e., m/z Δt_R) and variable wavelength detector (i.e., VWD Δt_R) between the two diastereomers (i.e., separation = absolute difference between L-*erythro* and L-*threo*). The Average Δt_R values are the average difference in retention time between the extracted ion and variable wavelength detector chromatograms.

	m/z Δt_R (min)	VWD Δt_R (min)	Average Δt_R (min)
Peak Separation	0.564	0.563	0.56

LC/MS Traces for the Attempted Resolution of the Beta Stereoisomers of the β-hydroxy-α-Amino Acid Corresponding to Adduct 23 with 1-Fluoro-2,4-dinitrobenzene (i.e., Sanger's reagent 10) with HPLC Method B (i.e., 10 min.)

Total Ion Mass Chromatogram (ESI-ToF). The Y-axis is ion counts, and the X-axis is acquisition time in minutes.

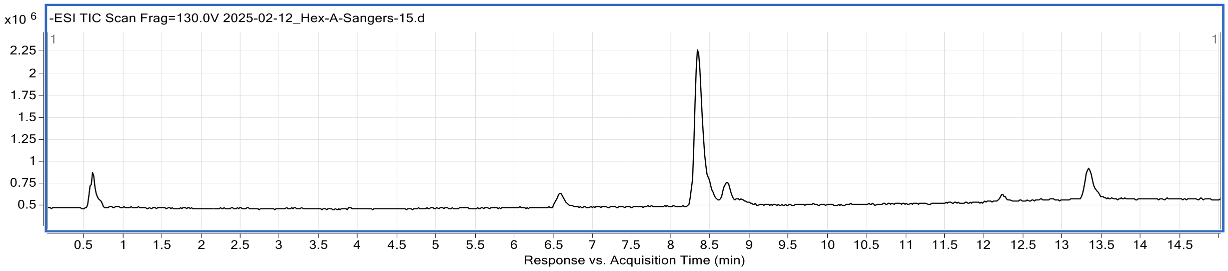

Variable Wavelength Detector Chromatogram (340 nm). The Y-axis is absorbance units, and the X-axis is acquisition time in minutes.

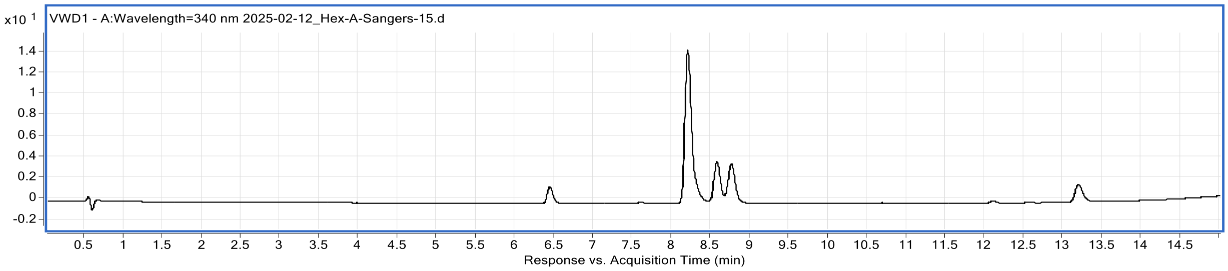

Extracted Ion Mass Chromatogram (ESI-ToF, extracted for m/z 340 ± 0.5). The Y-axis is ion counts, and the X-axis is acquisition time in minutes.

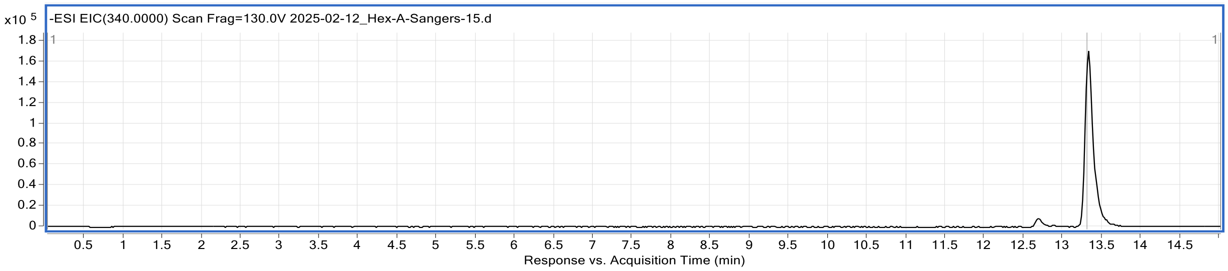

Zoomed and rescaled Extracted Ion (top) and Variable Wavelength Detector (bottom) Chromatograms. The Y axis on top is ion counts, the Y-axis on the bottom is absorbance units, and the X-axis for both is acquisition time in minutes. The peaks are labeled from left to right as Peak 1 and Peak 2.

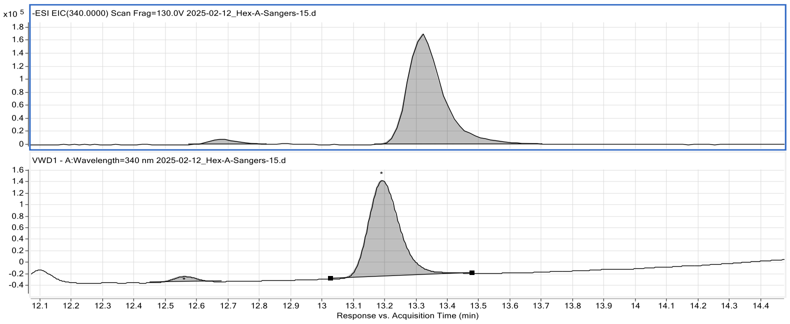

The following is a table of the peak data from the mass chromatogram. The Area % is the percent area relative to the tallest integrated peak, which has been set to 100%.

Peak	Assignment	t_R (min)	ES-ToF m/z [Neg]	Area %
1	L-*Erythro*	12.689	340.1143	4.04
2	L-*Threo*	13.320	340.1150	100

The calculated m/z for the adduct is 340.1150 for $C_{14}H_{18}N_3O_7^-$ $[M - H]^{-1}$.

The following is a table of the peak data from the variable wavelength detector chromatogram (340 nm). The Area % is the percent area relative to the tallest integrated peak, which has been set to 100%.

Peak	Assignment	t_R (min)	Area %
1	L-*Erythro*	12.560	4.43
2	L-*Threo*	13.190	100

The following is a table showing the difference in retention time on the extracted ion chromatogram (i.e., m/z Δt_R) and variable wavelength detector (i.e., VWD Δt_R) between the two diastereomers (i.e., separation = absolute difference between L-*erythro* and L-*threo*). The Average Δt_R values are the average difference in retention time between the extracted ion and variable wavelength detector chromatograms.

	m/z Δt_R (min)	VWD Δt_R (min)	Average Δt_R (min)
Peak Separation	0.631	0.630	0.63

LC/MS Traces for the Attempted Resolution of the Beta Stereoisomers of the β-hydroxy-α-Amino Acid Corresponding to Adduct 24 with 1-Fluoro-2,4-dinitrophenyl-5-L-alanine Amide (i.e., L-FDAA 7) with HPLC Method B (i.e., 10 min.)

Total Ion Mass Chromatogram (ESI-ToF). The Y-axis is ion counts, and the X-axis is acquisition time in minutes.

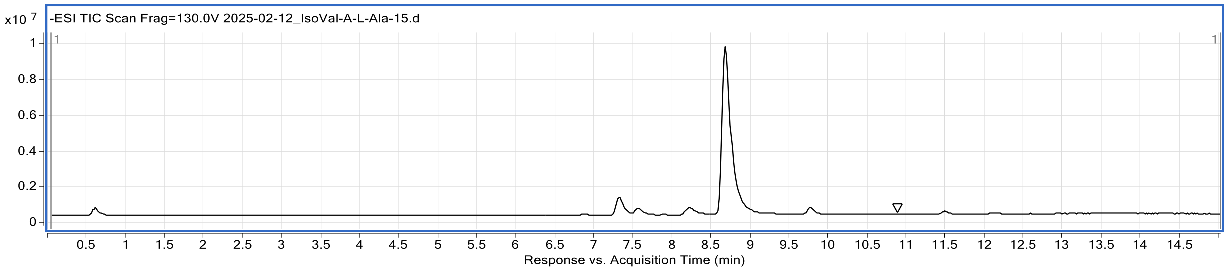

Variable Wavelength Detector Chromatogram (340 nm). The Y-axis is absorbance units, and the X-axis is acquisition time in minutes.

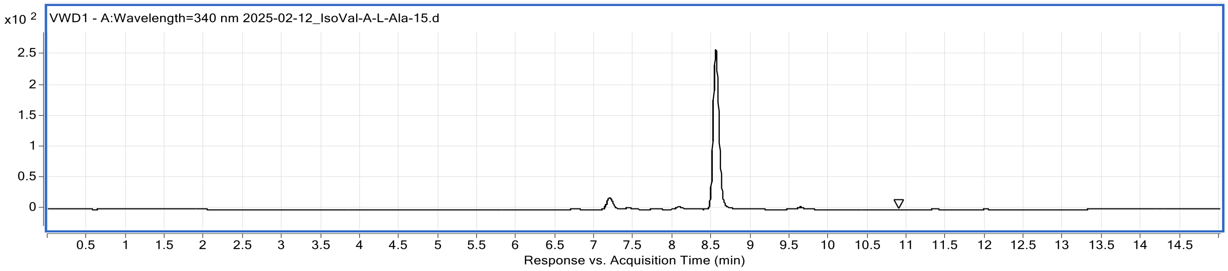

Extracted Ion Mass Chromatogram (ESI-ToF, extracted for m/z 412 ± 0.5). The Y-axis is ion counts, and the X-axis is acquisition time in minutes.

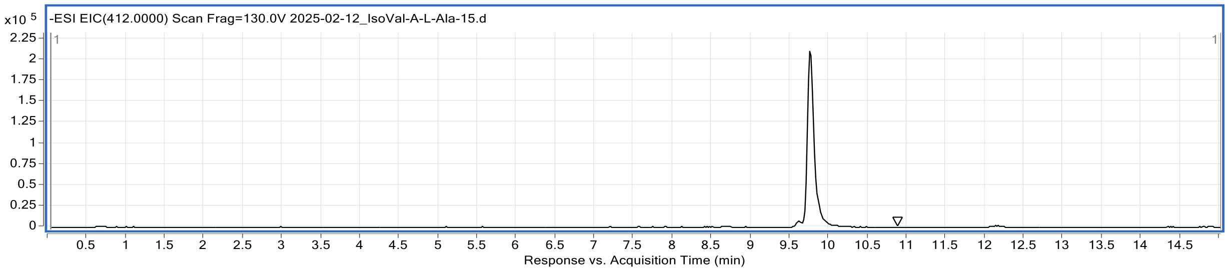

Zoomed and rescaled Extracted Ion (top) and Variable Wavelength Detector (bottom) Chromatograms. The Y axis on top is ion counts, the Y-axis on the bottom is absorbance units, and the X-axis for both is acquisition time in minutes. The peaks are labeled from left to right as Peak 1 and Peak 2.

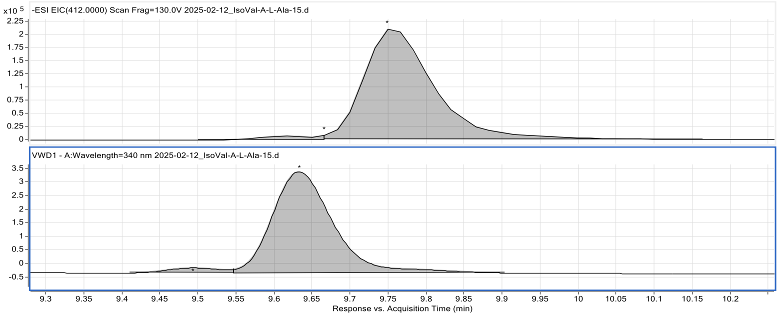

The following is a table of the peak data from the mass chromatogram. The Area % is the percent area relative to the tallest integrated peak, which has been set to 100%.

Peak	Assignment	t_R (min)	ES-ToF m/z [Neg]	Area %
1	L-*Erythro*	9.616	412.1450	3.02
2	L-*Threo*	9.749	412.1476	100

The calculated m/z for the adduct is 412.1474 for $C_{16}H_{22}N_5O_8^-$ $[M – H]^{-1}$.

The following is a table of the peak data from the variable wavelength detector chromatogram (340 nm). The Area % is the percent area relative to the tallest integrated peak, which has been set to 100%.

Peak	Assignment	t_R (min)	Area %
1	L-*Erythro*	9.493	4.44
2	L-*Threo*	9.633	100

The following is a table showing the difference in retention time on the extracted ion chromatogram (i.e., m/z Δt_R) and variable wavelength detector (i.e., VWD Δt_R) between the two diastereomers (i.e., separation = absolute difference between L-*erythro* and L-*threo*). The Average Δt_R values are the average difference in retention time between the extracted ion and variable wavelength detector chromatograms.

	m/z Δt_R (min)	VWD Δt_R (min)	Average Δt_R (min)
Peak Separation	0.133	0.140	0.14

LC/MS Traces for the Attempted Resolution of the Beta Stereoisomers of the β-hydroxy-α-Amino Acid Corresponding to Adduct 24 with 1-Fluoro-2,4-dinitrophenyl-5-ᴅ-alanine Amide (i.e., ᴅ-FDAA ᴅ-7) with HPLC Method B (i.e., 10 min.)

Total Ion Mass Chromatogram (ESI-ToF). The Y-axis is ion counts, and the X-axis is acquisition time in minutes.

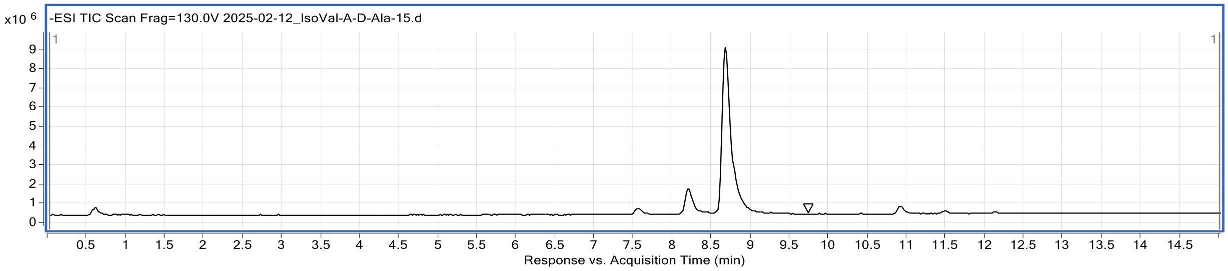

Variable Wavelength Detector Chromatogram (340 nm). The Y-axis is absorbance units, and the X-axis is acquisition time in minutes.

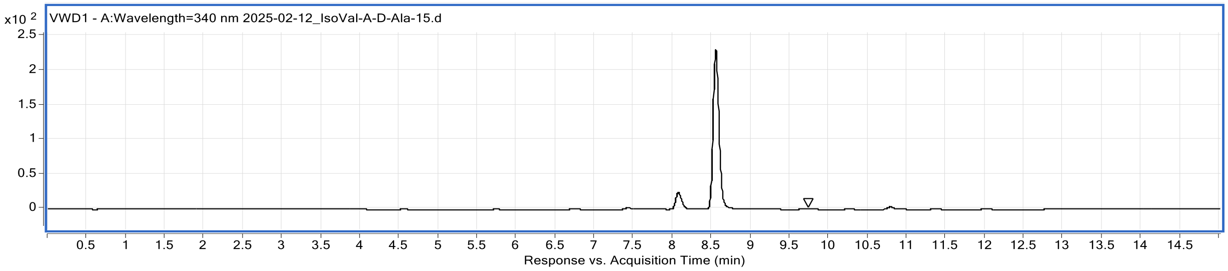

Extracted Ion Mass Chromatogram (ESI-ToF, extracted for m/z 412 ± 0.5). The Y-axis is ion counts, and the X-axis is acquisition time in minutes.

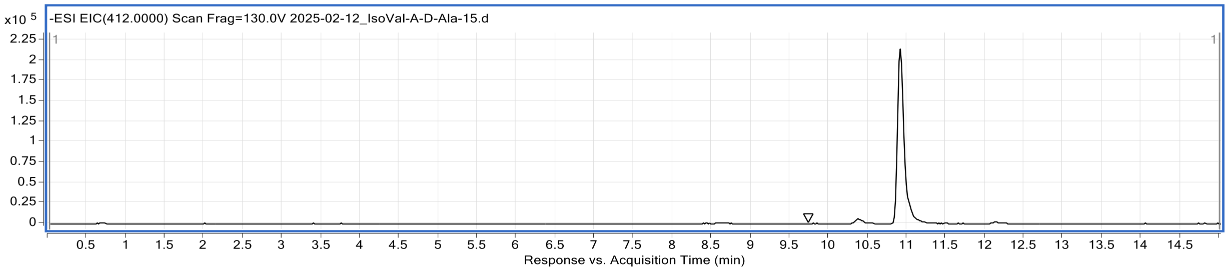

Zoomed and rescaled Extracted Ion (top) and Variable Wavelength Detector (bottom) Chromatograms. The Y axis on top is ion counts, the Y-axis on the bottom is absorbance units, and the X-axis for both is acquisition time in minutes. The peaks are labeled from left to right as Peak 1 and Peak 2.

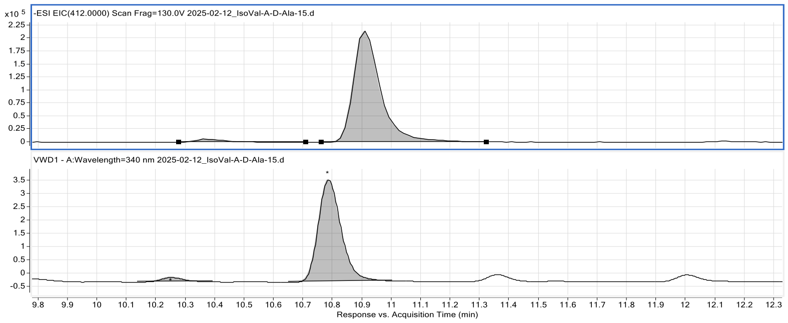

The following is a table of the peak data from the mass chromatogram. The Area % is the percent area relative to the tallest integrated peak, which has been set to 100%.

Peak	Assignment	t_R (min)	ES-ToF m/z [Neg]	Area %
1	L-*Erythro*	10.360	412.1457	2.92
2	L-*Threo*	10.908	412.1474	100

The calculated m/z for the adduct is 412.1474 for $C_{16}H_{22}N_5O_8^-$ $[M - H]^{-1}$.

The following is a table of the peak data from the variable wavelength detector chromatogram (340 nm). The Area % is the percent area relative to the tallest integrated peak, which has been set to 100%.

Peak	Assignment	t_R (min)	Area %
1	L-*Erythro*	10.250	4.32
2	L-*Threo*	10.783	100

The following is a table showing the difference in retention time on the extracted ion chromatogram (i.e., m/z Δt_R) and variable wavelength detector (i.e., VWD Δt_R) between the two diastereomers (i.e., separation = absolute difference between L-*erythro* and L-*threo*). The Average Δt_R values are the average difference in retention time between the extracted ion and variable wavelength detector chromatograms.

	m/z Δt_R (min)	VWD Δt_R (min)	Average Δt_R (min)
Peak Separation	0.548	0.533	0.54

LC/MS Traces for the Attempted Resolution of the Beta Stereoisomers of the β-hydroxy-α-Amino Acid Corresponding to Adduct 24 with 1-Fluoro-2,4-dinitrophenyl-5-L-proline Amide (i.e., L-FDPA 9) with HPLC Method B (i.e., 10 min.)

Total Ion Mass Chromatogram (ESI-ToF). The Y-axis is ion counts, and the X-axis is acquisition time in minutes.

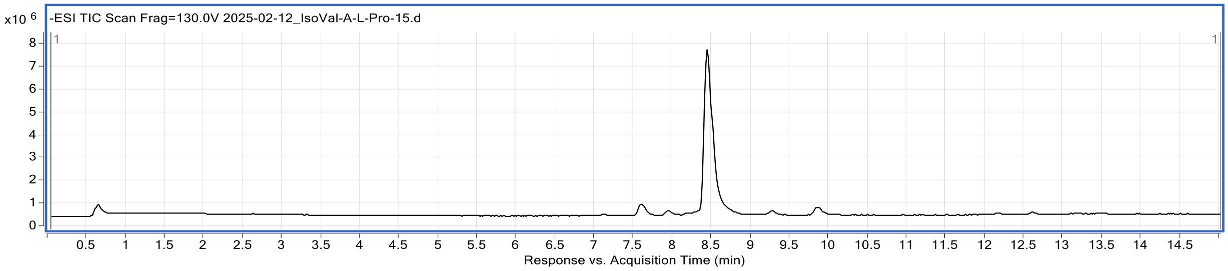

Variable Wavelength Detector Chromatogram (340 nm). The Y-axis is absorbance units, and the X-axis is acquisition time in minutes.

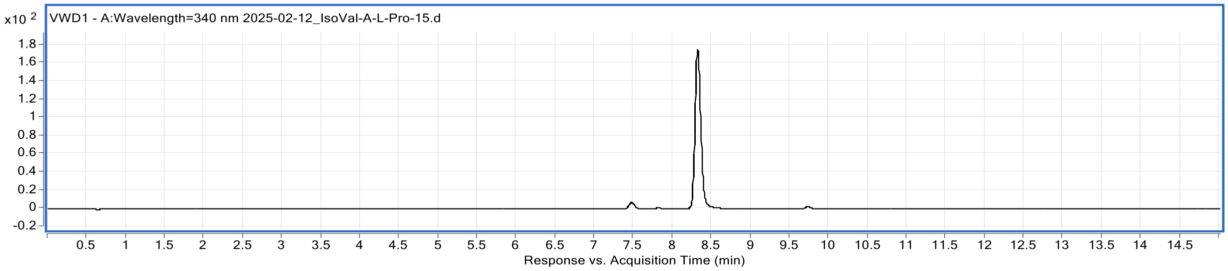

Extracted Ion Mass Chromatogram (ESI-ToF, extracted for m/z 438 ± 0.5). The Y-axis is ion counts, and the X-axis is acquisition time in minutes.

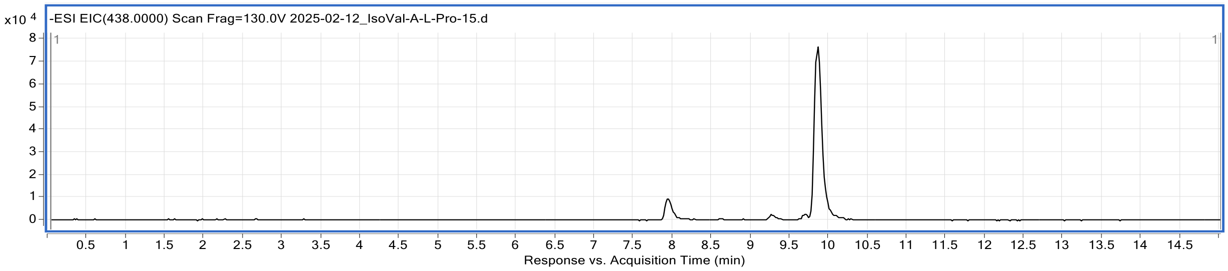

Zoomed and rescaled Extracted Ion (top) and Variable Wavelength Detector (bottom) Chromatograms. The Y axis on top is ion counts, the Y-axis on the bottom is absorbance units, and the X-axis for both is acquisition time in minutes. The peaks are labeled from left to right as Peak 1 and Peak 2.

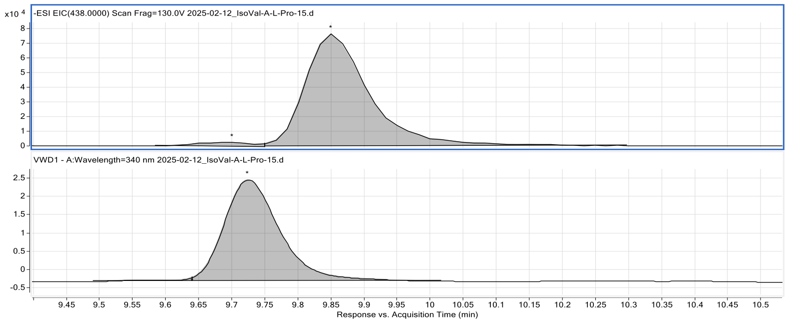

The following is a table of the peak data from the mass chromatogram. The Area % is the percent area relative to the tallest integrated peak, which has been set to 100%.

Peak	Assignment	t_R (min)	ES-ToF m/z [Neg]	Area %
1	L-*Erythro*	9.700	438.1621	3.54
2	L-*Threo*	9.850	438.1628	100

The calculated m/z for the adduct is 438.1630 for $C_{18}H_{24}N_5O_8^-$ $[M - H]^{-1}$.

The following is a table of the peak data from the variable wavelength detector chromatogram (340 nm). The Area % is the percent area relative to the tallest integrated peak, which has been set to 100%.

Peak	Assignment	t_R (min)	Area %
1	L-*Erythro*	9.640	1.62
2	L-*Threo*	9.723	100

The following is a table showing the difference in retention time on the extracted ion chromatogram (i.e., m/z Δt_R) and variable wavelength detector (i.e., VWD Δt_R) between the two diastereomers (i.e., separation = absolute difference between L-*erythro* and L-*threo*). The Average Δt_R values are the average difference in retention time between the extracted ion and variable wavelength detector chromatograms.

	m/z Δt_R (min)	VWD Δt_R (min)	Average Δt_R (min)
Peak Separation	0.150	0.083	0.12

LC/MS Traces for the Attempted Resolution of the Beta Stereoisomers of the β-hydroxy-α-Amino Acid Corresponding to Adduct 24 with 1-Fluoro-2,4-dinitrophenyl-5-ᴅ-proline Amide (i.e., ᴅ-FDPA ᴅ-9) with HPLC Method B (i.e., 10 min.)

Total Ion Mass Chromatogram (ESI-ToF). The Y-axis is ion counts, and the X-axis is acquisition time in minutes.

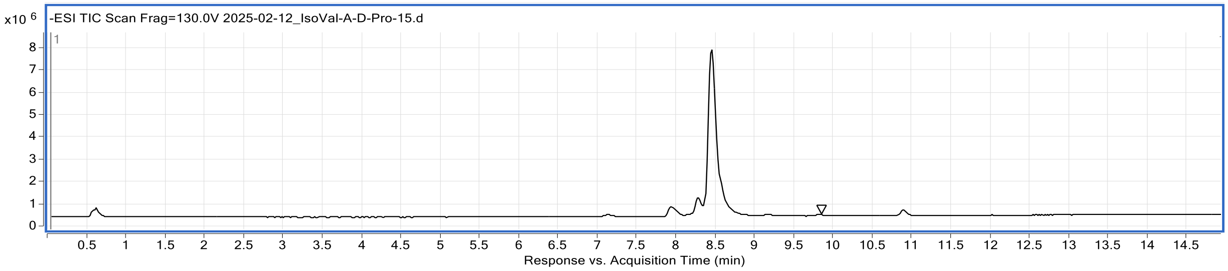

Variable Wavelength Detector Chromatogram (340 nm). The Y-axis is absorbance units, and the X-axis is acquisition time in minutes.

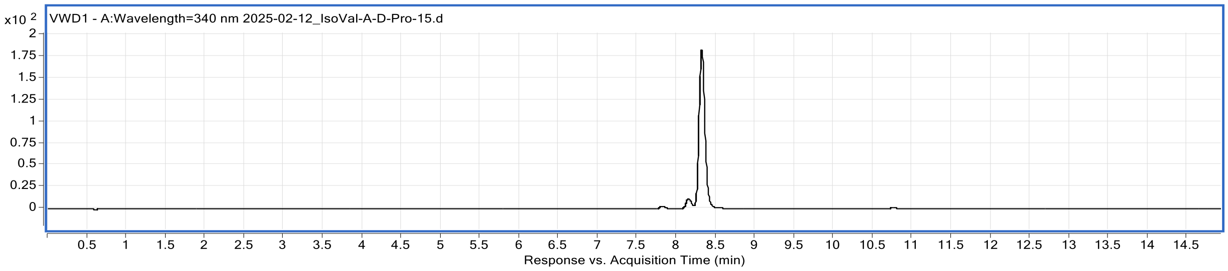

Extracted Ion Mass Chromatogram (ESI-ToF, extracted for m/z 438 ± 0.5). The Y-axis is ion counts, and the X-axis is acquisition time in minutes. Control experiments confirmed that the spurious peak at 7.9 and 9.8 min did not result from stereoisomer of the amino acid.

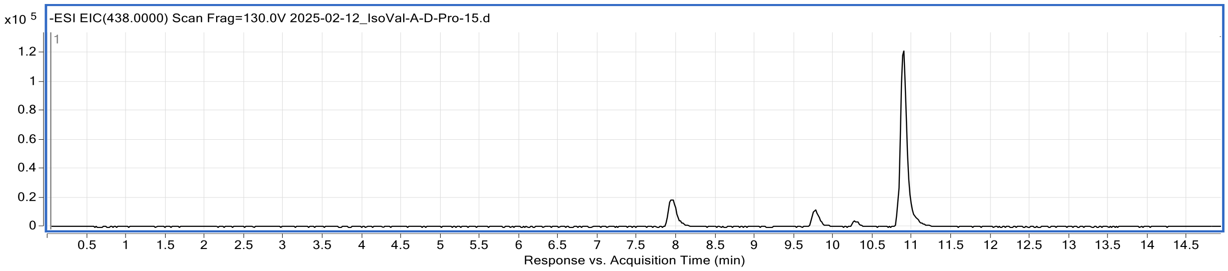

Zoomed and rescaled Extracted Ion (top) and Variable Wavelength Detector (bottom) Chromatograms. The Y axis on top is ion counts, the Y-axis on the bottom is absorbance units, and the X-axis for both is acquisition time in minutes. The peaks are labeled from left to right as Peak 1 and Peak 2.

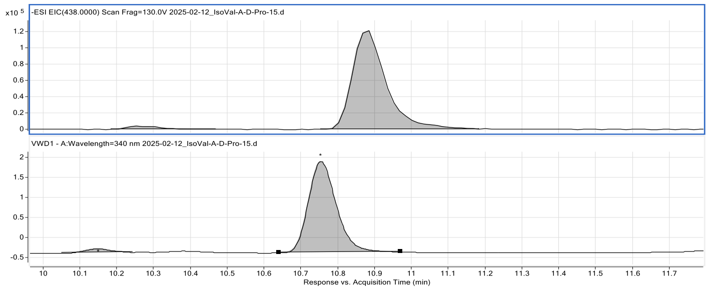

The following is a table of the peak data from the mass chromatogram. The Area % is the percent area relative to the tallest integrated peak, which has been set to 100%.

Peak	Assignment	t_R (min)	ES-ToF m/z [Neg]	Area %
1	L-*Erythro*	10.270	438.1655	3.33
2	L-*Threo*	10.884	438.1620	100

The calculated m/z for the adduct is 438.1630 for $C_{18}H_{24}N_5O_8^-$ $[M - H]^{-1}$.

The following is a table of the peak data from the variable wavelength detector chromatogram (340 nm). The Area % is the percent area relative to the tallest integrated peak, which has been set to 100%.

Peak	Assignment	t_R (min)	Area %
1	L-*Erythro*	10.150	3.89
2	L-*Threo*	10.753	100

The following is a table showing the difference in retention time on the extracted ion chromatogram (i.e., m/z Δt_R) and variable wavelength detector (i.e., VWD Δt_R) between the two diastereomers (i.e., separation = absolute difference between L-*erythro* and L-*threo*). The Average Δt_R values are the average difference in retention time between the extracted ion and variable wavelength detector chromatograms.

	m/z Δt_R (min)	VWD Δt_R (min)	Average Δt_R (min)
Peak Separation	0.614	0.603	0.61

LC/MS Traces for the Attempted Resolution of the Beta Stereoisomers of the β-hydroxy-α-Amino Acid Corresponding to Adduct 24 with 1-Fluoro-2,4-dinitrobenzene (i.e., Sanger's reagent 10) with HPLC Method B (i.e., 10 min.)

Total Ion Mass Chromatogram (ESI-ToF). The Y-axis is ion counts, and the X-axis is acquisition time in minutes.

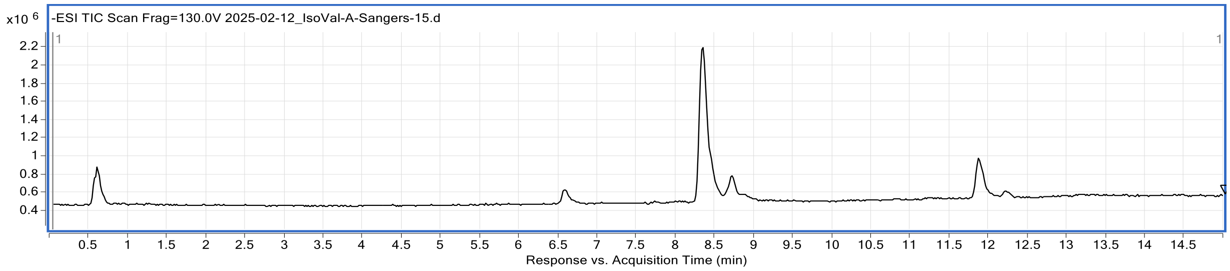

Variable Wavelength Detector Chromatogram (340 nm). The Y-axis is absorbance units, and the X-axis is acquisition time in minutes.

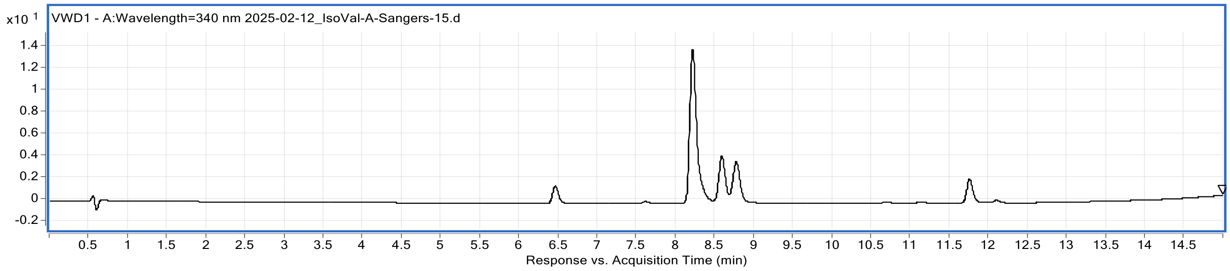

Extracted Ion Mass Chromatogram (ESI-ToF, extracted for m/z 326 ± 0.5). The Y-axis is ion counts, and the X-axis is acquisition time in minutes.

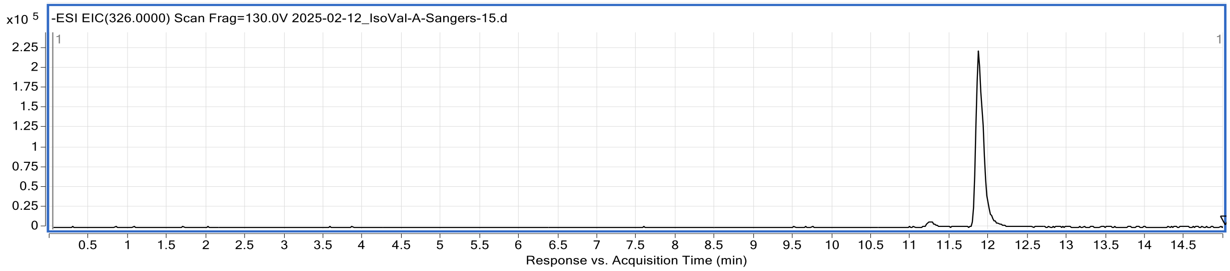

Zoomed and rescaled Extracted Ion (top) and Variable Wavelength Detector (bottom) Chromatograms. The Y axis on top is ion counts, the Y-axis on the bottom is absorbance units, and the X-axis for both is acquisition time in minutes. The peaks are labeled from left to right as Peak 1 and Peak 2.

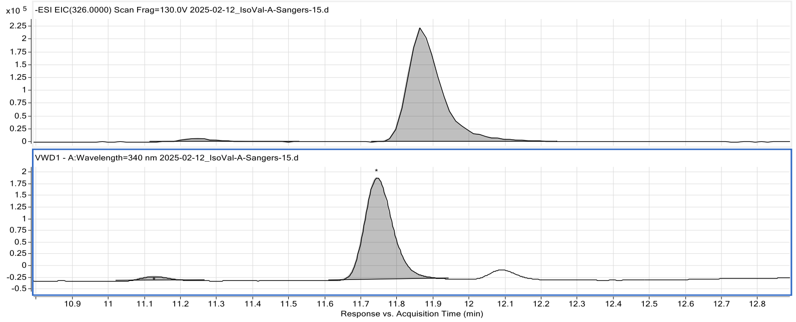

The following is a table of the peak data from the mass chromatogram. The Area % is the percent area relative to the tallest integrated peak, which has been set to 100%.

Peak	Assignment	t_R (min)	ES-ToF m/z [Neg]	Area %
1	L-*Erythro*	11.247	326.0979	3.35
2	L-*Threo*	11.861	326.0998	100

The calculated m/z for the adduct is 326.0994 for $C_{13}H_{16}N_3O_7^-$ $[M - H]^{-1}$.

The following is a table of the peak data from the variable wavelength detector chromatogram (340 nm). The Area % is the percent area relative to the tallest integrated peak, which has been set to 100%.

Peak	Assignment	t_R (min)	Area %
1	L-*Erythro*	11.127	4.21
2	L-*Threo*	11.743	100

The following is a table showing the difference in retention time on the extracted ion chromatogram (i.e., m/z Δt_R) and variable wavelength detector (i.e., VWD Δt_R) between the two diastereomers (i.e., separation = absolute difference between L-*erythro* and L-*threo*). The Average Δt_R values are the average difference in retention time between the extracted ion and variable wavelength detector chromatograms.

	m/z Δt_R (min)	VWD Δt_R (min)	Average Δt_R (min)
Peak Separation	0.614	0.616	0.62

LC/MS Traces for the Attempted Resolution of the Beta Stereoisomers of the β-hydroxy-α-Amino Acid Corresponding to Adduct 25 with 1-Fluoro-2,4-dinitrophenyl-5-L-alanine Amide (i.e., L-FDAA 7) with HPLC Method A (i.e., 25 min.)

Total Ion Mass Chromatogram (ESI-ToF). The Y-axis is ion counts, and the X-axis is acquisition time in minutes.

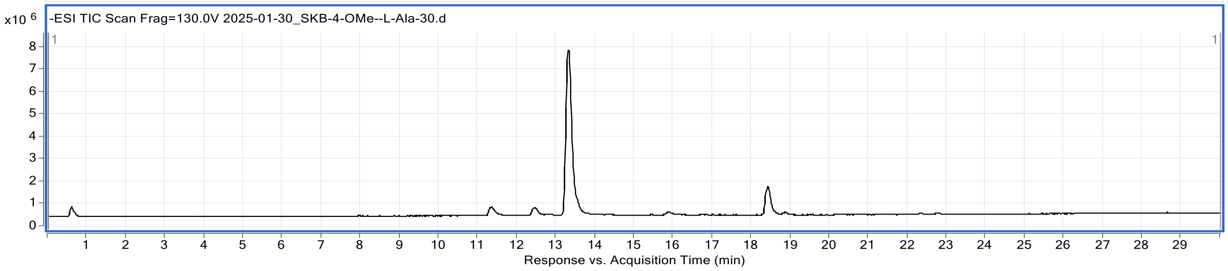

Variable Wavelength Detector Chromatogram (340 nm). The Y-axis is absorbance units, and the X-axis is acquisition time in minutes.

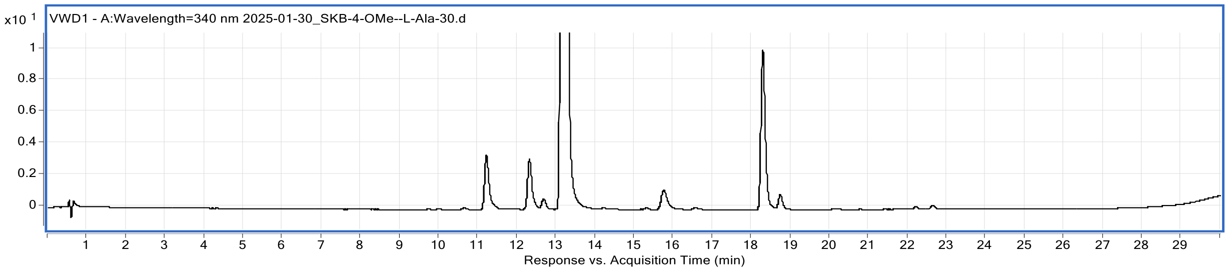

Extracted Ion Mass Chromatogram (ESI-ToF, extracted for m/z 490 ± 0.5). The Y-axis is ion counts, and the X-axis is acquisition time in minutes.

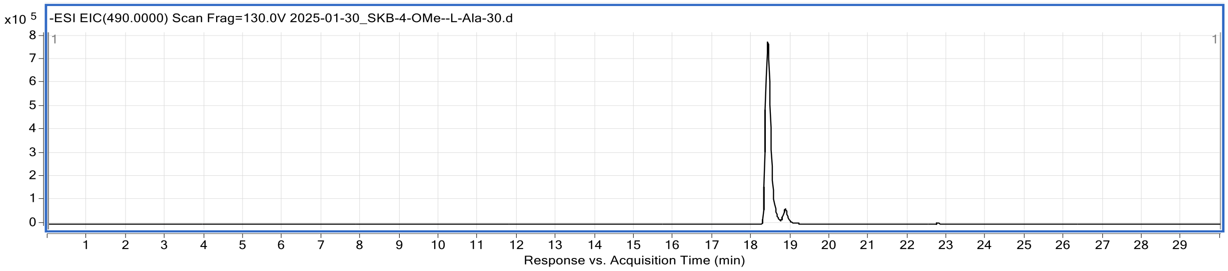

Zoomed and rescaled Extracted Ion (top) and Variable Wavelength Detector (bottom) Chromatograms. The Y axis on top is ion counts, the Y-axis on the bottom is absorbance units, and the X-axis for both is acquisition time in minutes. The peaks are labeled from left to right as Peak 1 and Peak 2.

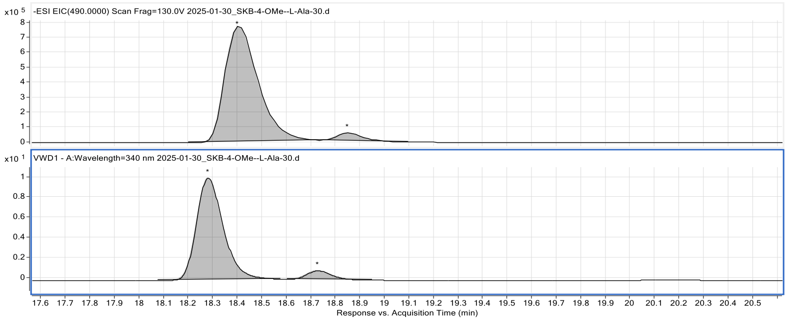

The following is a table of the peak data from the mass chromatogram. The Area % is the percent area relative to the tallest integrated peak, which has been set to 100%.

Peak	Assignment	t_R (min)	ES-ToF m/z [Neg]	Area %
1	L-*Threo*	18.400	490.1586	100
2	L-*Erythro*	18.848	490.1577	5.28

The calculated m/z for the adduct is 490.1580 for $C_{21}H_{24}N_5O_9^-$ $[M - H]^{-1}$.

The following is a table of the peak data from the variable wavelength detector chromatogram (340 nm). The Area % is the percent area relative to the tallest integrated peak, which has been set to 100%.

Peak	Assignment	t_R (min)	Area %
1	L-*Threo*	18.280	100
2	L-*Erythro*	18.720	8.03

The following is a table showing the difference in retention time on the extracted ion chromatogram (i.e., m/z Δt_R) and variable wavelength detector (i.e., VWD Δt_R) between the two diastereomers (i.e., separation = absolute difference between L-*erythro* and L-*threo*). The Average Δt_R values are the average difference in retention time between the extracted ion and variable wavelength detector chromatograms.

	m/z Δt_R (min)	VWD Δt_R (min)	Average Δt_R (min)
Peak Separation	0.448	0.440	0.44

LC/MS Traces for the Attempted Resolution of the Beta Stereoisomers of the β-hydroxy-α-Amino Acid Corresponding to Adduct 25 with 1-Fluoro-2,4-dinitrophenyl-5-D-alanine Amide (i.e., D-FDAA D-7) with HPLC Method A (i.e., 25 min.)

Total Ion Mass Chromatogram (ESI-ToF). The Y-axis is ion counts, and the X-axis is acquisition time in minutes.

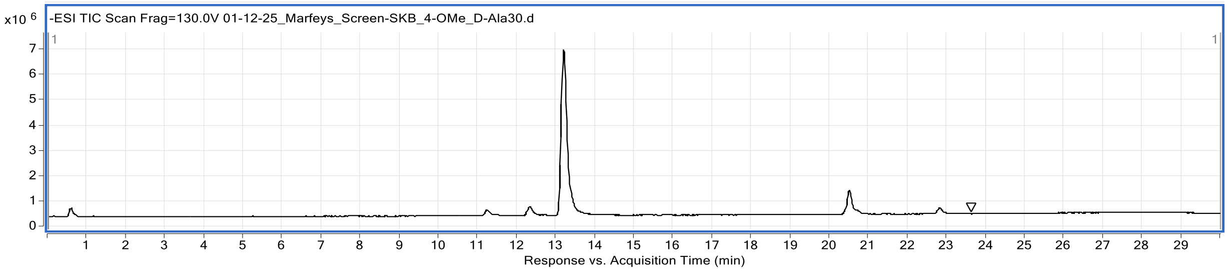

Variable Wavelength Detector Chromatogram (340 nm). The Y-axis is absorbance units, and the X-axis is acquisition time in minutes.

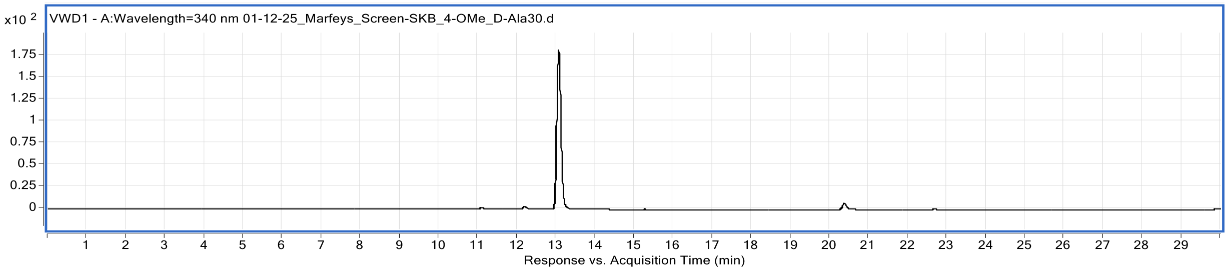

Extracted Ion Mass Chromatogram (ESI-ToF, extracted for m/z 490 ± 0.5). The Y-axis is ion counts, and the X-axis is acquisition time in minutes.

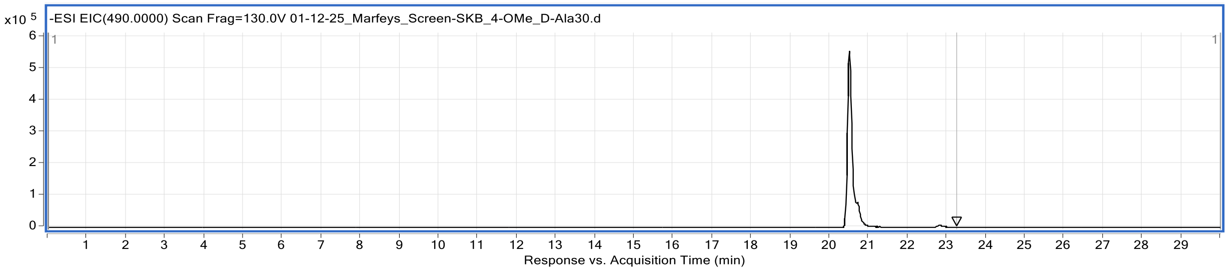

Zoomed and rescaled Extracted Ion (top) and Variable Wavelength Detector (bottom) Chromatograms. The Y axis on top is ion counts, the Y-axis on the bottom is absorbance units, and the X-axis for both is acquisition time in minutes. The peaks are labeled from left to right as Peak 1 and Peak 2.

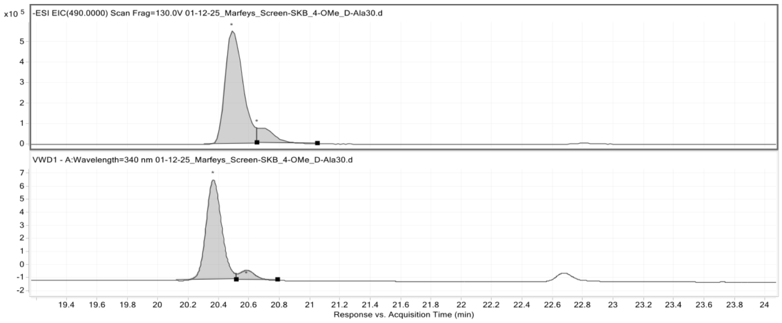

The following is a table of the peak data from the mass chromatogram. The Area % is the percent area relative to the tallest integrated peak, which has been set to 100%.

Peak	Assignment	t_R (min)	ES-ToF m/z [Neg]	Area %
1	L-*Threo*	20.487	490.1580	100
2	L-*Erythro*	20.702	490.1586	12.29

The calculated m/z for the adduct is 490.1580 for $C_{21}H_{24}N_5O_9^-$ $[M - H]^{-1}$.

The following is a table of the peak data from the variable wavelength detector chromatogram (340 nm). The Area % is the percent area relative to the tallest integrated peak, which has been set to 100%.

Peak	Assignment	t_R (min)	Area %
1	L-*Threo*	20.363	100
2	L-*Erythro*	20.583	10.16

The following is a table showing the difference in retention time on the extracted ion chromatogram (i.e., m/z Δt_R) and variable wavelength detector (i.e., VWD Δt_R) between the two diastereomers (i.e., separation = absolute difference between L-*erythro* and L-*threo*). The Average Δt_R values are the average difference in retention time between the extracted ion and variable wavelength detector chromatograms.

	m/z Δt_R (min)	VWD Δt_R (min)	Average Δt_R (min)
Peak Separation	0.215	0.220	0.22

LC/MS Traces for the Attempted Resolution of the Beta Stereoisomers of the β-hydroxy-α-Amino Acid Corresponding to Adduct 25 with 1-Fluoro-2,4-dinitrophenyl-5-L-proline Amide (i.e., L-FDPA 9) with HPLC Method A (i.e., 25 min.)

Total Ion Mass Chromatogram (ESI-ToF). The Y-axis is ion counts, and the X-axis is acquisition time in minutes.

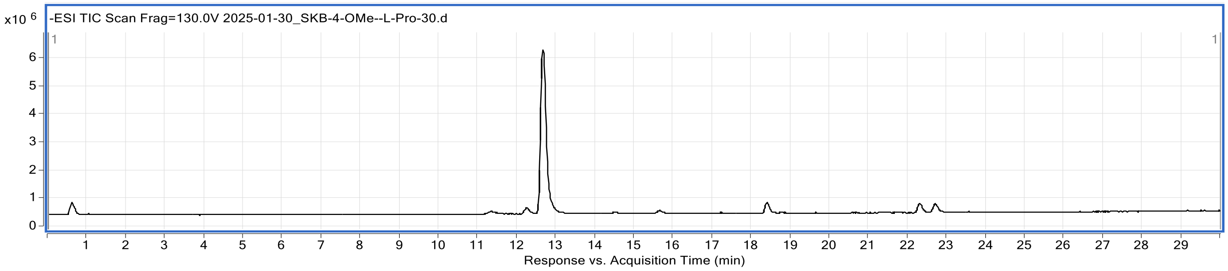

Variable Wavelength Detector Chromatogram (340 nm). The Y-axis is absorbance units, and the X-axis is acquisition time in minutes.

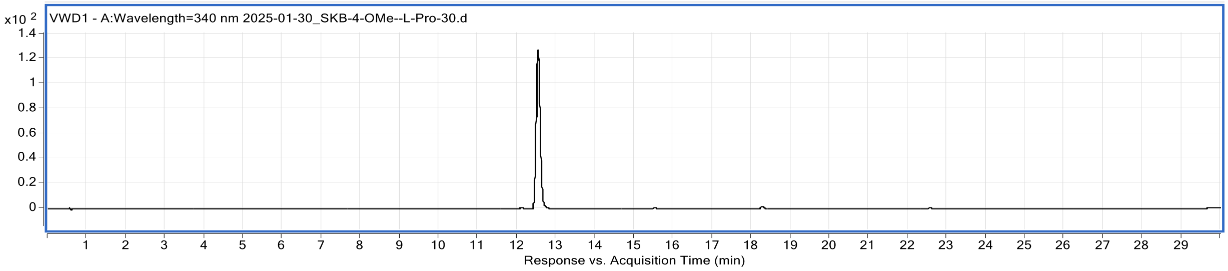

Extracted Ion Mass Chromatogram (ESI-ToF, extracted for m/z 516 ± 0.5). The Y-axis is ion counts, and the X-axis is acquisition time in minutes.

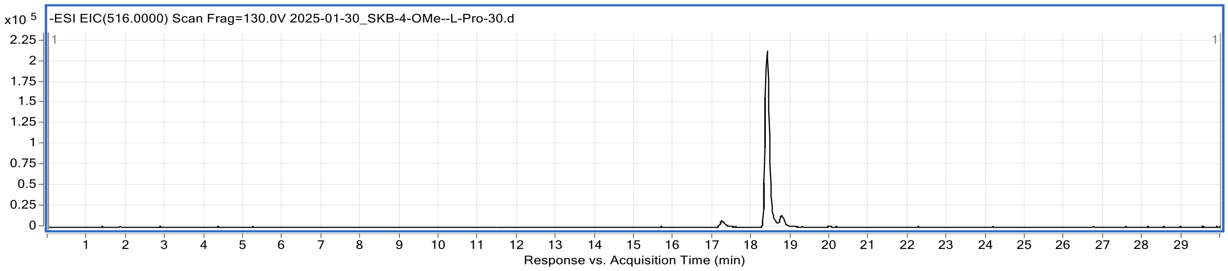

Zoomed and rescaled Extracted Ion (top) and Variable Wavelength Detector (bottom) Chromatograms. The Y axis on top is ion counts, the Y-axis on the bottom is absorbance units, and the X-axis for both is acquisition time in minutes. The peaks are labeled from left to right as Peak 1 and Peak 2.

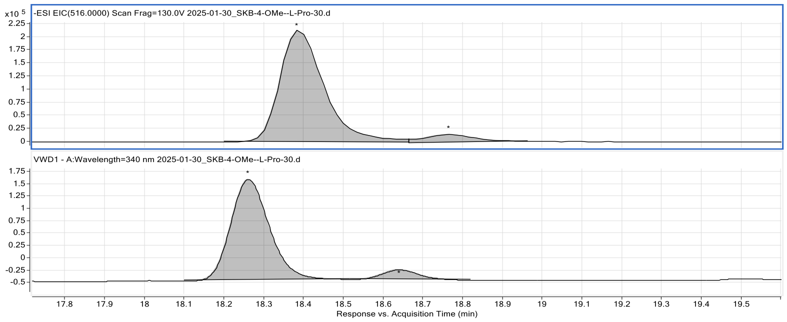

The following is a table of the peak data from the mass chromatogram. The Area % is the percent area relative to the tallest integrated peak, which has been set to 100%.

Peak	Assignment	t_R (min)	ES-ToF m/z [Neg]	Area %
1	L-*Threo*	18.383	516.1759	100
2	L-*Erythro*	18.765	516.1734	8.47

The calculated m/z for the adduct is 516.1736 for $C_{23}H_{26}N_5O_9^-$ $[M - H]^{-1}$.

The following is a table of the peak data from the variable wavelength detector chromatogram (340 nm). The Area % is the percent area relative to the tallest integrated peak, which has been set to 100%.

Peak	Assignment	t_R (min)	Area %
1	L-*Threo*	18.260	100
2	L-*Erythro*	18.640	9.31

The following is a table showing the difference in retention time on the extracted ion chromatogram (i.e., m/z Δt_R) and variable wavelength detector (i.e., VWD Δt_R) between the two diastereomers (i.e., separation = absolute difference between L-*erythro* and L-*threo*). The Average Δt_R values are the average difference in retention time between the extracted ion and variable wavelength detector chromatograms.

	m/z Δt_R (min)	VWD Δt_R (min)	Average Δt_R (min)
Peak Separation	0.382	0.380	0.38

LC/MS Traces for the Attempted Resolution of the Beta Stereoisomers of the β-hydroxy-α-Amino Acid Corresponding to Adduct 25 with 1-Fluoro-2,4-dinitrophenyl-5-D-proline Amide (i.e., D-FDPA D-9) with HPLC Method A (i.e., 25 min.)

Total Ion Mass Chromatogram (ESI-ToF). The Y-axis is ion counts, and the X-axis is acquisition time in minutes.

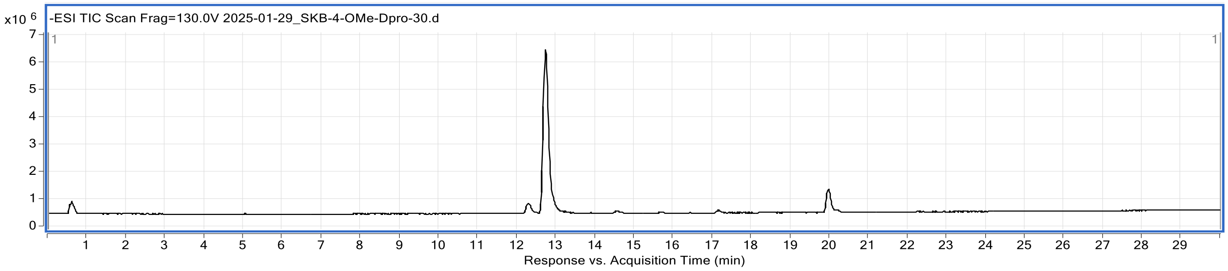

Variable Wavelength Detector Chromatogram (340 nm). The Y-axis is absorbance units, and the X-axis is acquisition time in minutes.

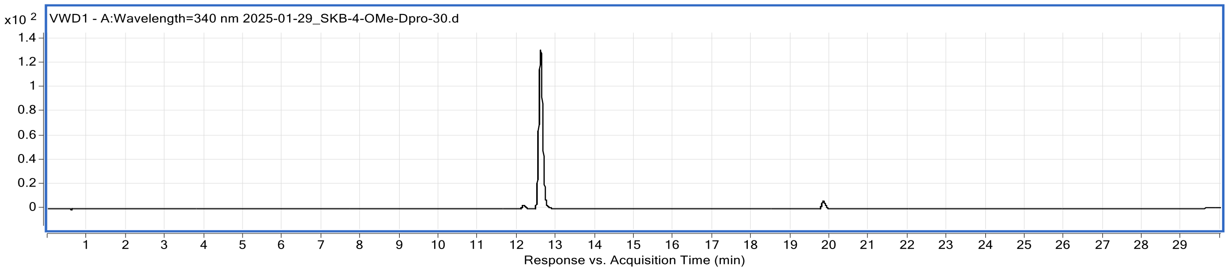

Extracted Ion Mass Chromatogram (ESI-ToF, extracted for m/z 516 ± 0.5). The Y-axis is ion counts, and the X-axis is acquisition time in minutes. Control experiments confirmed that the spurious peak at 17.3 min did not result from stereoisomer of the amino acid.

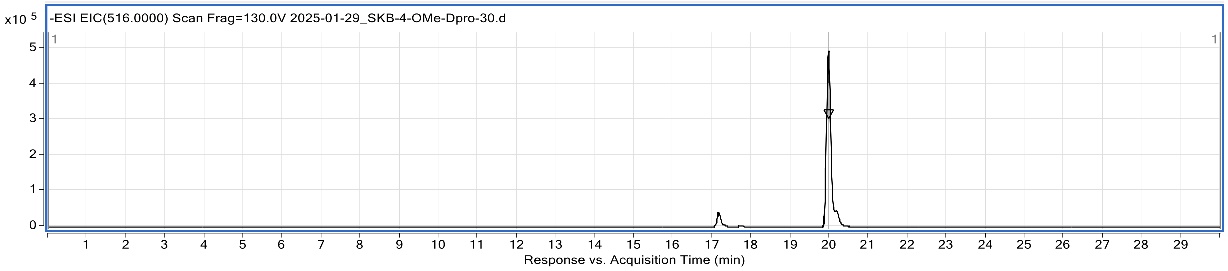

Zoomed and rescaled Extracted Ion (top) and Variable Wavelength Detector (bottom) Chromatograms. The Y axis on top is ion counts, the Y-axis on the bottom is absorbance units, and the X-axis for both is acquisition time in minutes. The peaks are labeled from left to right as Peak 1 and Peak 2.

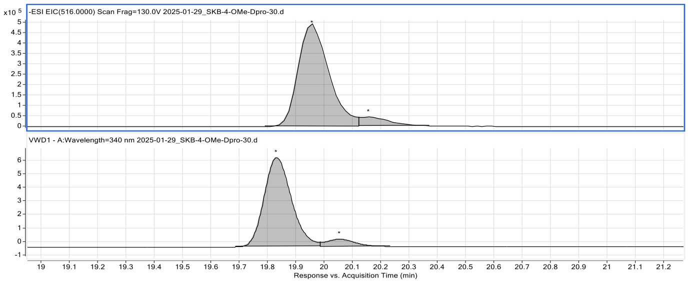

The following is a table of the peak data from the mass chromatogram. The Area % is the percent area relative to the tallest integrated peak, which has been set to 100%.

Peak	Assignment	t_R (min)	ES-ToF m/z [Neg]	Area %
1	L-*Threo*	19.957	516.1764	100
2	L-*Erythro*	20.156	516.1757	7.98

The calculated m/z for the adduct is 516.1736 for $C_{23}H_{26}N_5O_9^-$ $[M - H]^{-1}$.

The following is a table of the peak data from the variable wavelength detector chromatogram (340 nm). The Area % is the percent area relative to the tallest integrated peak, which has been set to 100%.

Peak	Assignment	t_R (min)	Area %
1	L-*Threo*	19.830	100
2	L-*Erythro*	20.053	9.42

The following is a table showing the difference in retention time on the extracted ion chromatogram (i.e., m/z Δt_R) and variable wavelength detector (i.e., VWD Δt_R) between the two diastereomers (i.e., separation = absolute difference between L-*erythro* and L-*threo*). The Average Δt_R values are the average difference in retention time between the extracted ion and variable wavelength detector chromatograms.

	m/z Δt_R (min)	VWD Δt_R (min)	Average Δt_R (min)
Peak Separation	0.199	0.223	0.22

LC/MS Traces for the Attempted Resolution of the Beta Stereoisomers of the β-hydroxy-α-Amino Acid Corresponding to Adduct 25 with 1-Fluoro-2,4-dinitrobenzene (i.e., Sanger's reagent 10) with HPLC Method A (i.e., 25 min.)

Total Ion Mass Chromatogram (ESI-ToF). The Y-axis is ion counts, and the X-axis is acquisition time in minutes.

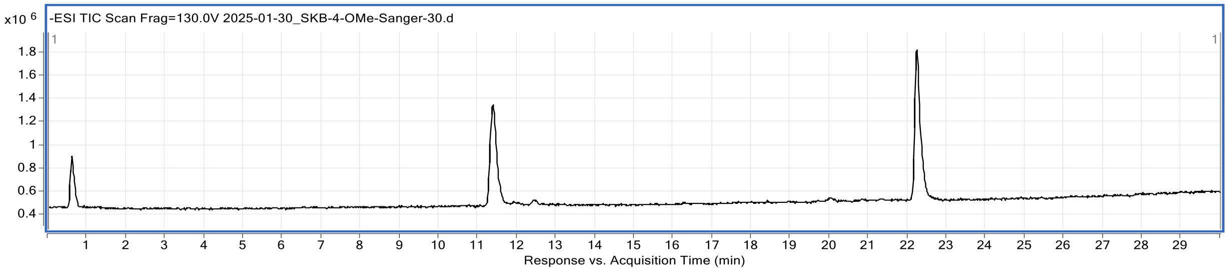

Variable Wavelength Detector Chromatogram (340 nm). The Y-axis is absorbance units, and the X-axis is acquisition time in minutes.

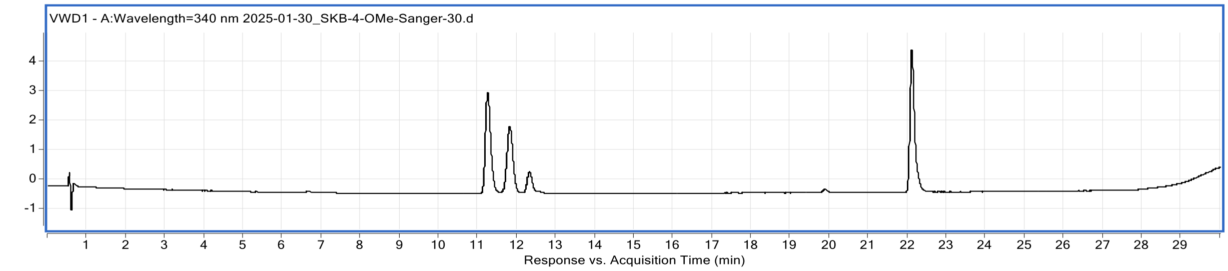

Extracted Ion Mass Chromatogram (ESI-ToF, extracted for m/z 404 ± 0.5). The Y-axis is ion counts, and the X-axis is acquisition time in minutes.

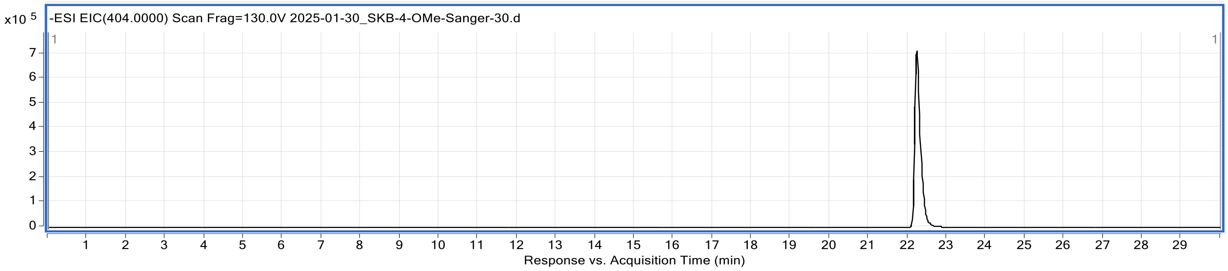

Zoomed and rescaled Extracted Ion (top) and Variable Wavelength Detector (bottom) Chromatograms. The Y axis on top is ion counts, the Y-axis on the bottom is absorbance units, and the X-axis for both is acquisition time in minutes. The minor adduct can be seen from the peak tailing, but the resolution is not sufficient to evaluate isomer separation.

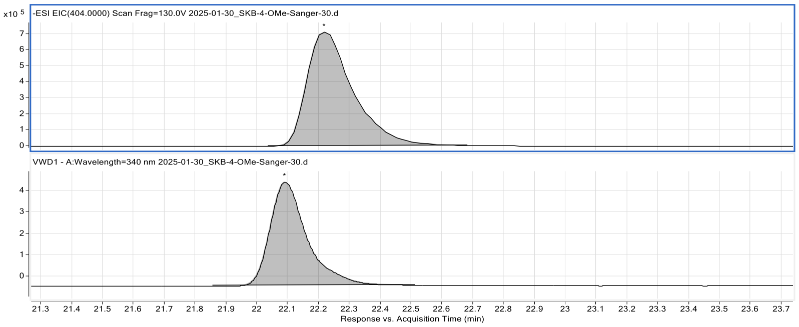

The following is a table of the peak data from the mass chromatogram. The Area % is the percent area relative to the tallest integrated peak, which has been set to 100%.

Peak	Assignment	t_R (min)	ES-ToF m/z [Neg]	Area %
1	L-*Threo*/ L-*Erythro*	22.218	404.1129	100

The calculated m/z for the adduct is 404.1099 for $C_{18}H_{18}N_3O_8^-$ $[M – H]^{-1}$.

The following is a table of the peak data from the variable wavelength detector chromatogram (340 nm). The Area % is the percent area relative to the tallest integrated peak, which has been set to 100%.

Peak	Assignment	t_R (min)	Area %
1	L-*Threo*/ L-*Erythro*	22.090	100

The following is a table showing the difference in retention time on the extracted ion chromatogram (i.e., m/z Δt_R) and variable wavelength detector (i.e., VWD Δt_R) between the two diastereomers (i.e., separation = absolute difference between L-*erythro* and L-*threo*). The Average Δt_R values are the average difference in retention time between the extracted ion and variable wavelength detector chromatograms.

	m/z Δt_R (min)	VWD Δt_R (min)	Average Δt_R (min)
Peak Separation	0	0	0

LC/MS Traces for the Attempted Resolution of the Beta Stereoisomers of the β-hydroxy-α-Amino Acid Corresponding to Adduct 26 with 1-Fluoro-2,4-dinitrophenyl-5-L-alanine Amide (i.e., L-FDAA 7) with HPLC Method A (i.e., 25 min.)

Total Ion Mass Chromatogram (ESI-ToF). The Y-axis is ion counts, and the X-axis is acquisition time in minutes.

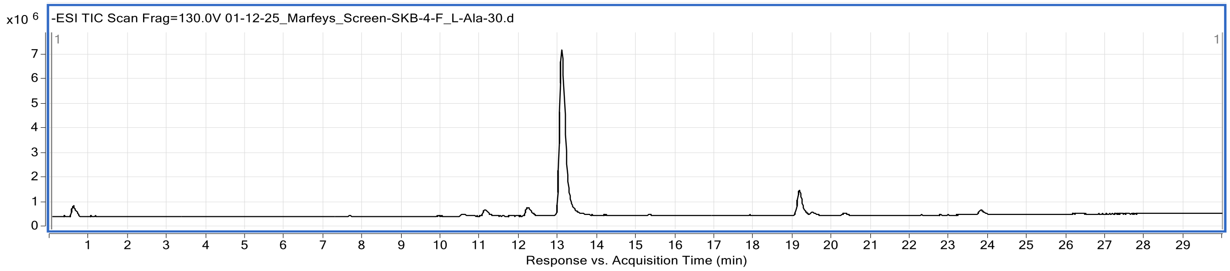

Variable Wavelength Detector Chromatogram (340 nm). The Y-axis is absorbance units, and the X-axis is acquisition time in minutes.

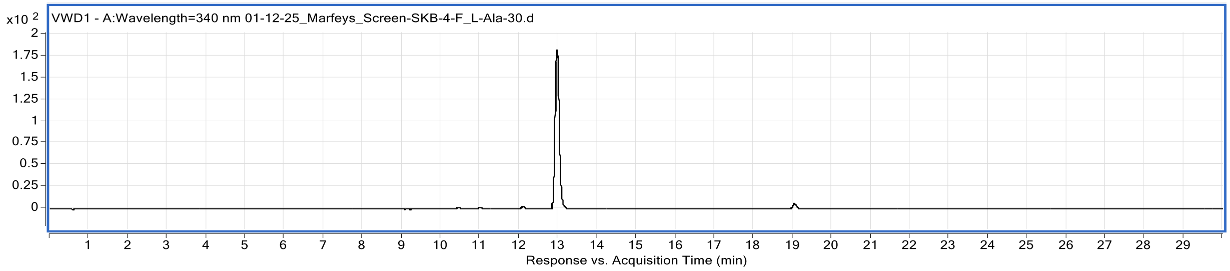

Extracted Ion Mass Chromatogram (ESI-ToF, extracted for m/z 478 ± 0.5). The Y-axis is ion counts, and the X-axis is acquisition time in minutes.

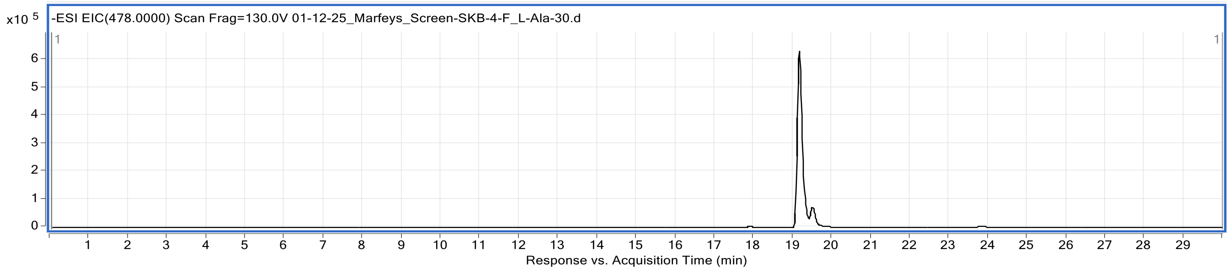

Zoomed and rescaled Extracted Ion (top) and Variable Wavelength Detector (bottom) Chromatograms. The Y axis on top is ion counts, the Y-axis on the bottom is absorbance units, and the X-axis for both is acquisition time in minutes. The peaks are labeled from left to right as Peak 1 and Peak 2.

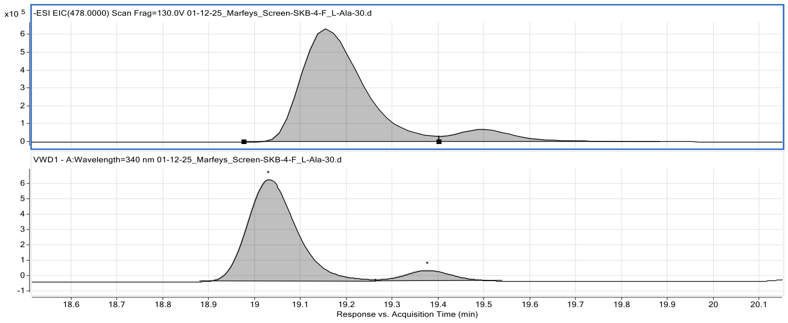

The following is a table of the peak data from the mass chromatogram. The Area % is the percent area relative to the tallest integrated peak, which has been set to 100%.

Peak	Assignment	t_R (min)	ES-ToF m/z [Neg]	Area %
1	L-*Threo*	19.153	478.1403	100
2	L-*Erythro*	19.501	478.1405	11.75

The calculated m/z for the adduct is 478.1380 for $C_{20}H_{21}FN_5O_8^-$ $[M - H]^{-1}$.

The following is a table of the peak data from the variable wavelength detector chromatogram (340 nm). The Area % is the percent area relative to the tallest integrated peak, which has been set to 100%.

Peak	Assignment	t_R (min)	Area %
1	L-*Threo*	19.030	100
2	L-*Erythro*	19.377	10.66

The following is a table showing the difference in retention time on the extracted ion chromatogram (i.e., m/z Δt_R) and variable wavelength detector (i.e., VWD Δt_R) between the two diastereomers (i.e., separation = absolute difference between L-*erythro* and L-*threo*). The Average Δt_R values are the average difference in retention time between the extracted ion and variable wavelength detector chromatograms.

	m/z Δt_R (min)	VWD Δt_R (min)	Average Δt_R (min)
Peak Separation	0.348	0.347	0.35

LC/MS Traces for the Attempted Resolution of the Beta Stereoisomers of the β-hydroxy-α-Amino Acid Corresponding to Adduct 26 with 1-Fluoro-2,4-dinitrophenyl-5-D-alanine Amide (i.e., D-FDAA D-7) with HPLC Method A (i.e., 25 min.)

Total Ion Mass Chromatogram (ESI-ToF). The Y-axis is ion counts, and the X-axis is acquisition time in minutes.

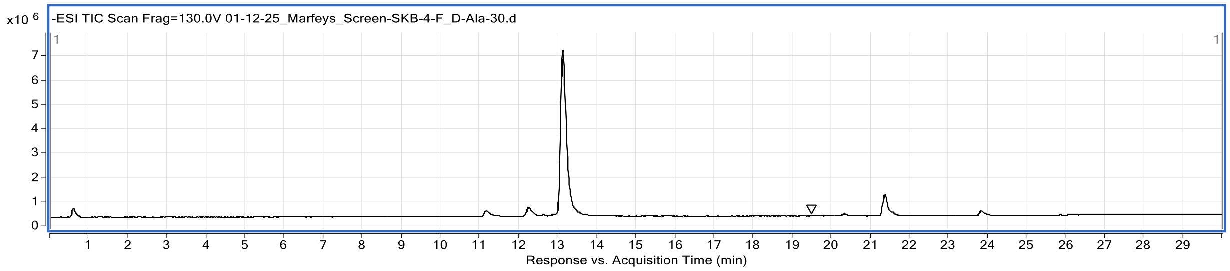

Variable Wavelength Detector Chromatogram (340 nm). The Y-axis is absorbance units, and the X-axis is acquisition time in minutes.

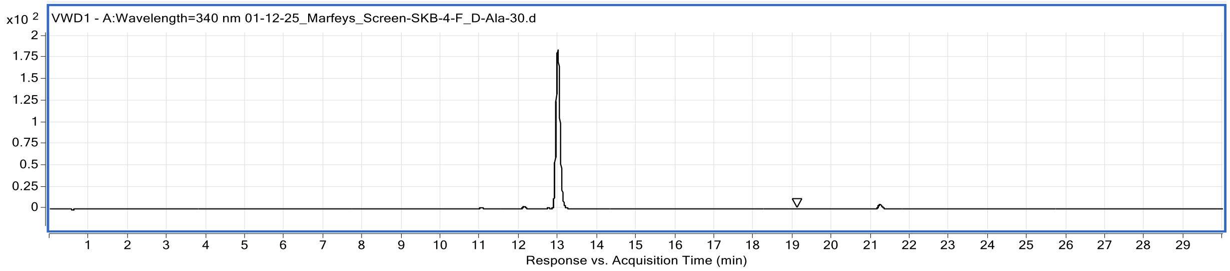

Extracted Ion Mass Chromatogram (ESI-ToF, extracted for m/z 478 ± 0.5). The Y-axis is ion counts, and the X-axis is acquisition time in minutes.

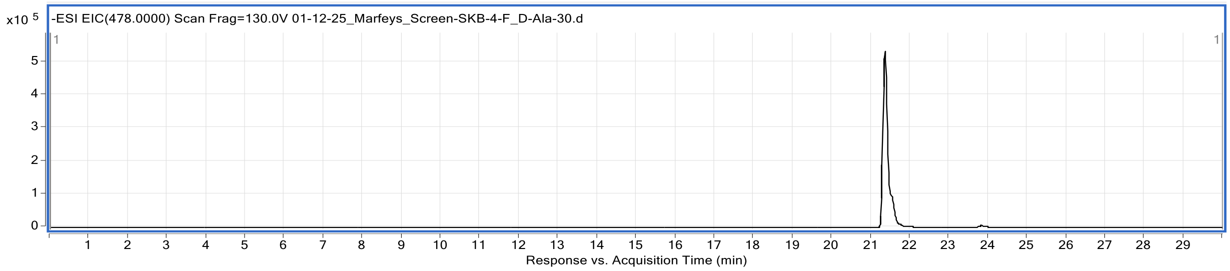

Zoomed and rescaled Extracted Ion (top) and Variable Wavelength Detector (bottom) Chromatograms. The Y axis on top is ion counts, the Y-axis on the bottom is absorbance units, and the X-axis for both is acquisition time in minutes. The peaks are labeled from left to right as Peak 1 and Peak 2.

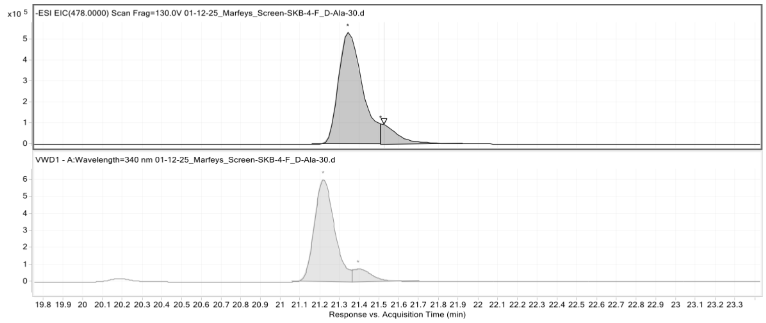

The following is a table of the peak data from the mass chromatogram. The Area % is the percent area relative to the tallest integrated peak, which has been set to 100%.

Peak	Assignment	t_R (min)	ES-ToF m/z [Neg]	Area %
1	L-*Threo*	21.342	478.1403	100
2	L-*Erythro*	21.524	478.1395	13.57

The calculated m/z for the adduct is 478.1380 for $C_{20}H_{21}FN_5O_8^-$ $[M - H]^{-1}$.

The following is a table of the peak data from the variable wavelength detector chromatogram (340 nm). The Area % is the percent area relative to the tallest integrated peak, which has been set to 100%.

Peak	Assignment	t_R (min)	Area %
1	L-*Threo*	21.217	100
2	L-*Erythro*	21.393	12.72

The following is a table showing the difference in retention time on the extracted ion chromatogram (i.e., m/z Δt_R) and variable wavelength detector (i.e., VWD Δt_R) between the two diastereomers (i.e., separation = absolute difference between L-*erythro* and L-*threo*). The Average Δt_R values are the average difference in retention time between the extracted ion and variable wavelength detector chromatograms.

	m/z Δt_R (min)	VWD Δt_R (min)	Average Δt_R (min)
Peak Separation	0.182	0.176	0.18

LC/MS Traces for the Attempted Resolution of the Beta Stereoisomers of the β-hydroxy-α-Amino Acid Corresponding to Adduct 26 with 1-Fluoro-2,4-dinitrophenyl-5-L-proline Amide (i.e., L-FDPA 9) with HPLC Method A (i.e., 25 min.)

Total Ion Mass Chromatogram (ESI-ToF). The Y-axis is ion counts, and the X-axis is acquisition time in minutes.

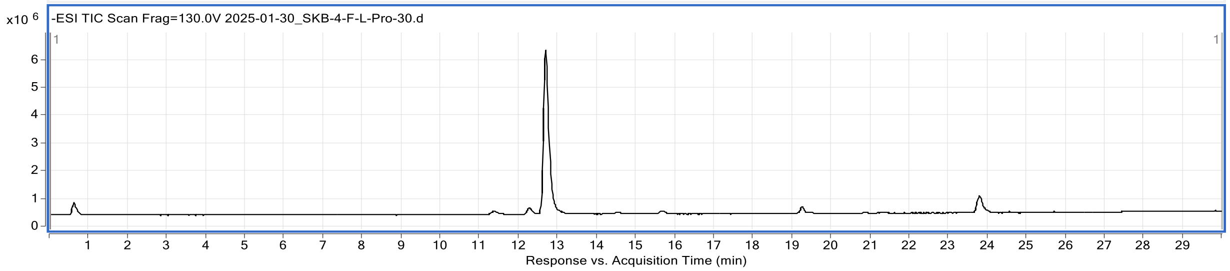

Variable Wavelength Detector Chromatogram (340 nm). The Y-axis is absorbance units, and the X-axis is acquisition time in minutes.

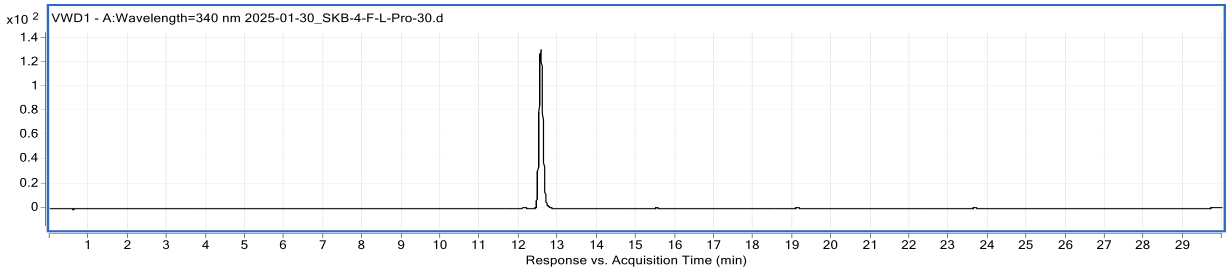

Extracted Ion Mass Chromatogram (ESI-ToF, extracted for m/z 504 ± 0.5). The Y-axis is ion counts, and the X-axis is acquisition time in minutes.

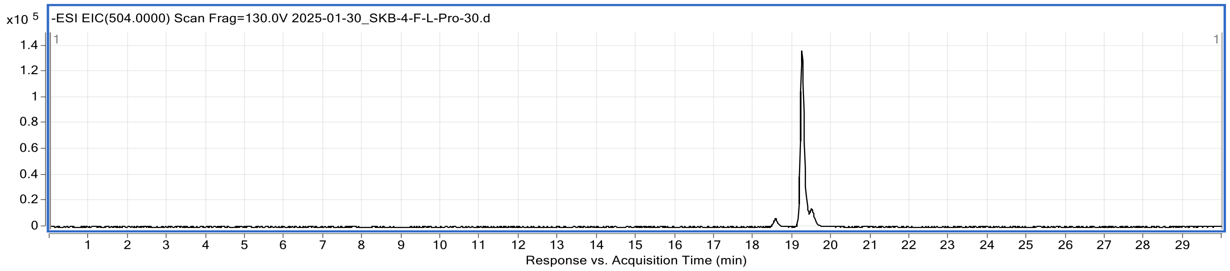

Zoomed and rescaled Extracted Ion (top) and Variable Wavelength Detector (bottom) Chromatograms. The Y axis on top is ion counts, the Y-axis on the bottom is absorbance units, and the X-axis for both is acquisition time in minutes. The peaks are labeled from left to right as Peak 1 and Peak 2.

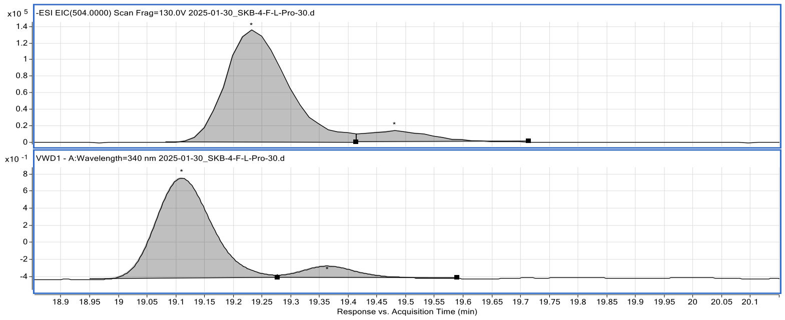

The following is a table of the peak data from the mass chromatogram. The Area % is the percent area relative to the tallest integrated peak, which has been set to 100%.

Peak	Assignment	t_R (min)	ES-ToF m/z [Neg]	Area %
1	L-*Threo*	19.231	504.1534	100
2	L-*Erythro*	19.480	504.1522	9.98

The calculated m/z for the adduct is 504.1536 for $C_{22}H_{23}FN_5O_8^-$ $[M - H]^{-1}$.

The following is a table of the peak data from the variable wavelength detector chromatogram (340 nm). The Area % is the percent area relative to the tallest integrated peak, which has been set to 100%.

Peak	Assignment	t_R (min)	Area %
1	L-*Threo*	19.110	100
2	L-*Erythro*	19.363	12.52

The following is a table showing the difference in retention time on the extracted ion chromatogram (i.e., m/z Δt_R) and variable wavelength detector (i.e., VWD Δt_R) between the two diastereomers (i.e., separation = absolute difference between L-*erythro* and L-*threo*). The Average Δt_R values are the average difference in retention time between the extracted ion and variable wavelength detector chromatograms.

	m/z Δt_R (min)	VWD Δt_R (min)	Average Δt_R (min)
Peak Separation	0.249	0.253	0.25

LC/MS Traces for the Attempted Resolution of the Beta Stereoisomers of the β-hydroxy-α-Amino Acid Corresponding to Adduct 26 with 1-Fluoro-2,4-dinitrophenyl-5-D-proline Amide (i.e., D-FDPA D-9) with HPLC Method A (i.e., 25 min.)

Total Ion Mass Chromatogram (ESI-ToF). The Y-axis is ion counts, and the X-axis is acquisition time in minutes.

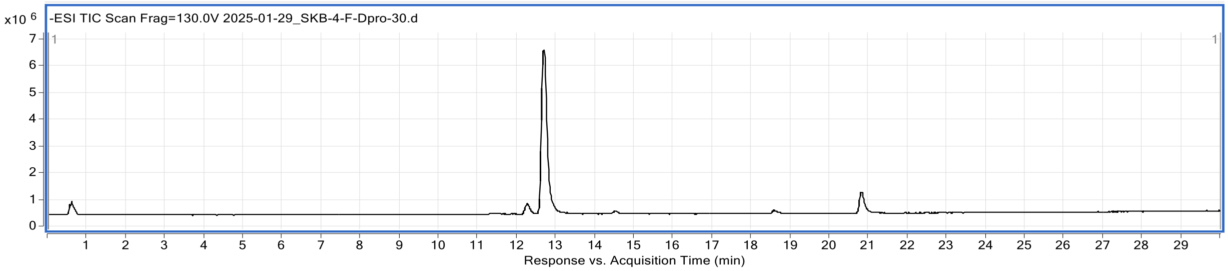

Variable Wavelength Detector Chromatogram (340 nm). The Y-axis is absorbance units, and the X-axis is acquisition time in minutes.

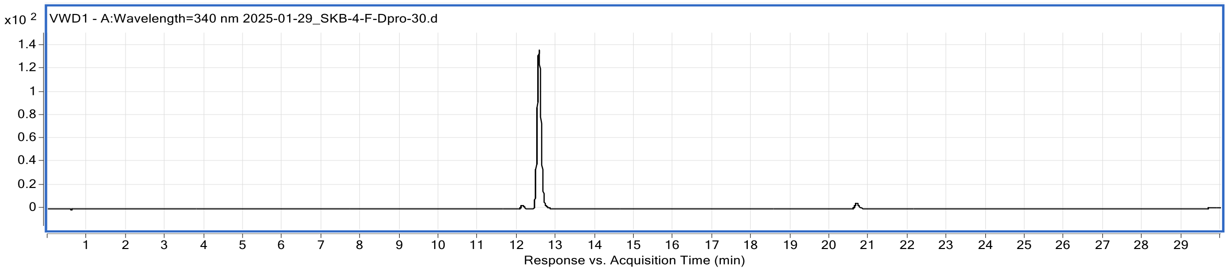

Extracted Ion Mass Chromatogram (ESI-ToF, extracted for m/z 504 ± 0.5). The Y-axis is ion counts, and the X-axis is acquisition time in minutes. Control experiments confirmed that the spurious peak at 18.7 min did not result from stereoisomer of the amino acid.

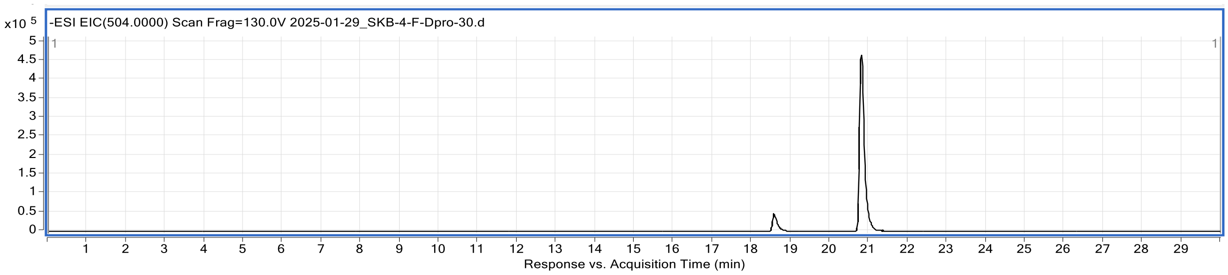

Zoomed and rescaled Extracted Ion (top) and Variable Wavelength Detector (bottom) Chromatograms. The Y axis on top is ion counts, the Y-axis on the bottom is absorbance units, and the X-axis for both is acquisition time in minutes. The minor adduct can be seen from the peak tailing, but the resolution is not sufficient to evaluate isomer separation.

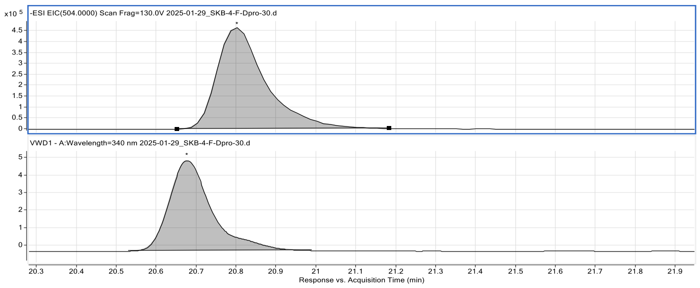

The following is a table of the peak data from the mass chromatogram. The Area % is the percent area relative to the tallest integrated peak, which has been set to 100%.

Peak	Assignment	t_R (min)	ES-ToF m/z [Neg]	Area %
1	L-*Threo*<br>L-*Erythro*	20.802	504.1559	100

The calculated m/z for the adduct is 504.1536 for $C_{22}H_{23}FN_5O_8^-$ $[M – H]^{-1}$.

The following is a table of the peak data from the variable wavelength detector chromatogram (340 nm). The Area % is the percent area relative to the tallest integrated peak, which has been set to 100%.

Peak	Assignment	t_R (min)	Area %
1	L-*Threo*<br>L-*Erythro*	20.677	100

The following is a table showing the difference in retention time on the extracted ion chromatogram (i.e., m/z Δt_R) and variable wavelength detector (i.e., VWD Δt_R) between the two diastereomers (i.e., separation = absolute difference between L-*erythro* and L-*threo*). The Average Δt_R values are the average difference in retention time between the extracted ion and variable wavelength detector chromatograms.

	m/z Δt_R (min)	VWD Δt_R (min)	Average Δt_R (min)
Peak Separation	0	0	0

LC/MS Traces for the Attempted Resolution of the Beta Stereoisomers of the β-hydroxy-α-Amino Acid Corresponding to Adduct 26 with 1-Fluoro-2,4-dinitrobenzene (i.e., Sanger's reagent 10) with HPLC Method A (i.e., 25 min.)

Total Ion Mass Chromatogram (ESI-ToF). The Y-axis is ion counts, and the X-axis is acquisition time in minutes.

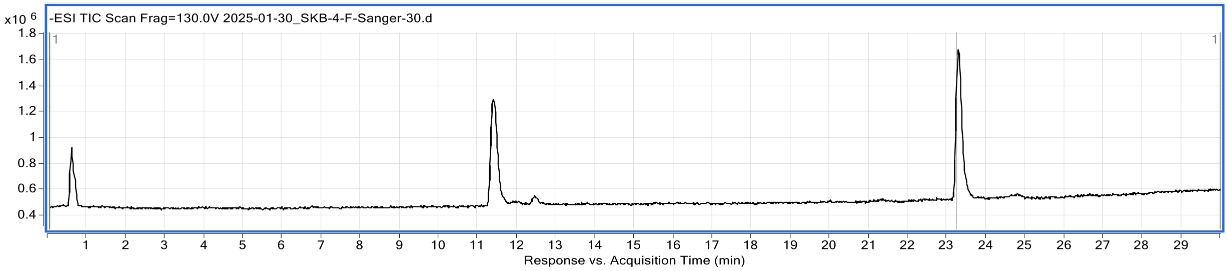

Variable Wavelength Detector Chromatogram (340 nm). The Y-axis is absorbance units, and the X-axis is acquisition time in minutes.

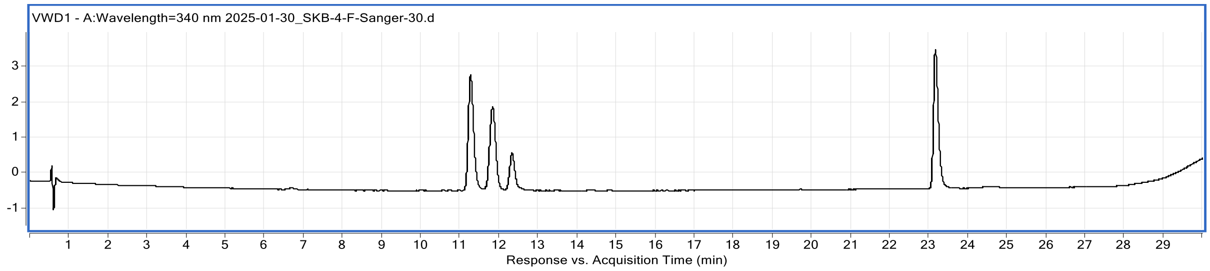

Extracted Ion Mass Chromatogram (ESI-ToF, extracted for m/z 392 ± 0.5). The Y-axis is ion counts, and the X-axis is acquisition time in minutes.

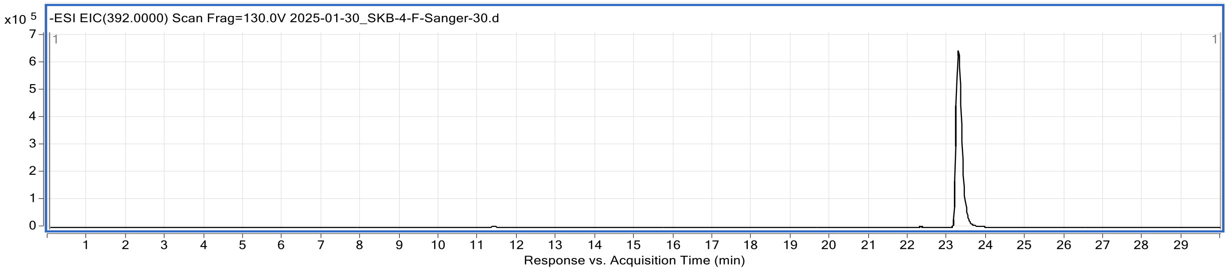

Zoomed and rescaled Extracted Ion (top) and Variable Wavelength Detector (bottom) Chromatograms. The Y axis on top is ion counts, the Y-axis on the bottom is absorbance units, and the X-axis for both is acquisition time in minutes. The minor adduct can be seen from the peak tailing, but the resolution is not sufficient to evaluate isomer separation.

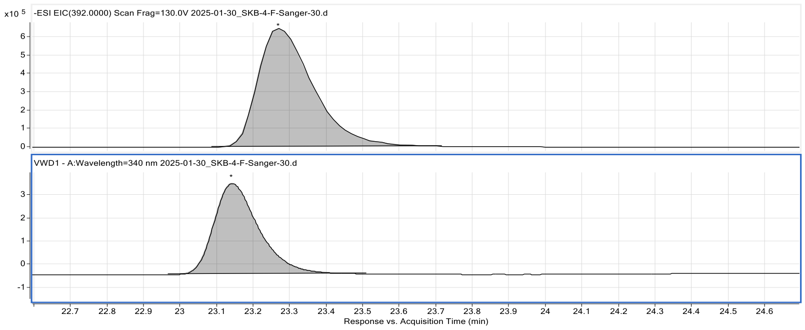

The following is a table of the peak data from the mass chromatogram. The Area % is the percent area relative to the tallest integrated peak, which has been set to 100%.

Peak	Assignment	t_R (min)	ES-ToF m/z [Neg]	Area %
1	L-*Threo*<br>L-*Erythro*	23.269	392.0928	100

The calculated m/z for the adduct is 392.0900 for $C_{17}H_{15}FN_3O_7^-$ $[M – H]^{-1}$.

The following is a table of the peak data from the variable wavelength detector chromatogram (340 nm). The Area % is the percent area relative to the tallest integrated peak, which has been set to 100%.

Peak	Assignment	t_R (min)	Area %
1	L-*Threo*<br>L-*Erythro*	23.140	100

The following is a table showing the difference in retention time on the extracted ion chromatogram (i.e., m/z Δt_R) and variable wavelength detector (i.e., VWD Δt_R) between the two diastereomers (i.e., separation = absolute difference between L-*erythro* and L-*threo*). The Average Δt_R values are the average difference in retention time between the extracted ion and variable wavelength detector chromatograms.

	m/z Δt_R (min)	VWD Δt_R (min)	Average Δt_R (min)
Peak Separation	0	0	0

LC/MS Traces for the Attempted Resolution of the Beta Stereoisomers of the β-hydroxy-α-Amino Acid Corresponding to Adduct 5 with 1-Fluoro-2,4-dinitrophenyl-5-L-alanine Amide (i.e., L-FDAA 7) with HPLC Method A (i.e., 25 min.)

Total Ion Mass Chromatogram (ESI-ToF). The Y-axis is ion counts, and the X-axis is acquisition time in minutes.

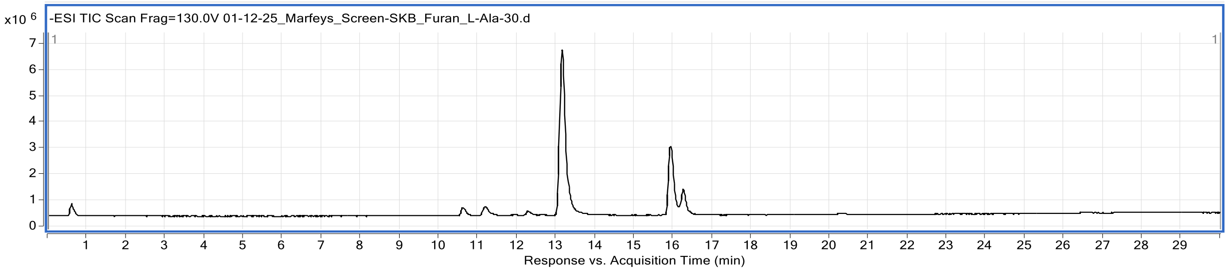

Variable Wavelength Detector Chromatogram (340 nm). The Y-axis is absorbance units, and the X-axis is acquisition time in minutes.

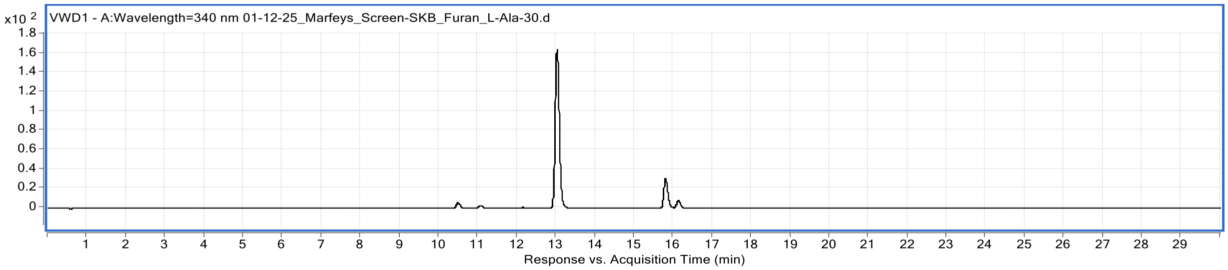

Extracted Ion Mass Chromatogram (ESI-ToF, extracted for m/z 450 ± 0.5). The Y-axis is ion counts, and the X-axis is acquisition time in minutes.

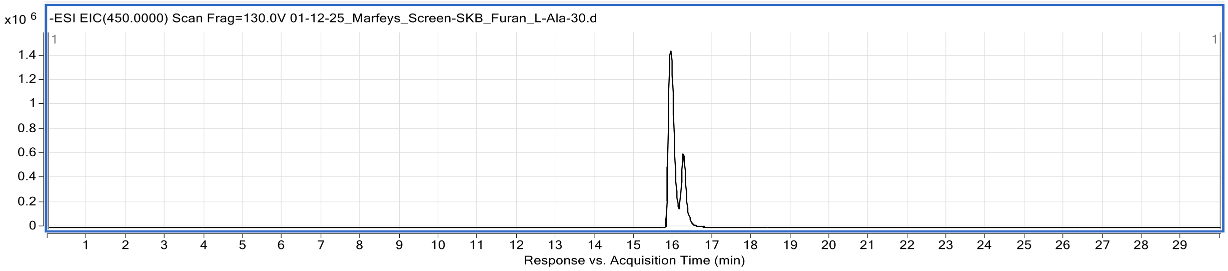

Zoomed and rescaled Extracted Ion (top) and Variable Wavelength Detector (bottom) Chromatograms. The Y axis on top is ion counts, the Y-axis on the bottom is absorbance units, and the X-axis for both is acquisition time in minutes. The peaks are labeled from left to right as Peak 1 and Peak 2.

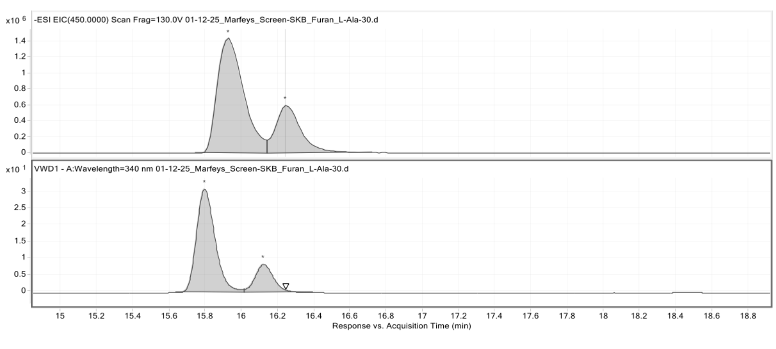

The following is a table of the peak data from the mass chromatogram. The Area % is the percent area relative to the tallest integrated peak, which has been set to 100%.

Peak	Assignment	t_R (min)	ES-ToF m/z [Neg]	Area %
1	L-*Threo*	15.927	450.1295	100
2	L-*Erythro*	16.243	450.1297	39.77

The calculated m/z for the adduct is 450.1267 for $C_{18}H_{20}N_5O_9^-$ $[M - H]^{-1}$.

The following is a table of the peak data from the variable wavelength detector chromatogram (340 nm). The Area % is the percent area relative to the tallest integrated peak, which has been set to 100%.

Peak	Assignment	t_R (min)	Area %
1	L-*Threo*	15.797	100
2	L-*Erythro*	16.120	27.84

The following is a table showing the difference in retention time on the extracted ion chromatogram (i.e., m/z Δt_R) and variable wavelength detector (i.e., VWD Δt_R) between the two diastereomers (i.e., separation = absolute difference between L-*erythro* and L-*threo*). The Average Δt_R values are the average difference in retention time between the extracted ion and variable wavelength detector chromatograms.

	m/z Δt_R (min)	VWD Δt_R (min)	Average Δt_R (min)
Peak Separation	0.316	0.323	0.32

LC/MS Traces for the Attempted Resolution of the Beta Stereoisomers of the β-hydroxy-α-Amino Acid Corresponding to Adduct 5 with 1-Fluoro-2,4-dinitrophenyl-5-D-alanine Amide (i.e., D-FDAA D-7) with HPLC Method A (i.e., 25 min.)

Total Ion Mass Chromatogram (ESI-ToF). The Y-axis is ion counts, and the X-axis is acquisition time in minutes.

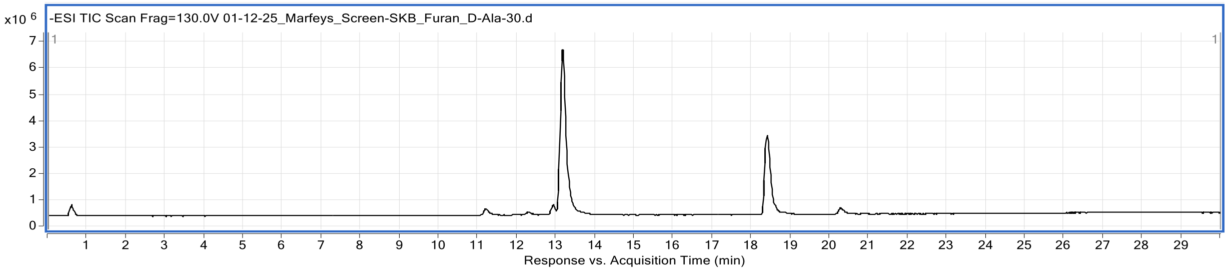

Variable Wavelength Detector Chromatogram (340 nm). The Y-axis is absorbance units, and the X-axis is acquisition time in minutes.

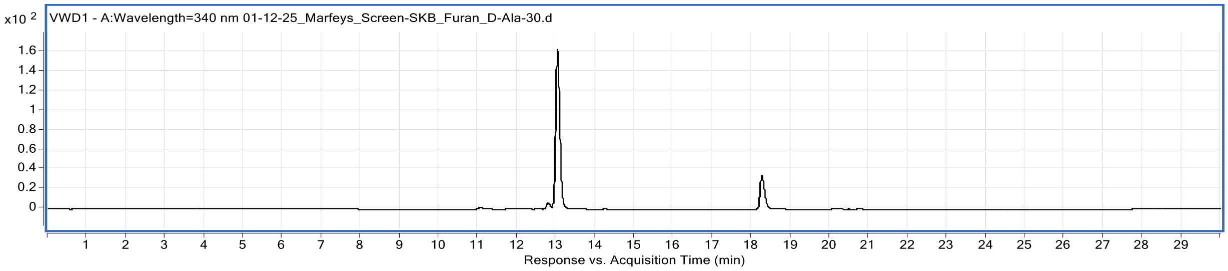

Extracted Ion Mass Chromatogram (ESI-ToF, extracted for m/z 450 ± 0.5). The Y-axis is ion counts, and the X-axis is acquisition time in minutes.

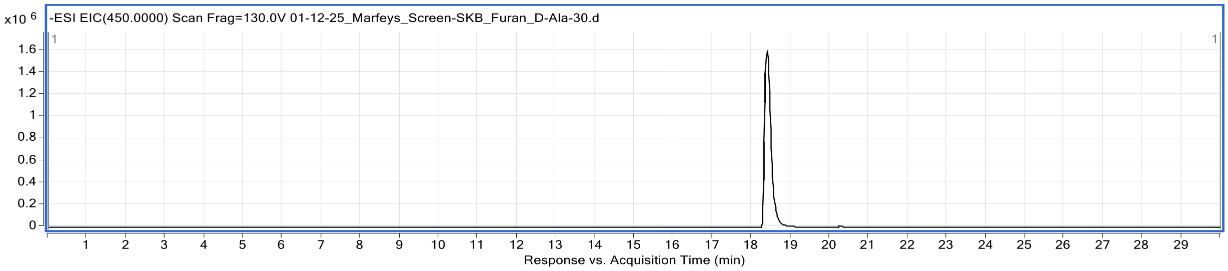

Zoomed and rescaled Extracted Ion (top) and Variable Wavelength Detector (bottom) Chromatograms. The Y axis on top is ion counts, the Y-axis on the bottom is absorbance units, and the X-axis for both is acquisition time in minutes. The minor adduct can be seen from the peak tailing, but the resolution is not sufficient to evaluate isomer separation.

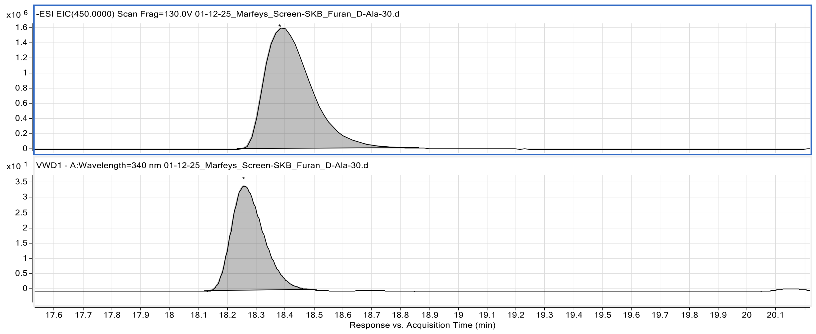

The following is a table of the peak data from the mass chromatogram. The Area % is the percent area relative to the tallest integrated peak, which has been set to 100%.

Peak	Assignment	t_R (min)	ES-ToF m/z [Neg]	Area %
1	L-*Threo*/ L-*Erythro*	18.382	450.1286	100

The calculated m/z for the adduct is 450.1267 for $C_{18}H_{20}N_5O_9^-$ $[M - H]^{-1}$.

The following is a table of the peak data from the variable wavelength detector chromatogram (340 nm). The Area % is the percent area relative to the tallest integrated peak, which has been set to 100%.

Peak	Assignment	t_R (min)	Area %
1	L-*Threo*/ L-*Erythro*	18.257	100

The following is a table showing the difference in retention time on the extracted ion chromatogram (i.e., m/z Δt_R) and variable wavelength detector (i.e., VWD Δt_R) between the two diastereomers (i.e., separation = absolute difference between L-*erythro* and L-*threo*). The Average Δt_R values are the average difference in retention time between the extracted ion and variable wavelength detector chromatograms.

	m/z Δt_R (min)	VWD Δt_R (min)	Average Δt_R (min)
Peak Separation	0	0	0

LC/MS Traces for the Attempted Resolution of the Beta Stereoisomers of the β-hydroxy-α-Amino Acid Corresponding to Adduct 5 with 1-Fluoro-2,4-dinitrophenyl-5-L-proline Amide (i.e., L-FDPA 9) with HPLC Method A (i.e., 25 min.)

Total Ion Mass Chromatogram (ESI-ToF). The Y-axis is ion counts, and the X-axis is acquisition time in minutes.

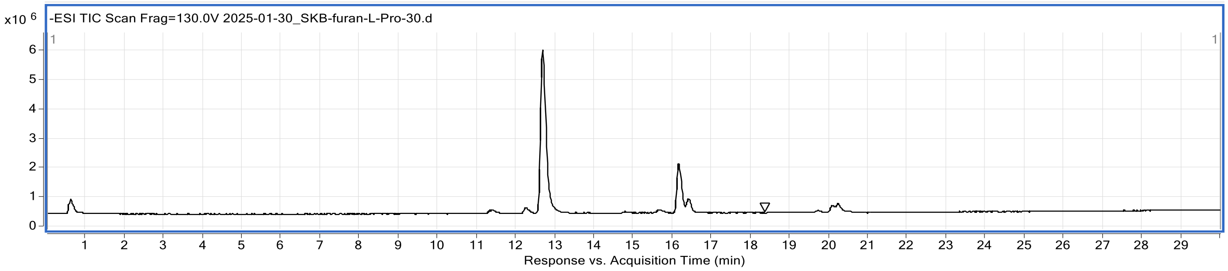

Variable Wavelength Detector Chromatogram (340 nm). The Y-axis is absorbance units, and the X-axis is acquisition time in minutes.

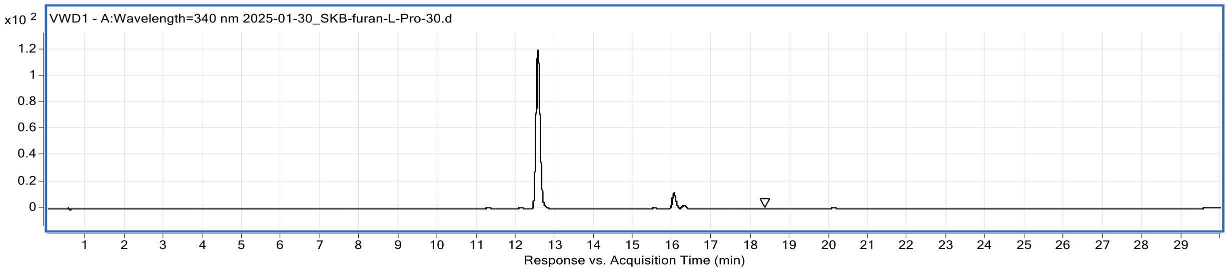

Extracted Ion Mass Chromatogram (ESI-ToF, extracted for m/z 476 ± 0.5). The Y-axis is ion counts, and the X-axis is acquisition time in minutes.

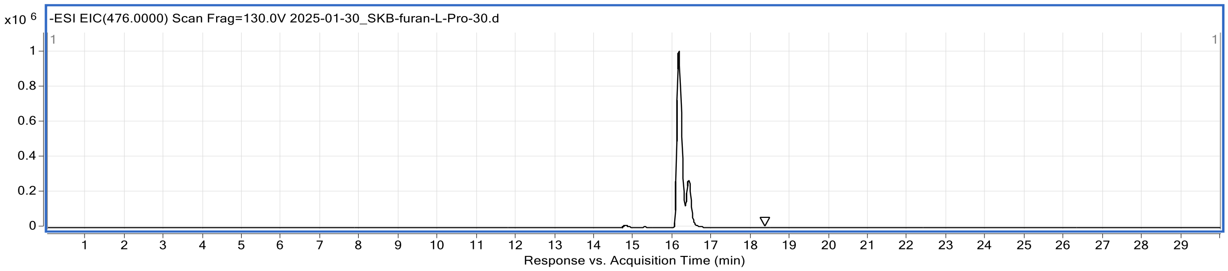

Zoomed and rescaled Extracted Ion (top) and Variable Wavelength Detector (bottom) Chromatograms. The Y axis on top is ion counts, the Y-axis on the bottom is absorbance units, and the X-axis for both is acquisition time in minutes. The peaks are labeled from left to right as Peak 1 and Peak 2.

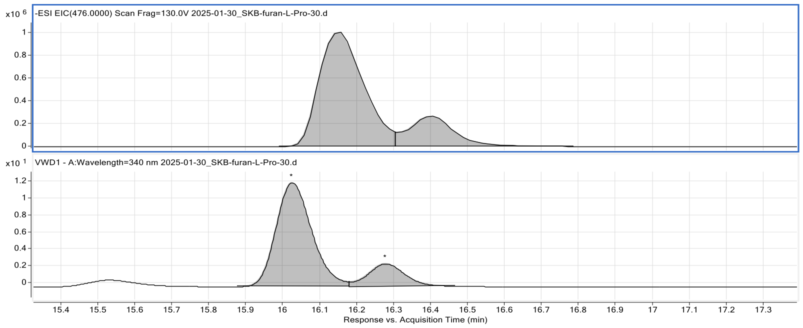

The following is a table of the peak data from the mass chromatogram. The Area % is the percent area relative to the tallest integrated peak, which has been set to 100%.

Peak	Assignment	t_R (min)	ES-ToF m/z [Neg]	Area %
1	L-*Threo*	16.156	478.1447	100
2	L-*Erythro*	16.404	478.1445	27.71

The calculated m/z for the adduct is 476.1423 for $C_{20}H_{22}NO_9^-$ $[M - H]^{-1}$.

The following is a table of the peak data from the variable wavelength detector chromatogram (340 nm). The Area % is the percent area relative to the tallest integrated peak, which has been set to 100%.

Peak	Assignment	t_R (min)	Area %
1	L-*Threo*	16.023	100
2	L-*Erythro*	16.277	23.68

The following is a table showing the difference in retention time on the extracted ion chromatogram (i.e., m/z Δt_R) and variable wavelength detector (i.e., VWD Δt_R) between the two diastereomers (i.e., separation = absolute difference between L-*erythro* and L-*threo*). The Average Δt_R values are the average difference in retention time between the extracted ion and variable wavelength detector chromatograms.

	m/z Δt_R (min)	VWD Δt_R (min)	Average Δt_R (min)
Peak Separation	0.248	0.254	0.25

LC/MS Traces for the Attempted Resolution of the Beta Stereoisomers of the β-hydroxy-α-Amino Acid Corresponding to Adduct 5 with 1-Fluoro-2,4-dinitrophenyl-5-D-proline Amide (i.e., D-FDPA D-9) with HPLC Method A (i.e., 25 min.)

Total Ion Mass Chromatogram (ESI-ToF). The Y-axis is ion counts, and the X-axis is acquisition time in minutes.

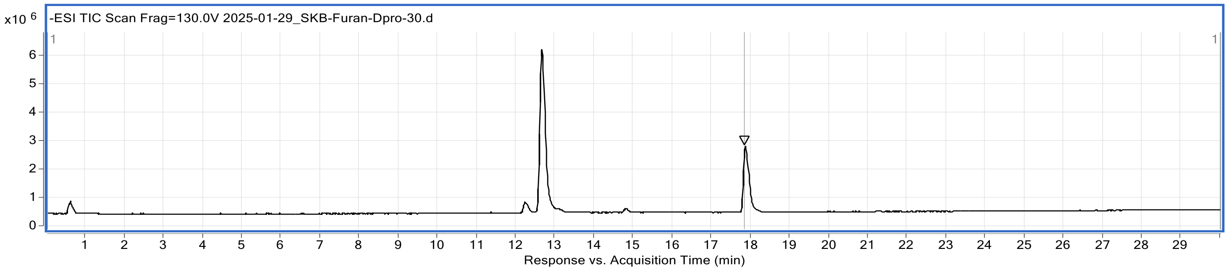

Variable Wavelength Detector Chromatogram (340 nm). The Y-axis is absorbance units, and the X-axis is acquisition time in minutes.

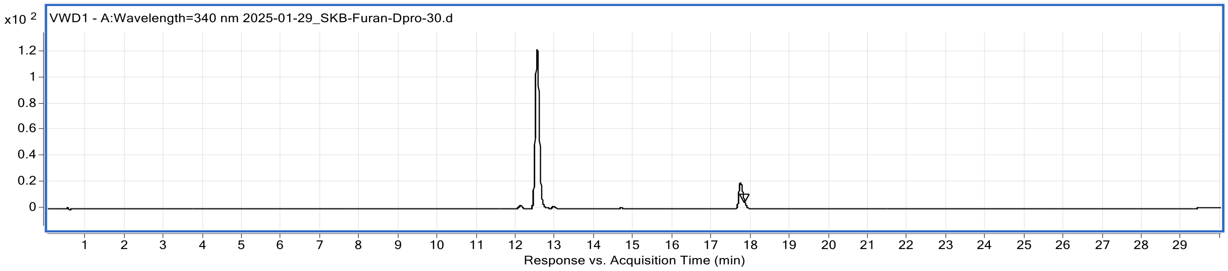

Extracted Ion Mass Chromatogram (ESI-ToF, extracted for m/z 476 ± 0.5). The Y-axis is ion counts, and the X-axis is acquisition time in minutes. Control experiments confirmed that the spurious peak at 14.9 min did not result from stereoisomer of the amino acid.

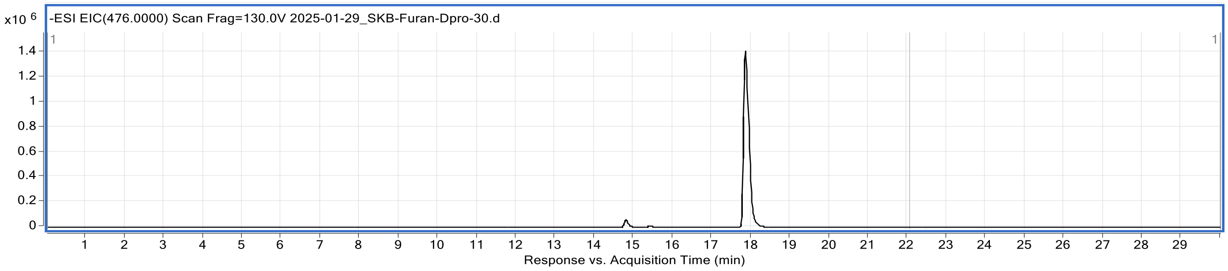

Zoomed and rescaled Extracted Ion (top) and Variable Wavelength Detector (bottom) Chromatograms. The Y axis on top is ion counts, the Y-axis on the bottom is absorbance units, and the X-axis for both is acquisition time in minutes. The minor adduct can be seen from the peak tailing, but the resolution is not sufficient to evaluate isomer separation.

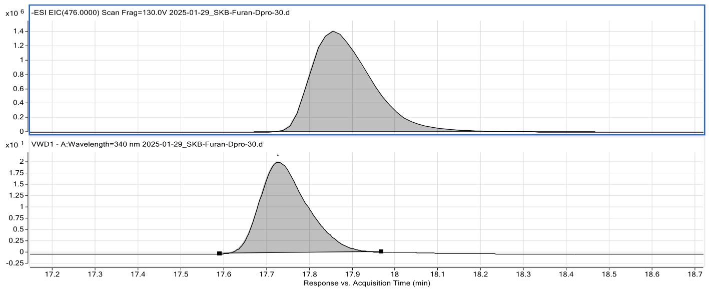

The following is a table of the peak data from the mass chromatogram. The Area % is the percent area relative to the tallest integrated peak, which has been set to 100%.

Peak	Assignment	t_R (min)	ES-ToF m/z [Neg]	Area %
1	L-*Threo/* L-*Erythro*	17.853	476.1433	100

The calculated m/z for the adduct is 476.1423 for $C_{20}H_{22}NO_9^-$ $[M – H]^{-1}$.

The following is a table of the peak data from the variable wavelength detector chromatogram (340 nm). The Area % is the percent area relative to the tallest integrated peak, which has been set to 100%.

Peak	Assignment	t_R (min)	Area %
1	L-*Threo/* L-*Erythro*	17.727	100

The following is a table showing the difference in retention time on the extracted ion chromatogram (i.e., m/z Δt_R) and variable wavelength detector (i.e., VWD Δt_R) between the two diastereomers (i.e., separation = absolute difference between L-*erythro* and L-*threo*). The Average Δt_R values are the average difference in retention time between the extracted ion and variable wavelength detector chromatograms.

	m/z Δt_R (min)	VWD Δt_R (min)	Average Δt_R (min)
Peak Separation	0	0	0

LC/MS Traces for the Attempted Resolution of the Beta Stereoisomers of the β-hydroxy-α-Amino Acid Corresponding to Adduct 5 with 1-Fluoro-2,4-dinitrobenzene (i.e., Sanger's reagent 10) with HPLC Method A (i.e., 25 min.)

Total Ion Mass Chromatogram (ESI-ToF). The Y-axis is ion counts, and the X-axis is acquisition time in minutes.

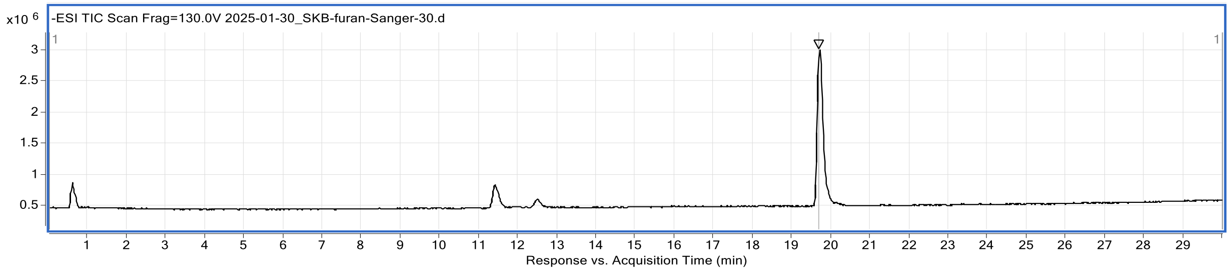

Variable Wavelength Detector Chromatogram (340 nm). The Y-axis is absorbance units, and the X-axis is acquisition time in minutes.

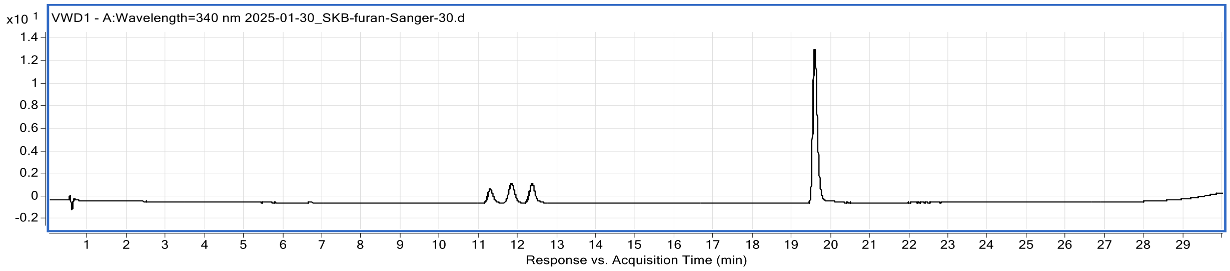

Extracted Ion Mass Chromatogram (ESI-ToF, extracted for m/z 364 ± 0.5). The Y-axis is ion counts, and the X-axis is acquisition time in minutes.

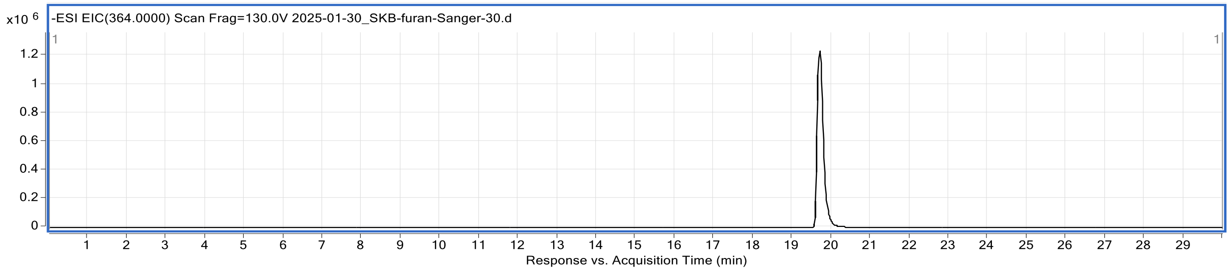

Zoomed and rescaled Extracted Ion (top) and Variable Wavelength Detector (bottom) Chromatograms. The Y axis on top is ion counts, the Y-axis on the bottom is absorbance units, and the X-axis for both is acquisition time in minutes. The minor adduct can be seen from the peak tailing, but the resolution is not sufficient to evaluate isomer separation.

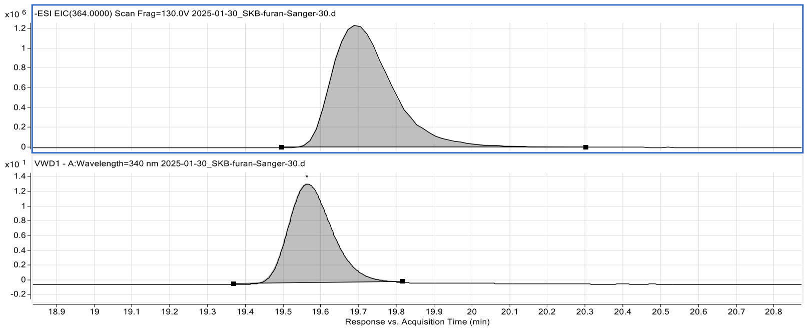

The following is a table of the peak data from the mass chromatogram. The Area % is the percent area relative to the tallest integrated peak, which has been set to 100%.

Peak	Assignment	t_R (min)	ES-ToF m/z [Neg]	Area %
1	L-*Threo/*<br>L-*Erythro*	19.687	364.0811	100

The calculated m/z for the adduct is 364.0786 for $C_{15}H_{14}N_3O_8^-$ $[M - H]^{-1}$.

The following is a table of the peak data from the variable wavelength detector chromatogram (340 nm). The Area % is the percent area relative to the tallest integrated peak, which has been set to 100%.

Peak	Assignment	t_R (min)	Area %
1	L-*Threo/*<br>L-*Erythro*	19.563	100

The following is a table showing the difference in retention time on the extracted ion chromatogram (i.e., m/z Δt_R) and variable wavelength detector (i.e., VWD Δt_R) between the two diastereomers (i.e., separation = absolute difference between L-*erythro* and L-*threo*). The Average Δt_R values are the average difference in retention time between the extracted ion and variable wavelength detector chromatograms.

	m/z Δt_R (min)	VWD Δt_R (min)	Average Δt_R (min)
Peak Separation	0	0	0

LC/MS Traces for the Attempted Resolution of the Phenylserine Diastereomers After Derivatization with (*S*)-1-(5-Fluoro-2,4-dinitrophenyl)piperidine-2-carboxamide (i.e., 28, Figure 4) Using HPLC Method A (i.e., 25-minute gradient)

Total Ion Mass Chromatogram (ESI-ToF). The Y-axis is ion counts, and the X-axis is acquisition time in minutes.

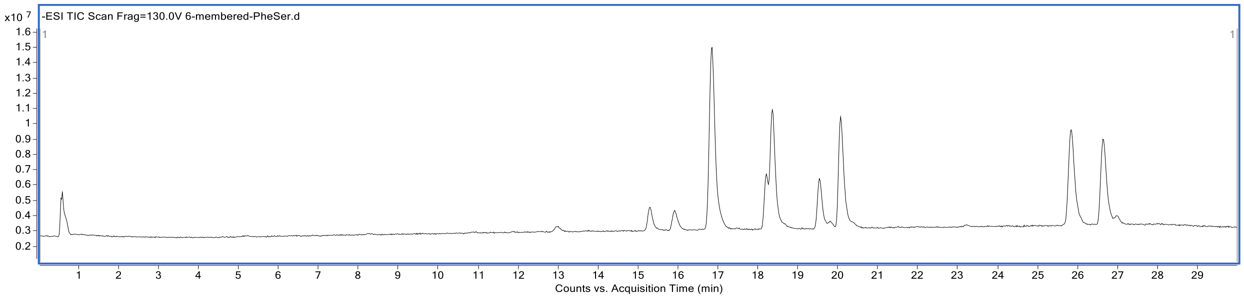

Variable Wavelength Detector Chromatogram (340 nm). The Y-axis is absorbance units, and the X-axis is acquisition time in minutes.

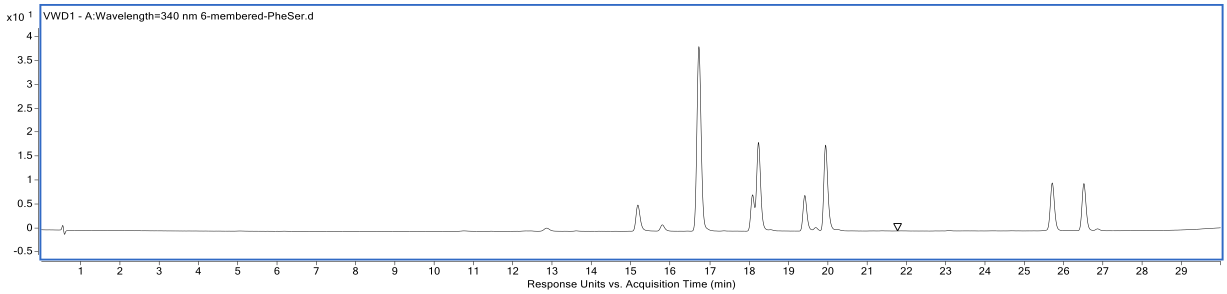

Extracted Ion Mass Chromatogram (ESI-ToF, extracted for m/z 472 ± 0.5). The Y-axis is ion counts, and the X-axis is acquisition time in minutes.

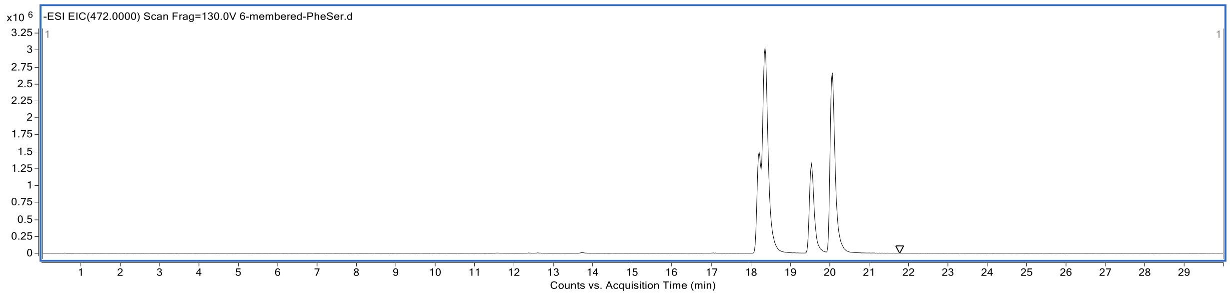

Zoomed and rescaled Extracted Ion (top) and Variable Wavelength Detector (bottom) Chromatograms. The Y axis on top is ion counts, the Y-axis on the bottom is absorbance units, and the X-axis for both is acquisition time in minutes. The peaks are labeled from left to right as Peak 1, Peak 2, Peak 3, and Peak 4.

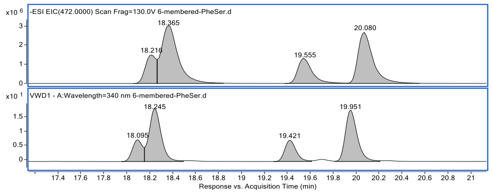

The following is a table of the peak data from the mass chromatogram. The Area % is the percent area relative to the tallest integrated peak, which has been set to 100%.

Peak	Assignment	t_R (min)	ES-ToF m/z [Neg]	Area %
1	L-*Erythro*	18.216	472.1473	49.18%
2	L-*Threo*	18.365	472.1465	100
3	D-*Erythro*	19.555	472.1465	41.09%
4	D-*Threo*	20.080	472.1467	87.93%

The calculated m/z for the adduct of phenylserine with piperidine-containing **28** is 472.1474 for $C_{21}H_{22}N_5O_8^-$ $[M-H]^{-1}$.

The following is a table of the peak data from the variable wavelength detector chromatogram (340 nm). The Area % is the percent area relative to the tallest integrated peak, which has been set to 100%.

Peak	Assignment	t_R (min)	Area %
1	L-*Erythro*	18.095	34.65%
2	L-*Threo*	18.245	100%
3	D-*Erythro*	19.421	38.88%
4	D-*Threo*	19.951	97.97%

The following is a table showing the difference in retention time on the extracted ion chromatogram (i.e., m/z Δt_R) and variable wavelength detector (i.e., VWD Δt_R) between the *threo* diastereomers (i.e., *Threo* separation = absolute difference between L-*threo* and D-*threo*), the *erythro* diastereomers (i.e., *Erythro* separation = absolute difference between L-*erythro* and D-*erythro*), alpha-L-diastereomers (i.e., L-separation = absolute difference between L-*threo* and L-*erythro*), and the alpha-D-diastereomers (i.e., D-separation = absolute difference between D-*threo* and D-*erythro*). The Average Δt_R values are the average difference in retention time between the extracted ion and variable wavelength detector chromatograms.

	m/z Δt_R (min)	VWD Δt_R (min)	Average Δt_R (min)
Threo Separation	1.715	1.706	1.71
Erythro Separation	1.339	1.326	1.33
L-Separation	0.149	0.150	0.15
D-Separation	0.525	0.530	0.53

LC/MS Traces for the Attempted Resolution of the Phenylserine Diastereomers After Derivatization with (*S*)-1-(5-Fluoro-2,4-dinitrophenyl)azetidine-2-carboxamide (i.e., 29, Figure 4) Using HPLC Method A (i.e., 25-minute gradient)

Total Ion Mass Chromatogram (ESI-ToF). The Y-axis is ion counts, and the X-axis is acquisition time in minutes.

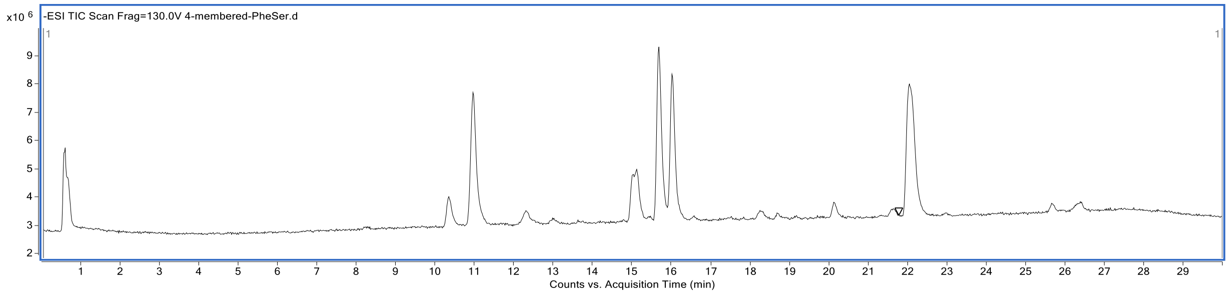

Variable Wavelength Detector Chromatogram (340 nm). The Y-axis is absorbance units, and the X-axis is acquisition time in minutes.

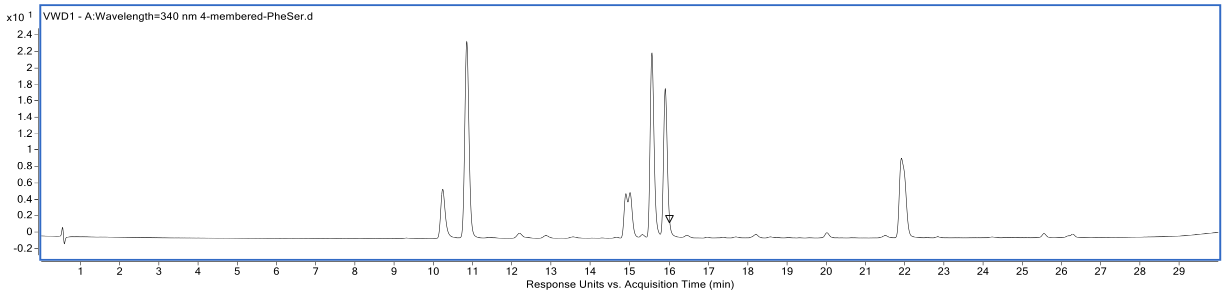

Extracted Ion Mass Chromatogram (ESI-ToF, extracted for m/z 444 ± 0.5). The Y-axis is ion counts, and the X-axis is acquisition time in minutes.

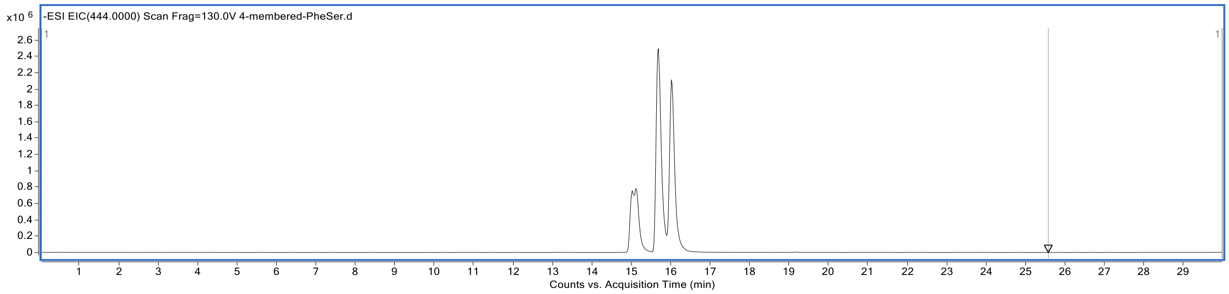

Zoomed and rescaled Extracted Ion (top) and Variable Wavelength Detector (bottom) Chromatograms. The Y axis on top is ion counts, the Y-axis on the bottom is absorbance units, and the X-axis for both is acquisition time in minutes. The peaks are labeled from left to right as Peak 1, Peak 2, Peak 3, and Peak 4.

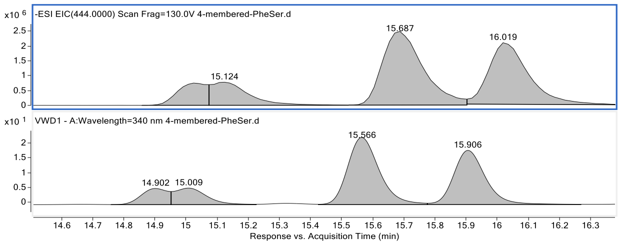

The following is a table of the peak data from the mass chromatogram. The Area % is the percent area relative to the tallest integrated peak, which has been set to 100%. The peak assignments were determined by comparing t_R values against an authentic standard of L-*erythro* and L-*threo* **3** after derivatizing with Marfey's reagent **29**.

Peak	Assignment	t_R (min)	ES-ToF m/z [Neg]	Area %
1	L-*Erythro*	15.025	444.1156	21.62%
2	D-*Erythro*	15.124	444.1158	28.74%
3	L-*Threo*	15.687	444.1152	100.00%
4	D-*Threo*	16.019	444.1149	82.40%

The calculated m/z for the adduct of phenylserine with azetidine-containing **29** is 444.1161 for $C_{19}H_{18}N_5O_8^-$ $[M - H]^{-1}$.

The following is a table of the peak data from the variable wavelength detector chromatogram (340 nm). The Area % is the percent area relative to the tallest integrated peak, which has been set to 100%.

Peak	Assignment	t_R (min)	Area %
1	L-*Erythro*	14.902	20.28%
2	D-*Erythro*	15.009	24.97%
3	L-*Threo*	15.566	100.00%
4	D-*Threo*	15.906	81.38%

The following is a table showing the difference in retention time on the extracted ion chromatogram (i.e., m/z Δt_R) and variable wavelength detector (i.e., VWD Δt_R) between the *threo* diastereomers (i.e., *Threo* separation = absolute difference between L-*threo* and D-*threo*), the *erythro* diastereomers (i.e., *Erythro* separation = absolute difference between L-*erythro* and D-*erythro*), alpha-L-diastereomers (i.e., L-separation = absolute difference between L-*threo* and L-*erythro*), and the alpha-D-diastereomers (i.e., D-separation = absolute difference between D-*threo* and D-*erythro*). The Average Δt_R values are the average difference in retention time between the extracted ion and variable wavelength detector chromatograms.

	m/z Δt_R (min)	VWD Δt_R (min)	Average Δt_R (min)
Threo Separation	0.332	0.340	0.336
Erythro Separation	0.100	0.107	0.103
L-Separation	0.662	0.664	0.663
D-Separation	0.895	0.897	0.896

III-D. Mixed Marfey's Reaction for the Multiplexed Method Scouting of Different Chiral Derivatizing Agents in the Resolution of the Four Stereoisomers of Phenylserine

A mixture of the four stereoisomers (30 µL, 10 mM in amino acid) was diluted in aq. $NaHCO_3$ (120 µL, 15 mM), and a solution of several Marfey's reagents (150 µL, 1.25 mM of Marfey's reagents **7**, **9**, **15**–**19** along with 1.25 mM Sanger's reagent **10**) was added. The final concentrations were 1 mM for the amino acids and 5 mM for the derivatizing agents. The resulting mixture was vortexed briefly and incubated at 37 ºC. After 20 h, the mixture was diluted with acetonitrile (150 µL) and aq. HCl (150 µL, 60 mM) and vortexed briefly. The resulting mixture was diluted 5-fold in 1:1 MeCN:H_2O (i.e., 200 µL of mixture diluted in 800 µL of 1:1 MeCN:H_2O) and analyzed by HPLC-MS using HPLC Method A.

LC/MS Traces for the Attempted Resolution of the Four Diastereomers of Phenylserine with a Mixture of Seven Marfey's Reagents and Sanger's Reagent (i.e., Mixed Marfey's Reaction) with HPLC Method A (i.e., 25 min.)

Total Ion Mass Chromatogram (ESI-ToF). The Y-axis is ion counts, and the X-axis is acquisition time in minutes.

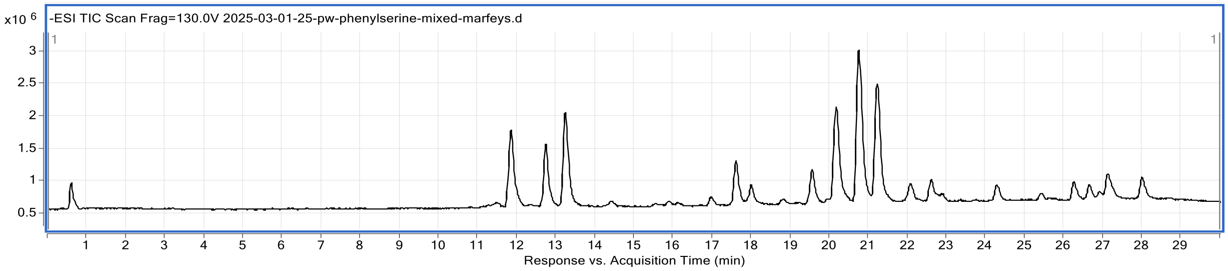

Variable Wavelength Detector Chromatogram (340 nm). The Y-axis is absorbance units, and the X-axis is acquisition time in minutes.

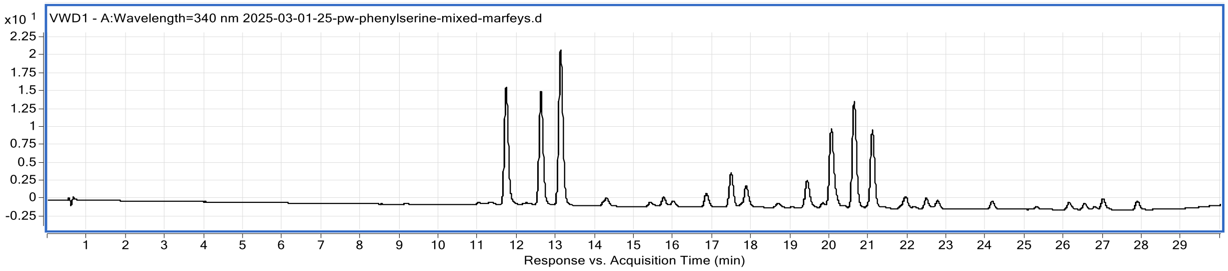

Extracted Ion Mass Spectrometry Data for the L-FDAA (i.e., 7) Adducts of Phenylserine from the Mixed Marfey's Derivatization Experiment

Extracted Ion Mass Chromatogram (ESI-ToF, extracted for m/z 432.1161 ± 0.005) for the adducts of Marfey's Reagent **7**. The Y-axis is ion counts, and the X-axis is acquisition time in minutes.

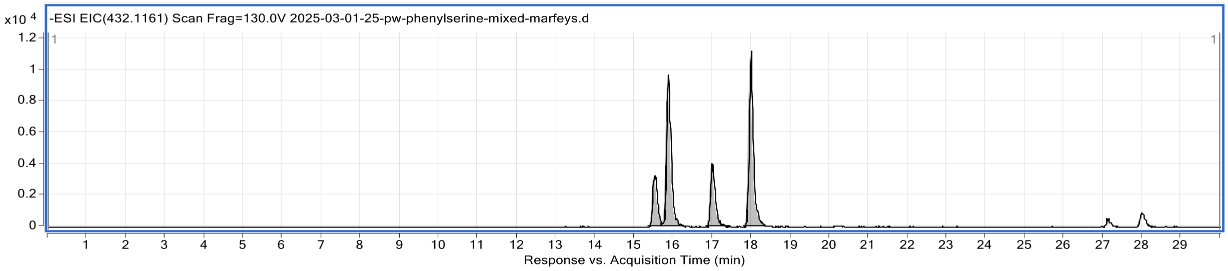

The following is a table of the peak data from the mass chromatogram. The Area % is the percent area relative to the tallest integrated peak, which has been set to 100%. The ΔArea is the absolute difference in percent area between the single and mixed Marfey's experiments.

Peak	Assignment	t_R (min)	Area (%)	ΔArea (%)
1	L-*Erythro*	15.529	32.67	3.3
2	L-*Threo*	15.878	93.38	0.9
3	D-*Erythro*	16.990	40.26	10.6
4	D-*Threo*	17.986	100	0

The following is a table showing the variation in Δt_R between the single Marfey's (cf. Section III-B) and mixed Marfey's derivatization reactions. Shown is the difference in retention time on the extracted ion chromatogram (i.e., m/z Δt_R) between the *threo* diastereomers (i.e., *Threo* separation = absolute difference between L-*threo* and D-*threo*), the *erythro* diastereomers (i.e., *Erythro* separation = absolute difference between L-*erythro* and D-*erythro*), alpha-L-diastereomers (i.e., L-separation = absolute difference between L-*threo* and L-*erythro*), and the alpha-D-diastereomers (i.e., D-separation = absolute difference between D-*threo* and D-*erythro*). The change in Δt_R values are the absolute differences between the single Marfey's and mixed Marfey's reactions.

	Single Marfey's m/z Δt_R (min)	Mixed Marfey's m/z Δt_R (min)	Change in Δt_R (min)
Threo Separation	2.108	2.108	0.00
Erythro Separation	1.478	1.461	0.02
L-Separation	0.332	0.349	0.02
D-Separation	0.962	0.996	0.03

Extracted Ion Mass Spectrometry Data for the L-FDVA (i.e., 15) Adducts of Phenylserine from the Mixed Marfey's Derivatization Experiment

Extracted Ion Mass Chromatogram (ESI-ToF, extracted for m/z 460.1474 ± 0.005) for the adducts of Marfey's Reagent **15**. The Y-axis is ion counts, and the X-axis is acquisition time in minutes.

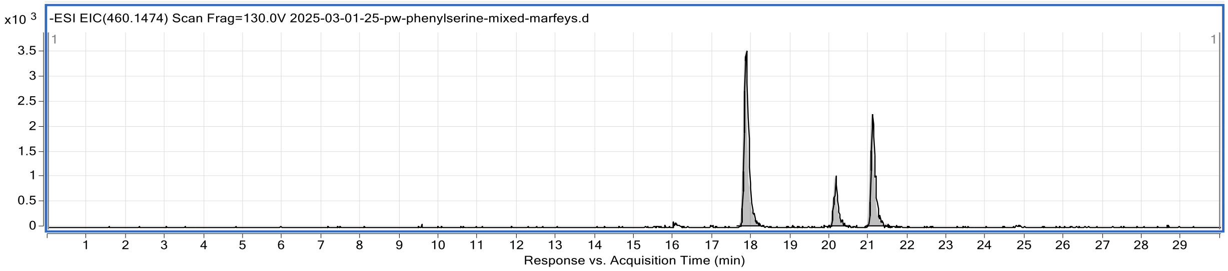

The following is a table of the peak data from the mass chromatogram. The Area % is the percent area relative to the tallest integrated peak, which has been set to 100%. The Area % is the percent area relative to the tallest integrated peak, which has been set to 100%. The ΔArea is the absolute difference in percent area between the single and mixed Marfey's experiments.

Peak	Assignment	t_R (min)	Area (%)	ΔArea (%)
1	L-*Erythro*/ L-*Threo*	17.870	100	0
2	D-*Erythro*	20.143	24.66	1.97
3	D-*Threo*	21.090	60.41	21.98

The following is a table showing the variation in Δt_R between the single Marfey's (cf. Section III-B) and mixed Marfey's derivatization reactions. Shown is the difference in retention time on the extracted ion chromatogram (i.e., m/z Δt_R) between the *threo* diastereomers (i.e., *Threo* separation = absolute difference between L-*threo* and D-*threo*), the *erythro* diastereomers (i.e., *Erythro* separation = absolute difference between L-*erythro* and D-*erythro*), alpha-L-diastereomers (i.e., L-separation = absolute difference between L-*threo* and L-*erythro*), and the alpha-D-diastereomers (i.e., D-separation = absolute difference between D-*threo* and D-*erythro*). The change in Δt_R values are the absolute differences between the single Marfey's and mixed Marfey's reactions.

	Single Marfey's m/z Δt_R (min)	Mixed Marfey's m/z Δt_R (min)	Change in Δt_R (min)
Threo Separation	3.220	3.220	0.00
Erythro Separation	2.274	2.273	0.00
L-Separation	0	0	0
D-Separation	0.946	0.947	0.00

Extracted Ion Mass Spectrometry Data for the L-FDIA (i.e., 16) Adducts of Phenylserine from the Mixed Marfey's Derivatization Experiment

Extracted Ion Mass Chromatogram (ESI-ToF, extracted for m/z 474.1630 ± 0.005) for the adducts of Marfey's Reagent **16**. The Y-axis is ion counts, and the X-axis is acquisition time in minutes.

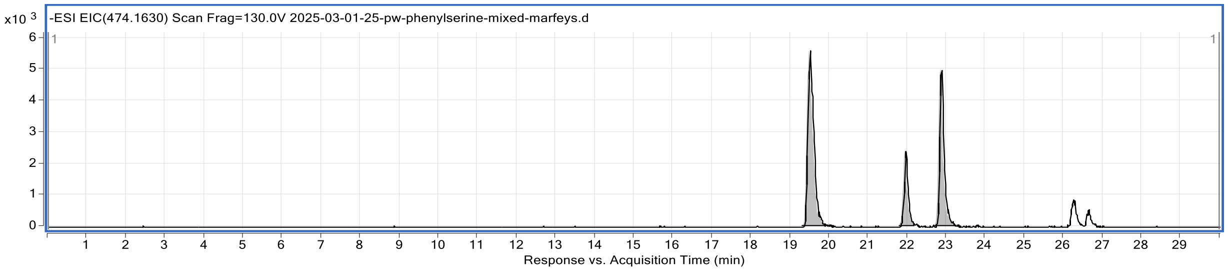

The following is a table of the peak data from the mass chromatogram. The Area % is the percent area relative to the tallest integrated peak, which has been set to 100%. The ΔArea is the absolute difference in percent area between the single and mixed Marfey's experiments.

Peak	Assignment	t_R (min)	Area (%)	ΔArea (%)
1	L-*Erythro*/ L-*Threo*	19.496	100	0.0
2	D-*Erythro*	21.953	28.22	8.2
3	D-*Threo*	22.866	68.69	4.3

The following is a table showing the variation in Δt_R between the single Marfey's (cf. Section III-B) and mixed Marfey's derivatization reactions. Shown is the difference in retention time on the extracted ion chromatogram (i.e., m/z Δt_R) between the *threo* diastereomers (i.e., *Threo* separation = absolute difference between L-*threo* and D-*threo*), the *erythro* diastereomers (i.e., *Erythro* separation = absolute difference between L-*erythro* and D-*erythro*), alpha-L-diastereomers (i.e., L-separation = absolute difference between L-*threo* and L-*erythro*), and the alpha-D-diastereomers (i.e., D-separation = absolute difference between D-*threo* and D-*erythro*). The change in Δt_R values are the absolute differences between the single Marfey's and mixed Marfey's reactions.

	Single Marfey's m/z Δt_R (min)	Mixed Marfey's m/z Δt_R (min)	Change in Δt_R (min)
Threo Separation	3.353	3.370	0.02
Erythro Separation	2.457	2.457	0.00
L-Separation	0	0	0
D-Separation	0.896	0.913	0.02

Extracted Ion Mass Spectrometry Data for the L-FDTA (i.e., 17) Adducts of Phenylserine from the Mixed Marfey's Derivatization Experiment

Extracted Ion Mass Chromatogram (ESI-ToF, extracted for m/z 462.1267 ± 0.005) for the adducts of Marfey's Reagent **17**. The Y-axis is ion counts, and the X-axis is acquisition time in minutes.

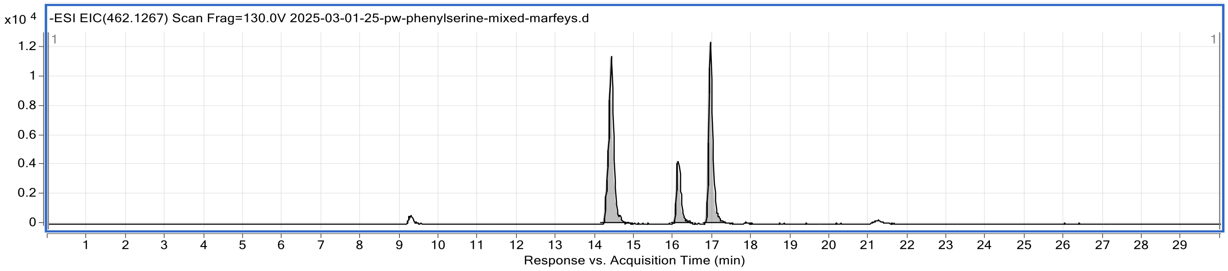

The following is a table of the peak data from the mass chromatogram. The Area % is the percent area relative to the tallest integrated peak, which has been set to 100%. The ΔArea is the absolute difference in percent area between the single and mixed Marfey's experiments.

Peak	Assignment	t_R (min)	Area (%)	ΔArea (%)
1	L-*Erythro*/ L-*Threo*	14.401	100	0.0
2	D-*Erythro*	16.127	33.36	4.7
3	D-*Threo*	16.94	89.68	1.6

The following is a table showing the variation in Δt_R between the single Marfey's (cf. Section III-B) and mixed Marfey's derivatization reactions. Shown is the difference in retention time on the extracted ion chromatogram (i.e., m/z Δt_R) between the *threo* diastereomers (i.e., *Threo* separation = absolute difference between L-*threo* and D-*threo*), the *erythro* diastereomers (i.e., *Erythro* separation = absolute difference between L-*erythro* and D-*erythro*), alpha-L-diastereomers (i.e., L-separation = absolute difference between L-*threo* and L-*erythro*), and the alpha-D-diastereomers (i.e., D-separation = absolute difference between D-*threo* and D-*erythro*). The change in Δt_R values are the absolute differences between the single Marfey's and mixed Marfey's reactions.

	Single Marfey's m/z Δt_R (min)	Mixed Marfey's m/z Δt_R (min)	Change in Δt_R (min)
Threo Separation	2.523	2.539	0.02
Erythro Separation	1.709	1.726	0.02
L-Separation	0	0	0.00
D-Separation	0.814	0.813	0.00

Extracted Ion Mass Spectrometry Data for the L-FDFA (i.e., 18) Adducts of Phenylserine from the Mixed Marfey's Derivatization Experiment

Extracted Ion Mass Chromatogram (ESI-ToF, extracted for m/z 508.1474 ± 0.005) for the adducts of Marfey's Reagent **18**. The Y-axis is ion counts, and the X-axis is acquisition time in minutes.

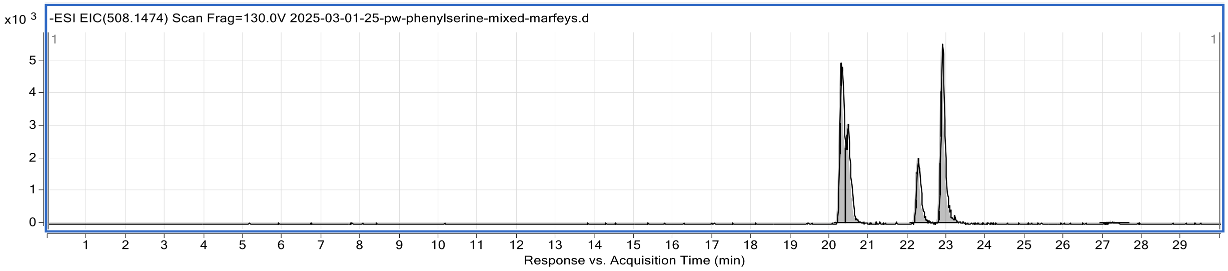

The following is a table of the peak data from the mass chromatogram. The Area % is the percent area relative to the tallest integrated peak, which has been set to 100%. The ΔArea is the absolute difference in percent area between the single and mixed Marfey's experiments.

Peak	Assignment	t_R (min)	Area (%)	ΔArea (%)
1	L-*Threo*	20.293	93.05	5.1
2	L-*Erythro*	20.475	50.01	2.7
3	D-*Erythro*	22.268	38.15	8.1
4	D-*Threo*	22.882	100	0.0

The following is a table showing the variation in Δt_R between the single Marfey's (cf. Section III-B) and mixed Marfey's derivatization reactions. Shown is the difference in retention time on the extracted ion chromatogram (i.e., m/z Δt_R) between the *threo* diastereomers (i.e., *Threo* separation = absolute difference between L-*threo* and D-*threo*), the *erythro* diastereomers (i.e., *Erythro* separation = absolute difference between L-*erythro* and D-*erythro*), alpha-L-diastereomers (i.e., L-separation = absolute difference between L-*threo* and L-*erythro*), and the alpha-D-diastereomers (i.e., D-separation = absolute difference between D-*threo* and D-*erythro*). The change in Δt_R values are the absolute differences between the single Marfey's and mixed Marfey's reactions.

	Single Marfey's m/z Δt_R (min)	Mixed Marfey's m/z Δt_R (min)	Change in Δt_R (min)
Threo Separation	2.572	2.589	0.02
Erythro Separation	1.792	1.793	0.00
L-Separation	0.166	0.182	0.02
D-Separation	0.614	0.614	0.00

Extracted Ion Mass Spectrometry Data for the L-FDWA (i.e., 19) Adducts of Phenylserine from the Mixed Marfey's Derivatization Experiment

Extracted Ion Mass Chromatogram (ESI-ToF, extracted for m/z 547.1583 ± 0.005) for the adducts of Marfey's Reagent **19**. The Y-axis is ion counts, and the X-axis is acquisition time in minutes.

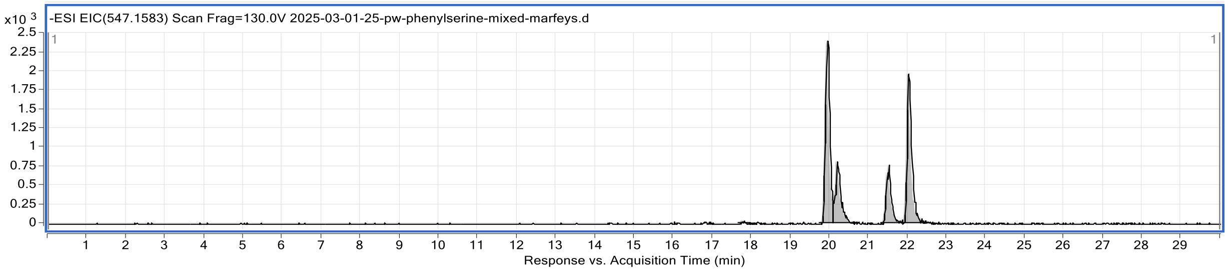

The following is a table of the peak data from the mass chromatogram. The Area % is the percent area relative to the tallest integrated peak, which has been set to 100%. The ΔArea is the absolute difference in percent area between the single and mixed Marfey's experiments.

Peak	Assignment	t_R (min)	Area (%)	ΔArea (%)
1	L-*Threo*	19.944	100	0.0
2	L-*Erythro*	20.177	41.22	8.7
3	D-*Erythro*	21.505	34.9	9.7
4	D-*Threo*	22.002	88.2	7.1

The following is a table showing the variation in Δt_R between the single Marfey's (cf. Section III-B) and mixed Marfey's derivatization reactions. Shown is the difference in retention time on the extracted ion chromatogram (i.e., m/z Δt_R) between the *threo* diastereomers (i.e., *Threo* separation = absolute difference between L-*threo* and D-*threo*), the *erythro* diastereomers (i.e., *Erythro* separation = absolute difference between L-*erythro* and D-*erythro*), alpha-L-diastereomers (i.e., L-separation = absolute difference between L-*threo* and L-*erythro*), and the alpha-D-diastereomers (i.e., D-separation = absolute difference between D-*threo* and D-*erythro*). The change in Δt_R values are the absolute differences between the single Marfey's and mixed Marfey's reactions.

	Single Marfey's m/z Δt_R (min)	Mixed Marfey's m/z Δt_R (min)	Change in Δt_R (min)
Threo Separation	2.074	2.058	0.02
Erythro Separation	1.294	1.328	0.03
L-Separation	0.249	0.233	0.02
D-Separation	0.531	0.497	0.03

Extracted Ion Mass Spectrometry Data for the L-FDPA (i.e., 9) Adducts of Phenylserine from the Mixed Marfey's Derivatization Experiment

Extracted Ion Mass Chromatogram (ESI-ToF, extracted for m/z 458.1317 ± 0.005) for the adducts of Marfey's Reagent **9**. The Y-axis is ion counts, and the X-axis is acquisition time in minutes.

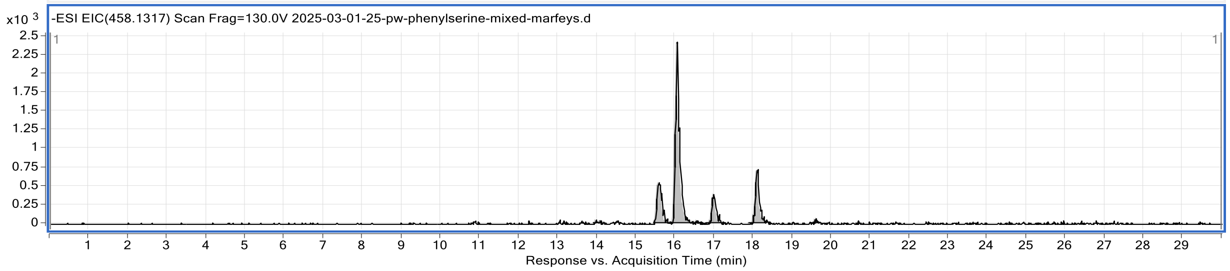

The following is a table of the peak data from the mass chromatogram. The Area % is the percent area relative to the tallest integrated peak, which has been set to 100%. The ΔArea is the absolute difference in percent area between the single and mixed Marfey's experiments.

Peak	Assignment	t_R (min)	Area (%)	ΔArea (%)
1	L-*Erythro*	15.579	29.04	1.1
2	L-*Threo*	16.06	100	0.0
3	D-*Erythro*	16.99	17.77	5.5
4	D-*Threo*	18.119	29.4	45.3

The following is a table showing the variation in Δt_R between the single Marfey's (cf. III-B) and mixed Marfey's derivatization reactions. Shown is the difference in retention time on the extracted ion chromatogram (i.e., m/z Δt_R) between the *threo* diastereomers (i.e., *Threo* separation = absolute difference between L-*threo* and D-*threo*), the *erythro* diastereomers (i.e., *Erythro* separation = absolute difference between L-*erythro* and D-*erythro*), alpha-L-diastereomers (i.e., L-separation = absolute difference between L-*threo* and L-*erythro*), and the alpha-D-diastereomers (i.e., D-separation = absolute difference between D-*threo* and D-*erythro*). The change in Δt_R values are the absolute differences between the single Marfey's and mixed Marfey's reactions.

	Single Marfey's m/z Δt_R (min)	Mixed Marfey's m/z Δt_R (min)	Change in Δt_R (min)
Threo Separation	2.058	2.059	0.00
Erythro Separation	1.411	1.411	0.00
L-Separation	0.449	0.481	0.03
D-Separation	1.096	1.129	0.03

Extracted Ion Mass Spectrometry Data for Sanger's reagent (i.e., 10) Adducts of Phenylserine from the Mixed Marfey's Derivatization Experiment

Extracted Ion Mass Chromatogram (ESI-ToF, extracted for m/z 346.0681 ± 0.005) for the adducts of Marfey's Reagent **10**. The Y-axis is ion counts, and the X-axis is acquisition time in minutes.

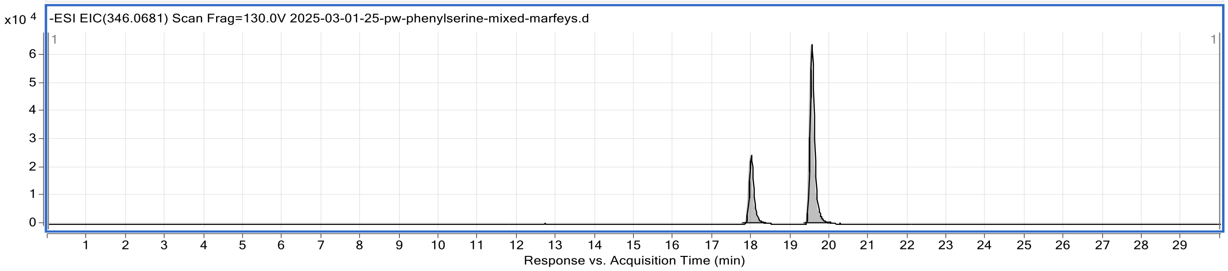

The following is a table of the peak data from the mass chromatogram. The Area % is the percent area relative to the tallest integrated peak, which has been set to 100%. The ΔArea is the absolute difference in percent area between the single and mixed Marfey's experiments.

Peak	Assignment	t_R (min)	Area (%)	ΔArea (%)
1	D,L-*Erythro*	17.986	38.5	2.8
2	D,L-*Threo*	19.529	100	0.0

The following is a table showing the variation in Δt_R between the single Marfey's (cf. Section III-B) and mixed Marfey's derivatization reactions. Shown is the difference in retention time on the extracted ion chromatogram (i.e., m/z Δt_R) between the two diastereomers (i.e., separation = absolute difference between D,L-*erythro* and D,L-*threo*). The change in Δt_R values are the absolute differences between the single Marfey's and mixed Marfey's reactions.

	Single Marfey's m/z Δt_R (min)	Mixed Marfey's m/z Δt_R (min)	Change in Δt_R (min)
Peak Separation	1.543	1.543	0.00

III-E. Comparison of the Mixed Marfey's Reaction on a Pure Sample Versus a "Complex" Sample of Phenylserine Diastereomers

**Note: The reactions discussed in this section were performed using a new version of the* Agilent Zorbax Extend-C18 column, 2.1 x 50 mm (1.8 µm), which caused a slight change in retention time from the experiments conducted in Sections III-B and III-D.

Repeating the Mixed Marfey's Reaction of Pure Samples Phenylserine Stereoisomers Using a new HPLC column:* A mixture of the four stereoisomers of phenylserine (30 µL, 10 mM in amino acid) was diluted in aq. $NaHCO_3$ (120 µL, 15 mM), and a solution of several Marfey's reagents (150 µL, 1.25 mM of Marfey's reagents **7**, **9**, **15**–**19** along with 1.25 mM Sanger's reagent **10**) was added. The final concentrations were 1 mM for the amino acids and 5 mM for the derivatizing agents. The resulting mixture was vortexed briefly and incubated at 37 ºC. After 20 h, the mixture was diluted with acetonitrile (150 µL) and aq. HCl (150 µL, 60 mM) and vortexed briefly. The resulting mixture was diluted 5-fold in 1:1 MeCN:H_2O (i.e., 200 µL of mixture diluted in 800 µL of 1:1 MeCN:H_2O), and analyzed by HPLC-MS using HPLC Method A. Because a new column was used, the retention times changed slightly from those observed with the experiments discussed in Section III-D.

Complex Mixed Marfey's Reaction of Phenylserine Stereoisomers Mixed with Nine Additional Amino Acids:* 30 µL of a mixture containing the four stereoisomers of phenylserine (3.3 mM), D-isoleucine (1.1 mM), L-isoleucine (1.1 mM), D-*allo*-isoleucine (1.1 mM), L-*allo*-isoleucine (1.1 mM), glycine (0.7 mM), L-threonine (0.7 mM), L-leucine (0.7 mM), L-arginine (0.7 mM), and L-phenylalanine (0.7 mM) was diluted in aq. $NaHCO_3$ (120 µL, 15 mM), and a solution of several Marfey's reagents (150 µL, 1.25 mM of Marfey's reagents **7**, **9**, **15**–**19** along with 1.25 mM Sanger's reagent **10**) was added. The resulting mixture was vortexed briefly and incubated at 37 ºC. After 20 h, the mixture was diluted with acetonitrile (150 µL) and aq. HCl (150 µL, 60 mM) and vortexed briefly. The resulting mixture was diluted 5-fold in 1:1 MeCN:H_2O (i.e., 200 µL of mixture diluted in 800 µL of 1:1 MeCN:H_2O), and analyzed by HPLC-MS using HPLC Method A.

LC/MS Traces Related to Section III-E

Total Ion Mass Chromatogram (ESI-ToF) for the mixed Marfey's reaction of pure samples phenylserine stereoisomers using a new HPLC column (Top) and complex mixed Marfey's reaction of phenylserine stereoisomers mixed with nine additional amino acids (Bottom). The Y-axis is ion counts, and the X-axis is acquisition time in minutes.

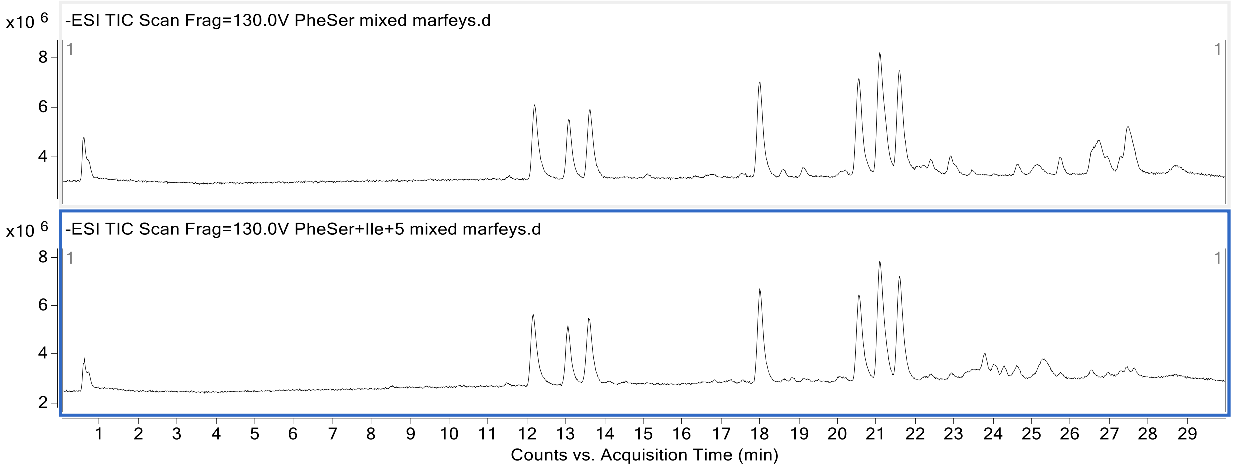

Comparing the Extracted Ion Mass Spectrometry Data for the L-FDAA (i.e., 7) Adducts of Phenylserine from the Mixed Marfey's and "Complex" Mixed Marfey's Experiment

Extracted Ion Mass Chromatogram (ESI-ToF, extracted for m/z 432.1161 ± 0.005) for the mixed Marfey's reaction of pure samples phenylserine stereoisomers using a new HPLC column (Top) and complex mixed Marfey's reaction of phenylserine stereoisomers mixed with nine additional amino acids (Bottom). The Y-axis is ion counts, and the X-axis is acquisition time in minutes.

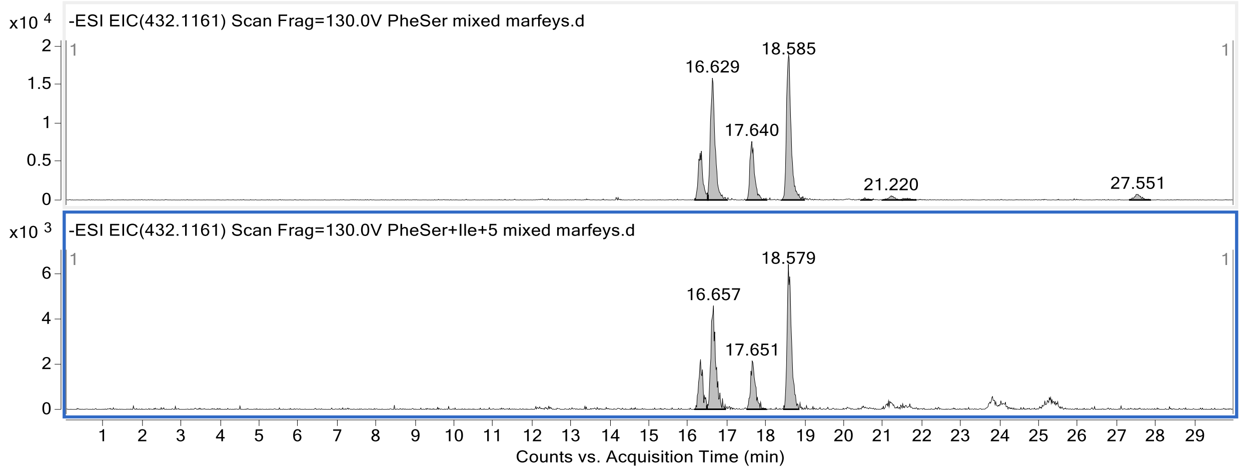

The following is a table showing the variation in t_R for the above Extracted Ion Chromatograms (i.e., the mixed Marfey's and "complex" mixed Marfey's derivatization reactions).

Peak	Assignment	Mixed Marfey's m/z t_R (min)	Complex Mixed Marfey's m/z t_R (min)	Δt_R (min)
1	L-*Erythro*	16.347	16.325	0.02
2	L-*Threo*	16.629	16.657	0.03
3	D-*Erythro*	17.640	17.651	0.01
4	D-*Threo*	18.585	18.579	0.01

Comparing the Extracted Ion Mass Spectrometry Data for the L-FDVA (i.e., 15) Adducts of Phenylserine from the Mixed Marfey's and "Complex" Mixed Marfey's Experiment

Extracted Ion Mass Chromatogram (ESI-ToF, extracted for m/z 460.1474 ± 0.005) for the mixed Marfey's reaction of pure samples phenylserine stereoisomers using a new HPLC column (Top) and complex mixed Marfey's reaction of phenylserine stereoisomers mixed with nine additional amino acids (Bottom). The Y-axis is ion counts, and the X-axis is acquisition time in minutes.

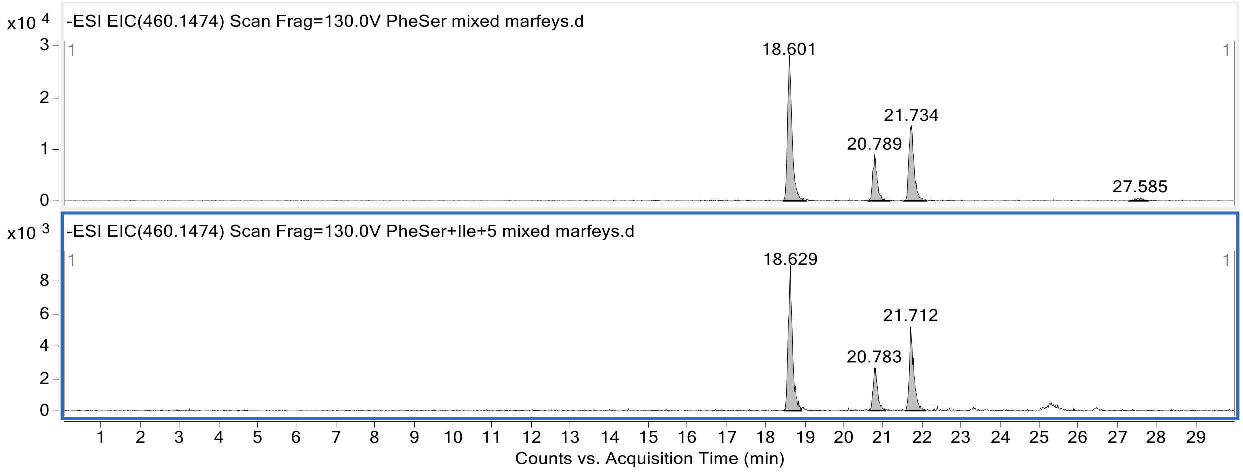

The following is a table showing the variation in t_R for the above Extracted Ion Chromatograms (i.e., the mixed Marfey's and "complex" mixed Marfey's derivatization reactions).

Peak	Assignment	Mixed Marfey's m/z t_R (min)	Complex Mixed Marfey's m/z t_R (min)	Δt_R (min)
1	L-*Erythro*/ L-*Threo*	18.601	18.629	0.03
2	D-*Erythro*	20.789	20.783	0.01
3	D-*Threo*	21.734	21.712	0.02

Comparing the Extracted Ion Mass Spectrometry Data for the L-FDIA (i.e., 16) Adducts of Phenylserine from the Mixed Marfey's and "Complex" Mixed Marfey's Experiment

Extracted Ion Mass Chromatogram (ESI-ToF, extracted for m/z 474.1630 ± 0.005) for the mixed Marfey's reaction of pure samples phenylserine stereoisomers using a new HPLC column (Top) and complex mixed Marfey's reaction of phenylserine stereoisomers mixed with nine additional amino acids (Bottom). The Y-axis is ion counts, and the X-axis is acquisition time in minutes.

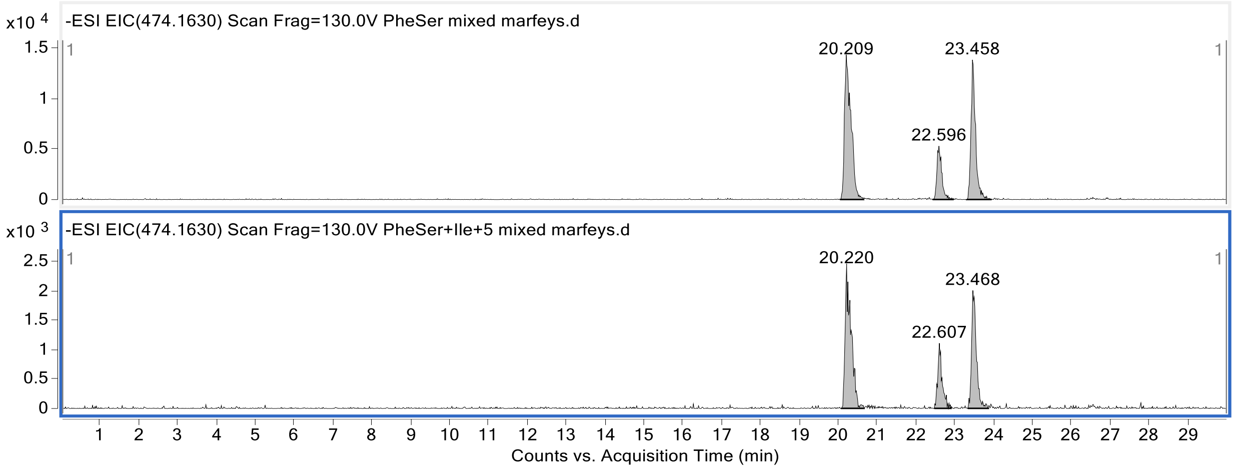

The following is a table showing the variation in t_R for the above Extracted Ion Chromatograms (i.e., the mixed Marfey's and "complex" mixed Marfey's derivatization reactions).

Peak	Assignment	Mixed Marfey's m/z t_R (min)	Complex Mixed Marfey's m/z t_R (min)	Δt_R (min)
1	L-*Erythro*/ L-*Threo*	20.209	20.220	0.01
2	D-*Erythro*	22.596	22.607	0.01
3	D-*Threo*	23.458	23.468	0.01

Comparing the Extracted Ion Mass Spectrometry Data for the L-FDTA (i.e., 17) Adducts of Phenylserine from the Mixed Marfey's and "Complex" Mixed Marfey's Experiment

Extracted Ion Mass Chromatogram (ESI-ToF, extracted for m/z 462.1267 ± 0.005) for the mixed Marfey's reaction of pure samples phenylserine stereoisomers using a new HPLC column (Top) and complex mixed Marfey's reaction of phenylserine stereoisomers mixed with nine additional amino acids (Bottom). The Y-axis is ion counts, and the X-axis is acquisition time in minutes.

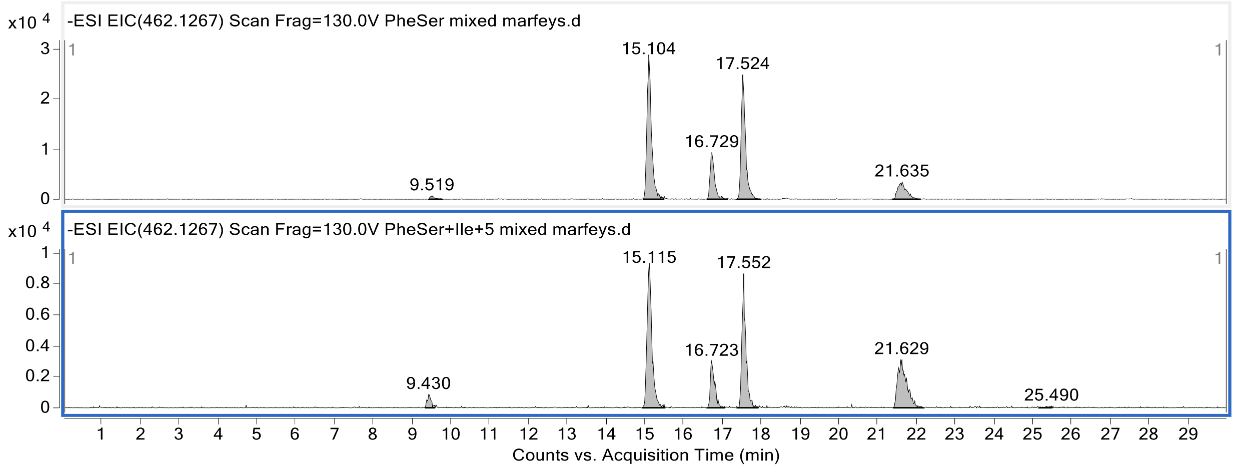

The following is a table showing the variation in t_R for the above Extracted Ion Chromatograms (i.e., the mixed Marfey's and "complex" mixed Marfey's derivatization reactions).

Peak	Assignment	Mixed Marfey's m/z t_R (min)	Complex Mixed Marfey's m/z t_R (min)	Δt_R (min)
1	L-*Erythro*/ L-*Threo*	15.104	15.115	0.01
2	D-*Erythro*	16.729	16.723	0.01
3	D-*Threo*	17.524	17.552	0.03

Comparing the Extracted Ion Mass Spectrometry Data for the L-FDFA (i.e., 18) Adducts of Phenylserine from the Mixed Marfey's and "Complex" Mixed Marfey's Experiment

Extracted Ion Mass Chromatogram (ESI-ToF, extracted for m/z 508.1474 ± 0.005) for the mixed Marfey's reaction of pure samples phenylserine stereoisomers using a new HPLC column (Top) and complex mixed Marfey's reaction of phenylserine stereoisomers mixed with nine additional amino acids (Bottom). The Y-axis is ion counts, and the X-axis is acquisition time in minutes.

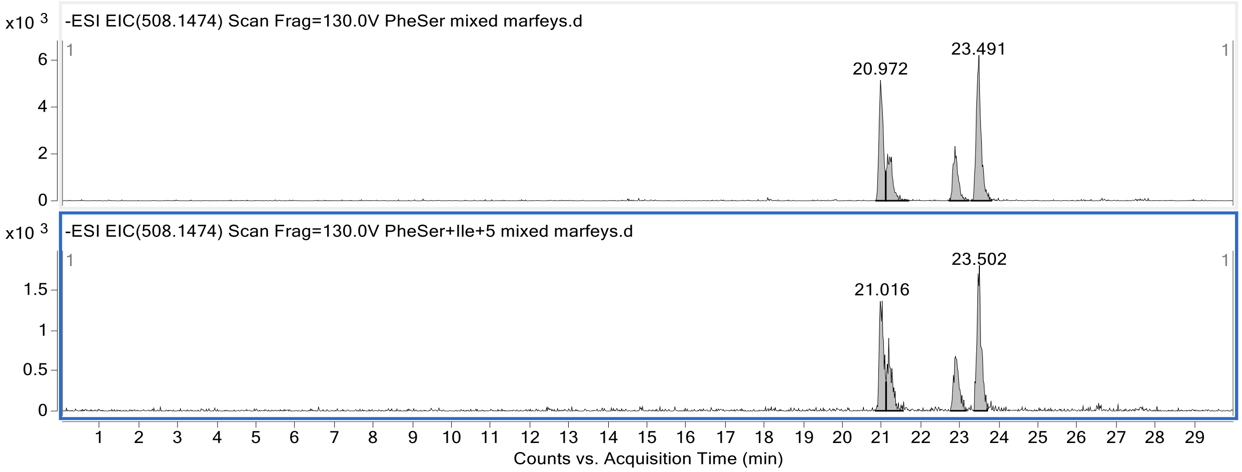

The following is a table showing the variation in t_R for the above Extracted Ion Chromatograms (i.e., the mixed Marfey's and "complex" mixed Marfey's derivatization reactions).

Peak	Assignment	Mixed Marfey's m/z t_R (min)	Complex Mixed Marfey's m/z t_R (min)	Δt_R (min)
1	L-*Threo*	20.972	21.016	0.04
2	L-*Erythro*	21.154	21.181	0.03
3	D-*Erythro*	22.878	22.888	0.01
4	D-*Threo*	23.491	23.502	0.01

Comparing the Extracted Ion Mass Spectrometry Data for the L-FDWA (i.e., 19) Adducts of Phenylserine from the Mixed Marfey's and "Complex" Mixed Marfey's Experiment

Extracted Ion Mass Chromatogram (ESI-ToF, extracted for m/z 547.1583 ± 0.005) for the mixed Marfey's reaction of pure samples phenylserine stereoisomers using a new HPLC column (Top) and complex mixed Marfey's reaction of phenylserine stereoisomers mixed with nine additional amino acids (Bottom). The Y-axis is ion counts, and the X-axis is acquisition time in minutes.

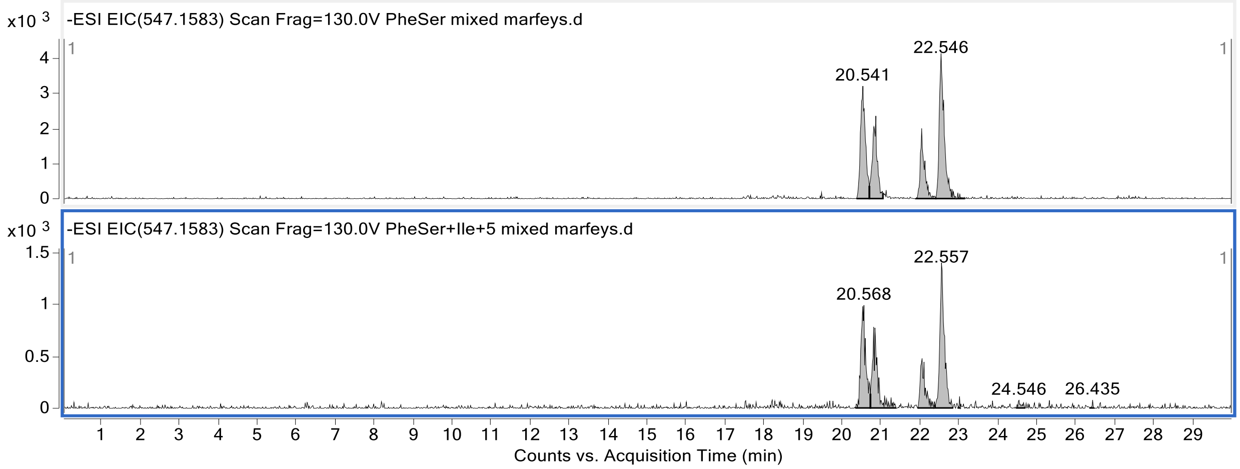

The following is a table showing the variation in t_R for the above Extracted Ion Chromatograms (i.e., the mixed Marfey's and "complex" mixed Marfey's derivatization reactions).

Peak	Assignment	Mixed Marfey's m/z t_R (min)	Complex Mixed Marfey's m/z t_R (min)	Δt_R (min)
1	L-*Threo*	20.541	20.568	0.03
2	L-*Erythro*	20.872	20.817	0.05
3	D-*Erythro*	22.049	22.060	0.01
4	D-*Threo*	22.546	22.557	0.01

Comparing the Extracted Ion Mass Spectrometry Data for the L-FDPA (i.e., 9) Adducts of Phenylserine from the Mixed Marfey's and "Complex" Mixed Marfey's Experiment

Extracted Ion Mass Chromatogram (ESI-ToF, extracted for m/z 458.1317 ± 0.005) for the mixed Marfey's reaction of pure samples phenylserine stereoisomers using a new HPLC column (Top) and complex mixed Marfey's reaction of phenylserine stereoisomers mixed with nine additional amino acids (Bottom). The Y-axis is ion counts, and the X-axis is acquisition time in minutes.

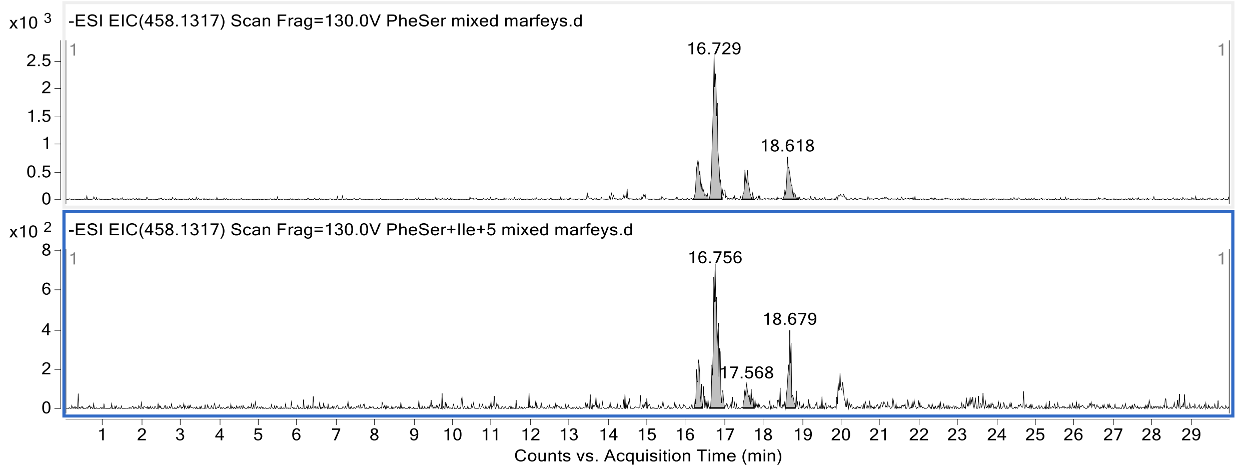

The following is a table showing the variation in t_R for the above Extracted Ion Chromatograms (i.e., the mixed Marfey's and "complex" mixed Marfey's derivatization reactions).

Peak	Assignment	Mixed Marfey's m/z t_R (min)	Complex Mixed Marfey's m/z t_R (min)	Δt_R (min)
1	L-*Erythro*	16.314	16.325	0.01
2	L-*Threo*	16.729	16.756	0.03
3	D-*Erythro*	17.524	17.568	0.04
4	D-*Threo*	18.618	18.679	0.06

Comparing the Extracted Ion Mass Spectrometry Data for Sanger's reagent (i.e., 10) Adducts of Phenylserine from the Mixed Marfey's and "Complex" Mixed Marfey's Experiment

Extracted Ion Mass Chromatogram (ESI-ToF, extracted for m/z 346.0681 ± 0.005) for the mixed Marfey's reaction of pure samples phenylserine stereoisomers using a new HPLC column (Top) and complex mixed Marfey's reaction of phenylserine stereoisomers mixed with nine additional amino acids (Bottom). The Y-axis is ion counts, and the X-axis is acquisition time in minutes.

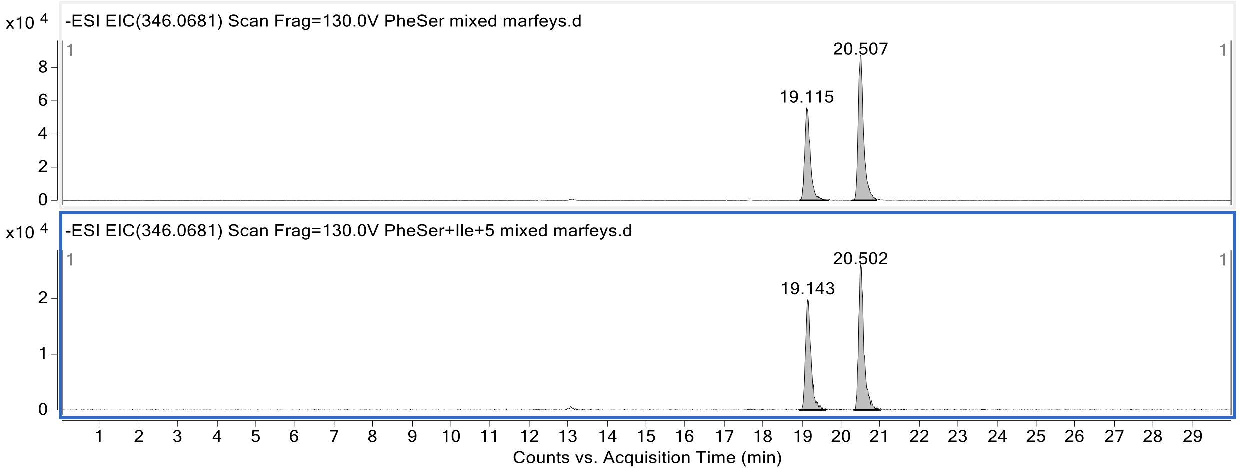

The following is a table showing the variation in t_R for the above Extracted Ion Chromatograms (i.e., the mixed Marfey's and "complex" mixed Marfey's derivatization reactions).

Peak	Assignment	Mixed Marfey's m/z t_R (min)	Complex Mixed Marfey's m/z t_R (min)	Δt_R (min)
1	D,L-*Erythro*	19.115	19.143	0.03
2	D,L-*Threo*	20.507	20.502	0.01

III-F. Mixed Marfey's Reaction for the Multiplexed Method Scouting of Different Chiral Derivatizing Agents in the Resolution of the Four Stereoisomers of Isoleucine

A mixture of the four stereoisomers (30 µL, ~2 mM L-Ile, ~2 mM D-Ile, ~3 mM L-*allo*-Ile, and ~3 mM D-*allo*-Ile) was diluted in aq. $NaHCO_3$ (120 µL, 15 mM), and a solution of several Marfey's reagents (150 µL, 1.25 mM of Marfey's reagents **7**, **9**, **15**–**19** along with 1.25 mM Sanger's reagent **10**) was added. The final concentrations were 1 mM for the amino acids and 5 mM for the derivatizing agents. The resulting mixture was vortexed briefly and incubated at 37 ºC. After 20 h, the mixture was diluted with acetonitrile (150 µL) and aq. HCl (150 µL, 60 mM) and vortexed briefly. The resulting mixture was diluted 5-fold in 1:1 MeCN:H_2O (i.e., 200 µL of mixture diluted in 800 µL of 1:1 MeCN:H_2O), and analyzed by HPLC-MS using HPLC Method A.

LC/MS Traces for the Attempted Resolution of the Diastereomers of Isoleucine with a Mixture of Seven Marfey's Reagents and Sanger's Reagent (i.e., Mixed Marfey's Reaction) with HPLC Method A (i.e., 25 min.)

Total Ion Mass Chromatogram (ESI-ToF). The Y-axis is ion counts, and the X-axis is acquisition time in minutes.

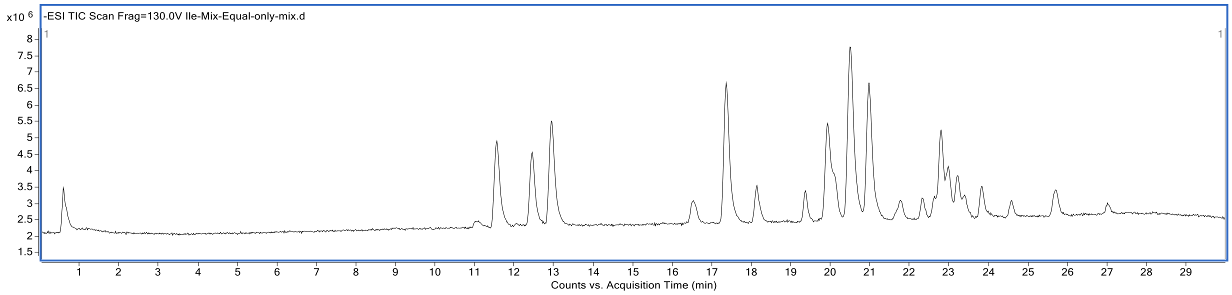

Variable Wavelength Detector Chromatogram (340 nm). The Y-axis is absorbance units, and the X-axis is acquisition time in minutes.

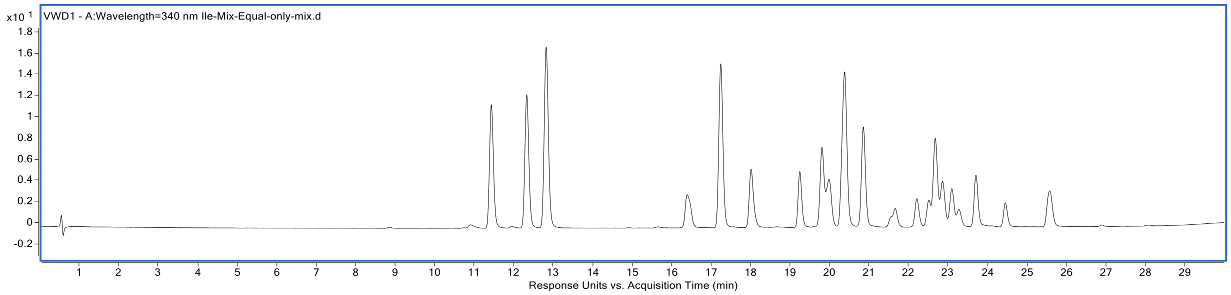

Extracted Ion Mass Spectrometry Data for the L-FDAA (i.e., 7) Adducts of Isoleucine from the Mixed Marfey's Derivatization Experiment

Extracted Ion Mass Chromatogram (ESI-ToF, extracted for m/z 382.1368 ± 0.005) for the adducts of Marfey's Reagent **7**. The Y-axis is ion counts, and the X-axis is acquisition time in minutes.

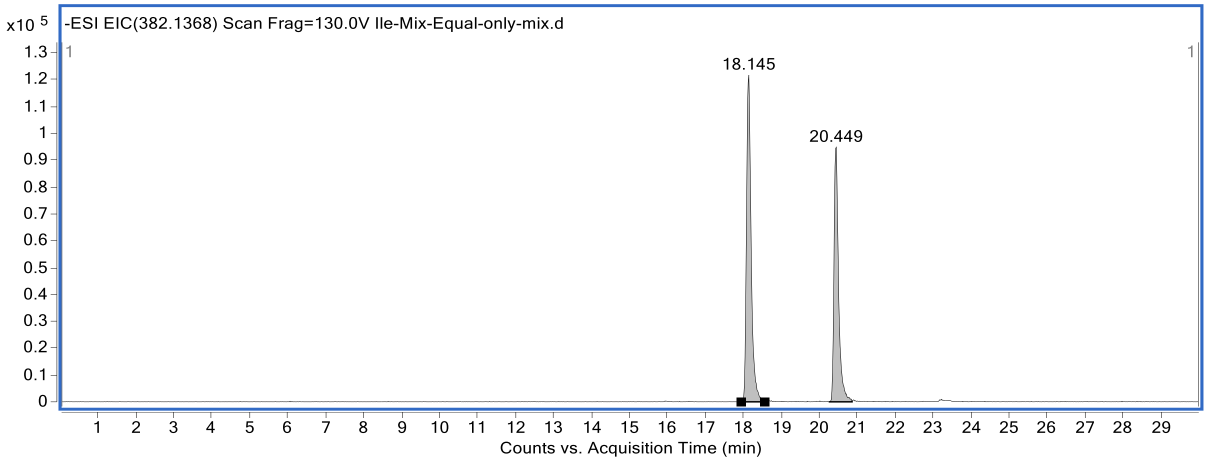

The following is a table of the peak data from the mass chromatogram.

Peak	Assignment	t_R (min)
1	L-*allo*-Ile/ L-Ile	18.145
2	D-*allo*-Ile/ D-Ile	20.449

The following is a table showing the variation in Δt_R between the single Marfey's (cf. Section III-B) and mixed Marfey's derivatization reactions. Shown is the difference in retention time on the extracted ion chromatogram (i.e., m/z Δt_R) between the D and L diastereomers (i.e., separation = absolute difference between L-Ile/L-*allo*-Ile and D-Ile/D-*allo*-Ile). The change in Δt_R values are the absolute differences between the single Marfey's and mixed Marfey's reactions.

	Single Marfey's m/z Δt_R (min)	Mixed Marfey's m/z Δt_R (min)	Change in Δt_R (min)
Peak Separation	2.328	2.304	0.02

Extracted Ion Mass Spectrometry Data for the L-FDVA (i.e., 15) Adducts of Isoleucine from the Mixed Marfey's Derivatization Experiment

Extracted Ion Mass Chromatogram (ESI-ToF, extracted for m/z 410.1681 ± 0.005) for the adducts of Marfey's Reagent **15**. The Y-axis is ion counts, and the X-axis is acquisition time in minutes.

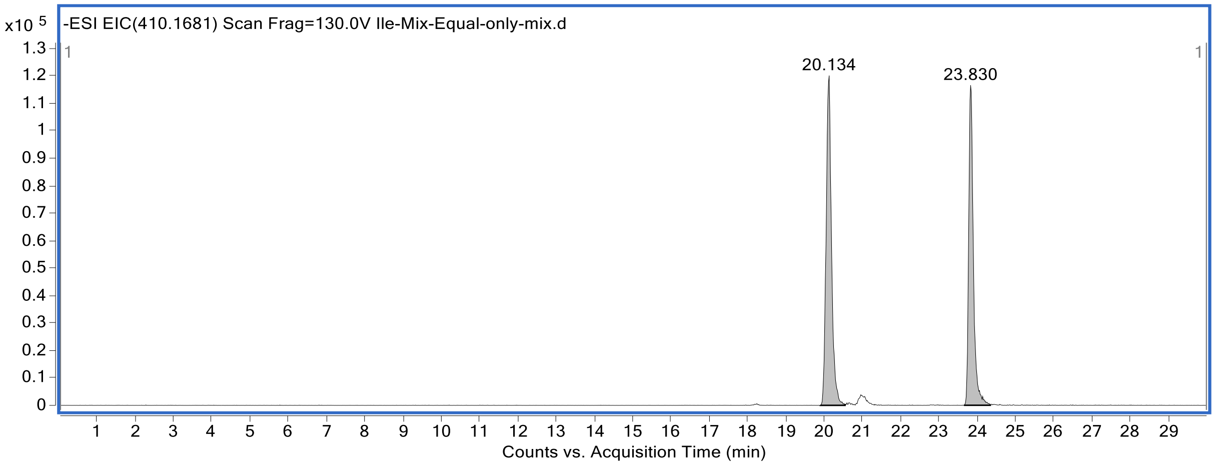

The following is a table of the peak data from the mass chromatogram.

Peak	Assignment	t_R (min)
1	L-*allo*-Ile/ L-Ile	20.134
2	D-*allo*-Ile/ D-Ile	23.830

The following is a table showing the variation in Δt_R between the single Marfey's (cf. Section III-B) and mixed Marfey's derivatization reactions. Shown is the difference in retention time on the extracted ion chromatogram (i.e., m/z Δt_R) between the D and L diastereomers (i.e., separation = absolute difference between L-Ile/L-*allo*-Ile and D-Ile/D-*allo*-Ile). The change in Δt_R values are the absolute differences between the single Marfey's and mixed Marfey's reactions.

	Single Marfey's m/z Δt_R (min)	Mixed Marfey's m/z Δt_R (min)	Change in Δt_R (min)
Peak Separation	3.696	3.696	0.02

Extracted Ion Mass Spectrometry Data for the L-FDIA (i.e., 16) Adducts of Isoleucine from the Mixed Marfey's Derivatization Experiment

Extracted Ion Mass Chromatogram (ESI-ToF, extracted for m/z 424.1838 ± 0.005) for the adducts of Marfey's Reagent **16**. The Y-axis is ion counts, and the X-axis is acquisition time in minutes.

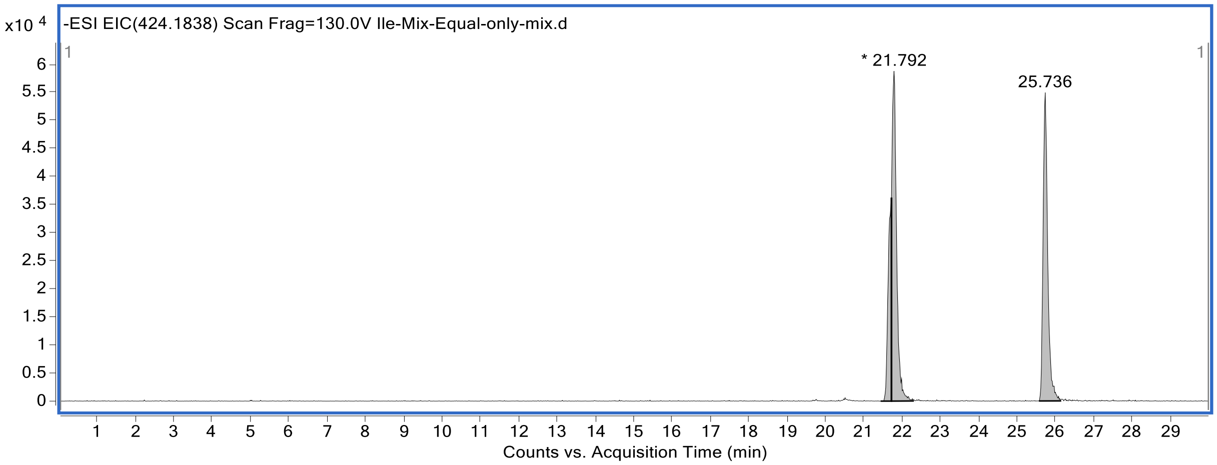

The following is a table of the peak data from the mass chromatogram.

Peak	Assignment	t_R (min)
1	L-Ile	21.692
2	L-*allo*-Ile	21.792
3	D-*allo*-Ile, D-Ile	25.736

The following is a table showing the variation in Δt_R between the single Marfey's (cf. Section III-B) and mixed Marfey's derivatization reactions. Shown is the difference in retention time on the extracted ion chromatogram (i.e., m/z Δt_R) between the D and L diastereomers. The L-separation was estimated from the shoulder observed on the distinguishing of Peaks 1 and 2. The Ile separation was determined from the difference in t_R between peaks 2 and 3. The change in Δt_R values are the absolute differences between the single Marfey's and mixed Marfey's reactions.

	Single Marfey's m/z Δt_R (min)	Mixed Marfey's m/z Δt_R (min)	Change in Δt_R (min)
allo-Ile Separation	3.957	3.944	0.01
Ile Separation	4.056	4.044	0.01
L-Separation	0.099	0.100	0.00
D-Separation	0	0	0

Extracted Ion Mass Spectrometry Data for the L-FDTA (i.e., 17) Adducts of Isoleucine from the Mixed Marfey's Derivatization Experiment

Extracted Ion Mass Chromatogram (ESI-ToF, extracted for m/z 412.1474 ± 0.005) for the adducts of Marfey's Reagent **17**. The Y-axis is ion counts, and the X-axis is acquisition time in minutes.

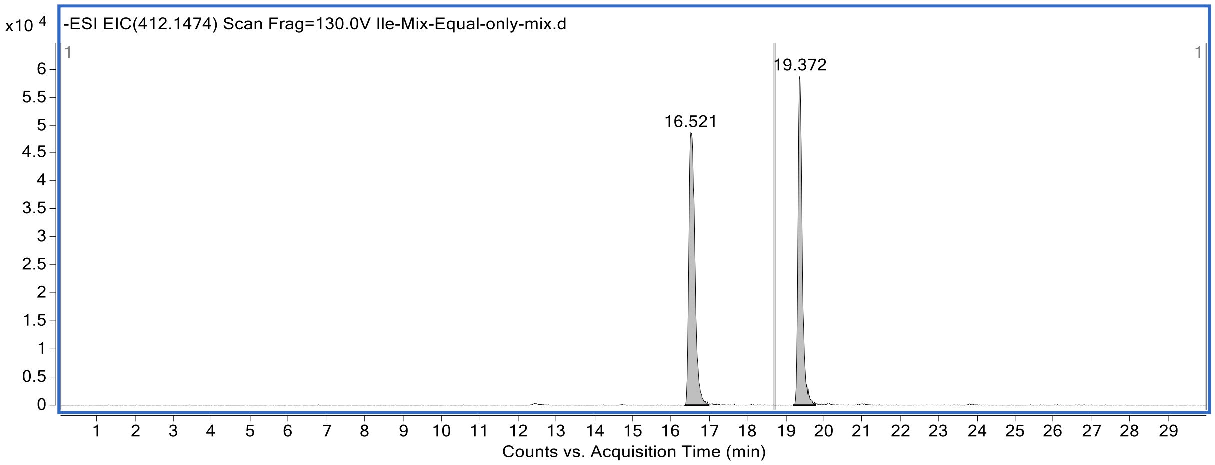

The following is a table of the peak data from the mass chromatogram.

Peak	Assignment	t_R (min)
1	L-*allo*-Ile/ L-Ile	16.521
2	D-*allo*-Ile/ D-Ile	19.372

The following is a table showing the variation in Δt_R between the single Marfey's (cf. Section III-B) and mixed Marfey's derivatization reactions. Shown is the difference in retention time on the extracted ion chromatogram (i.e., m/z Δt_R) between the D and L diastereomers (i.e., separation = absolute difference between L-Ile/L-*allo*-Ile and D-Ile/D-*allo*-Ile). The change in Δt_R values are the absolute differences between the single Marfey's and mixed Marfey's reactions.

	Single Marfey's m/z Δt_R (min)	Mixed Marfey's m/z Δt_R (min)	Change in Δt_R (min)
Peak Separation	2.851	2.851	0.00

Extracted Ion Mass Spectrometry Data for the L-FDFA (i.e., 18) Adducts of Isoleucine from the Mixed Marfey's Derivatization Experiment

Extracted Ion Mass Chromatogram (ESI-ToF, extracted for m/z 458.1681 ± 0.005) for the adducts of Marfey's Reagent **18**. The Y-axis is ion counts, and the X-axis is acquisition time in minutes.

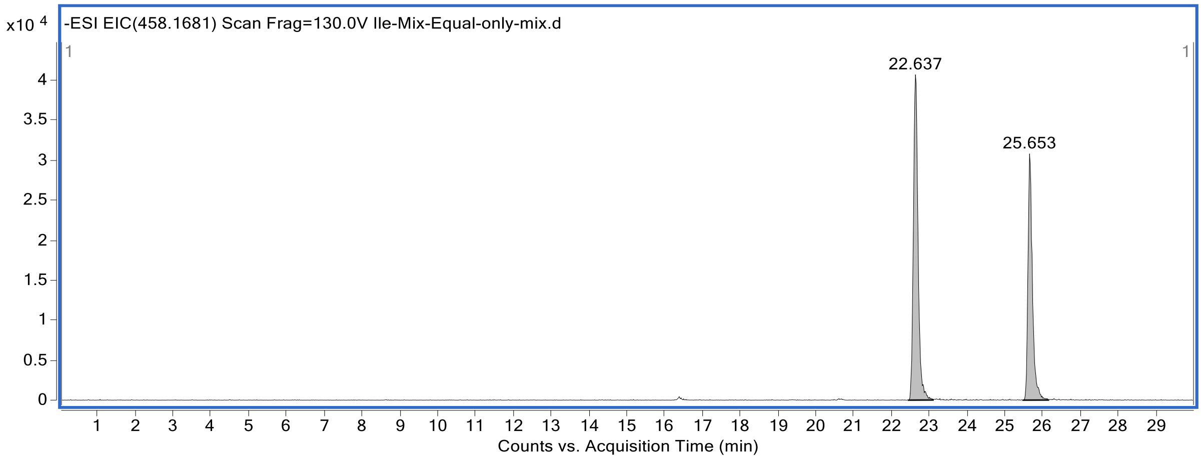

The following is a table of the peak data from the mass chromatogram.

Peak	Assignment	t_R (min)
1	L-*allo*-Ile/ L-Ile	22.637
2	D-*allo*-Ile/ D-Ile	25.653

The following is a table showing the variation in Δt_R between the single Marfey's (cf. Section III-B) and mixed Marfey's derivatization reactions. Shown is the difference in retention time on the extracted ion chromatogram (i.e., m/z Δt_R) between the D and L diastereomers (i.e., separation = absolute difference between L-Ile/L-*allo*-Ile and D-Ile/D-*allo*-Ile). The change in Δt_R values are the absolute differences between the single Marfey's and mixed Marfey's reactions.

	Single Marfey's m/z Δt_R (min)	Mixed Marfey's m/z Δt_R (min)	Change in Δt_R (min)
Peak Separation	3.019	3.016	0.00

Extracted Ion Mass Spectrometry Data for the L-FDWA (i.e., 19) Adducts of Isoleucine from the Mixed Marfey's Derivatization Experiment

Extracted Ion Mass Chromatogram (ESI-ToF, extracted for m/z 497.1790 ± 0.005) for the adducts of Marfey's Reagent **19**. The Y-axis is ion counts, and the X-axis is acquisition time in minutes.

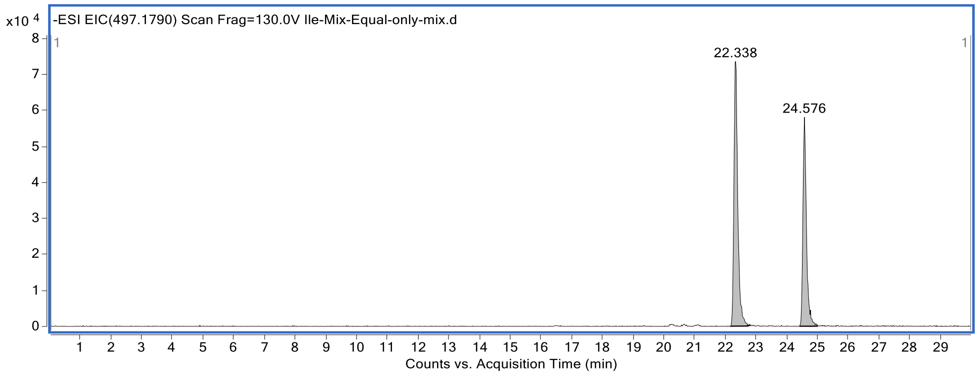

The following is a table of the peak data from the mass chromatogram.

Peak	Assignment	t_R (min)
1	L-*allo*-Ile/ L-Ile	22.338
2	D-*allo*-Ile/ D-Ile	24.576

The following is a table showing the variation in Δt_R between the single Marfey's (cf. Section III-B) and mixed Marfey's derivatization reactions. Shown is the difference in retention time on the extracted ion chromatogram (i.e., m/z Δt_R) between the D and L diastereomers (i.e., separation = absolute difference between L-Ile/L-*allo*-Ile and D-Ile/D-*allo*-Ile). The change in Δt_R values are the absolute differences between the single Marfey's and mixed Marfey's reactions.

	Single Marfey's m/z Δt_R (min)	Mixed Marfey's m/z Δt_R (min)	Change in Δt_R (min)
Peak Separation	2.254	2.238	0.02

Extracted Ion Mass Spectrometry Data for the L-FDPA (i.e., 9) Adducts of Isoleucine from the Mixed Marfey's Derivatization Experiment

Extracted Ion Mass Chromatogram (ESI-ToF, extracted for m/z 408.1525 ± 0.005) for the adducts of Marfey's Reagent **9**. The Y-axis is ion counts, and the X-axis is acquisition time in minutes.

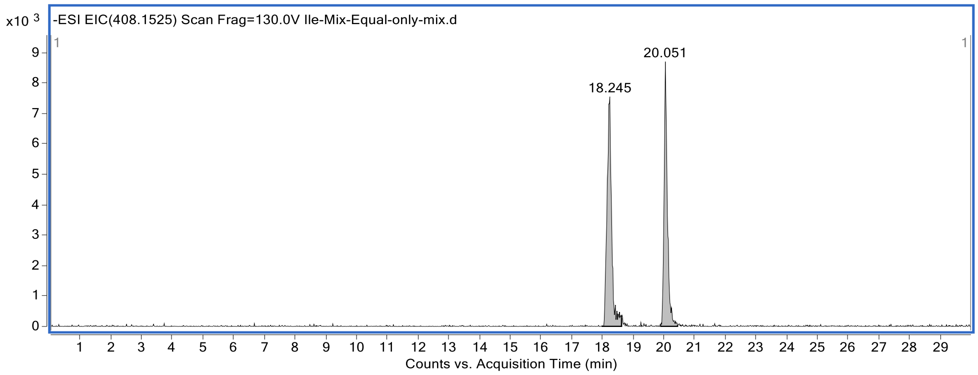

The following is a table of the peak data from the mass chromatogram.

Peak	Assignment	t_R (min)
1	L-*allo*-Ile/ L-Ile	18.245
2	D-*allo*-Ile/ D-Ile	20.051

The following is a table showing the variation in Δt_R between the single Marfey's (cf. Section III-B) and mixed Marfey's derivatization reactions. Shown is the difference in retention time on the extracted ion chromatogram (i.e., m/z Δt_R) between the D and L diastereomers (i.e., separation = absolute difference between L-Ile/L-*allo*-Ile and D-Ile/D-*allo*-Ile). The change in Δt_R values are the absolute differences between the single Marfey's and mixed Marfey's reactions.

	Single Marfey's m/z Δt_R (min)	Mixed Marfey's m/z Δt_R (min)	Change in Δt_R (min)
Peak Separation	1.840	1.806	0.03

Extracted Ion Mass Spectrometry Data for Sanger's reagent (i.e., 10) Adducts of Isoleucine from the Mixed Marfey's Derivatization Experiment

Extracted Ion Mass Chromatogram (ESI-ToF, extracted for m/z 296.0888 ± 0.005) for the adducts of Marfey's Reagent **10**. The Y-axis is ion counts, and the X-axis is acquisition time in minutes.

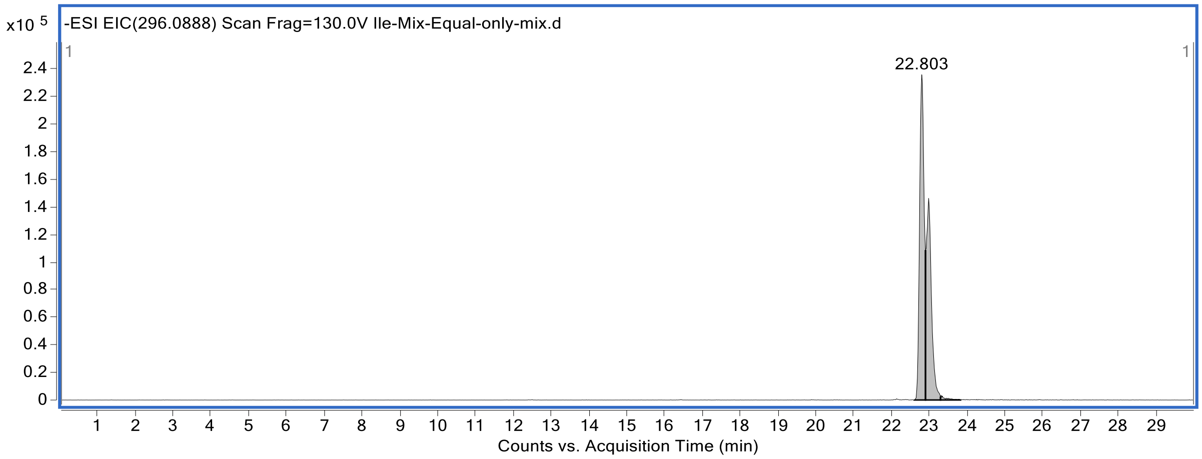

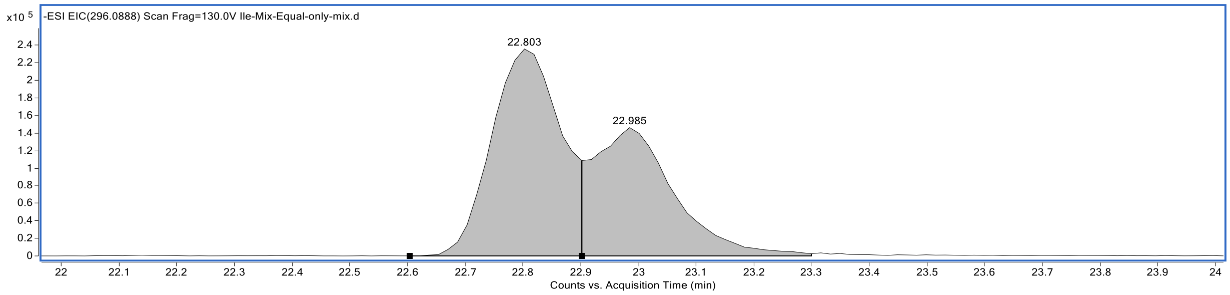

The following is a table of the peak data from the mass chromatogram.

Peak	Assignment	t_R (min)
1	D,L-*allo*-Ile	22.803
2	D,L-Ile	22.985

The following is a table showing the variation in Δt_R between the single Marfey's (cf. Section III-B) and mixed Marfey's derivatization reactions. Shown is the difference in retention time on the extracted ion chromatogram (i.e., m/z Δt_R) between the D and L diastereomers (i.e., separation = absolute difference between L-Ile/L-*allo*-Ile and D-Ile/D-*allo*-Ile). The change in Δt_R values are the absolute differences between the single Marfey's and mixed Marfey's reactions.

	Single Marfey's m/z Δt_R (min)	Mixed Marfey's m/z Δt_R (min)	Change in Δt_R (min)
Peak Separation	0.182	0.182	0.02

III-G. Separation of a Stereoisomeric Mixture of Isoleucine with Various Marfey's Reagents

LC/MS Traces for the Attempted Resolution of the Four Stereoisomers of Isoleucine with 1-Fluoro-2,4-dinitrophenyl-5-L-alanine Amide (i.e., L-FDAA 7) with HPLC Method A (i.e., 25 min.)

Total Ion Mass Chromatogram (ESI-ToF). The Y-axis is ion counts, and the X-axis is acquisition time in minutes.

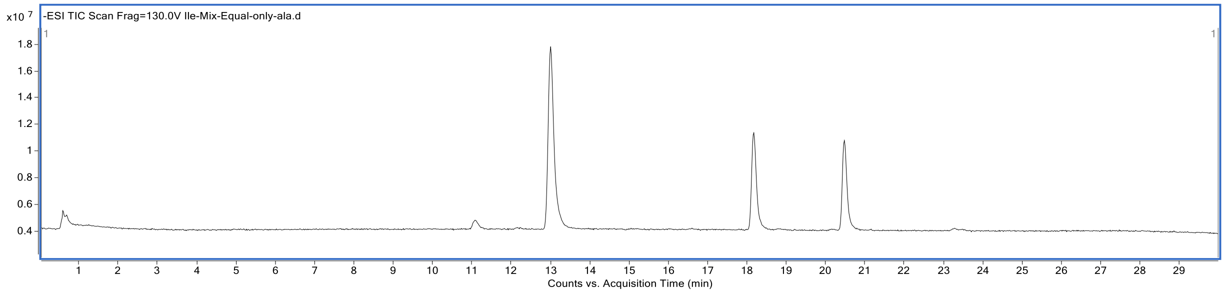

Variable Wavelength Detector Chromatogram (340 nm). The Y-axis is absorbance units, and the X-axis is acquisition time in minutes.

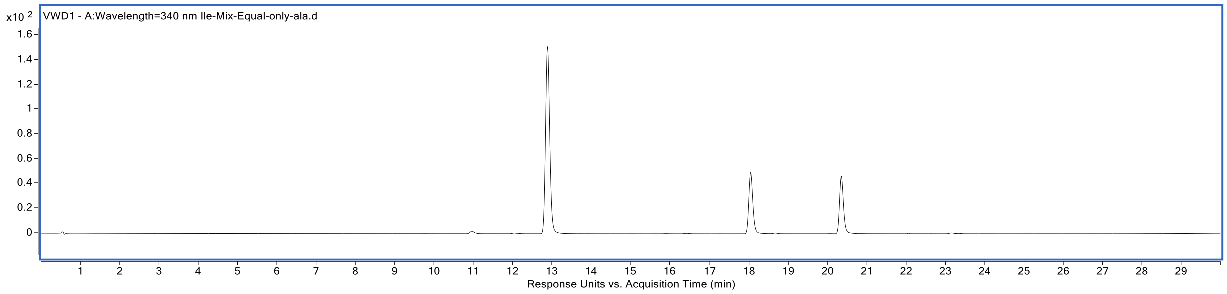

Extracted Ion Mass Chromatogram (ESI-ToF, extracted for m/z 382 ± 0.5). The Y-axis is ion counts, and the X-axis is acquisition time in minutes.

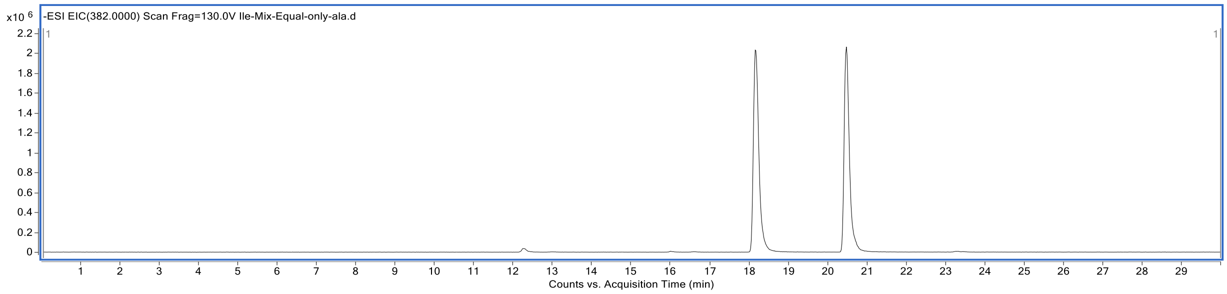

Zoomed and rescaled Extracted Ion (top) and Variable Wavelength Detector (bottom) Chromatograms. The Y axis on top is ion counts, the Y-axis on the bottom is absorbance units, and the X-axis for both is acquisition time in minutes. The peaks are labeled from left to right as Peak 1 and Peak 2.

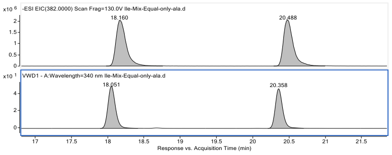

The following is a table of the peak data from the mass chromatogram. The Area % is the percent area relative to the tallest integrated peak, which has been set to 100%.

Peak	Assignment	t_R (min)	ES-ToF m/z [Neg]	Area %
1	L-*allo*-Ile/ L-Ile	18.160	382.1389	100
2	D-*allo*-Ile/ D-Ile	20.488	382.1389	97.10

The calculated m/z for the adduct of isoleucine with the Marfey's reagent is 382.1368 for $C_{15}H_{20}N_5O_7^-$ $[M - H]^{-1}$.

The following is a table of the peak data from the variable wavelength detector chromatogram (340 nm). The Area % is the percent area relative to the tallest integrated peak, which has been set to 100%.

Peak	Assignment	t_R (min)	Area %
1	L-*allo*-Ile/ L-Ile	18.051	100
2	D-*allo*-Ile/ D-Ile	20.358	91.64

The following is a table showing the difference in retention time on the extracted ion chromatogram (i.e., m/z Δt_R) and variable wavelength detector (i.e., VWD Δt_R) between the *allo*-Ile diastereomers (i.e., *allo*-Ile separation = absolute difference between L-*allo*-Ile and D-*allo*-Ile), the canonical isoleucine diastereomers (i.e., Ile separation = absolute difference between L-Ile and D-Ile), alpha-L-diastereomers (i.e., L-separation = absolute difference between L-*allo*-Ile and L-Ile), and the alpha-D-diastereomers (i.e., D-separation = absolute difference between D-*allo*-Ile and D-Ile). The Average Δt_R values are the average difference in retention time between the extracted ion and variable wavelength detector chromatograms.

	m/z Δt_R (min)	VWD Δt_R (min)	Average Δt_R (min)
allo-Ile Separation	2.328	2.307	2.32
Ile Separation	2.328	2.307	2.32
L-Separation	0	0	0
D-Separation	0	0	0

LC/MS Traces for the Attempted Resolution of the Four Stereoisomers of Isoleucine with 1-Fluoro-2,4-dinitrophenyl-5-L-valine Amide (i.e., L-FDVA 15) with HPLC Method A (i.e., 25 min.)

Total Ion Mass Chromatogram (ESI-ToF). The Y-axis is ion counts, and the X-axis is acquisition time in minutes.

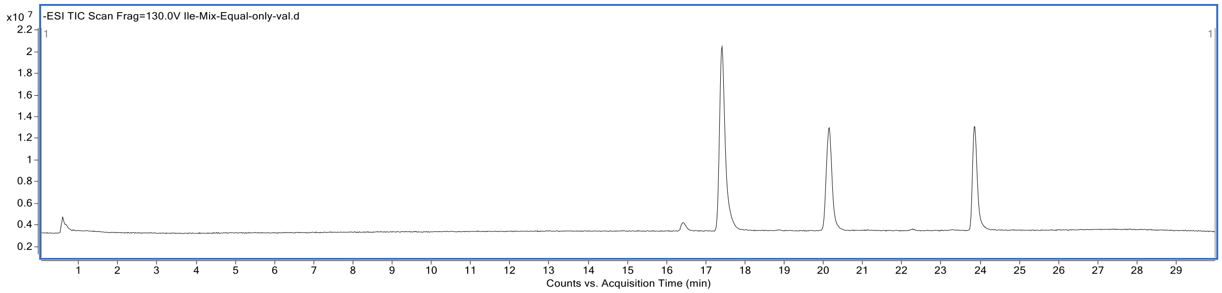

Variable Wavelength Detector Chromatogram (340 nm). The Y-axis is absorbance units, and the X-axis is acquisition time in minutes.

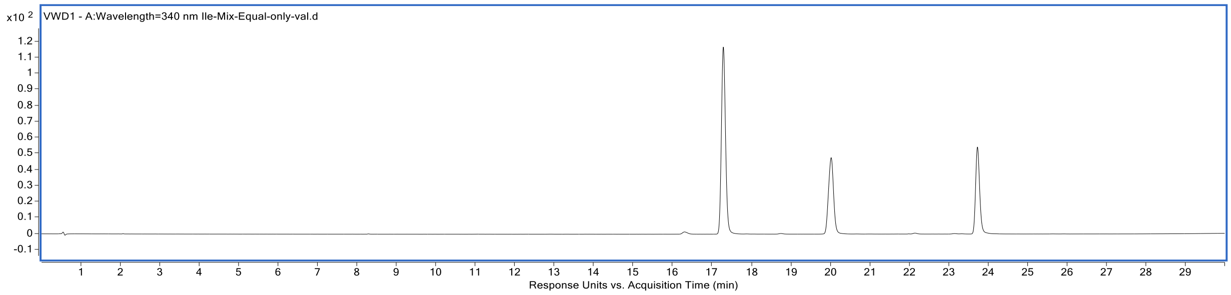

Extracted Ion Mass Chromatogram (ESI-ToF, extracted for m/z 410 ± 0.5). The Y-axis is ion counts, and the X-axis is acquisition time in minutes.

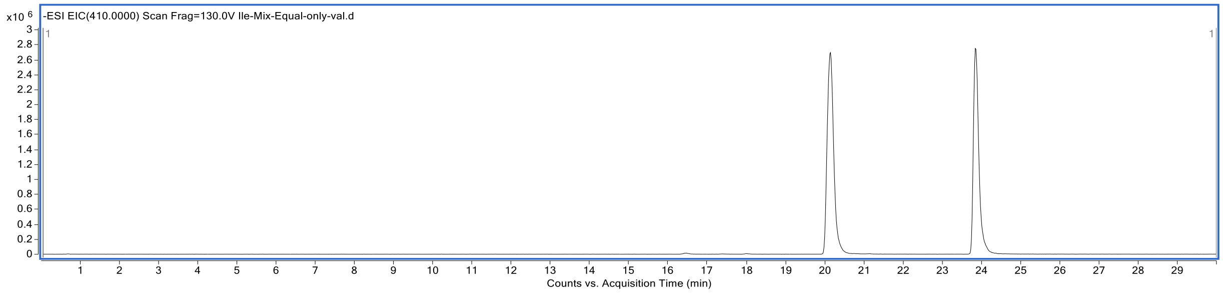

Zoomed and rescaled Extracted Ion (top) and Variable Wavelength Detector (bottom) Chromatograms. The Y axis on top is ion counts, the Y-axis on the bottom is absorbance units, and the X-axis for both is acquisition time in minutes. The peaks are labeled from left to right as Peak 1 and Peak 2.

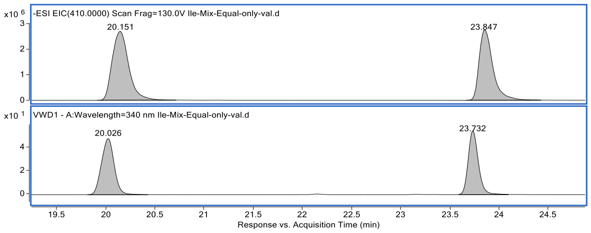

The following is a table of the peak data from the mass chromatogram. The Area % is the percent area relative to the tallest integrated peak, which has been set to 100%.

Peak	Assignment	t_R (min)	ES-ToF m/z [Neg]	Area %
1	L-*allo*-Ile/ L-Ile	20.151	410.1710	100
2	D-*allo*-Ile/ D-Ile	23.847	410.1698	85.24

The calculated m/z for the adduct of isoleucine with the Marfey's reagent is 410.1681 for $C_{17}H_{24}N_5O_7^-$ $[M - H]^{-1}$.

The following is a table of the peak data from the variable wavelength detector chromatogram (340 nm). The Area % is the percent area relative to the tallest integrated peak, which has been set to 100%.

Peak	Assignment	t_R (min)	Area %
1	L-*allo*-Ile/ L-Ile	20.026	100
2	D-*allo*-Ile/ D-Ile	23.732	91.02

The following is a table showing the difference in retention time on the extracted ion chromatogram (i.e., m/z Δt_R) and variable wavelength detector (i.e., VWD Δt_R) between the *allo*-Ile diastereomers (i.e., *allo*-Ile separation = absolute difference between L-*allo*-Ile and D-*allo*-Ile), the canonical isoleucine diastereomers (i.e., Ile separation = absolute difference between L-Ile and D-Ile), alpha-L-diastereomers (i.e., L-separation = absolute difference between L-*allo*-Ile and L-Ile), and the alpha-D-diastereomers (i.e., D-separation = absolute difference between D-*allo*-Ile and D-Ile). The Average Δt_R values are the average difference in retention time between the extracted ion and variable wavelength detector chromatograms.

	m/z Δt_R (min)	VWD Δt_R (min)	Average Δt_R (min)
allo-Ile Separation	3.696	3.706	3.70
Ile Separation	3.696	3.706	3.70
L-Separation	0	0	0
D-Separation	0	0	0

LC/MS Traces for the Attempted Resolution of the Four Stereoisomers of Isoleucine with 1-Fluoro-2,4-dinitrophenyl-5-L-isoleucine Amide (i.e., L-FDIA 16) with HPLC Method A (i.e., 25 min.)

Total Ion Mass Chromatogram (ESI-ToF). The Y-axis is ion counts, and the X-axis is acquisition time in minutes.

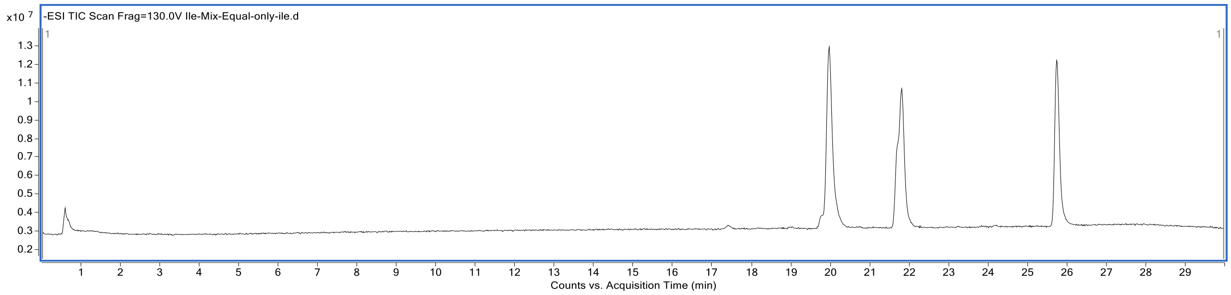

Variable Wavelength Detector Chromatogram (340 nm). The Y-axis is absorbance units, and the X-axis is acquisition time in minutes.

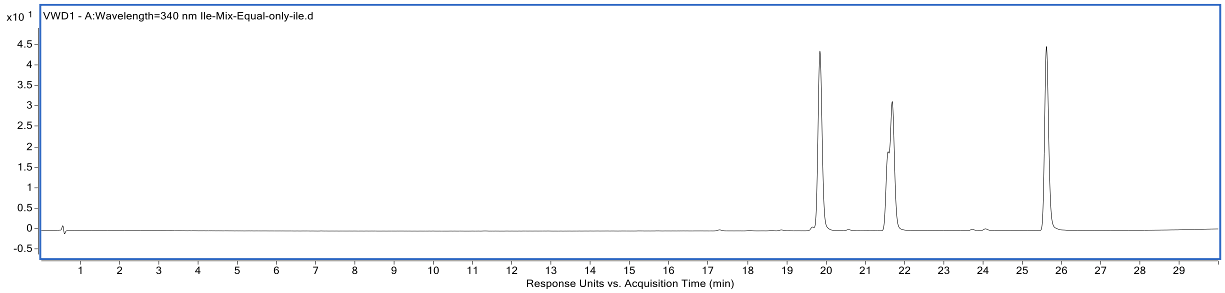

Extracted Ion Mass Chromatogram (ESI-ToF, extracted for m/z 424 ± 0.5). The Y-axis is ion counts, and the X-axis is acquisition time in minutes.

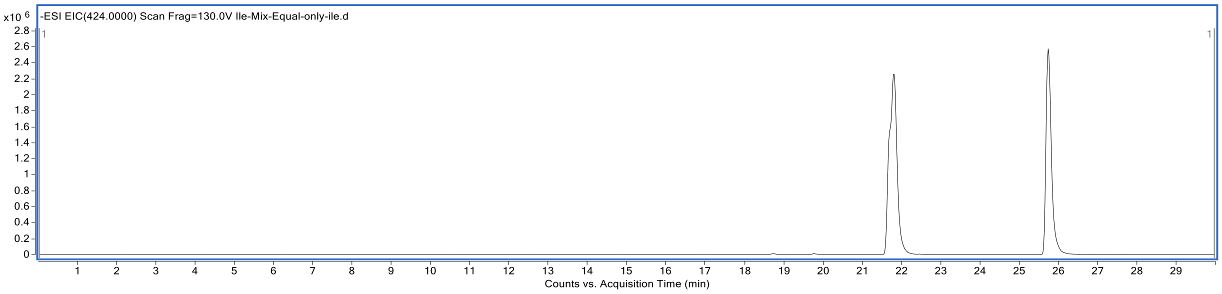

Zoomed and rescaled Extracted Ion (top) and Variable Wavelength Detector (bottom) Chromatograms. The Y axis on top is ion counts, the Y-axis on the bottom is absorbance units, and the X-axis for both is acquisition time in minutes. The peaks are labeled from left to right as Peak 1, Peak 2, and Peak 3.

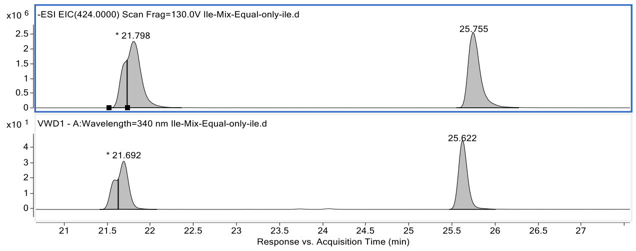

The following is a table of the peak data from the mass chromatogram. The Area % is the percent area relative to the tallest integrated peak, which has been set to 100%.

Peak	Assignment	t_R (min)	ES-ToF m/z [Neg]	Area %
1	L-Ile	21.699	424.1867	34
2	L-*allo*-Ile	21.798	424.1868	91
3	D-*allo*-Ile, D-Ile	25.755	424.1862	100

The calculated m/z for the adduct of isoleucine with the Marfey's reagent is 424.1838 for $C_{18}H_{26}N_5O_7^-$ $[M - H]^{-1}$.

The following is a table of the peak data from the variable wavelength detector chromatogram (340 nm). The Area % is the percent area relative to the tallest integrated peak, which has been set to 100%.

Peak	Assignment	t_R (min)	Area %
1	L-Ile	21.583	33.68
2	L-*allo*-Ile	21.692	71.97
3	D-*allo*-Ile, D-Ile	25.622	100

The following is a table showing the difference in retention time on the extracted ion chromatogram (i.e., m/z Δt_R) and variable wavelength detector (i.e., VWD Δt_R) between the alpha amino diastereomers (i.e., The Ile separation was determined from the difference in t_R between peaks 2 and 3, which equals absolute difference between averaged L-Ile t_R and average D-Ile t_R), alpha-L-diastereomers (i.e., L-separation = absolute difference between L-*allo*-Ile and L-Ile), and the alpha-D-diastereomers (i.e., D-separation = absolute difference between D-*allo*-Ile and D-Ile). The Average Δt_R values are the average difference in retention time between the extracted ion and variable wavelength detector chromatograms.

	m/z Δt_R (min)	VWD Δt_R (min)	Average Δt_R (min)
allo-Ile Separation	3.957	3.930	3.94
Ile Separation	4.056	4.039	4.05
L-Separation	0.099	0.109	0.10
D-Separation	0	0	0

LC/MS Traces for the Attempted Resolution of the Four Stereoisomers of Isoleucine with 1-Fluoro-2,4-dinitrophenyl-5-L-threonine Amide (i.e., L-FDTA 17) with HPLC Method A (i.e., 25 min.)

Total Ion Mass Chromatogram (ESI-ToF). The Y-axis is ion counts, and the X-axis is acquisition time in minutes.

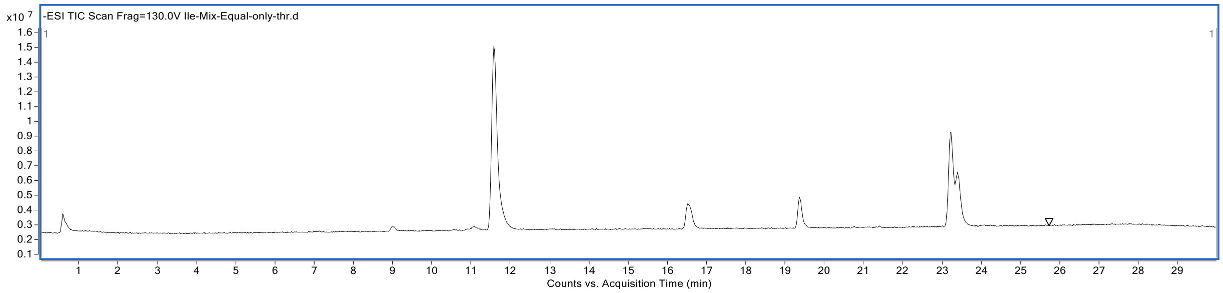

Variable Wavelength Detector Chromatogram (340 nm). The Y-axis is absorbance units, and the X-axis is acquisition time in minutes.

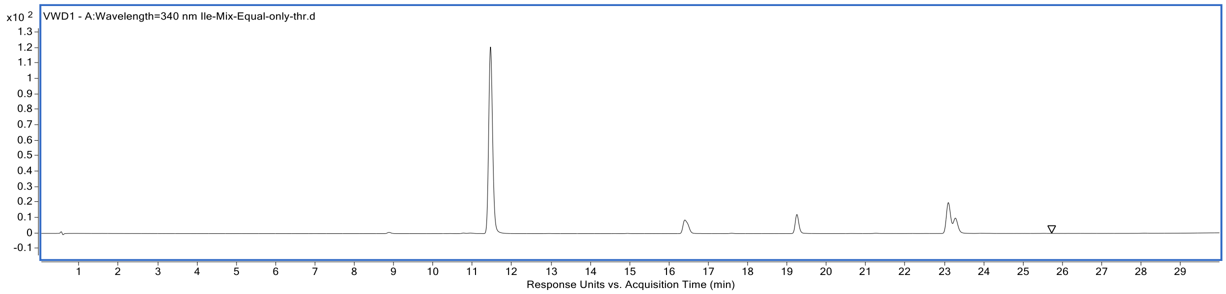

Extracted Ion Mass Chromatogram (ESI-ToF, extracted for m/z 412 ± 0.5). The Y-axis is ion counts, and the X-axis is acquisition time in minutes.

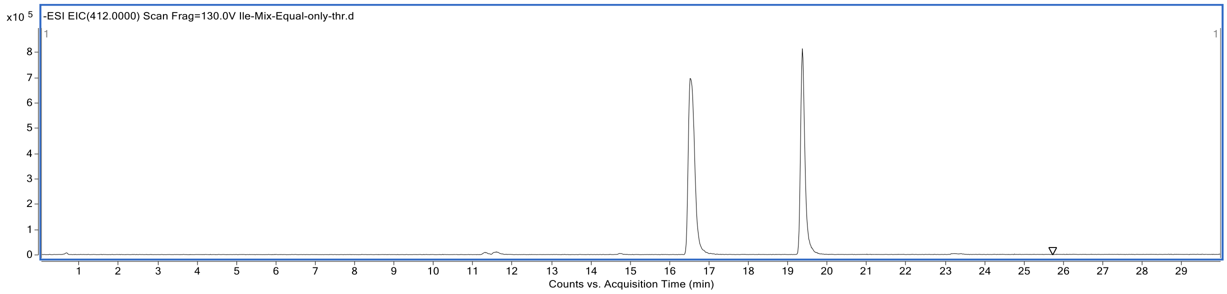

Zoomed and rescaled Extracted Ion (top) and Variable Wavelength Detector (bottom) Chromatograms. The Y axis on top is ion counts, the Y-axis on the bottom is absorbance units, and the X-axis for both is acquisition time in minutes. The peaks are labeled from left to right as Peak 1 and Peak 2.

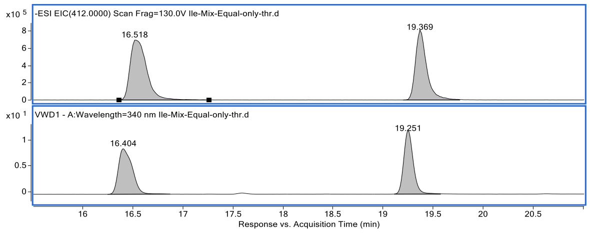

The following is a table of the peak data from the mass chromatogram. The Area % is the percent area relative to the tallest integrated peak, which has been set to 100%.

Peak	Assignment	t_R (min)	ES-ToF m/z [Neg]	Area %
1	L-*allo*-Ile/ L-Ile	16.518	412.1502	100
2	D-*allo*-Ile/ D-Ile	19.396	412.1502	79.08

The calculated m/z for the adduct of isoleucine with the Marfey's reagent is 412.1474 for $C_{16}H_{22}N_5O_8^-$ $[M - H]^{-1}$.

The following is a table of the peak data from the variable wavelength detector chromatogram (340 nm). The Area % is the percent area relative to the tallest integrated peak, which has been set to 100%.

Peak	Assignment	t_R (min)	Area %
1	L-*allo*-Ile/ L-Ile	16.404	100
2	D-*allo*-Ile/ D-Ile	19.251	95.84

The following is a table showing the difference in retention time on the extracted ion chromatogram (i.e., m/z Δt_R) and variable wavelength detector (i.e., VWD Δt_R) between the *allo*-Ile diastereomers (i.e., *allo*-Ile separation = absolute difference between L-*allo*-Ile and D-*allo*-Ile), the canonical isoleucine diastereomers (i.e., Ile separation = absolute difference between L-Ile and D-Ile), alpha-L-diastereomers (i.e., L-separation = absolute difference between L-*allo*-Ile and L-Ile), and the alpha-D-diastereomers (i.e., D-separation = absolute difference between D-*allo*-Ile and D-Ile). The Average Δt_R values are the average difference in retention time between the extracted ion and variable wavelength detector chromatograms.

	m/z Δt_R (min)	VWD Δt_R (min)	Average Δt_R (min)
allo-Ile Separation	2.851	2.847	2.85
Ile Separation	2.851	2.847	2.85
L-Separation	0	0	0
D-Separation	0	0	0

LC/MS Traces for the Attempted Resolution of the Four Stereoisomers of Isoleucine with 1-Fluoro-2,4-dinitrophenyl-5-L-phenylalanine Amide (i.e., L-FDFA 18) with HPLC Method A (i.e., 25 min.)

Total Ion Mass Chromatogram (ESI-ToF). The Y-axis is ion counts, and the X-axis is acquisition time in minutes.

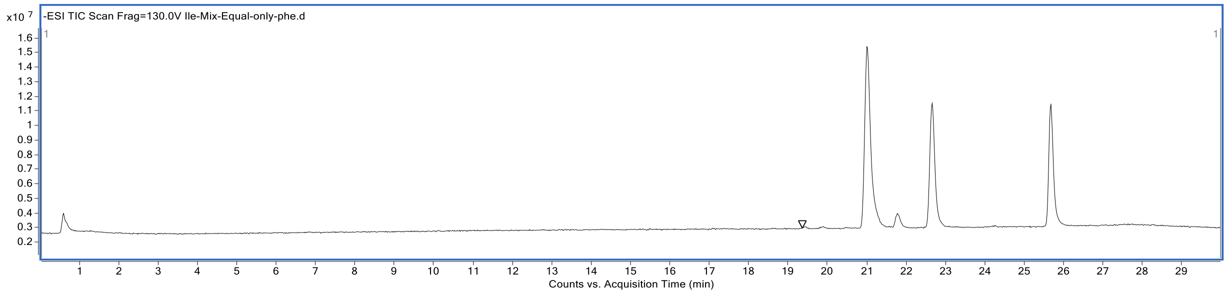

Variable Wavelength Detector Chromatogram (340 nm). The Y-axis is absorbance units, and the X-axis is acquisition time in minutes.

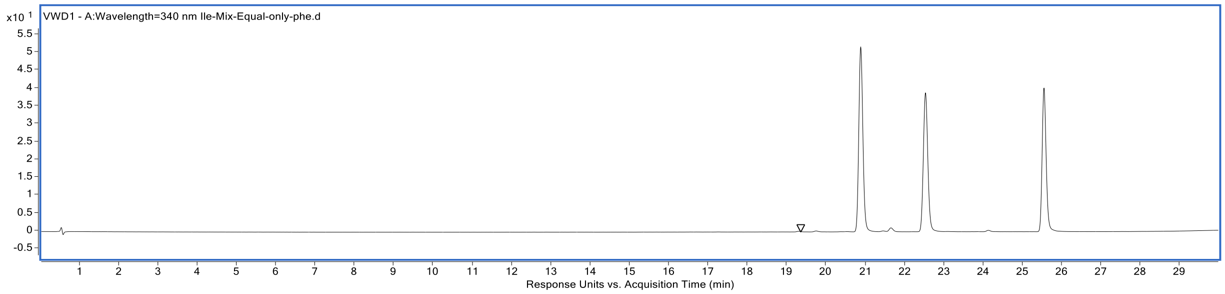

Extracted Ion Mass Chromatogram (ESI-ToF, extracted for m/z 458 ± 0.5). The Y-axis is ion counts, and the X-axis is acquisition time in minutes.

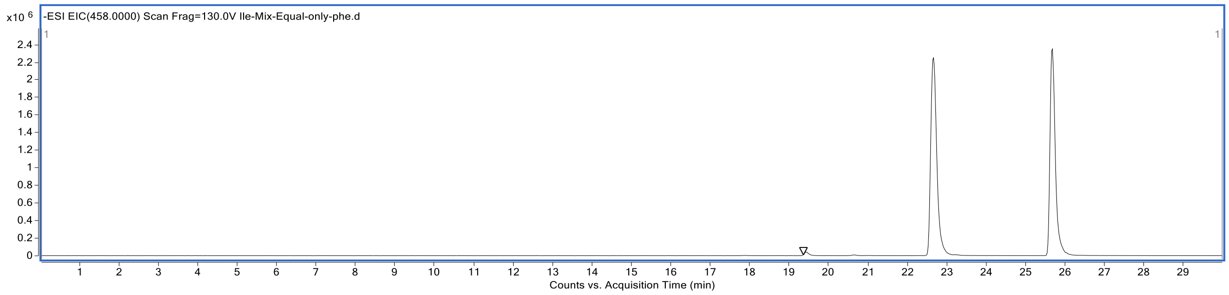

Zoomed and rescaled Extracted Ion (top) and Variable Wavelength Detector (bottom) Chromatograms. The Y axis on top is ion counts, the Y-axis on the bottom is absorbance units, and the X-axis for both is acquisition time in minutes. The peaks are labeled from left to right as Peak 1 and Peak 2.

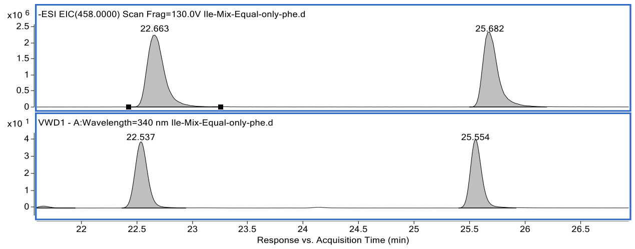

The following is a table of the peak data from the mass chromatogram. The Area % is the percent area relative to the tallest integrated peak, which has been set to 100%.

Peak	Assignment	t_R (min)	ES-ToF m/z [Neg]	Area %
1	L-*allo*-Ile/ L-Ile	22.663	458.1713	100
2	D-*allo*-Ile/ D-Ile	25.682	458.1720	91.55

The calculated m/z for the adduct of isoleucine with the Marfey's reagent is 458.1681 for $C_{21}H_{24}N_5O_7^-$ $[M – H]^{-1}$.

The following is a table of the peak data from the variable wavelength detector chromatogram (340 nm). The Area % is the percent area relative to the tallest integrated peak, which has been set to 100%.

Peak	Assignment	t_R (min)	Area %
1	L-*allo*-Ile/ L-Ile	22.537	100
2	D-*allo*-Ile/ D-Ile	25.554	93.16

The following is a table showing the difference in retention time on the extracted ion chromatogram (i.e., m/z Δt_R) and variable wavelength detector (i.e., VWD Δt_R) between the *allo*-Ile diastereomers (i.e., *allo*-Ile separation = absolute difference between L-*allo*-Ile and D-*allo*-Ile), the canonical isoleucine diastereomers (i.e., Ile separation = absolute difference between L-Ile and D-Ile), alpha-L-diastereomers (i.e., L-separation = absolute difference between L-*allo*-Ile and L-Ile), and the alpha-D-diastereomers (i.e., D-separation = absolute difference between D-*allo*-Ile and D-Ile). The Average Δt_R values are the average difference in retention time between the extracted ion and variable wavelength detector chromatograms.

	m/z Δt_R (min)	VWD Δt_R (min)	Average Δt_R (min)
allo-Ile Separation	3.019	3.017	3.02
Ile Separation	3.019	3.017	3.02
L-Separation	0	0	0
D-Separation	0	0	0

LC/MS Traces for the Attempted Resolution of the Four Stereoisomers of Isoleucine with 1-Fluoro-2,4-dinitrophenyl-5-L-tryptophan Amide (i.e., L-FDWA 19) with HPLC Method A (i.e., 25 min.)

Total Ion Mass Chromatogram (ESI-ToF). The Y-axis is ion counts, and the X-axis is acquisition time in minutes.

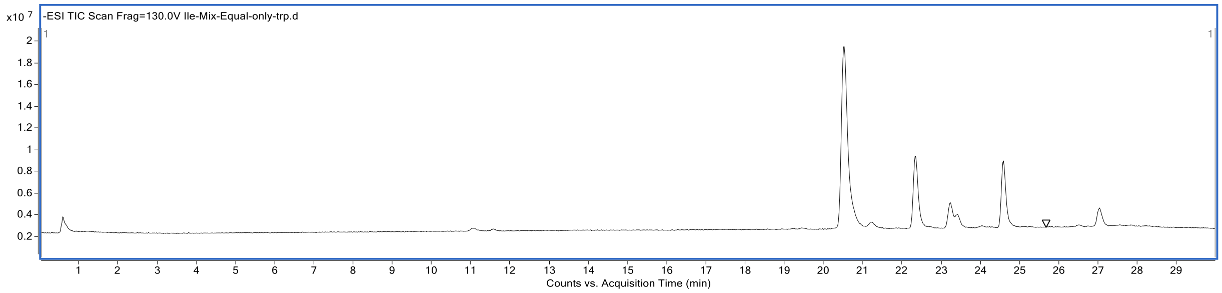

Variable Wavelength Detector Chromatogram (340 nm). The Y-axis is absorbance units, and the X-axis is acquisition time in minutes.

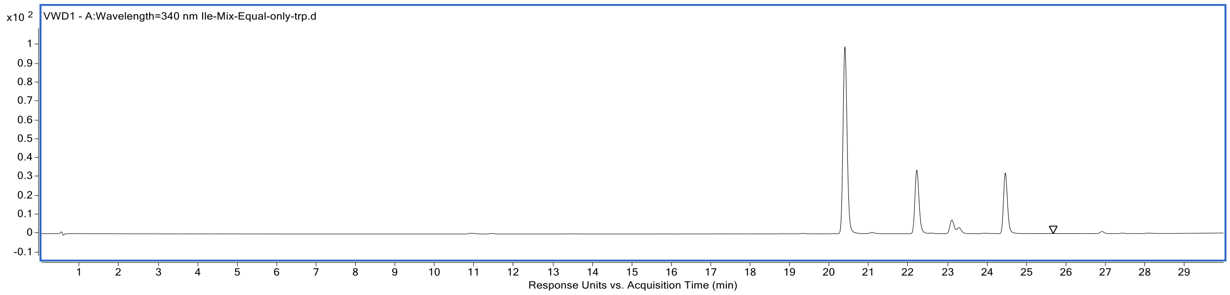

Extracted Ion Mass Chromatogram (ESI-ToF, extracted for m/z 497 ± 0.5). The Y-axis is ion counts, and the X-axis is acquisition time in minutes.

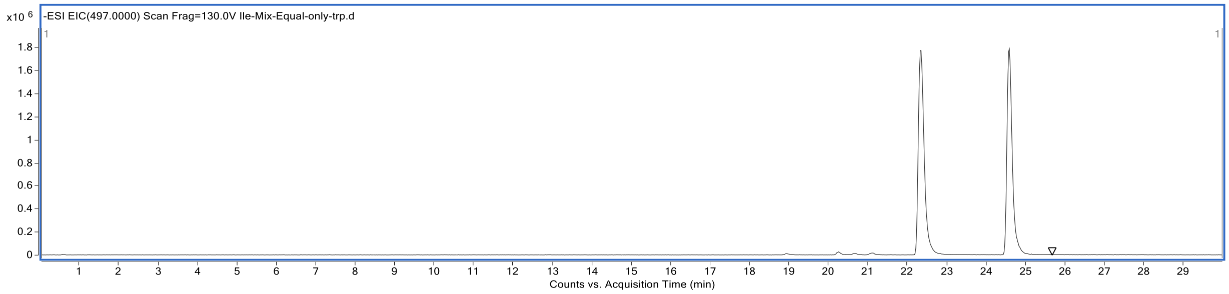

Zoomed and rescaled Extracted Ion (top) and Variable Wavelength Detector (bottom) Chromatograms. The Y axis on top is ion counts, the Y-axis on the bottom is absorbance units, and the X-axis for both is acquisition time in minutes. The peaks are labeled from left to right as Peak 1 and Peak 2.

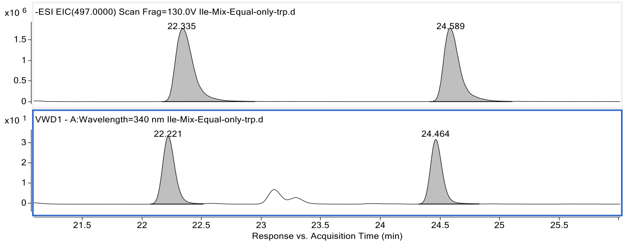

The following is a table of the peak data from the mass chromatogram. The Area % is the percent area relative to the tallest integrated peak, which has been set to 100%.

Peak	Assignment	t_R (min)	ES-ToF m/z [Neg]	Area %
1	L-*allo*-Ile/ L-Ile	22.335	497.1816	100
2	D-*allo*-Ile/ D-Ile	24.589	497.1818	91.18

The calculated m/z for the adduct of isoleucine with the Marfey's reagent is 497.1790 for $C_{23}H_{25}N_6O_7^-$ $[M - H]^{-1}$.

The following is a table of the peak data from the variable wavelength detector chromatogram (340 nm). The Area % is the percent area relative to the tallest integrated peak, which has been set to 100%.

Peak	Assignment	t_R (min)	Area %
1	L-*allo*-Ile/ L-Ile	22.221	100
2	D-*allo*-Ile/ D-Ile	24.464	91.50

The following is a table showing the difference in retention time on the extracted ion chromatogram (i.e., m/z Δt_R) and variable wavelength detector (i.e., VWD Δt_R) between the *allo*-Ile diastereomers (i.e., *allo*-Ile separation = absolute difference between L-*allo*-Ile and D-*allo*-Ile), the canonical isoleucine diastereomers (i.e., Ile separation = absolute difference between L-Ile and D-Ile), alpha-L-diastereomers (i.e., L-separation = absolute difference between L-*allo*-Ile and L-Ile), and the alpha-D-diastereomers (i.e., D-separation = absolute difference between D-*allo*-Ile and D-Ile). The Average Δt_R values are the average difference in retention time between the extracted ion and variable wavelength detector chromatograms.

	m/z Δt_R (min)	VWD Δt_R (min)	Average Δt_R (min)
allo-Ile Separation	2.254	2.234	2.24
Ile Separation	2.254	2.234	2.24
L-Separation	0	0	0
D-Separation	0	0	0

LC/MS Traces for the Attempted Resolution of the Four Stereoisomers of Isoleucine with 1-Fluoro-2,4-dinitrophenyl-5-L-proline Amide (i.e., L-FDPA 9) with HPLC Method A (i.e., 25 min.)

Total Ion Mass Chromatogram (ESI-ToF). The Y-axis is ion counts, and the X-axis is acquisition time in minutes.

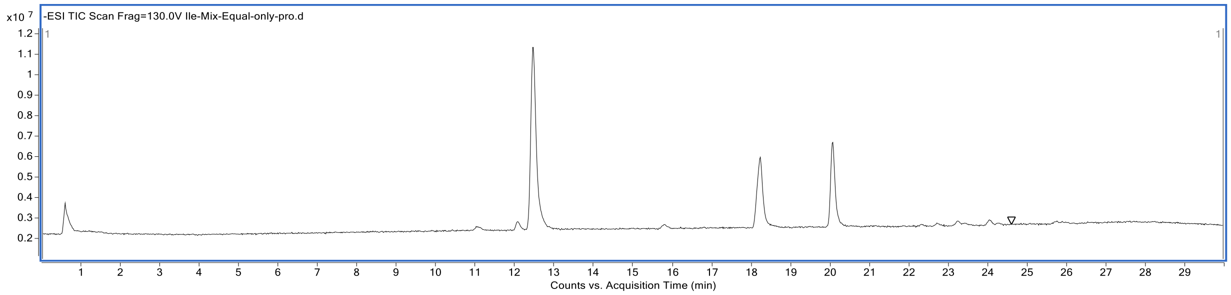

Variable Wavelength Detector Chromatogram (340 nm). The Y-axis is absorbance units, and the X-axis is acquisition time in minutes.

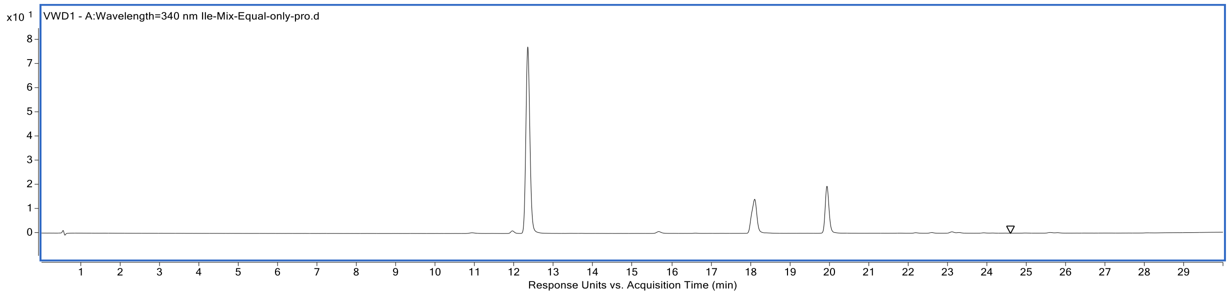

Extracted Ion Mass Chromatogram (ESI-ToF, extracted for m/z 408 ± 0.5). The Y-axis is ion counts, and the X-axis is acquisition time in minutes.

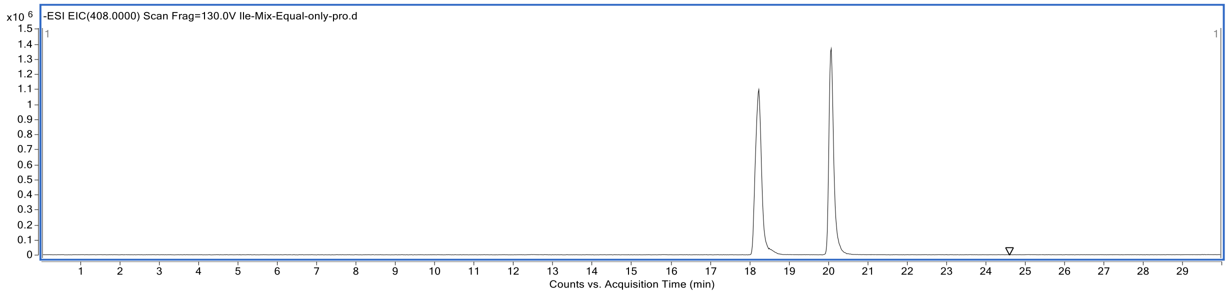

Zoomed and rescaled Extracted Ion (top) and Variable Wavelength Detector (bottom) Chromatograms. The Y axis on top is ion counts, the Y-axis on the bottom is absorbance units, and the X-axis for both is acquisition time in minutes. The peaks are labeled from left to right as Peak 1 and Peak 2. The adducts comprising Peak 1 can be seen from the peak tailing, but the resolution is not sufficient to evaluate isomer separation.

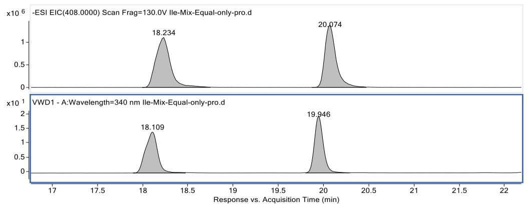

The following is a table of the peak data from the mass chromatogram. The Area % is the percent area relative to the tallest integrated peak, which has been set to 100%.

Peak	Assignment	t_R (min)	ES-ToF m/z [Neg]	Area %
1	L-*allo*-Ile/ L-Ile	18.234	408.1547	100
2	D-*allo*-Ile/ D-Ile	20.074	408.1552	96.43

The calculated m/z for the adduct of isoleucine with the Marfey's reagent is 408.1525 for $C_{17}H_{22}N_5O_7^-$ $[M - H]^{-1}$.

The following is a table of the peak data from the variable wavelength detector chromatogram (340 nm). The Area % is the percent area relative to the tallest integrated peak, which has been set to 100%.

Peak	Assignment	t_R (min)	Area %
1	L-*allo*-Ile/ L-Ile	18.109	99.48
2	D-*allo*-Ile/ D-Ile	19.946	100

The following is a table showing the difference in retention time on the extracted ion chromatogram (i.e., m/z Δt_R) and variable wavelength detector (i.e., VWD Δt_R) between the *allo*-Ile diastereomers (i.e., *allo*-Ile separation = absolute difference between L-*allo*-Ile and D-*allo*-Ile), the canonical isoleucine diastereomers (i.e., Ile separation = absolute difference between L-Ile and D-Ile), alpha-L-diastereomers (i.e., L-separation = absolute difference between L-*allo*-Ile and L-Ile), and the alpha-D-diastereomers (i.e., D-separation = absolute difference between D-*allo*-Ile and D-Ile). The Average Δt_R values are the average difference in retention time between the extracted ion and variable wavelength detector chromatograms.

	m/z Δt_R (min)	VWD Δt_R (min)	Average Δt_R (min)
allo-Ile Separation	1.840	1.837	1.84
Ile Separation	1.840	1.837	1.84
L-Separation	0	0	0
D-Separation	0	0	0

LC/MS Traces for the Attempted Resolution of L-Isoleucine and L-*allo*-Isoleucine with 1-Fluoro-2,4-dinitrobenzene (i.e., Sanger's Reagent 10) with HPLC Method A (i.e., 25 min.)

Total Ion Mass Chromatogram (ESI-ToF). The Y-axis is ion counts, and the X-axis is acquisition time in minutes.

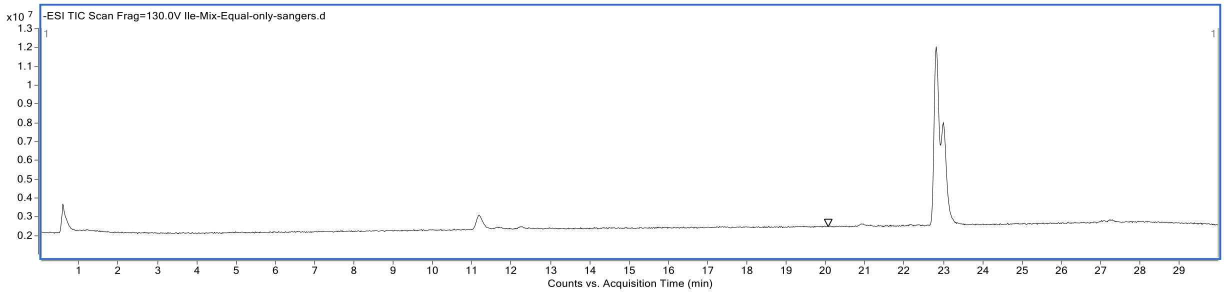

Variable Wavelength Detector Chromatogram (340 nm). The Y-axis is absorbance units, and the X-axis is acquisition time in minutes.

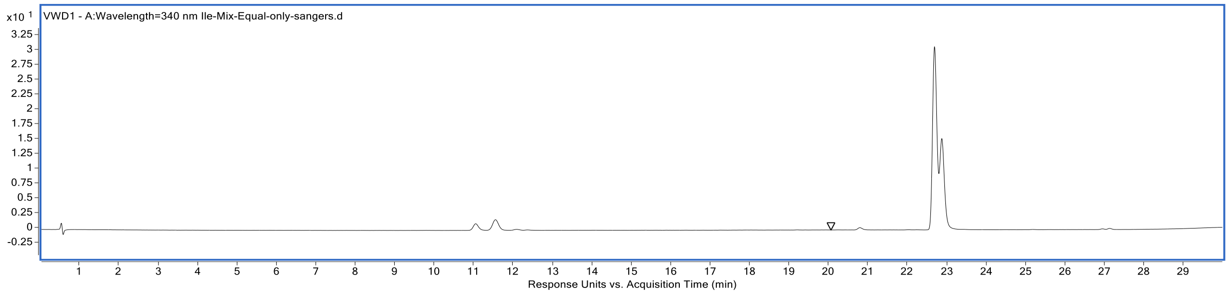

Extracted Ion Mass Chromatogram (ESI-ToF, extracted for m/z 296 ± 0.5). The Y-axis is ion counts, and the X-axis is acquisition time in minutes.

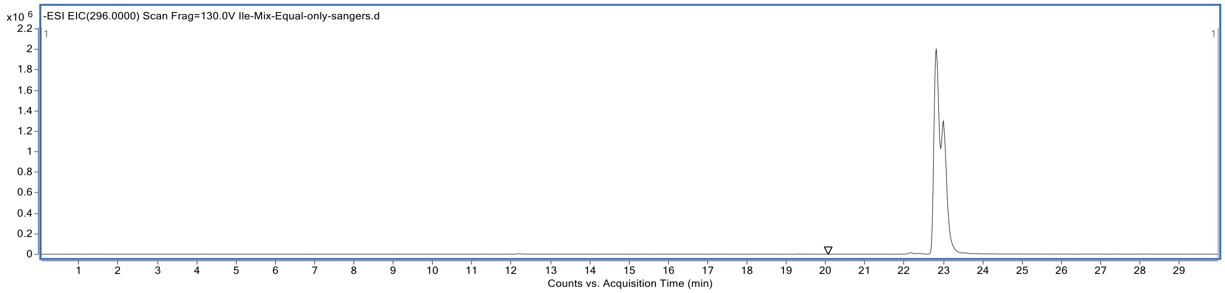

Zoomed and rescaled Extracted Ion (top) and Variable Wavelength Detector (bottom) Chromatograms. The Y axis on top is ion counts, the Y-axis on the bottom is absorbance units, and the X-axis for both is acquisition time in minutes. The peaks are labeled from left to right as Peak 1 and Peak 2.

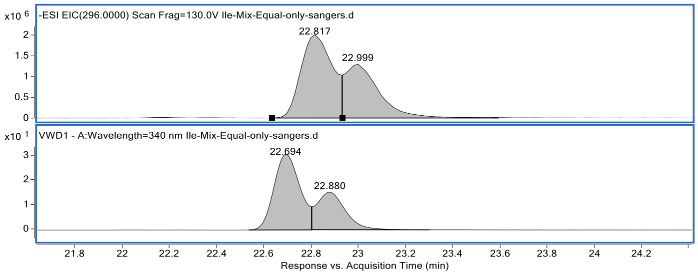

The following is a table of the peak data from the mass chromatogram. The Area % is the percent area relative to the tallest integrated peak, which has been set to 100%.

Peak	Assignment	t_R (min)	ES-ToF m/z [Neg]	Area %
1	D,L-*allo*-Ile	22.817	296.0909	100
2	D,L-Ile	22.999	296.0908	70.28

The calculated m/z for the adduct of isoleucine with Sanger's reagent is 296.0888 for $C_{12}H_{14}N_3O_6^-$ $[M - H]^{-1}$.

The following is a table of the peak data from the variable wavelength detector chromatogram (340 nm). The Area % is the percent area relative to the tallest integrated peak, which has been set to 100%.

Peak	Assignment	t_R (min)	Area %
1	D,L-*allo*-Ile	22.694	100
2	D,L-Ile	22.880	54.30

The following is a table showing the difference in retention time on the extracted ion chromatogram (i.e., m/z Δt_R) and variable wavelength detector (i.e., VWD Δt_R) between the two diastereomers (i.e., separation = absolute difference between Peak 1 and Peak 2). The Average Δt_R values are the average difference in retention time between the extracted ion and variable wavelength detector chromatograms.

	m/z Δt_R (min)	VWD Δt_R (min)	Average Δt_R (min)
Peak Separation	0.182	0.186	0.18

LC/MS Traces for the Attempted Resolution of L-Isoleucine and L-*allo*-Isoleucine After Fmoc Derivatization to Make 2-((((9*H*-Fluoren-9-yl)methoxy)carbonyl)amino)-3-hydroxy-3-phenylpropanoic Acid (*N*-Fmoc L-Ile and L-*allo*-Ile) with HPLC Method A (i.e., 25 min.)

Total Ion Mass Chromatogram (ESI-ToF). The Y-axis is ion counts, and the X-axis is acquisition time in minutes.

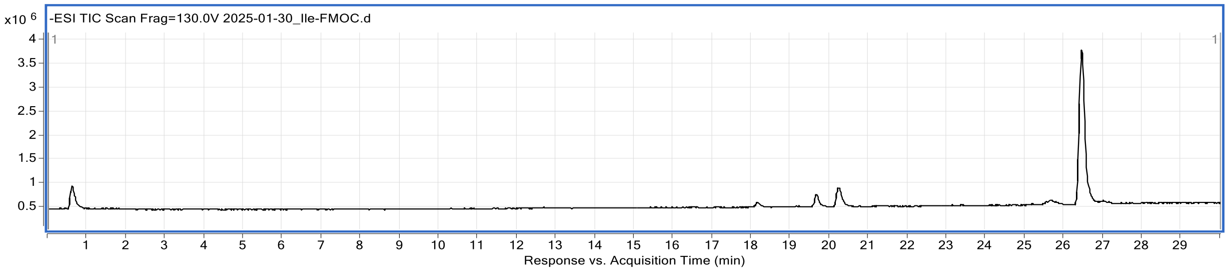

Variable Wavelength Detector Chromatogram (300 nm). The Y-axis is absorbance units, and the X-axis is acquisition time in minutes.

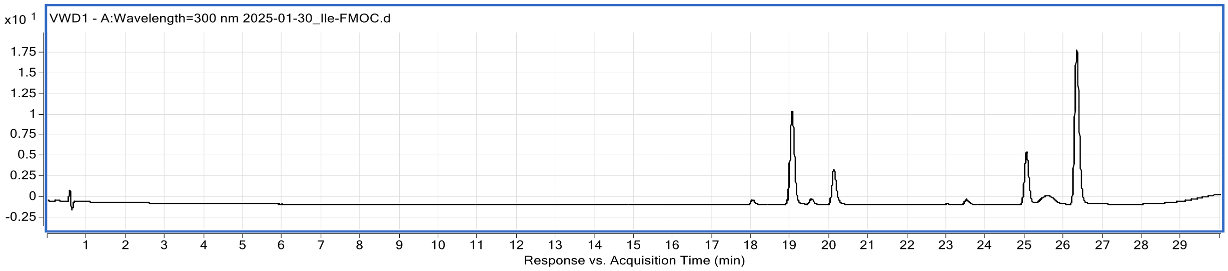

Extracted Ion Mass Chromatogram (ESI-ToF, extracted for m/z 352 ± 0.5). The Y-axis is ion counts, and the X-axis is acquisition time in minutes.

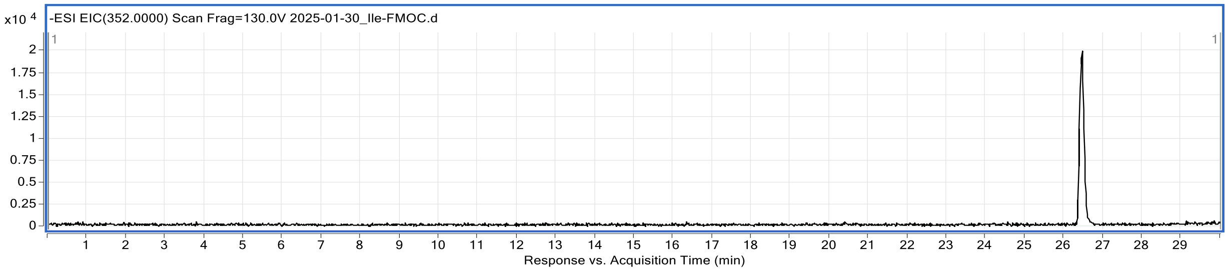

Zoomed and rescaled Extracted Ion (top) and Variable Wavelength Detector (bottom) Chromatograms. The Y axis on top is ion counts, the Y-axis on the bottom is absorbance units, and the X-axis for both is acquisition time in minutes.

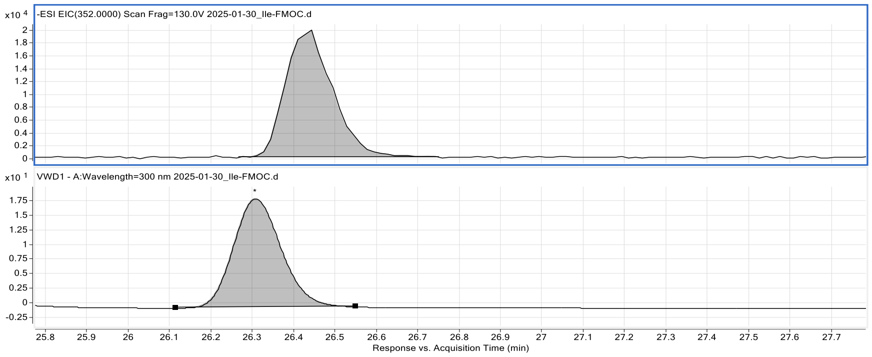

The following is a table of the peak data from the mass chromatogram. The Area % is the percent area relative to the tallest integrated peak, which has been set to 100%.

Peak	Assignment	t_R (min)	ES-ToF m/z [Neg]	Area %
1	L-Ile and L-*allo*-Ile	26.443	352.1563	100

The calculated m/z for the adduct of isoleucine after Fmoc derivatization is 352.1554 for $C_{21}H_{22}NO_4^-$ $[M - H]^{-1}$.

The following is a table of the peak data from the variable wavelength detector chromatogram (300 nm). The Area % is the percent area relative to the tallest integrated peak, which has been set to 100%.

Peak	Assignment	t_R (min)	Area %
1	L-Ile and L-*allo*-Ile	26.307	100

The following is a table showing the difference in retention time on the extracted ion chromatogram (i.e., m/z Δt_R) and variable wavelength detector (i.e., VWD Δt_R) between the two diastereomers (i.e., separation = absolute difference between Peak 1 and Peak 2). The Average Δt_R values are the average difference in retention time between the extracted ion and variable wavelength detector chromatograms.

	m/z Δt_R (min)	VWD Δt_R (min)	Average Δt_R (min)
Peak Separation	0	0	0

III-H. Separation of Various Mixtures of L-*allo*-Isoleucine and L-Isoleucine

LC/MS Traces for the Attempted Resolution of a 1:1 Mixture of L-*allo*-Isoleucine and L-Isoleucine After Derivatization with 1-Fluoro-2,4-dinitrobenzene (i.e., Sanger's Reagent 10) with HPLC Method C (i.e., 30% aq. MeCN isocratic, 30 min.)

A 1:1 mixture of L-*allo*-Ile and L-Ile (total amino acid concentration = 10 mM) was derivatized with Sanger's reagent under the standard Marfey's derivatization protocol (cf. General Procedure B, replacing phenylserine with the amino acid solution described above). The HPLC method used to resolve the derivatized amino acids was isocratic 30% MeCN in H_2O + 0.1% HCO_2H over 30 minutes with a flow rate 0.35 mL min^{-1} and using an Agilent Zorbax Extend-C18 column, 2.1 x 50 mm (1.8 µm). The Agilent column part number is 727700-902.

Total Ion Mass Chromatogram (ESI-ToF). The Y-axis is ion counts, and the X-axis is acquisition time in minutes.

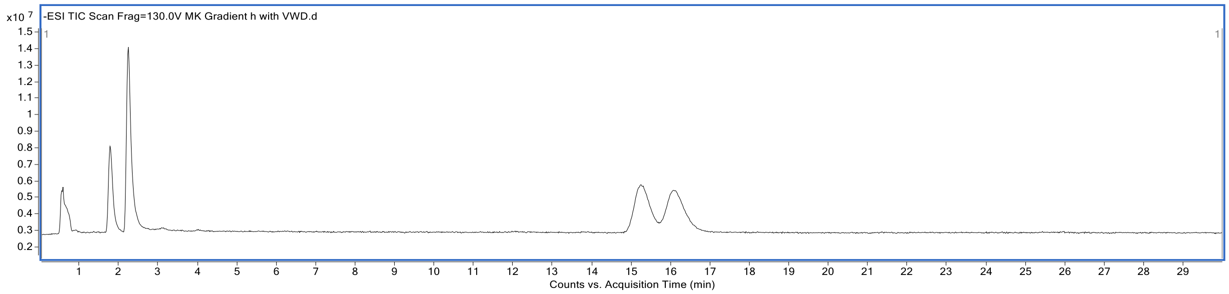

Variable Wavelength Detector Chromatogram (340 nm). The Y-axis is absorbance units, and the X-axis is acquisition time in minutes.

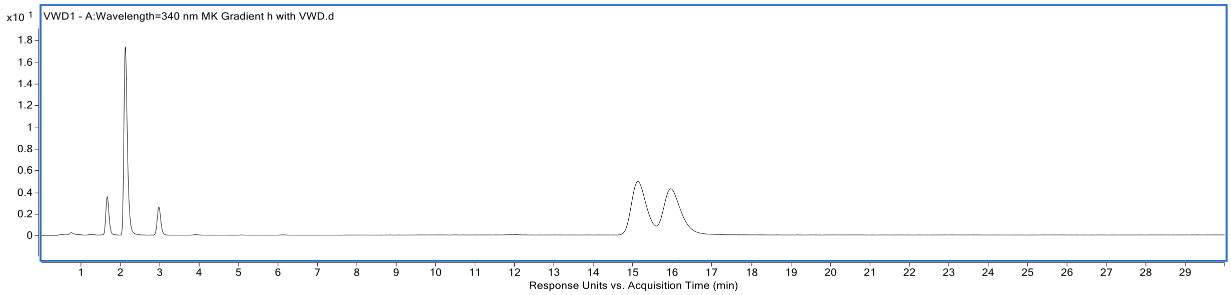

Extracted Ion Mass Chromatogram (ESI-ToF, extracted for m/z 296 ± 0.5). The Y-axis is ion counts, and the X-axis is acquisition time in minutes.

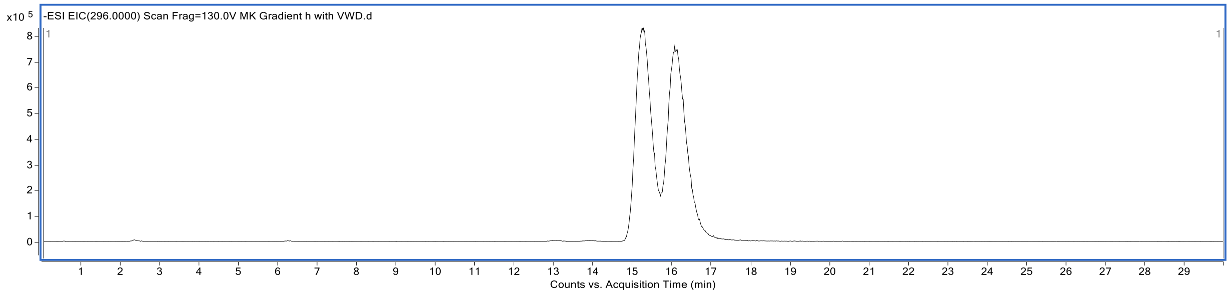

Zoomed and rescaled Extracted Ion (top) and Variable Wavelength Detector (bottom) Chromatograms. The Y axis on top is ion counts, the Y-axis on the bottom is absorbance units, and the X-axis for both is acquisition time in minutes. The peaks are labeled from left to right as Peak 1 and Peak 2.

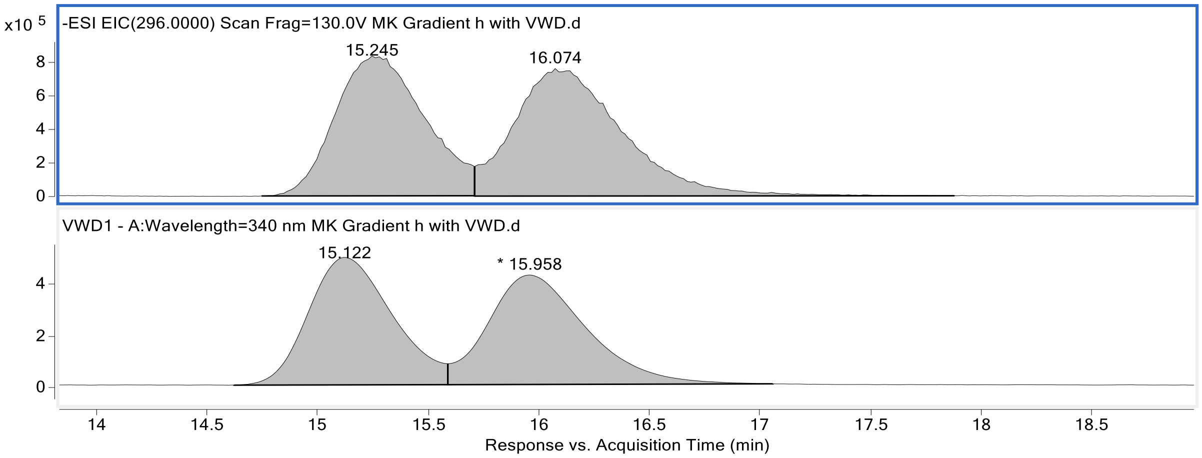

The following is a table of the peak data from the mass chromatogram. The Area % is the percent area relative to the tallest integrated peak, which has been set to 100%.

Peak	Assignment	t_R (min)	ES-ToF m/z [Neg]	Area %
1	L-*allo*-Ile	15.245	296.0897	90.49
2	L-Ile	16.074	296.0892	100

The calculated m/z for the adduct of isoleucine with Sanger's reagent is 296.0888 for $C_{12}H_{14}N_3O_6^-$ $[M - H]^{-1}$.

The following is a table of the peak data from the variable wavelength detector chromatogram (340 nm). The Area % is the percent area relative to the tallest integrated peak, which has been set to 100%.

Peak	Assignment	t_R (min)	Area %
1	L-*allo*-Ile	15.122	99.45
2	L-Ile	15.958	100

The following is a table showing the difference between the experimental ratio of L-*allo*-Ile to L-Ile and the ratio measured by HPLC using extracted ion mass spectrometry (i.e., m/z HPLC Ratio) and 340 nm variable wavelength (i.e., VWD HPLC Ratio) detection. The mixture ratio was set when the L-*allo*-Ile and L-Ile were mixed prior to derivatization with Sanger's reagent. The HPLC ratios were calculated from the areas shown in the preceding tables where the lower area was normalized to 1.0 in the ratio. %Error = $|1.0 - x| / 1.0)$ where x is the value deviating from 1.0 in the in the HPLC measured ratio.

	Mixture Ratio	m/z HPLC Ratio	VWD HPLC Ratio
Ratio	1.0 : 1.0	1.0 : 1.11	1.0 : 1.01
%Error from Mixture Ratio		11%	1.0%

Calculation of Δt_R, Resolution (R_S), and Separation/Selectivity Factor (α) for the Separation of a 1:1 Mixture of L-*allo*-Isoleucine and L-Isoleucine After Derivatization with 1-Fluoro-2,4-dinitrobenzene (i.e., Sanger's reagent 10) with HPLC Method C (i.e., 30% aq. MeCN isocratic, 30 min.)

The table below includes the 340 nm VWD peak data used in the calculation of R_S and α. Where tm is the column void volume, k is the retention/capacity factor, and FWHM or $W_{0.5h}$ is the full width at half maximum. An observed t_M of 0.480 minutes was used in the calculation of both $t_R - t_M$ and k, where k equals $(t_R - t_M) / t_M$.

Peak	Assignment	t_R (min)	$t_R - t_M$ (min)	k	FWHM
1	L-*allo*-Ile	15.122	14.462	30.5	0.416
2	L-Ile	15.958	15.478	32.2	0.465

Δt_R = 15.958 – 15.122 = 0.836 minutes

α = 32.2 / 30.5 = 1.06

R_S = 1.18 * 0.836 / (0.416 + 0.465) = 1.12

LC/MS Traces for the Attempted Resolution of a 10:1 Mixture of L-*allo*-Isoleucine and L-Isoleucine After Derivatization with 1-Fluoro-2,4-dinitrobenzene (i.e., Sanger's Reagent 10) with HPLC Method C (i.e., 30% aq. MeCN isocratic, 30 min.)

A 10:1 mixture of L-*allo*-Ile and L-Ile (total amino acid concentration = 10 mM) was derivatized with Sanger's reagent under the standard Marfey's derivatization protocol (cf. General Procedure B, replacing phenylserine with the amino acid solution described above). The HPLC method used to resolve the derivatized amino acids was isocratic 30% MeCN in H_2O + 0.1% HCO_2H over 30 minutes with a flow rate 0.35 mL min^{-1} and using an Agilent Zorbax Extend-C18 column, 2.1 x 50 mm (1.8 µm). The Agilent column part number is 727700-902.

Total Ion Mass Chromatogram (ESI-ToF). The Y-axis is ion counts, and the X-axis is acquisition time in minutes.

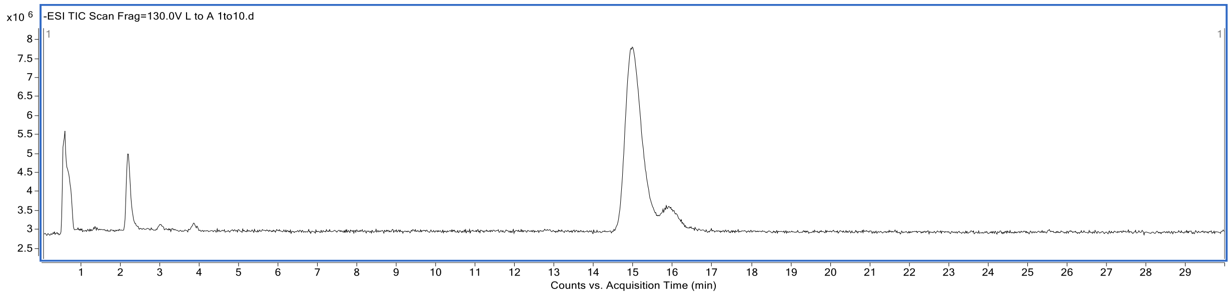

Variable Wavelength Detector Chromatogram (340 nm). The Y-axis is absorbance units, and the X-axis is acquisition time in minutes.

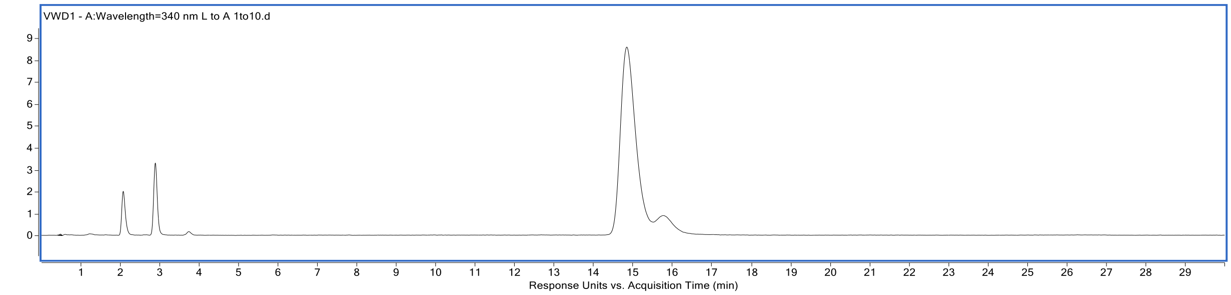

Extracted Ion Mass Chromatogram (ESI-ToF, extracted for m/z 296 ± 0.5). The Y-axis is ion counts, and the X-axis is acquisition time in minutes.

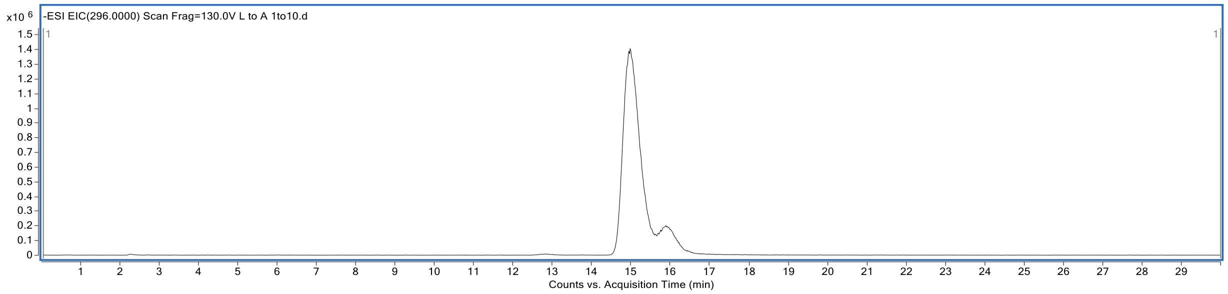

Zoomed and rescaled Extracted Ion (top) and Variable Wavelength Detector (bottom) Chromatograms. The Y axis on top is ion counts, the Y-axis on the bottom is absorbance units, and the X-axis for both is acquisition time in minutes. The peaks are labeled from left to right as Peak 1 and Peak 2.

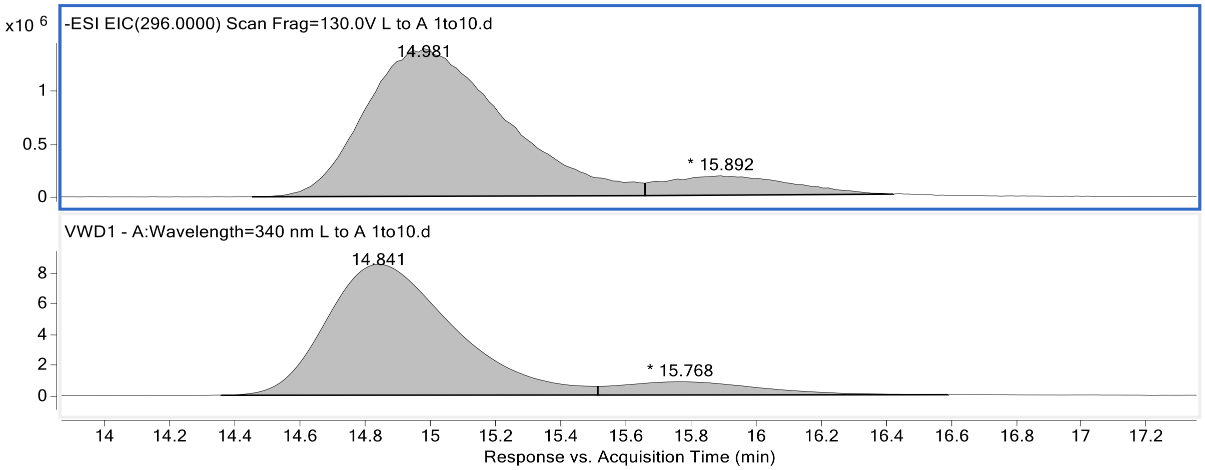

The following is a table of the peak data from the mass chromatogram. The Area % is the percent area relative to the tallest integrated peak, which has been set to 100%.

Peak	Assignment	t_R (min)	Area %
1	L-*allo*-Ile	14.981	100
2	L-Ile	15.892	11.90

The calculated m/z for the adduct of isoleucine with Sanger's reagent is 296.0888 for $C_{12}H_{14}N_3O_6^-$ $[M - H]^{-1}$.

The following is a table of the peak data from the variable wavelength detector chromatogram (340 nm). The Area % is the percent area relative to the tallest integrated peak, which has been set to 100%.

Peak	Assignment	t_R (min)	Area %
1	L-*allo*-Ile	14.841	100
2	L-Ile	15.768	11.15

The following is a table showing the difference between the experimental ratio of L-*allo*-Ile to L-Ile and the ratio measured by HPLC using extracted ion mass spectrometry (i.e., m/z HPLC Ratio) and 340 nm variable wavelength (i.e., VWD HPLC Ratio) detection. The mixture ratio was set when the L-*allo*-Ile and L-Ile were mixed prior to derivatization with Sanger's reagent. The HPLC ratios were calculated from the areas shown in the preceding tables where the lower area was normalized to 1.0 in the ratio. %Error = $|10 - x| / 10)$ where x is the value deviating from 10 in the in the HPLC measured ratio.

	Mixture Ratio	m/z HPLC Ratio	VWD HPLC Ratio
Ratio	10. : 1.0	8.40 : 1.0	8.97 : 1.0
%Error from Mixture Ratio		16%	10.%

LC/MS Traces for the Attempted Resolution of a 2:1 Mixture of L-*allo*-Isoleucine and L-Isoleucine After Derivatization with 1-Fluoro-2,4-dinitrobenzene (i.e., Sanger's Reagent 10) with HPLC Method C (i.e., 30% aq. MeCN isocratic, 30 min.)

A 2:1 mixture of L-*allo*-Ile and L-Ile (total amino acid concentration = 10 mM) was derivatized with Sanger's reagent under the standard Marfey's derivatization protocol (cf. General Procedure B, replacing phenylserine with the amino acid solution described above). The HPLC method used to resolve the derivatized amino acids was isocratic 30% MeCN in H_2O + 0.1% HCO_2H over 30 minutes with a flow rate 0.35 mL min^{-1} and using an Agilent Zorbax Extend-C18 column, 2.1 x 50 mm (1.8 µm). The Agilent column part number is 727700-902.

Total Ion Mass Chromatogram (ESI-ToF). The Y-axis is ion counts, and the X-axis is acquisition time in minutes.

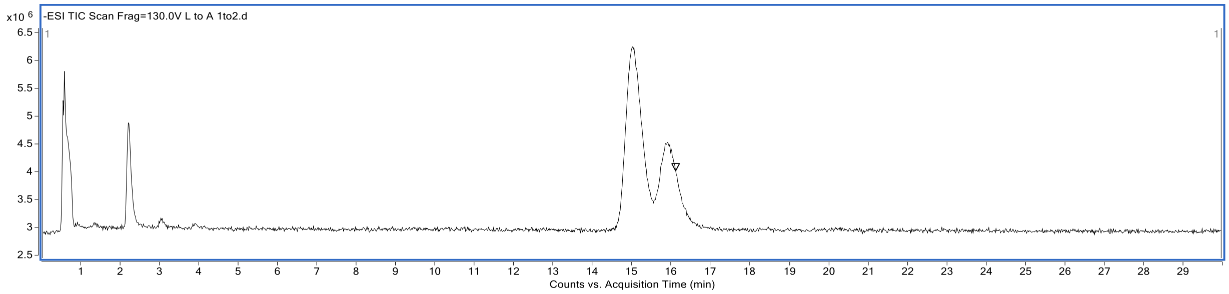

Variable Wavelength Detector Chromatogram (340 nm). The Y-axis is absorbance units, and the X-axis is acquisition time in minutes.

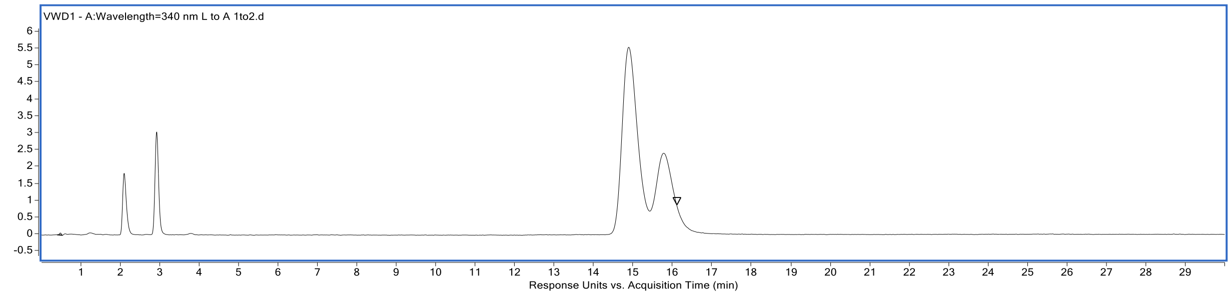

Extracted Ion Mass Chromatogram (ESI-ToF, extracted for m/z 296 ± 0.5). The Y-axis is ion counts, and the X-axis is acquisition time in minutes.

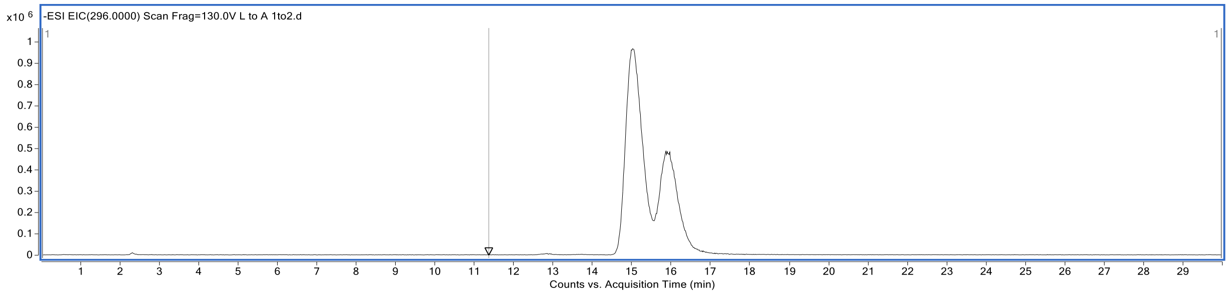

Zoomed and rescaled Extracted Ion (top) and Variable Wavelength Detector (bottom) Chromatograms. The Y axis on top is ion counts, the Y-axis on the bottom is absorbance units, and the X-axis for both is acquisition time in minutes. The peaks are labeled from left to right as Peak 1 and Peak 2.

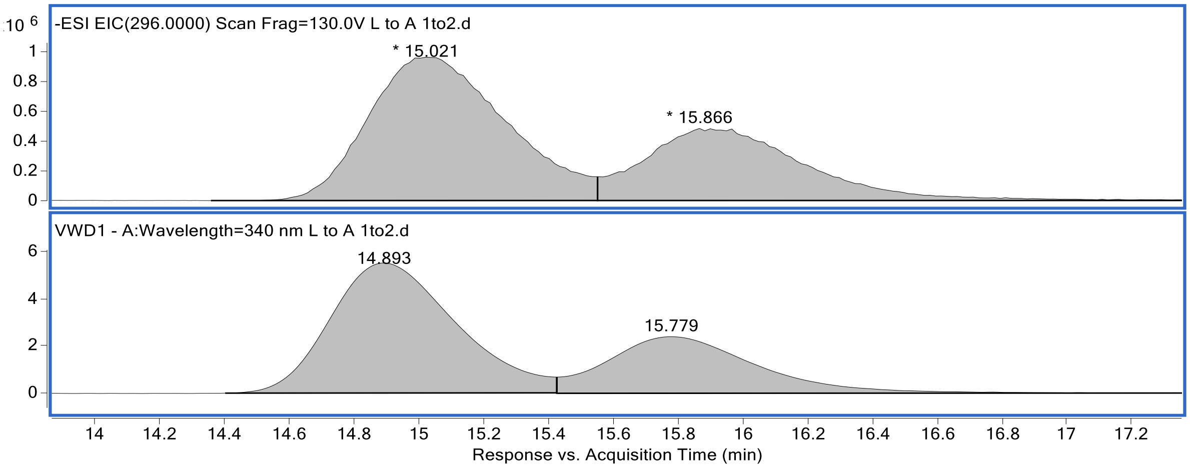

The following is a table of the peak data from the mass chromatogram. The Area % is the percent area relative to the tallest integrated peak, which has been set to 100%.

Peak	Assignment	t_R (min)	Area %
1	L-*allo*-Ile	15.021	58.82
2	L-Ile	15.866	100

The calculated m/z for the adduct of isoleucine with Sanger's reagent is 296.0888 for $C_{12}H_{14}N_3O_6^-$ $[M-H]^{-1}$.

The following is a table of the peak data from the variable wavelength detector chromatogram (340 nm). The Area % is the percent area relative to the tallest integrated peak, which has been set to 100%.

Peak	Assignment	t_R (min)	Area %
1	L-*allo*-Ile	14.893	50.53
2	L-Ile	15.779	100

The following is a table showing the difference between the experimental ratio of L-*allo*-Ile to L-Ile and the ratio measured by HPLC using extracted ion mass spectrometry (i.e., m/z HPLC Ratio) and 340 nm variable wavelength (i.e., VWD HPLC Ratio) detection. The mixture ratio was set when the L-*allo*-Ile and L-Ile were mixed prior to derivatization with Sanger's reagent. The HPLC ratios were calculated from the areas shown in the preceding tables where the lower area was normalized to 1.0 in the ratio. %Error = |2.0 – *x*| / 2.0) where *x* is the value deviating from 2.0 in the in the HPLC measured ratio.

	Mixture Ratio	m/z HPLC Ratio	VWD HPLC Ratio
Ratio	2.0 : 1.0	1.70 : 1.0	1.98 : 1.0
%Error from Mixture Ratio		15%	1.0%

LC/MS Traces for the Attempted Resolution of a 1:2 Mixture of L-*allo*-Isoleucine and L-Isoleucine After Derivatization with 1-Fluoro-2,4-dinitrobenzene (i.e., Sanger's Reagent 10) with HPLC Method C (i.e., 30% aq. MeCN isocratic, 30 min.)

A 1:2 mixture of L-*allo*-Ile and L-Ile (total amino acid concentration = 10 mM) was derivatized with Sanger's reagent under the standard Marfey's derivatization protocol (cf. General Procedure B, replacing phenylserine with the amino acid solution described above). The HPLC method used to resolve the derivatized amino acids was isocratic 30% MeCN in H_2O + 0.1% HCO_2H over 30 minutes with a flow rate 0.35 mL min^{-1} and using an Agilent Zorbax Extend-C18 column, 2.1 x 50 mm (1.8 µm). The Agilent column part number is 727700-902.

Total Ion Mass Chromatogram (ESI-ToF). The Y-axis is ion counts, and the X-axis is acquisition time in minutes.

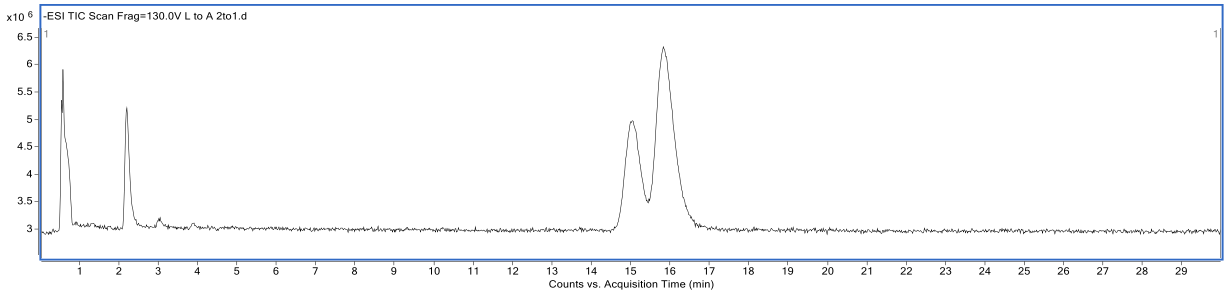

Variable Wavelength Detector Chromatogram (340 nm). The Y-axis is absorbance units, and the X-axis is acquisition time in minutes.

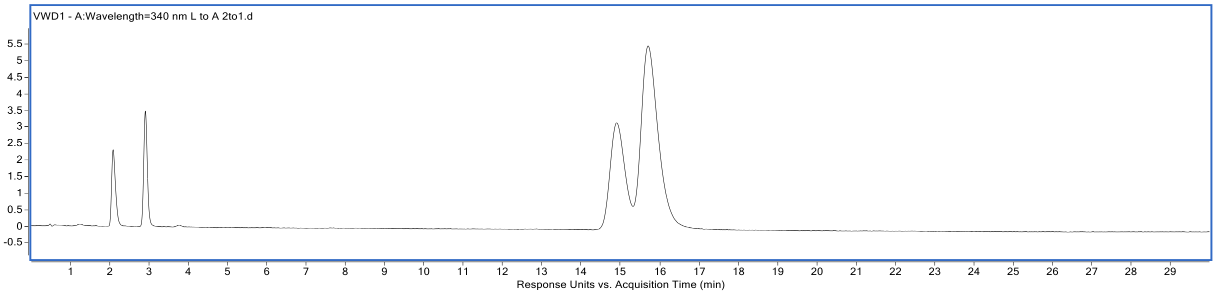

Extracted Ion Mass Chromatogram (ESI-ToF, extracted for m/z 296 ± 0.5). The Y-axis is ion counts, and the X-axis is acquisition time in minutes.

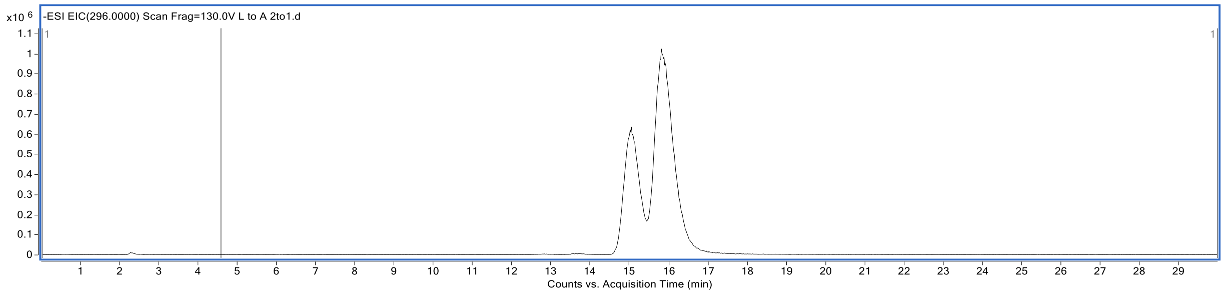

Zoomed and rescaled Extracted Ion (top) and Variable Wavelength Detector (bottom) Chromatograms. The Y axis on top is ion counts, the Y-axis on the bottom is absorbance units, and the X-axis for both is acquisition time in minutes. The peaks are labeled from left to right as Peak 1 and Peak 2.

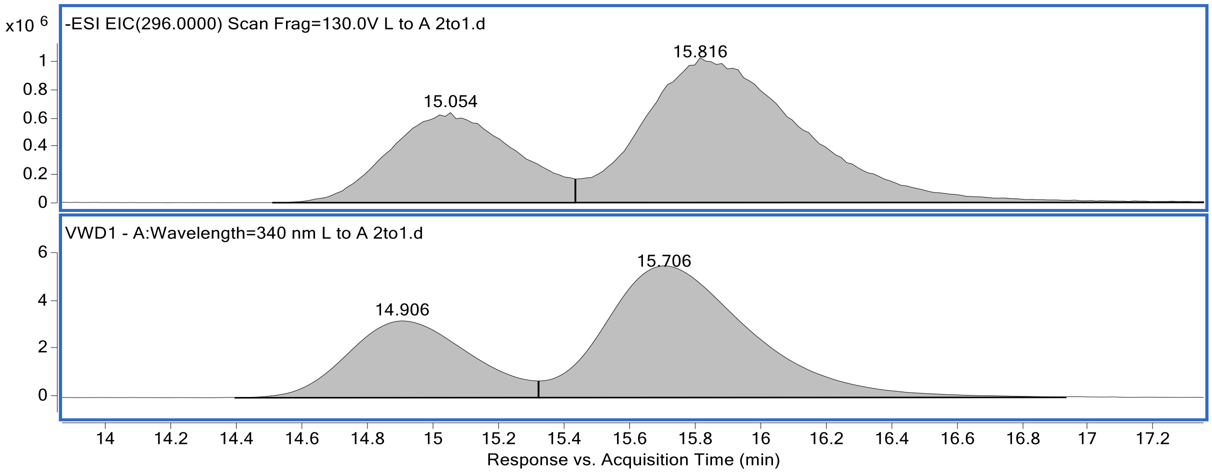

The following is a table of the peak data from the mass chromatogram. The Area % is the percent area relative to the tallest integrated peak, which has been set to 100%.

Peak	Assignment	t_R (min)	Area %
1	L-*allo*-Ile	15.054	48.96
2	L-Ile	15.816	100

The calculated m/z for the adduct of isoleucine with Sanger's reagent is 296.0888 for $C_{12}H_{14}N_3O_6^-$ $[M - H]^{-1}$.

The following is a table of the peak data from the variable wavelength detector chromatogram (340 nm). The Area % is the percent area relative to the tallest integrated peak, which has been set to 100%.

Peak	Assignment	t_R (min)	Area %
1	L-*allo*-Ile	14.906	49.46
2	L-Ile	15.706	100

The following is a table showing the difference between the experimental ratio of L-*allo*-Ile to L-Ile and the ratio measured by HPLC using extracted ion mass spectrometry (i.e., m/z HPLC Ratio) and 340 nm variable wavelength (i.e., VWD HPLC Ratio) detection. The mixture ratio was set when the L-*allo*-Ile and L-Ile were mixed prior to derivatization with Sanger's reagent. The HPLC ratios were calculated from the areas shown in the preceding tables where the lower area was normalized to 1.0 in the ratio. %Error = $|2.0 - x| / 2.0)$ where x is the value deviating from 2.0 in the in the HPLC measured ratio.

	Mixture Ratio	m/z HPLC Ratio	VWD HPLC Ratio
Ratio	1.0 : 2.0	1.0 : 2.04	1.0 : 2.02
%Error from Mixture Ratio		2.0%	1.1%

LC/MS Traces for the Attempted Resolution of a 1:10 Mixture of L-*allo*-Isoleucine and L-Isoleucine After Derivatization with 1-Fluoro-2,4-dinitrobenzene (i.e., Sanger's Reagent 10) with HPLC Method C (i.e., 30% aq. MeCN isocratic, 30 min.)

A 1:10 mixture of L-*allo*-Ile and L-Ile (total amino acid concentration = 10 mM) was derivatized with Sanger's reagent under the standard Marfey's derivatization protocol (cf. General Procedure B, replacing phenylserine with the amino acid solution described above). The HPLC method used to resolve the derivatized amino acids was isocratic 30% MeCN in H_2O + 0.1% HCO_2H over 30 minutes with a flow rate 0.35 mL min^{-1} and using an Agilent Zorbax Extend-C18 column, 2.1 x 50 mm (1.8 µm). The Agilent column part number is 727700-902.

Total Ion Mass Chromatogram (ESI-ToF). The Y-axis is ion counts, and the X-axis is acquisition time in minutes.

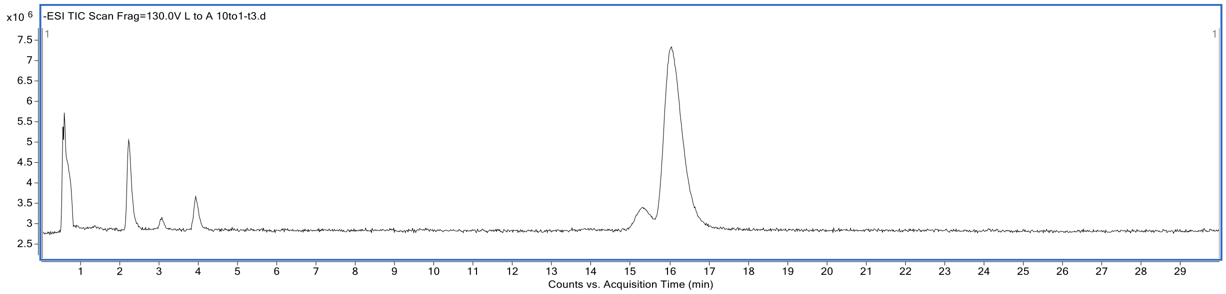

Variable Wavelength Detector Chromatogram (340 nm). The Y-axis is absorbance units, and the X-axis is acquisition time in minutes.

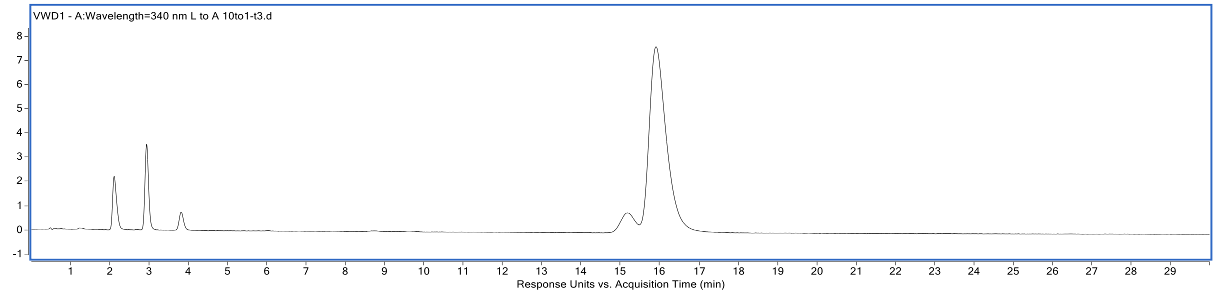

Extracted Ion Mass Chromatogram (ESI-ToF, extracted for m/z 296 ± 0.5). The Y-axis is ion counts, and the X-axis is acquisition time in minutes.

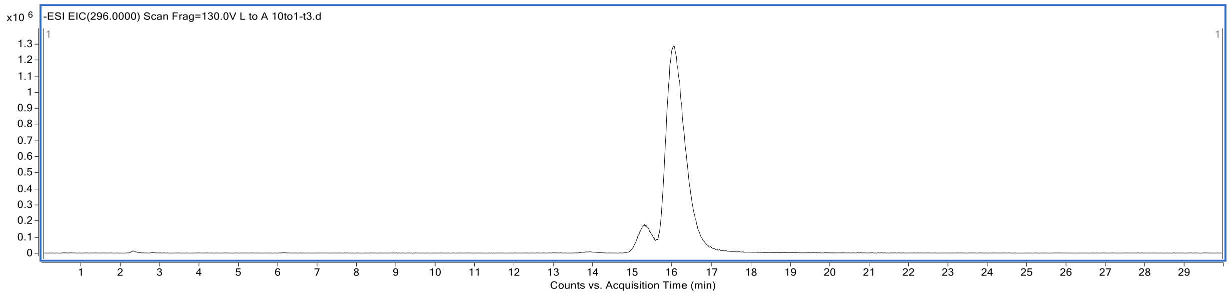

Zoomed and rescaled Extracted Ion (top) and Variable Wavelength Detector (bottom) Chromatograms. The Y axis on top is ion counts, the Y-axis on the bottom is absorbance units, and the X-axis for both is acquisition time in minutes. The peaks are labeled from left to right as Peak 1 and Peak 2.

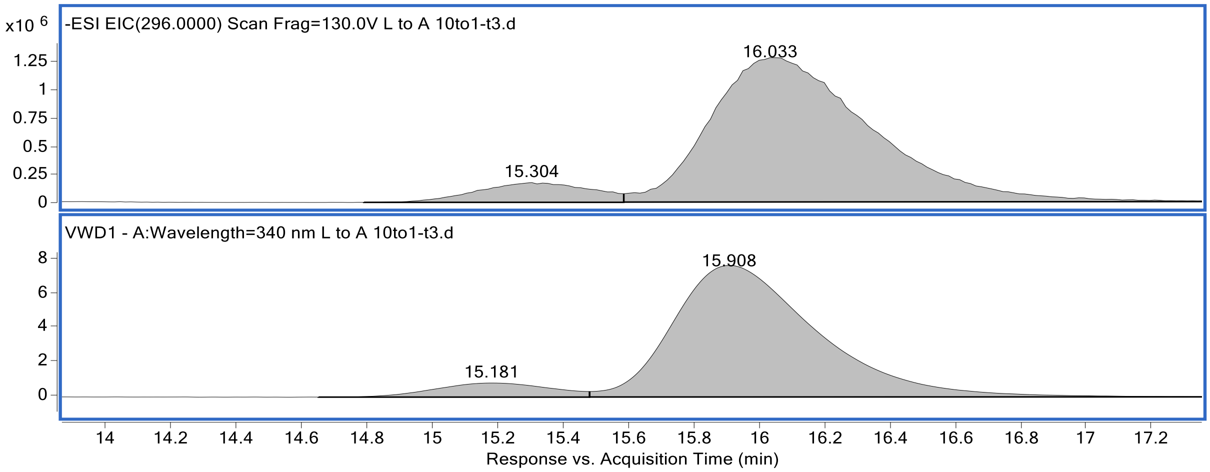

The following is a table of the peak data from the mass chromatogram. The Area % is the percent area relative to the tallest integrated peak, which has been set to 100%.

Peak	Assignment	t_R (min)	ES-ToF m/z [Neg]	Area %
1	L-*allo*-Ile	15.304	296.0897	8.70
2	L-Ile	16.033	296.0892	100

The calculated m/z for the adduct of isoleucine with Sanger's reagent is 296.0888 for $C_{12}H_{14}N_3O_6^-$ $[M - H]^{-1}$.

The following is a table of the peak data from the variable wavelength detector chromatogram (340 nm). The Area % is the percent area relative to the tallest integrated peak, which has been set to 100%.

Peak	Assignment	t_R (min)	Area %
1	L-*allo*-Ile	15.181	9.67
2	L-Ile	15.908	100

The following is a table showing the difference between the experimental ratio of L-*allo*-Ile to L-Ile and the ratio measured by HPLC using extracted ion mass spectrometry (i.e., m/z HPLC Ratio) and 340 nm variable wavelength (i.e., VWD HPLC Ratio) detection. The mixture ratio was set when the L-*allo*-Ile and L-Ile were mixed prior to derivatization with Sanger's reagent. The HPLC ratios were calculated from the areas shown in the preceding tables where the lower area was normalized to 1.0 in the ratio. %Error = |10. – *x*| / 10.) where *x* is the value deviating from 10. in the in the HPLC measured ratio.

	Mixture Ratio	m/z HPLC Ratio	VWD HPLC Ratio
Ratio	1.0 : 10.	1.0 : 11.5	1.0 : 10.3
%Error from Mixture Ratio		15%	3.4%

IV. References for Supporting Information

(1) Kakiuchi, R.; Hirayama, T.; Yanagisawa, D.; Tooyama, I.; Nagasawa, H. A ^{19}F-MRI Probe for the Detection of Fe(II) Ions in an Aqueous System. *Org Biomol Chem* **2020**, *18* (30), 5843–5849. https://doi.org/10.1039/D0OB00903B.

(2) Lanigan, R. M.; Karaluka, V.; Sabatini, M. T.; Starkov, P.; Badland, M.; Boulton, L.; Sheppard, T. D. Direct Amidation of Unprotected Amino Acids Using $B(OCH_2CF_3)_3$. *Chemical Communications* **2016**, *52* (57), 8846–8849. https://doi.org/10.1039/C6CC05147B.

(3) Kemker, I.; Schröder, D. C.; Feiner, R. C.; Müller, K. M.; Marion, A.; Sewald, N. Tuning the Biological Activity of RGD Peptides with Halotryptophans. *J Med Chem* **2021**, *64* (1), 586–601. https://doi.org/10.1021/acs.jmedchem.0c01536.

(4) Bhushan, R.; Kumar, V. Synthesis and Application of New Chiral Variants of Marfey's Reagent for Liquid Chromatographic Separation of the Enantiomers of α-Amino Acids. *Acta Chromatogr* **2008**, *20* (3). https://doi.org/10.1556/AChrom.20.2008.3.3.

(5) Salib, M. N.; Jamison, M. T.; Molinski, T. F. Bromo-Spiroisoxazoline Alkaloids, Including an Isoserine Peptide, from the Caribbean Marine Sponge *Aplysina lacunosa*. *J Nat Prod* **2020**, *83* (5), 1532–1540. https://doi.org/10.1021/acs.jnatprod.9b01286.

(6) Bruffy, S. K.; Meza, A.; Soler, J.; Doyon, T. J.; Young, S. H.; Lim, J.; Huseth, K. G.; Willoughby, P. H.; Garcia-Borràs, M.; Buller, A. R. Biocatalytic Asymmetric Aldol Addition into Unactivated Ketones. *Nat Chem* **2024**, *16* (12), 2076–2083. https://doi.org/10.1038/s41557-024-01647-1.

(7) Berrino, E.; Cantin, T.; Artault, M.; Beck, S.; Jessen, C.; Marrot, J.; Guégan, F.; Mingot, A.; Kornath, A.; Thibaudeau, S. Accumulation, Characterization and Reactivity of Chiral Ammonium-Carboxonium Dications in Superacid. *Angew Chem, Int Ed* **2024**, *63* (23). https://doi.org/10.1002/anie.202404066.

(8) Le, D. N.; Riedel, J.; Kozlyuk, N.; Martin, R. W.; Dong, V. M. Cyclizing Pentapeptides: Mechanism and Application of Dehydrophenylalanine as a Traceless Turn-Inducer. *Org Lett* **2017**, *19* (1), 114–117. https://doi.org/10.1021/acs.orglett.6b03308.

(9) Doyon, T. J.; Kumar, P.; Thein, S.; Kim, M.; Stitgen, A.; Grieger, A. M.; Madigan, C.; Willoughby, P. H.; Buller, A. R. Scalable and Selective β-Hydroxy-α-Amino Acid Synthesis Catalyzed by Promiscuous L-Threonine Transaldolase ObiH. *ChemBioChem* **2022**, *23* (2). https://doi.org/10.1002/cbic.202100577.

(10) Marfey, P. Determination of D-Amino Acids. II. Use of a Bifunctional Reagent, 1,5-Difluoro-2,4-Dinitrobenzene. *Carlsberg Res Commun* **1984**, *49* (6), 591–596. https://doi.org/10.1007/BF02908688.

(11) Bhushan, R.; Brückner, H. Marfey's Reagent for Chiral Amino Acid Analysis: A Review. *Amino Acids* **2004**, *27* (3–4), 231–247. https://doi.org/10.1007/s00726-004-0118-0.
